# Diretriz Brasileira de Fibrilação Atrial – 2025

**DOI:** 10.36660/abc.20250618

**Published:** 2025-09-16

**Authors:** Fatima Dumas Cintra, Cristiano Faria Pisani, André Gustavo da Silva Rezende, Benhur Davi Henz, Luciana Vidal Armaganijan, Maurício Pimentel, Renato D. Lopes, Thais Aguiar do Nascimento, Adalberto Menezes Lorga, Afonso Luiz Tavares de Albuquerque, Alexsandro Fagundes, Almir Alamino Lacalle, Ana Luisa Calixto Rodrigues, Andre d’Avila, Angelo Amato Vincenzo De Paola, Anibal Pires Borges, Carlos Antônio Abunader Kalil, Carlos Eduardo de Souza Miranda, Carlos Eduardo Duarte, Carlos Manuel de Almeida Brandão, Dalmo Antonio R. Moreira, Dário Celestino Sobral, Denise Tessariol Hachul, Eduardo Benchimol Saad, Elerson Arfelli, Enrique Indalécio Pachón Mateo, Érika Olivier Vilela Bragança, Fernando Ribeiro de Moraes, Francisco Carlos da Costa Darrieux, Guilherme Fenelon, Gustavo Glotz de Lima, Jacob Atié, João Carlos Ferreira Leal, Jose Carlos Moura Jorge, José Carlos Pachón Mateos, José Marcos Moreira, José Tarcísio Medeiros de Vasconcelos, Leandro Ioschpe Zimerman, Luciana Sacilotto, Luiz Pereira de Magalhães, Luiz Roberto Leite da Silva, Marcelo Garcia Leal, Marcio Augusto Silva, Marcio Jansen de Oliveira Figueiredo, Martha Valéria Tavares Pinheiro, Mauricio Ibrahim Scanavacca, Olga Ferreira de Souza, Ricardo Alves da Costa, Ricardo Ryoshim Kuniyoshi, Rui Manuel de Sousa Sequeira Antunes de Almeida, Silvia Helena Cardoso Boghossian, Tan Chen Wu, Thiago da Rocha Rodrigues, Veridiana Silva de Andrade

**Affiliations:** 1 Universidade Federal de São Paulo Escola Paulista de Medicina São Paulo SP Brasil Universidade Federal de São Paulo, Escola Paulista de Medicina, São Paulo, SP – Brasil; 2 Hospital das Clínicas da Faculdade de Medicina da Universidade de São Paulo São Paulo SP Brasil Hospital das Clínicas da Faculdade de Medicina da Universidade de São Paulo (HCFMUSP), São Paulo, SP – Brasil; 3 da Universidade de Pernambuco PROCAPE (Pronto Socorro Cardiológico Universitário de Pernambuco Prof. Luiz Tavares) Recife PE Brasil PROCAPE (Pronto Socorro Cardiológico Universitário de Pernambuco Prof. Luiz Tavares), da Universidade de Pernambuco (UPE) Recife, PE – Brasil; 4 Instituto Brasília de Arritmia Cardíaca Brasília DF Brasil Instituto Brasília de Arritmia Cardíaca, Brasília, DF – Brasil; 5 Hospital do Coração do Brasil Brasília DF Brasil Hospital do Coração do Brasil, Brasília, DF – Brasil; 6 Instituto Dante Pazzanese de Cardiologia São Paulo SP Brasil Instituto Dante Pazzanese de Cardiologia, São Paulo, SP – Brasil; 7 Hospital de Clínicas de Porto Alegre Porto Alegre RS Brasil Hospital de Clínicas de Porto Alegre, Porto Alegre, RS – Brasil; 8 Duke University Medical Center Durham EUA Duke University Medical Center, Durham – EUA; 9 Hospital Santa Izabel Salvador BA Brasil Hospital Santa Izabel, Salvador, BA – Brasil; 10 Hospital São Rafael Salvador BA Brasil Hospital São Rafael, Salvador, BA – Brasil; 11 Hospital Ana Nery Salvador BA Brasil Hospital Ana Nery, Salvador, BA – Brasil; 12 Instituto de Moléstias Cardiovasculares São José de Rio Preto SP Brasil Instituto de Moléstias Cardiovasculares (IMC), São José de Rio Preto, SP – Brasil; 13 Hospital de Base da Faculdade de Medicina de S. J. Rio Preto – FAMERP São José de Rio Preto SP Brasil Hospital de Base da Faculdade de Medicina de S. J. Rio Preto – FAMERP, São José de Rio Preto, SP – Brasil; 14 Universidade do Estado da Bahia Salvador BA Brasil Universidade do Estado da Bahia, Salvador, BA – Brasil; 15 Hospital das Clínicas da Faculdade de Medicina da Universidade de São Paulo de Ribeirão Preto Ribeirão Preto SP Brasil Hospital das Clínicas da Faculdade de Medicina da Universidade de São Paulo de Ribeirão Preto (HCRPUSP), Ribeirão Preto, SP – Brasil; 16 Santa Casa de Votuporanga Votuporanga SP Brasil Santa Casa de Votuporanga, Votuporanga, SP – Brasil; 17 Hospital Felício Rocho Belo Horizonte MG Brasil Hospital Felício Rocho, Belo Horizonte, MG – Brasil; 18 Hospital SOS Cardio Florianópolis SC Brasil Hospital SOS Cardio, Florianópolis, SC – Brasil; 19 Santa Casa de Misericórdia de Porto Alegre Porto Alegre RS Brasil Santa Casa de Misericórdia de Porto Alegre, Porto Alegre, RS – Brasil; 20 Hospital de Clínicas de Porto Alegre Porto Alegre RS Brasil Hospital de Clínicas de Porto Alegre, Porto Alegre, RS – Brasil; 21 Hospital Madre Teresa Belo Horizonte MG Brasil Hospital Madre Teresa, Belo Horizonte, MG – Brasil; 22 Hospital Beneficência Portuguesa de São Paulo São Paulo SP Brasil Hospital Beneficência Portuguesa de São Paulo, São Paulo, SP – Brasil; 23 Hospital das Clínicas da Faculdade de Medicina da Universidade de São Paulo Instituto do Coração São Paulo SP Brasil Instituto do Coração (Incor) do Hospital das Clínicas da Faculdade de Medicina da Universidade de São Paulo (HCFMUSP), São Paulo, SP – Brasil; 24 Universidade de Pernambuco Recife PE Brasil Universidade de Pernambuco, Recife, PE – Brasil; 25 Hospital Samaritano Rio de Janeiro RJ Brasil Hospital Samaritano, Rio de Janeiro, RJ – Brasil; 26 Hospital do Coração São Paulo SP Brasil Hospital do Coração (HCor), São Paulo, SP – Brasil; 27 RitmoCheck São José dos Campos SP Brasil RitmoCheck, São José dos Campos, SP – Brasil; 28 Universidade Federal de Pernambuco Recife PE Brasil Universidade Federal de Pernambuco, Recife, PE – Brasil; 29 Hospital Israelita Albert Einstein São Paulo SP Brasil Hospital Israelita Albert Einstein, São Paulo, SP – Brasil; 30 Instituto de Cardiologia do Rio Grande do Sul Porto Alegre RS Brasil Instituto de Cardiologia do Rio Grande do Sul, Porto Alegre, RS – Brasil; 31 Universidade Federal do Rio de Janeiro Rio de Janeiro RJ Brasil Universidade Federal do Rio de Janeiro (UFRJ), Rio de Janeiro RJ – Brasil; 32 Faculdade Estadual de Medicina de São José do Rio Preto São José do Rio Preto SP Brasil Faculdade Estadual de Medicina de São José do Rio Preto (FAMERP), São José do Rio Preto, SP – Brasil; 33 Hospital Beneficência Portuguesa de São José do Rio Preto São José do Rio Preto SP Brasil Hospital Beneficência Portuguesa de São José do Rio Preto, São José do Rio Preto, SP – Brasil; 34 Pontifícia Universidade Católica do Paraná Curitiba PR Brasil Pontifícia Universidade Católica do Paraná, Curitiba, PR – Brasil; 35 SEMAP Arritmias Dr. Pachón São Paulo SP Brasil SEMAP Arritmias Dr. Pachón, São Paulo, SP – Brasil; 36 Hospital do Servidor Público Estadual de São Paulo São Paulo SP Brasil Hospital do Servidor Público Estadual de São Paulo, São Paulo, SP – Brasil; 37 Hospital Moinhos de Vento Porto Alegre RS Brasil Hospital Moinhos de Vento, Porto Alegre, RS – Brasil; 38 Hospital Universitário Professor Edgard Santos da Faculdade de Medicina da Universidade Federal da Bahia Salvador BA Brasil Hospital Universitário Professor Edgard Santos da Faculdade de Medicina da Universidade Federal da Bahia, Salvador, BA – Brasil; 39 Grupo Santa Hospitais Brasília DF Brasil Grupo Santa Hospitais, Brasília, DF – Brasil; 40 Vitória Apart Hospital Serra ES Brasil Vitória Apart Hospital, Serra, ES – Brasil; 41 Hospital Universitário Cassiano Antônio de Moraes Vitória ES Brasil Hospital Universitário Cassiano Antônio de Moraes (UFES), Vitória, ES – Brasil; 42 Unimed Campinas Campinas SP Brasil Unimed Campinas, Campinas, SP – Brasil; 43 RedeD’Or Hospitais Rio de Janeiro RJ Brasil RedeD’Or Hospitais, Rio de Janeiro RJ – Brasil; 44 Centrocor Vitória Vitória ES Brasil Centrocor Vitória, Vitória, ES – Brasil; 45 Centro Universitário Assis Gurgacz Cascavel PR Brasil Centro Universitário Assis Gurgacz, Cascavel, PR – Brasil; 46 Universidade do Estado do Rio de Janeiro Rio de Janeiro RJ Brasil Universidade do Estado do Rio de Janeiro (UERJ), Rio de Janeiro RJ – Brasil

**Table:** 

Diretriz Brasileira de Fibrilação Atrial – 2025	
O relatório abaixo lista as declarações de interesse conforme relatadas à SBC pelos especialistas durante o período de desenvolvimento deste posicionamento, 2023/2025.	
Especialista	Tipo de relacionamento com a indústria
Adalberto Menezes Lorga Filho	Declaração financeira A - Pagamento de qualquer espécie e desde que economicamente apreciáveis, feitos a (i) você, (ii) ao seu cônjuge/ companheiro ou a qualquer outro membro que resida com você, (iii) a qualquer pessoa jurídica em que qualquer destes seja controlador, sócio, acionista ou participante, de forma direta ou indireta, recebimento por palestras, aulas, atuação como proctor de treinamentos, remunerações, honorários pagos por participações em conselhos consultivos, de investigadores, ou outros comitês, etc. Provenientes da indústria farmacêutica, de órteses, próteses, equipamentos e implantes, brasileiras ou estrangeiras: - Libbs, Daiichi Sankyo: fibrilação atrial.
Afonso Luiz Tavares de Albuquerque	Nada a ser declarado
Alexsandro Fagundes	Nada a ser declarado
Almir Alamino Lacalle	Nada a ser declarado
Ana Luisa Calixto Rodrigues	Nada a ser declarado
Andre D'Avila	Nada a ser declarado
André Gustavo da Silva Rezende	Outros relacionamentos Atuação no último ano como auditor médico para empresa operadora de planos de saúde ou assemelhada: - Parecerista técnico na área de Arritmias Cardíacas nas operadoras Hapvida, Notre Dame, Intermedicas.
Angelo Amato Vincenzo de Paola	Nada a ser declarado
Anibal Pires Borges	Declaração financeira A - Pagamento de qualquer espécie e desde que economicamente apreciáveis, feitos a (i) você, (ii) ao seu cônjuge/ companheiro ou a qualquer outro membro que resida com você, (iii) a qualquer pessoa jurídica em que qualquer destes seja controlador, sócio, acionista ou participante, de forma direta ou indireta, recebimento por palestras, aulas, atuação como proctor de treinamentos, remunerações, honorários pagos por participações em conselhos consultivos, de investigadores, ou outros comitês, etc. Provenientes da indústria farmacêutica, de órteses, próteses, equipamentos e implantes, brasileiras ou estrangeiras: - Jonhson & Johnson: palestras. Outros relacionamentos Financiamento de atividades de educação médica continuada, incluindo viagens, hospedagens e inscrições para congressos e cursos, provenientes da indústria farmacêutica, de órteses, próteses, equipamentos e implantes, brasileiras ou estrangeiras: - Johnson & Johnson: eventos próprios da empresa.
Benhur Davi Henz	Nada a ser declarado
Carlos Antônio Abunader Kalil	Declaração financeira A - Pagamento de qualquer espécie e desde que economicamente apreciáveis, feitos a (i) você, (ii) ao seu cônjuge/ companheiro ou a qualquer outro membro que resida com você, (iii) a qualquer pessoa jurídica em que qualquer destes seja controlador, sócio, acionista ou participante, de forma direta ou indireta, recebimento por palestras, aulas, atuação como proctor de treinamentos, remunerações, honorários pagos por participações em conselhos consultivos, de investigadores, ou outros comitês, etc. Provenientes da indústria farmacêutica, de órteses, próteses, equipamentos e implantes, brasileiras ou estrangeiras: - Biotronik, ABBOTT, Boston Sc, Johnson & Johnson: palestras Outros relacionamentos Financiamento de atividades de educação médica continuada, incluindo viagens, hospedagens e inscrições para congressos e cursos, provenientes da indústria farmacêutica, de órteses, próteses, equipamentos e implantes, brasileiras ou estrangeiras: - Biotronik, ABBOTT, Boston Sc, Johnson & Jonhson: eventos
Carlos Eduardo de Souza Miranda	Declaração financeira A - Pagamento de qualquer espécie e desde que economicamente apreciáveis, feitos a (i) você, (ii) ao seu cônjuge/ companheiro ou a qualquer outro membro que resida com você, (iii) a qualquer pessoa jurídica em que qualquer destes seja controlador, sócio, acionista ou participante, de forma direta ou indireta, recebimento por palestras, aulas, atuação como proctor de treinamentos, remunerações, honorários pagos por participações em conselhos consultivos, de investigadores, ou outros comitês, etc. Provenientes da indústria farmacêutica, de órteses, próteses, equipamentos e implantes, brasileiras ou estrangeiras: - Laboratório: Área de atuação/Medicamento/Atividade. - Daiichi Sankyo: Lixiana.
Carlos Eduardo Duarte	Declaração financeira A - Pagamento de qualquer espécie e desde que economicamente apreciáveis, feitos a (i) você, (ii) ao seu cônjuge/ companheiro ou a qualquer outro membro que resida com você, (iii) a qualquer pessoa jurídica em que qualquer destes seja controlador, sócio, acionista ou participante, de forma direta ou indireta, recebimento por palestras, aulas, atuação como proctor de treinamentos, remunerações, honorários pagos por participações em conselhos consultivos, de investigadores, ou outros comitês, etc. Provenientes da indústria farmacêutica, de órteses, próteses, equipamentos e implantes, brasileiras ou estrangeiras: - Medtronic: Micra Estimulação Cardíaca Artificial.
Carlos Manuel de Almeida Brandão	Nada a ser declarado
Cristiano Faria Pisani	Declaração financeira A - Pagamento de qualquer espécie e desde que economicamente apreciáveis, feitos a (i) você, (ii) ao seu cônjuge/ companheiro ou a qualquer outro membro que resida com você, (iii) a qualquer pessoa jurídica em que qualquer destes seja controlador, sócio, acionista ou participante, de forma direta ou indireta, recebimento por palestras, aulas, atuação como proctor de treinamentos, remunerações, honorários pagos por participações em conselhos consultivos, de investigadores, ou outros comitês, etc. Provenientes da indústria farmacêutica, de órteses, próteses, equipamentos e implantes, brasileiras ou estrangeiras: - Laboratório: Área de atuação/Medicamento/Atividade. - Johnson & Johnson.
Dalmo Antonio R. Moreira	Nada a ser declarado
Dário Celestino Sobral Filho	Nada a ser declarado
Denise Tessariol Hachul	Nada a ser declarado
Eduardo Benchimol Saad	Declaração financeira A - Pagamento de qualquer espécie e desde que economicamente apreciáveis, feitos a (i) você, (ii) ao seu cônjuge/ companheiro ou a qualquer outro membro que resida com você, (iii) a qualquer pessoa jurídica em que qualquer destes seja controlador, sócio, acionista ou participante, de forma direta ou indireta, recebimento por palestras, aulas, atuação como proctor de treinamentos, remunerações, honorários pagos por participações em conselhos consultivos, de investigadores, ou outros comitês, etc. Provenientes da indústria farmacêutica, de órteses, próteses, equipamentos e implantes, brasileiras ou estrangeiras: - Abbott; J&J.
Elerson Arfelli	Declaração financeira A - Pagamento de qualquer espécie e desde que economicamente apreciáveis, feitos a (i) você, (ii) ao seu cônjuge/ companheiro ou a qualquer outro membro que resida com você, (iii) a qualquer pessoa jurídica em que qualquer destes seja controlador, sócio, acionista ou participante, de forma direta ou indireta, recebimento por palestras, aulas, atuação como proctor de treinamentos, remunerações, honorários pagos por participações em conselhos consultivos, de investigadores, ou outros comitês, etc. Provenientes da indústria farmacêutica, de órteses, próteses, equipamentos e implantes, brasileiras ou estrangeiras: - Daiichi Sankyo.
Enrique Indalécio Pachón Mateo	Nada a ser declarado
Érika Olivier Vilela Bragança	Declaração financeira A - Pagamento de qualquer espécie e desde que economicamente apreciáveis, feitos a (i) você, (ii) ao seu cônjuge/ companheiro ou a qualquer outro membro que resida com você, (iii) a qualquer pessoa jurídica em que qualquer destes seja controlador, sócio, acionista ou participante, de forma direta ou indireta, recebimento por palestras, aulas, atuação como proctor de treinamentos, remunerações, honorários pagos por participações em conselhos consultivos, de investigadores, ou outros comitês, etc. Provenientes da indústria farmacêutica, de órteses, próteses, equipamentos e implantes, brasileiras ou estrangeiras: - Biolab: Dozoito. Outros relacionamentos Financiamento de atividades de educação médica continuada, incluindo viagens, hospedagens e inscrições para congressos e cursos, provenientes da indústria farmacêutica, de órteses, próteses, equipamentos e implantes, brasileiras ou estrangeiras: - Merck.
Fatima Dumas Cintra	Outros relacionamentos Financiamento de atividades de educação médica continuada, incluindo viagens, hospedagens e inscrições para congressos e cursos, provenientes da indústria farmacêutica, de órteses, próteses, equipamentos e implantes, brasileiras ou estrangeiras: - Takeda. Participação societária de qualquer natureza e qualquer valor economicamente apreciável de empresas na área de saúde, de ensino ou em empresas concorrentes ou fornecedoras da SBC: - Área da saúde.
Fernando Ribeiro de Moraes Neto	Declaração financeira A - Pagamento de qualquer espécie e desde que economicamente apreciáveis, feitos a (i) você, (ii) ao seu cônjuge/ companheiro ou a qualquer outro membro que resida com você, (iii) a qualquer pessoa jurídica em que qualquer destes seja controlador, sócio, acionista ou participante, de forma direta ou indireta, recebimento por palestras, aulas, atuação como proctor de treinamentos, remunerações, honorários pagos por participações em conselhos consultivos, de investigadores, ou outros comitês, etc. Provenientes da indústria farmacêutica, de órteses, próteses, equipamentos e implantes, brasileiras ou estrangeiras: - Edwards: Bioprótese, troca valvar. Outros relacionamentos Financiamento de atividades de educação médica continuada, incluindo viagens, hospedagens e inscrições para congressos e cursos, provenientes da indústria farmacêutica, de órteses, próteses, equipamentos e implantes, brasileiras ou estrangeiras: - Edwards: educação médica continuada.
Francisco Carlos da Costa Darrieux	Declaração financeira A - Pagamento de qualquer espécie e desde que economicamente apreciáveis, feitos a (i) você, (ii) ao seu cônjuge/ companheiro ou a qualquer outro membro que resida com você, (iii) a qualquer pessoa jurídica em que qualquer destes seja controlador, sócio, acionista ou participante, de forma direta ou indireta, recebimento por palestras, aulas, atuação como proctor de treinamentos, remunerações, honorários pagos por participações em conselhos consultivos, de investigadores, ou outros comitês, etc. Provenientes da indústria farmacêutica, de órteses, próteses, equipamentos e implantes, brasileiras ou estrangeiras: - Laboratório: Área de atuação/Medicamento/Atividade. - Daichii Sankyo; Adium; Aché.
Guilherme Fenelon	Outros relacionamentos Financiamento de atividades de educação médica continuada, incluindo viagens, hospedagens e inscrições para congressos e cursos, provenientes da indústria farmacêutica, de órteses, próteses, equipamentos e implantes, brasileiras ou estrangeiras: - Johnson & Johnson: ablação por cateter.
Gustavo Glotz de Lima	Declaração financeira A - Pagamento de qualquer espécie e desde que economicamente apreciáveis, feitos a (i) você, (ii) ao seu cônjuge/ companheiro ou a qualquer outro membro que resida com você, (iii) a qualquer pessoa jurídica em que qualquer destes seja controlador, sócio, acionista ou participante, de forma direta ou indireta, recebimento por palestras, aulas, atuação como proctor de treinamentos, remunerações, honorários pagos por participações em conselhos consultivos, de investigadores, ou outros comitês, etc. Provenientes da indústria farmacêutica, de órteses, próteses, equipamentos e implantes, brasileiras ou estrangeiras: - Abbott: arritmias. Outros relacionamentos Financiamento de atividades de educação médica continuada, incluindo viagens, hospedagens e inscrições para congressos e cursos, provenientes da indústria farmacêutica, de órteses, próteses, equipamentos e implantes, brasileiras ou estrangeiras: - Abbott: arritmias.
Jacob Atié	Declaração financeira A - Pagamento de qualquer espécie e desde que economicamente apreciáveis, feitos a (i) você, (ii) ao seu cônjuge/ companheiro ou a qualquer outro membro que resida com você, (iii) a qualquer pessoa jurídica em que qualquer destes seja controlador, sócio, acionista ou participante, de forma direta ou indireta, recebimento por palestras, aulas, atuação como proctor de treinamentos, remunerações, honorários pagos por participações em conselhos consultivos, de investigadores, ou outros comitês, etc. Provenientes da indústria farmacêutica, de órteses, próteses, equipamentos e implantes, brasileiras ou estrangeiras: - Boston Scientific, Johnson & Johnson: palestras sobre fibrilação atrial. Outros relacionamentos Financiamento de atividades de educação médica continuada, incluindo viagens, hospedagens e inscrições para congressos e cursos, provenientes da indústria farmacêutica, de órteses, próteses, equipamentos e implantes, brasileiras ou estrangeiras: - Boston Scientific, Johnson & Johnson: PFA ablação de FA.
João Carlos Ferreira Leal	Declaração financeira A - Pagamento de qualquer espécie e desde que economicamente apreciáveis, feitos a (i) você, (ii) ao seu cônjuge/ companheiro ou a qualquer outro membro que resida com você, (iii) a qualquer pessoa jurídica em que qualquer destes seja controlador, sócio, acionista ou participante, de forma direta ou indireta, recebimento por palestras, aulas, atuação como proctor de treinamentos, remunerações, honorários pagos por participações em conselhos consultivos, de investigadores, ou outros comitês, etc. Provenientes da indústria farmacêutica, de órteses, próteses, equipamentos e implantes, brasileiras ou estrangeiras: - Neomex/Artivion: prótese híbrida Evita open plus e On-X; Braile Biomédica: prótese de liberação rápida Alpha. B - Financiamento de pesquisas sob sua responsabilidade direta/pessoal (direcionado ao departamento ou instituição) provenientes da indústria farmacêutica, de órteses, próteses, equipamentos e implantes, brasileiras ou estrangeiras: - Braile Biomédica: PI do estudo prótese biológica Vivere. C - Financiamento de pesquisa (pessoal), cujas receitas tenham sido provenientes da indústria farmacêutica, de órteses, próteses, equipamentos e implantes, brasileiras ou estrangeiras: - Braile Biomédica: PI do estudo prótese biológica Vivere. Outros relacionamentos Financiamento de atividades de educação médica continuada, incluindo viagens, hospedagens e inscrições para congressos e cursos, provenientes da indústria farmacêutica, de órteses, próteses, equipamentos e implantes, brasileiras ou estrangeiras: - Neomex/Artivion; Braile Biomédica.
Jose Carlos Moura Jorge	Nada a ser declarado
José Carlos Pachón Mateos	Nada a ser declarado
José Marcos Moreira	Nada a ser declarado
José Tarcísio Medeiros de Vasconcelos	Declaração financeira A - Pagamento de qualquer espécie e desde que economicamente apreciáveis, feitos a (i) você, (ii) ao seu cônjuge/ companheiro ou a qualquer outro membro que resida com você, (iii) a qualquer pessoa jurídica em que qualquer destes seja controlador, sócio, acionista ou participante, de forma direta ou indireta, recebimento por palestras, aulas, atuação como proctor de treinamentos, remunerações, honorários pagos por participações em conselhos consultivos, de investigadores, ou outros comitês, etc. Provenientes da indústria farmacêutica, de órteses, próteses, equipamentos e implantes, brasileiras ou estrangeiras: - Boston Scientific, Abbott. B - Financiamento de pesquisas sob sua responsabilidade direta/pessoal (direcionado ao departamento ou instituição) provenientes da indústria farmacêutica, de órteses, próteses, equipamentos e implantes, brasileiras ou estrangeiras: - Daichi Sankyo. C - Financiamento de pesquisa (pessoal), cujas receitas tenham sido provenientes da indústria farmacêutica, de órteses, próteses, equipamentos e implantes, brasileiras ou estrangeiras: - Daichi Sankyo. Outros relacionamentos Financiamento de atividades de educação médica continuada, incluindo viagens, hospedagens e inscrições para congressos e cursos, provenientes da indústria farmacêutica, de órteses, próteses, equipamentos e implantes, brasileiras ou estrangeiras: - Boston Scientific, Abbott.
Leandro Ioschpe Zimerman	Outros relacionamentos Participação em comitês de compras de materiais ou fármacos em instituições de saúde ou funções assemelhadas: - Chefe da Unidade de Diagnóstico e Terapia Cardiovascular do Hospital de Clínicas de Porto Alegre; decisão sobre eventual compra de materiais.
Luciana Sacilotto	Nada a ser declarado
Luciana Vidal Armaganijan	Nada a ser declarado
Luiz Pereira de Magalhães	Nada a ser declarado
Luiz Roberto Leite da Silva	Outros relacionamentos Financiamento de atividades de educação médica continuada, incluindo viagens, hospedagens e inscrições para congressos e cursos, provenientes da indústria farmacêutica, de órteses, próteses, equipamentos e implantes, brasileiras ou estrangeiras: - Boisense Webster: ablação de fibrilação atrial.
Marcelo Garcia Leal	Nada a ser declarado
Marcio Augusto Silva	Nada a ser declarado
Marcio Jansen de Oliveira Figueiredo	Declaração financeira A - Pagamento de qualquer espécie e desde que economicamente apreciáveis, feitos a (i) você, (ii) ao seu cônjuge/ companheiro ou a qualquer outro membro que resida com você, (iii) a qualquer pessoa jurídica em que qualquer destes seja controlador, sócio, acionista ou participante, de forma direta ou indireta, recebimento por palestras, aulas, atuação como proctor de treinamentos, remunerações, honorários pagos por participações em conselhos consultivos, de investigadores, ou outros comitês, etc. Provenientes da indústria farmacêutica, de órteses, próteses, equipamentos e implantes, brasileiras ou estrangeiras: - Laboratório: Área de atuação/Medicamento/Atividade. - Abbott: Ritmonorm; EMS: Xakylis.
Martha Valéria Tavares Pinheiro	Outros relacionamentos Financiamento de atividades de educação médica continuada, incluindo viagens, hospedagens e inscrições para congressos e cursos, provenientes da indústria farmacêutica, de órteses, próteses, equipamentos e implantes, brasileiras ou estrangeiras: - Boston: Farapulse.
Mauricio Ibrahim Scanavacca	Declaração financeira A - Pagamento de qualquer espécie e desde que economicamente apreciáveis, feitos a (i) você, (ii) ao seu cônjuge/ companheiro ou a qualquer outro membro que resida com você, (iii) a qualquer pessoa jurídica em que qualquer destes seja controlador, sócio, acionista ou participante, de forma direta ou indireta, recebimento por palestras, aulas, atuação como proctor de treinamentos, remunerações, honorários pagos por participações em conselhos consultivos, de investigadores, ou outros comitês, etc. Provenientes da indústria farmacêutica, de órteses, próteses, equipamentos e implantes, brasileiras ou estrangeiras: - Daichii Sankyo: anticoagulação. B - Financiamento de pesquisas sob sua responsabilidade direta/pessoal (direcionado ao departamento ou instituição) provenientes da indústria farmacêutica, de órteses, próteses, equipamentos e implantes, brasileiras ou estrangeiras: - J&J: ablação de taquicardia ventricular. C - Financiamento de pesquisa (pessoal), cujas receitas tenham sido provenientes da indústria farmacêutica, de órteses, próteses, equipamentos e implantes, brasileiras ou estrangeiras: - ABBOT: ablação por cateter da cindam vaso vagal. Outros relacionamentos Financiamento de atividades de educação médica continuada, incluindo viagens, hospedagens e inscrições para congressos e cursos, provenientes da indústria farmacêutica, de órteses, próteses, equipamentos e implantes, brasileiras ou estrangeiras: - J&J: simpósio patrocinado pela indústria.
Maurício Pimentel	Declaração financeira A - Pagamento de qualquer espécie e desde que economicamente apreciáveis, feitos a (i) você, (ii) ao seu cônjuge/ companheiro ou a qualquer outro membro que resida com você, (iii) a qualquer pessoa jurídica em que qualquer destes seja controlador, sócio, acionista ou participante, de forma direta ou indireta, recebimento por palestras, aulas, atuação como proctor de treinamentos, remunerações, honorários pagos por participações em conselhos consultivos, de investigadores, ou outros comitês, etc. Provenientes da indústria farmacêutica, de órteses, próteses, equipamentos e implantes, brasileiras ou estrangeiras: - Laboratório: Área de atuação/Medicamento/Atividade. - Libbs: fibrilação atrial.
Olga Ferreira de Souza	Nada a ser declarado
Renato D. Lopes	Declaração financeira A - Pagamento de qualquer espécie e desde que economicamente apreciáveis, feitos a (i) você, (ii) ao seu cônjuge/ companheiro ou a qualquer outro membro que resida com você, (iii) a qualquer pessoa jurídica em que qualquer destes seja controlador, sócio, acionista ou participante, de forma direta ou indireta, recebimento por palestras, aulas, atuação como proctor de treinamentos, remunerações, honorários pagos por participações em conselhos consultivos, de investigadores, ou outros comitês, etc. Provenientes da indústria farmacêutica, de órteses, próteses, equipamentos e implantes, brasileiras ou estrangeiras: - Pfizer, Daiichi Sankyo, Novo Nordisk, Bayer, Boehringer Ingelheim, Bristol-Myers Squibb. B - Financiamento de pesquisas sob sua responsabilidade direta/pessoal (direcionado ao departamento ou instituição) provenientes da indústria farmacêutica, de órteses, próteses, equipamentos e implantes, brasileiras ou estrangeiras: - Amgen, Bristol-Myers Squibb, GlaxoSmithKline, Medtronic, Pfizer, Sanofi-Aventis. Outros relacionamentos Financiamento de atividades de educação médica continuada, incluindo viagens, hospedagens e inscrições para congressos e cursos, provenientes da indústria farmacêutica, de órteses, próteses, equipamentos e implantes, brasileiras ou estrangeiras: - Pfizer, Daiichi Sankyo, Novo Nordisk, Novartis.
Ricardo Alves da Costa	Nada a ser declarado
Ricardo Ryoshim Kuniyoshi	Declaração financeira A - Pagamento de qualquer espécie e desde que economicamente apreciáveis, feitos a (i) você, (ii) ao seu cônjuge/ companheiro ou a qualquer outro membro que resida com você, (iii) a qualquer pessoa jurídica em que qualquer destes seja controlador, sócio, acionista ou participante, de forma direta ou indireta, recebimento por palestras, aulas, atuação como proctor de treinamentos, remunerações, honorários pagos por participações em conselhos consultivos, de investigadores, ou outros comitês, etc. Provenientes da indústria farmacêutica, de órteses, próteses, equipamentos e implantes, brasileiras ou estrangeiras: - Daiichi Sankyo: Lixiana; Boehringer Ingelheim: Pradaxa; Biotronik: arritmias.
Rui Manuel de Sousa Sequeira Antunes de Almeida	Declaração financeira A - Pagamento de qualquer espécie e desde que economicamente apreciáveis, feitos a (i) você, (ii) ao seu cônjuge/ companheiro ou a qualquer outro membro que resida com você, (iii) a qualquer pessoa jurídica em que qualquer destes seja controlador, sócio, acionista ou participante, de forma direta ou indireta, recebimento por palestras, aulas, atuação como proctor de treinamentos, remunerações, honorários pagos por participações em conselhos consultivos, de investigadores, ou outros comitês, etc. Provenientes da indústria farmacêutica, de órteses, próteses, equipamentos e implantes, brasileiras ou estrangeiras: - Meril Brasil: Dafodil. B - Financiamento de pesquisas sob sua responsabilidade direta/pessoal (direcionado ao departamento ou instituição) provenientes da indústria farmacêutica, de órteses, próteses, equipamentos e implantes, brasileiras ou estrangeiras: - Meril Brasil: Dafodil.
Silvia Helena Cardoso Boghossian	Declaração financeira A - Pagamento de qualquer espécie e desde que economicamente apreciáveis, feitos a (i) você, (ii) ao seu cônjuge/ companheiro ou a qualquer outro membro que resida com você, (iii) a qualquer pessoa jurídica em que qualquer destes seja controlador, sócio, acionista ou participante, de forma direta ou indireta, recebimento por palestras, aulas, atuação como proctor de treinamentos, remunerações, honorários pagos por participações em conselhos consultivos, de investigadores, ou outros comitês, etc. Provenientes da indústria farmacêutica, de órteses, próteses, equipamentos e implantes, brasileiras ou estrangeiras: - Medtronic; Boston Scientific.
Tan Chen Wu	Nada a ser declarado
Thais Aguiar do Nascimento	Nada a ser declarado
Thiago da Rocha Rodrigues	Declaração financeira A - Pagamento de qualquer espécie e desde que economicamente apreciáveis, feitos a (i) você, (ii) ao seu cônjuge/ companheiro ou a qualquer outro membro que resida com você, (iii) a qualquer pessoa jurídica em que qualquer destes seja controlador, sócio, acionista ou participante, de forma direta ou indireta, recebimento por palestras, aulas, atuação como proctor de treinamentos, remunerações, honorários pagos por participações em conselhos consultivos, de investigadores, ou outros comitês, etc. Provenientes da indústria farmacêutica, de órteses, próteses, equipamentos e implantes, brasileiras ou estrangeiras: - Libbs.
Veridiana Silva de Andrade	Outros relacionamentos Financiamento de atividades de educação médica continuada, incluindo viagens, hospedagens e inscrições para congressos e cursos, provenientes da indústria farmacêutica, de órteses, próteses, equipamentos e implantes, brasileiras ou estrangeiras: - Medtronic: Curso Micra.

## Sumário

**1. Introdução** 12
**2. Epidemiologia** 13
**3. Definição e Classificação** 14
  **3.1. Definição** 14
  **3.2. Classificação** 14
**4. Mecanismos Fisiopatológicos** 18
  **4.1. Progressão da Doença 19 4.2. Cardiomiopatia Atrial** 19
  **4.3. Fatores Predisponentes para Fibrilação Atrial** 19
    **4.3.1. Fatores Intrínsecos** 20
      ***4.3.1.1. Idade e Sexo*** 20
      ***4.3.1.2. Polimorfismos Genéticos*** 20
    **4.3.2. Comorbidades** 20
      ***4.3.2.1. Hipertensão Arterial Sistêmica*** 20
      ***4.3.2.2. Insuficiência Cardíaca*** 21
      ***4.3.2.3. Diabetes Melito*** 21
      ***4.3.2.4. Refluxo Gastroesofágico*** 21
      ***4.3.2.5. Infecções Sistêmicas*** 22
      ***4.3.2.6. Outras Condições*** 22
  **4.4. Hábitos de Vida** 22
    **4.4.1. Obesidade** 22
    **4.4.2. Sedentarismo** 23
    **4.4.3. Má Qualidade de Sono** 23
    **4.4.4. Uso de Álcool** 23
    **4.4.5. Tabagismo** 24
    **4.4.6. Consumo de Cafeína** 24
**5. Diagnóstico de Fibrilação Atrial** 24
  **5.1. Manifestações Clínicas** 24
  **5.2. Impacto na Qualidade de Vida e Hospitalização** 26
  **5.3. Avaliação Clínica do Paciente com Fibrilação Atrial** 26
  **5.4. Triagem no Paciente com Risco de Fibrilação Atrial** 26
  **5.5. Monitorização Eletrocardiográfica Prolongada** 26
**6. Fibrilação Atrial e Fenômenos Tromboembólicos** 28
  **6.1. Algoritmos para Avaliação do Risco de Fenômenos Tromboembólicos** 28
  **6.2. Algoritmos para avaliação de Risco de Sangramento** 28
  **6.3. Prevenção de Fenômenos Tromboembólicos** 30
    **6.3.1. Anticoagulantes Disponíveis na Prática Clínica** 30
    **6.3.2. Individualização do Paciente para Decisão da Melhor Estratégia de Anticoagulação** 30
  **6.4. Orientações aos Pacientes em Uso de Anticoagulação** 32
    **6.4.1. Orientações nos Casos de Sangramento com os ACOD** 33
  **6.5. Uso dos Anticoagulantes em Situações Especiais** 35
    **6.5.1. Cardioversão Elétrica** 35
    **6.5.2. Fibrilação Atrial Valvar** 37
      ***6.5.2.1. Anticoagulação Oral em Pacientes com Doenças Cardíacas Valvares em Válvulas Nativas*** 37
      ***6.5.2.2. Anticoagulação Oral em Pacientes com Estenose Mitral Moderada a Grave*** 37
      ***6.5.2.3. Anticoagulação Oral em Pacientes com Próteses*** 38
    **6.5.3. Anticoagulação na Síndrome Coronariana Aguda** 39
    **6.5.4. Manejo da Anticoagulação no Perioperatório de Ablação e Cirurgia Cardíaca** 42
    **6.5.5. Anticoagulação na Fibrilação Atrial Subclínica** 44
  **6.6. Oclusão do Apêndice Atrial Esquerdo e Anticoagulação** 45
    **6.6.1. Contraindicações ao Uso de Anticoagulantes** 45
    **6.6.2. Oclusão Percutânea do Apêndice Atrial Esquerdo** 45
    **6.6.3. Oclusão Cirúrgica do Apêndice Atrial Esquerdo** 47
    **6.6.4. Anticoagulação após Oclusão do Apêndice Atrial Esquerdo** 48
      ***6.6.4.1. Anticoagulação após Oclusão Percutânea do Átrio Esquerdo*** 48
      ***6.6.4.2. Terapia Antitrombótica Pós-oclusão Cirúrgica do Apêndice Atrial Esquerdo*** 49
  **6.7. Estratégias para Minimizar o Risco de Sangramento** 49
  **6.8. Escolha do Anticoagulante Oral** 49
**7. Tratamento do Ritmo ou da Frequência Cardíaca na Fibrilação Atrial** 50
  **7.1. Fármacos Utilizados no Controle do Ritmo** 50
    **7.1.1. Propafenona** 51
    **7.1.2. Amiodarona** 51
    **7.1.3. Sotalol** 51
  **7.2. Fármacos Utilizados no Controle da Frequência Cardíaca** 52
    **7.2.1. Betabloqueadores** 52
    **7.2.2. Bloqueadores do Cálcio** 52
    **7.2.3. Digoxina** 52
  **7.3. Escolha entre Controle do Ritmo ou Controle da Frequência Cardíaca ** 52
  **7.4. Controle da Frequência** 53
    **7.4.1. Meta Terapêutica** 54
    **7.4.2. Controle Agudo** 54
    **7.4.3. Controle em Longo Prazo** 54
    **7.4.4. Ablação do Nó Atrioventricular** 55
  **7.5. Controle do Ritmo – Situações Especiais** 55
    **7.5.1. Cardioversão Elétrica** 55
    **7.5.2. Cardioversão Química** 55
      ***7.5.2.1. Uso do Esquema "Pill-in-the-pocket"*** 55
  **7.6. Tratamento Farmacológico para Manutenção do Ritmo Sinusal** 57
**8. Ablação de Fibrilação Atrial** 59
  **8.1. Tecnologias de Mapeamento e Ablação** 59
    **8.1.1. Sistemas de Mapeamento Tridimensional** 59
    **8.1.2. Eco Intracardíaco na Ablação de Fibrilação Atrial** 59
    **8.1.3. Tecnologias de Ablação** 60
  **8.2. Indicações para a Realização de Ablação de Fibrilação Atrial** 61
    **8.2.1. Estudos Clínicos em Ablação de Fibrilação Atrial** 61
  **8.3. Complicações** 63
  **8.4. Principais Complicações** 64
    **8.4.1. Lesão Térmica Esofágica** 64
    **8.4.2. Fístula Atrioesofágica ou Esôfago-pericárdica** 66
    **8.4.3. Alterações da Motilidade Gastroesofágica** 66
    **8.4.4. Tamponamento Cardíaco** 66
    **8.4.5. Acidente Tromboembólico Sistêmico** 67
    **8.4.6. Embolia Aérea** 67
    **8.4.7. Estenose de Veias Pulmonares** 67
    **8.4.8. Paralisia Frênica** 67
    **8.4.9. Complicação Vascular** 68
    **8.4.10. Outras Complicações** 68
  **8.5. Crioablação** 68
    **8.5.1. Técnica de Crioablação de Fibrilação Atrial** 68
  **8.6. Ablação por Cateter para Fibrilação Atrial em Pacientes com Insuficiência Cardíaca** 69
**9. Tratamento Cirúrgico da Fibrilação Atrial** 70
  **9.1. Indicações para o Tratamento Cirúrgico da Fibrilação Atrial ** 71
    **9.1.1. Tratamento Cirúrgico da Fibrilação Atrial Concomitante a Outra Cirurgia Cardíaca** 71
    **9.1.2. Tratamento Cirúrgico Isolado da Fibrilação Atrial ** 72
  **9.2. Técnicas Cirúrgicas** 72
  **9.3. Resultados a Longo Prazo e Complicações** 73
  **9.4. Procedimentos Híbridos** 73
**10. Abordagem Multidisciplinar da Fibrilação Atrial ** 73
  **10.1. Implementação de Programa de Mudança de Qualidade de Vida** 73
**11. Fibrilação Atrial em Situações Especiais** 73
  **11.1. Hemorragia Intracraniana** 73
  **11.2. População Muito Idosa** 74
  **11.3. Grupos Étnicos Minoritários** 74
  **11.4. Insuficiência Renal** 75
  **11.5. Fibrilação Atrial na Gestação** 76
  **11.6. Fibrilação Atrial, Sistema Nervoso Parassimpático e Cardioneuroablação** 76
  **11.7. Fibrilação Atrial no Atleta** 77
  **11.8. Síndromes Arrítmicas** 78
    **11.8.1. Síndrome Arrítmica em Jovens sem Aparente Cardiopatia Estrutural** 78
  **11.9. Fibrilação e Flutter Atrial em Cardiopatia Congênita do Adulto** 78
  **11.10. Peculiaridades da Fibrilação Atrial em Mulheres** 79
  **11.11. Fibrilação Atrial no Portador de Dispositivo Cardíaco Eletrônico Implantável** 81
    **11.11.1. Detecção** 81
    **11.11.2. Ajustes de Programação** 81
    **11.11.3. Cardioversão e Ablação** 81
  **11.12. Fibrilação Atrial no Pós-operatório de Cirurgia Cardíaca e Não Cardíaca** 82
    **11.12.1. Fatores Preditores** 82
    **11.12.2. Comorbidades e Dados Epidemiológicos** 82
      ***11.12.2.1. Preditores Cirúrgicos*** 82
    **11.12.3. Prevenção Pré-operatória** 83
    **11.12.4. Tratamento da Fibrilação Atrial no Pós-operatório** 83
**12. Fibrilação Atrial e Alterações Cognitivas** 83
**13. Importância de Ações Educacionais no Manejo do Paciente com Fibrilação Atrial** 84
**14. Análise de Custo-benefício e Uso Adequado de Recursos em Fibrilação Atrial** 84

## 1. Introdução

A fibrilação atrial (FA) é a arritmia sustentada mais comum na prática clínica. Com o envelhecimento populacional, as estimativas sobre prevalência e incidência dessa arritmia são alarmantes. Estima-se que o número de pacientes portadores de FA com idade superior a 55 anos será mais que o dobro em 2060.^1^ Isso poderá ocasionar grande impacto no sistema de saúde brasileiro e mundial, uma vez que a principal consequência da FA, o acidente vascular cerebral (AVC), é uma condição bastante debilitante. A FA aumenta em média quatro vezes a chance de AVC e está associada a maior risco de mortalidade por todas as causas e insuficiência cardíaca (IC).^2^

**Figure:**
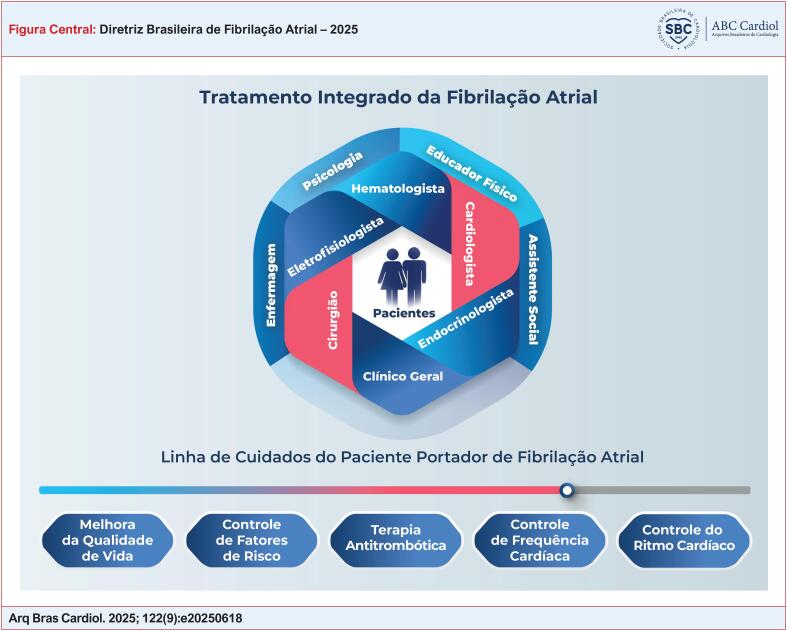
Representação dos pilares e da linha de cuidado no tratamento integrado do paciente com fibrilação atrial.

Nesse cenário, inúmeros grupos de pesquisa se dedicam para maior compreensão dos mecanismos fisiopatológicos e tratamento da FA, assim como para o desenvolvimento tecnológico necessário para o aprimoramento de sistemas de mapeamento e ablação. Paralelamente, houve um robusto progresso nas ferramentas diagnósticas da FA e nos sistemas de monitorização eletrocardiográfica prolongada. O uso dos novos anticoagulantes assumiu um papel de destaque na prevenção de fenômenos tromboembólicos, e o fechamento do apêndice atrial esquerdo (AAE) está cada vez mais acessível em centros de atendimento de pacientes com FA.

Com isso, tornou-se emergente a necessidade de uma nova diretriz que possa nortear o manejo clínico da FA, modificado significativamente nos últimos anos. Entre as principais mudanças, a multidisciplinaridade merece destaque: não apenas cardiologistas, mas também eletrofisiologistas, nefrologistas, hematologistas, enfermeiros, fisioterapeutas e cuidadores participam do manejo clínico. Todavia, o próprio paciente deve estar no centro do processo de tomada de decisão e respaldado por um time de especialistas de forma a individualizar a abordagem de acordo com as peculiaridades de cada um. Paralelamente, o tratamento assume frentes distintas incluindo cinco pilares de atuação para o melhor tratamento desses pacientes (Figura Central).

## 2. Epidemiologia

A FA é a arritmia recorrente mais comum na prática clínica, causadora de substancial morbimortalidade nos pacientes acometidos. Está associada de forma independente com piora da qualidade de vida (QV) e aumento do risco de AVC, IC e mortalidade total.^3,4^

**Figura 1:**
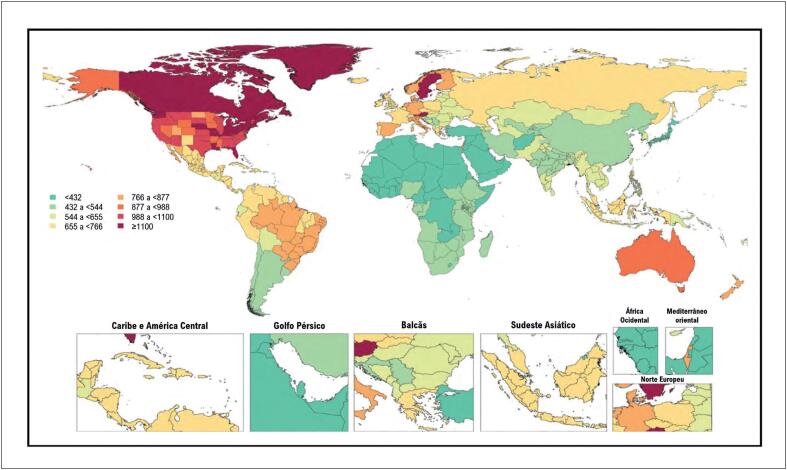
Prevalência global de fibrilação atrial (FA)/flutter atrial (FLA) padronizada por idade por 100.000, em ambos os sexos em 2020. Adaptado de Tsao et al.^6^

A incidência e prevalência da FA estão aumentando de forma global. Segundo dados do Estudo de Framingham, a prevalência de FA aumentou três vezes nos últimos 50 anos.^5^ Estima-se que a prevalência de FA nos Estados Unidos irá aumentar de 5,2 milhões em 2010 para 12,1 milhões em 2030.^6,7^ A prevalência padronizada para FA é maior em países de alta renda como na América do Norte e Australásia^6^ (Figura 1). Na Europa, a prevalência de FA em adultos acima de 55 anos era de 8,8 milhões em 2010 e a projeção é de 17,9 milhões em 2060.^8^

**Figura 2:**
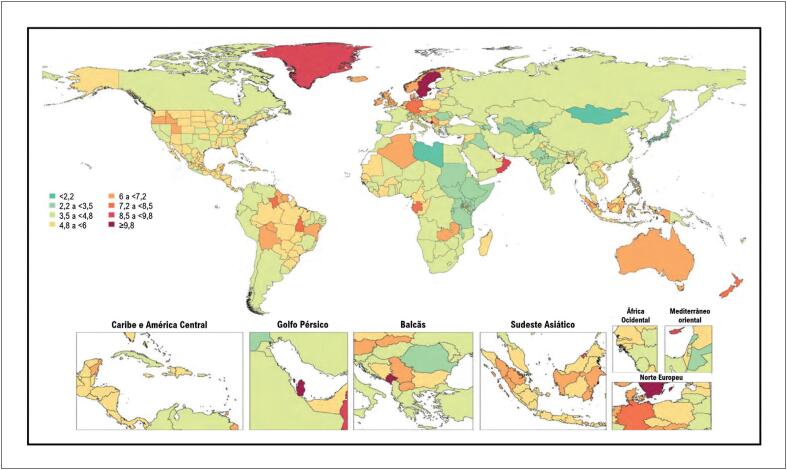
Mortalidade global de FA/FLA padronizada por idade, em ambos os sexos, em 2020. Adaptado de Tsao et al.^6^

Esses números expressivos também são acompanhados de grande impacto na saúde populacional. O número total de mortes atribuídas a FA e *flutter* atrial (FLA) em 2020 foi de 0,33 milhões, sendo 0,13 milhões no sexo masculino e 0,20 entre as mulheres. A mortalidade padronizada para idade é maior na Europa Ocidental e Australásia^6^ (Figura 2).

A prevalência padronizada por idade de FA/FLA no Brasil aumentou de 519/100.000 habitantes em 1990 para 537/100.000 habitantes em 2019, em ambos os sexos, com variação de 3,5%.^9^ Em análise do Estudo Longitudinal da Saúde do Adulto (ELSA - Brasil), foram avaliados a frequência de FA e FLA por eletrocardiograma (ECG) e o autorrelato, assim como o uso de medicamentos de prevenção de AVC em 13.260 pacientes. Foi demonstrado que 2,5% dos pacientes tinham FA/FLA e que o baixo uso de anticoagulantes era comum, especialmente nas mulheres.^10^ Outra análise, com 767 participantes do estudo EPISONO, com idade média de 42 anos, submetidos a polissonografia e análise do traçado eletrocardiográfico durante o registro do sono, demonstrou que a ocorrência de FA/FLA variou de 0,2 a 1,65%, sendo observadas porcentagens mais elevadas em pacientes portadores de apneia do sono grave.^11^ Por outro lado, em uma análise com 1.524 idosos moradores da cidade de São Paulo, a prevalência de FA/FLA chegou a 2,9% em homens e 2,0% em mulheres nessa população.^12^

**Figura 3:**
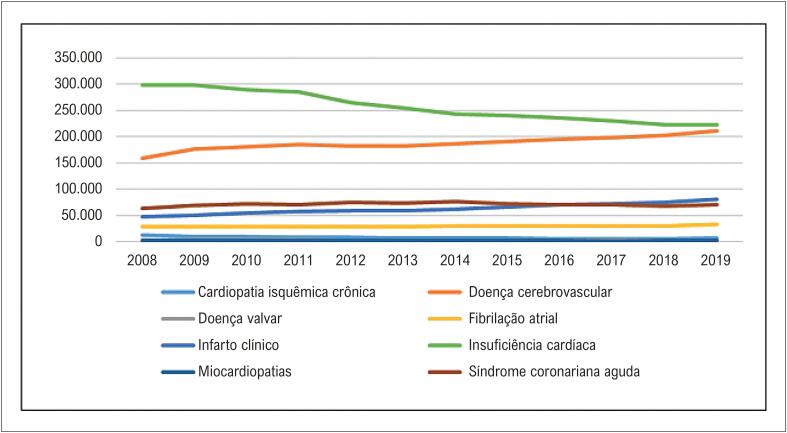
Total de hospitalizações para procedimentos clínicos para doenças cardiovasculares por ano de competência, Brasil, 2008 a 2019. Fonte: Sistema de Informações sobre Mortalidade – SIM/DATASUS. Adaptado de Oliveira et al.^9^

Os dados epidemiológicos robustos fazem da FA um grande problema de saúde pública, especialmente no cenário brasileiro, que está vivenciando um rápido e significativo envelhecimento populacional, com gastos públicos crescentes e grande consumo de recursos em saúde, especialmente com hospitalizações potencialmente evitáveis (Figura 3).

## 3. Definição e Classificação

### 3.1. Definição

**Figura 4:**
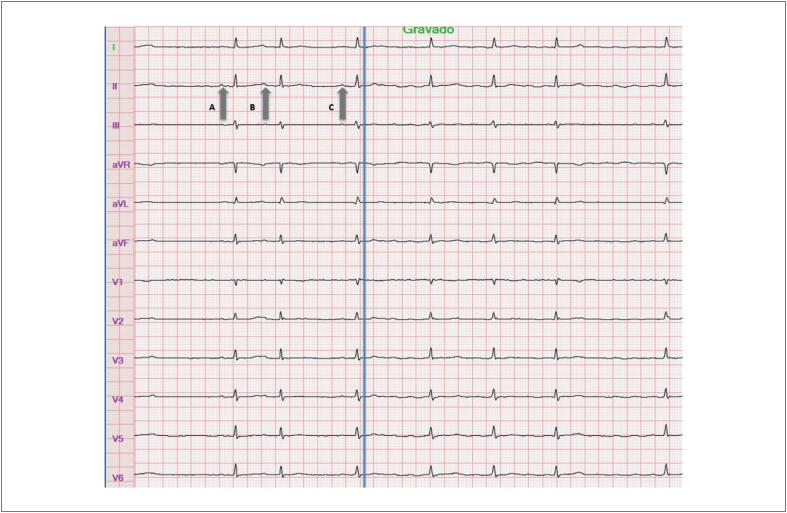
Traçado de Holter de 12 derivações mostrando a mudança de ritmo. Batimento A e C precedidos de onda p e batimento B compatível com ectopia atrial isolada. A linha azul sinaliza o momento da mudança de ritmo com ausência de onda p associado à irregularidade em RR. Figura cedida pela Dra. Fatima Dumas Cintra.

A FA é definida como uma arritmia atrial com completa desorganização da atividade elétrica atrial e consequente ineficácia da sua contração. No ECG, a FA caracteriza-se por apresentar intervalos RR irregulares (desde que a condução atrioventricular [AV] esteja preservada), ausência de ondas P bem definidas e sequenciais que são substituídas por ondas fibrilatórias (onda f) na linha de base do ECG, secundário à desorganização elétrica dos átrios (Figura 4).

### 3.2. Classificação

Uma vez definida a FA, classificá-la se torna de extrema importância, pois permite que se estabeleçam parâmetros diagnósticos e que se obtenham informações sobre a carga de arritmia e a inferência do grau de acometimento da doença. Além disso, essas informações serão fundamentais para a escolha terapêutica mais adequada e definição de critérios homogêneos para os próximos estudos clínicos.

Inicialmente, a FA deve ser classificada em clínica ou subclínica (Tabela 1 e Tabela 2). Com o avanço da tecnologia e maior conscientização sobre a importância do diagnóstico precoce da FA, a busca ativa por esses pacientes foi ampliada e hoje conta com dispositivos vestíveis inteligentes ou implantáveis de grande valia na detecção precoce da arritmia.^13-15^ Entretanto, é de suma importância definir critérios claros para o seu correto diagnóstico. Para isso, se faz necessário o registro eletrocardiográfico da arritmia, convencionando-se que o episódio de FA deva durar, ao menos, 30 segundos para ser considerada uma arritmia clínica.^16^ Dessa forma, independentemente da presença ou não de sintomas, a FA é diagnosticada como clínica se um médico validar a sua presença por um traçado de ECG convencional de 12 derivações ou um traçado de ritmo de ECG de derivação única com duração maior ou igual a 30 segundos.^17^ As recomendações para o diagnóstico de FA estão apresentadas no Quadro 1.

**Tabela 1:** Classificação da fibrilação atrial (FA): de acordo com a apresentação

FA clínica	FA sintomática ou assintomática, registrada em ECG de 12 derivações ou em ECG de ritmo com derivação única ≥ 30 segundos*.
FA subclínica	Refere-se a indivíduos assintomáticos com observação de ritmo atrial acelerado em DCEI#.

* Os registros eletrocardiográficos devem ser avaliados e atestados por médico capacitado.

# Com o objetivo de reduzir a presença de artefatos, normalmente considera-se como ritmo atrial acelerado os episódios com frequência ≥ 175 bpm, avaliados e atestados por um especialista. ECG: eletrocardiograma; DCEI: dispositivo cardíaco eletrônico implantável.

**Tabela 2:** Classificação da fibrilação atrial (FA): de acordo com a duração

FA paroxística	FA com menos de 7 dias de duração (independentemente da forma de reversão).
FA persistente	FA com mais de 7 dias e menos de 1 ano de duração.
FA persistente de longa duração	FA com mais de 1 ano de duração.
FA permanente	FA na qual o médico e o paciente chegaram à decisão de não tentar o reestabelecimento do ritmo sinusal, independentemente do seu tempo de instalação.
FA pós-ablação	Paciente submetido à ablação por cateter ou cirúrgica da FA que evolui sem recorrência da arritmia.

**Quadro 1:** Recomendação para o diagnóstico de fibrilação atrial (FA)

	Classe de recomendação	Nível evidência
A documentação eletrocardiográfica é necessária para o diagnóstico de FA	**I**	**C**
O diagnóstico de FA clínica deve ser realizado pelo eletrocardiograma de 12 derivações ou traçado de derivação única com ausência de onda P e irregularidade RR por, no mínimo, 30 segundos de duração	**I**	**C**

Já a FA subclínica ocorre em pacientes sem sintomas atribuídos à FA com registro de ritmo atrial acelerado através do eletrodo atrial de dispositivos cardíacos eletrônicos implantáveis (DCEI) ou registro sugestivo de FA em monitores de eventos implantáveis ou monitores vestíveis.^17^ Devido às grandes variações nos cortes de frequência cardíaca (FC) atrial (entre 160 e 200 bpm) e à duração dos episódios (10 segundos a 24 horas) utilizados como critério de inclusão nos principais estudos clínicos, existe grande dificuldade em se estabelecer parâmetros precisos de FC e duração dos episódios para que se considere um ritmo atrial acelerado. De forma geral, a frequência programada nos DCEI para detecção de um ritmo atrial acelerado é de ≥ 175 bpm, e o tempo de duração considerado é normalmente ≥ 5 minutos, para que se evitem registros de artefatos.

**Figura 5:**
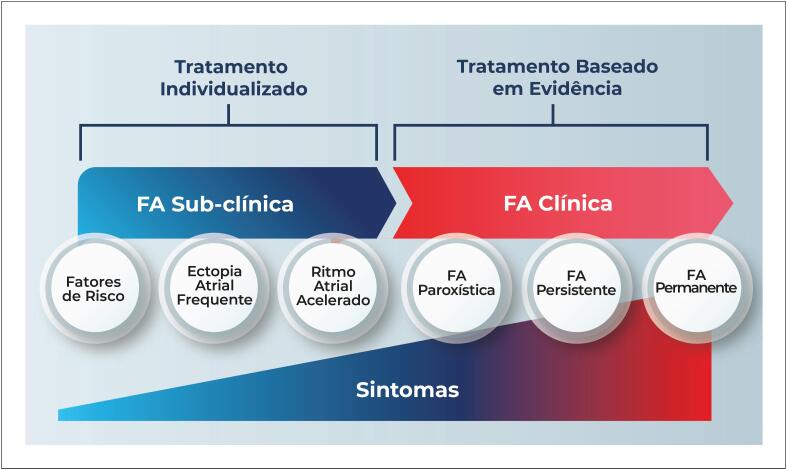
Intersecção entre fibrilação atrial (FA) clínica e FA subclínica para tomada de decisão do tratamento.

De fato, o avanço tecnológico nos meios de detecção de FA e episódios de ritmo atrial acelerado, através dos DCEI, monitores de eventos implantáveis ou dispositivos vestíveis, tem aumentado substancialmente o diagnóstico tanto de FA clínica quanto o de FA subclínica. Estudos de validação clínica com ECG demonstram que os dispositivos vestíveis apresentam sensibilidade de 67,7 a 100% e especificidade de 60,7 a 100% para o diagnóstico de FA.^18^ O fato de esses dispositivos, especialmente aqueles associados a celulares, estarem acessíveis a um número cada vez maior de pessoas representa uma oportunidade e um desafio para a implementação de estratégias de rastreamento da FA. Entre os principais desafios, estão a necessidade de definir qual grupo deve ser monitorado, qual o melhor dispositivo a ser utilizado, como acessar os serviços de saúde e qual conduta deve ser tomada de acordo com a informação gerada pelo dispositivo.^19,20^ Outra questão é que esses pacientes não foram contemplados nos grandes estudos clínicos que regem o tratamento atual da FA. Dessa forma, até que estudos mostrem as melhores opções terapêuticas para FA subclínica, esses casos devem ser conduzidos com cautela e analisados individualmente em seu contexto clínico, para que se adote a conduta mais apropriada^21^ (ver Item 6.5.4). A Figura 5 demonstra as características da FA clínica e subclínica.

Outra classificação importante da FA é quanto a sua duração (Tabela 1 e Tabela 2) que permitirá que se avalie, associado a outros fatores clínicos, o tempo de doença e quais as chances de reestabelecer e/ou manter o paciente em ritmo sinusal (RS).^17,22^ Dessa forma, a FA pode ser classificada em: FA paroxística, quando a FA durar menos que 7 dias, independentemente de como foi revertida (reversão espontânea, química ou elétrica); FA persistente, quando a FA durar mais que 7 dias e menos de 1 ano (mesmo com reversão espontânea, química ou elétrica nesse período); FA persistente de longa duração, em que a FA apresenta mais de 1 ano de duração, mas em que se optou adotar a estratégia terapêutica de controle do ritmo (reversão e manutenção do RS); e FA permanente, que são pacientes com FA instalada, independentemente do tempo, em que médico e paciente chegaram à decisão de não se tentar o reestabelecimento do RS. Nesses casos, se em algum momento de sua evolução porventura se reconsiderar o tratamento de controle do ritmo, essa FA deixa de ser classificada como permanente e passa a ser classificada como FA persistente de longa duração. Alguns pacientes apresentaram episódios de FA paroxística e episódios de FA persistente. Nesses casos, aconselha-se que se classifique o paciente conforme a forma mais prevalente.

Ainda em relação à classificação de FA, alguns termos amplamente utilizados na prática clínica no passado devem ser abandonados por não acrescentarem informações corretas e/ou relevantes ao seguimento do paciente. Um exemplo é o termo FA isolada ou solitária, que não é mais utilizado pelo fato de se admitir que, mesmo na ausência de patologias, toda FA tem uma causa presente, mesmo que seja um distúrbio elétrico intrínseco.^23^ Outro termo que está em desuso é FA valvar e não valvar.^9^ A recomendação hoje é que se especifique o problema de cada paciente e, ao se identificar, por exemplo, pacientes com estenose mitral moderada a grave ou com próteses valvares mecânicas, as condutas sejam tomadas de acordo com as recomendações para a condição clínica específica. Por fim, o termo FA crônica, que é inespecífico, não acrescenta nada à conduta clínica e, dessa forma, não deve ser utilizado.

**Figura 6:**
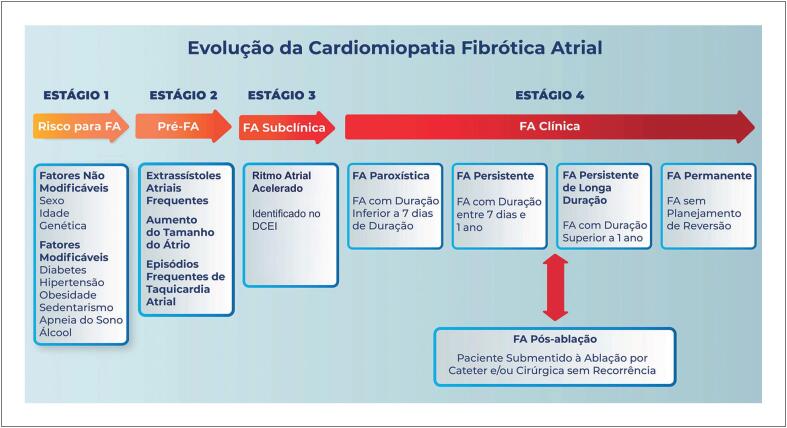
Estágios evolutivos da fibrilação atrial (FA). Adaptado de Joglar et al.^24^

A diretriz americana apresenta uma nova forma de classificação de acordo com a evolução da doença. Essa iniciativa é importante porque inclui pacientes submetidos à ablação com sucesso e sem recorrência clínica, um grupo de pacientes cada vez mais frequente na prática clínica e que não estava representado. Apesar de esses pacientes não apresentarem mais FA, ainda estão sob risco de apresentar novos eventos, sendo necessárias medidas terapêuticas distintas, como modificação de estilo de vida, monitorização de episódios sintomáticos e assintomáticos de FA e terapia anticoagulante adequada. A Figura 6 apresenta os estágios evolutivos da FA conforme a diretriz americana.^24^

## 4. Mecanismos Fisiopatológicos

**Figura 7:**
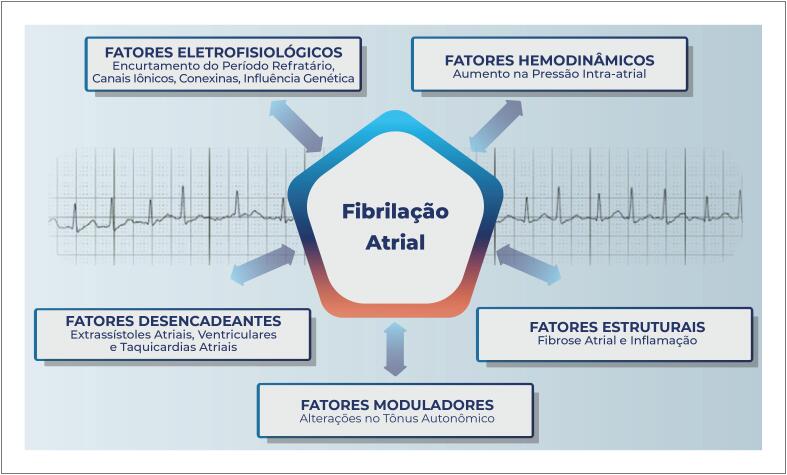
Fisiopatologia da fibrilação atrial. Adaptado de Cintra et al.^25^

A fisiopatologia do início e da manutenção da FA envolve uma complexa interrelação entre fatores deflagradores (gatilhos), fatores moduladores, alterações eletrofisiológicas, hemodinâmicas e estruturais dos átrios (substrato), demonstrada na Figura 7.

A principal hipótese para o início e a manutenção da FA inclui a presença de focos ectópicos como deflagradores da arritmia e a reentrada como fator de manutenção.^26^ A maioria desses focos ectópicos tem origem nas veias pulmonares, mas também são descritos focos originários da veia cava superior, ligamento de Marshall, seio coronário, parede do átrio esquerdo (AE) e crista terminalis.

As veias pulmonares têm papel fundamental na gênese e manutenção da FA. As veias pulmonares apresentam prolongamentos de cardiomiócitos que se estendem desde o AE, com propriedades eletrofisiológicas e anatômicas que facilitam a ocorrência da arritmia. A base molecular para os focos ectópicos é atribuída principalmente a anormalidades no fluxo do cálcio.^27^ Os prolongamentos de cardiomiócitos apresentam fibras dispostas em diferentes orientações, com pouco acoplamento lateral, favorecendo a ocorrência de áreas de fibrose e desarranjo local, que aumentam a suscetibilidade a focos ectópicos e favorecem fenômenos de reentrada.^26^

**Figura 8:**
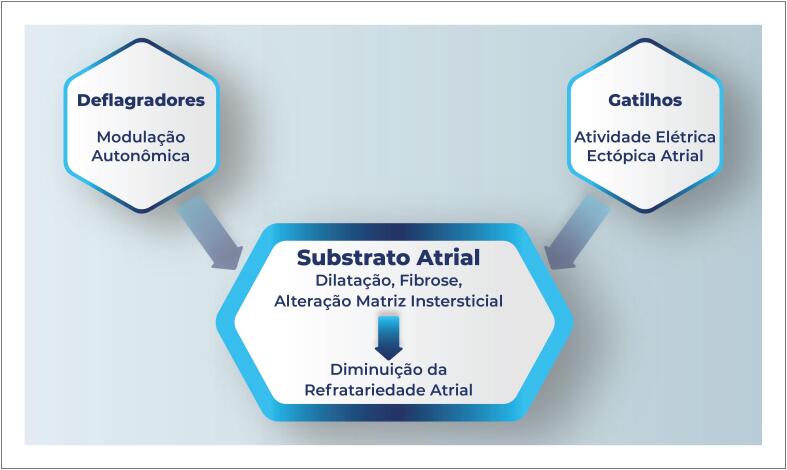
Modelo conceitual do mecanismo arritmogênico da fibrilação atrial baseado em três pilares fisiopatológicos: substrato atrial, gatilhos e deflagradores.

A ocorrência de circuitos de reentrada em um substrato atrial propício é o principal mecanismo de manutenção da FA. Alterações estruturais caracterizadas por dilatação atrial, alterações na matriz intersticial e fibrose alteram a condução normal e diminuem a refratariedade atrial, possibilitando, assim, a ocorrência de múltiplas ondas simultâneas de reentrada, facilitando a manutenção da FA. Além disso, a atividade autonômica também tem papel importante na fisiopatologia da FA. A ativação simpática e vagal combinada tem potencial de tornar o substrato atrial ainda mais propenso ao início e à perpetuação da arritmia^28,29^ (Figura 8).

### 4.1. Progressão da Doença

A FA tem uma história natural de progressão (Figura 6).^27^ Dados de registro mostram que a progressão de FA paroxística para formas persistentes é de 8,6% em 1 ano e de 24,7% em 5 anos.^28^ O remodelamento elétrico e a continuidade da exposição a fatores predisponentes determinam a natureza progressiva do substrato arritmogênico.

### 4.2. Cardiomiopatia Atrial

A cardiomiopatia atrial foi definida alterações estruturais, da arquitetura, contráteis ou eletrofisiológicas afetando os átrios com o potencial de produzir manifestações clínicas relevantes.^30^ O conceito da cardiomiopatia atrial busca o melhor entendimento da relação entre o substrato do miocárdio atrial e seu papel na arritmogênese e ocorrência de fenômenos tromboembólicos. A definição da cardiomiopatia atrial é histopatológica, porém alterações indicativas desta condição podem ser reconhecidas por meio de alterações em métodos não invasivos, como ECG (bloqueio interatrial, prolongamento da onda P, força terminal da onda P em V1), ecocardiograma (aumento do volume atrial), ressonância (volume atrial, realce tardio atrial) ou invasivos, como o registro de áreas de baixa voltagem durante o mapeamento eletroanatômico.^31^

A partir dessa perspectiva, a FA pode ser entendida como uma manifestação clínica de um miocárdio alterado, e não como um evento arrítmico independente. A cardiomiopatia atrial está associada a aumento do risco de eventos tromboembólicos (TE), independentemente do registro clínico de FA.^32,33^ Além disso, sinais de cardiomiopatia atrial mais avançada estão associados a menor taxa de sucesso na ablação de FA.^31^ Dessa forma, o reconhecimento da cardiomiopatia atrial é cada vez mais fundamental para a tomada de decisão clínica com relação ao manejo da FA e prevenção de eventos tromboembólicos.

### 4.3. Fatores Predisponentes para Fibrilação Atrial

Além de fatores intrínsecos como idade, sexo e influência genética, existem comorbidades e hábitos de vida que podem contribuir para o desenvolvimento da FA. Tais fatores são a base para a formação do substrato arritmogênico atrial para origem e manutenção dessa taquiarritmia. As evidências mais recentes apontam que o tratamento desses fatores de risco, além da mudança do estilo de vida, são partes fundamentais da prevenção e do tratamento da FA.^27,34-36^

As recomendações para o controle dos fatores de risco na FA estão apresentadas no Quadro 2.

**Quadro 2:** Controle dos fatores de risco para fibrilação atrial (FA)

Recomendação para o controle dos fatores de risco na FA	Classe	Nível de evidência
O tratamento da HAS com controle rigoroso dos níveis pressóricos está indicado para reduzir a recorrência de FA, o risco de acidente vascular cerebral e a progressão da doença	**I**	**A**
Todo paciente com FA e IC deve receber tratamento otimizado para IC	**I**	**A**
O tratamento do diabetes e o controle adequado da glicemia são recomendados para reduzir a recorrência de FA e a progressão da doença	**I**	**C**
Em pacientes com FA e doença do refluxo gastroesofágico, o tratamento adequado do refluxo pode reduzir o risco de FA	**I**	B
Uma redução de pelo menos 10% do peso é recomendada em pacientes com sobrepeso ou obesidade com FA	**I**	B
Um programa de atividade física regular é indicado para a prevenção de FA em pacientes quando não houver contraindicações	**I**	B
A redução do consumo de álcool (inferior a 30 gramas/semana) é recomendada para reduzir a recorrência de FA	**I**	B
A investigação clínica de distúrbio respiratório do sono é recomendada em pacientes com FA; entretanto, o benefício do tratamento com CPAP é incerto	IIa	B
Exercício de alto desempenho não deve ser indicado para a prevenção de FA	III	B

3 HAS: hipertensão arterial sistêmica; IC: insuficiência cardíaca; CPAP: pressão positiva contínua nas vias aéreas.

#### 4.3.1. Fatores Intrínsecos

##### 4.3.1.1. Idade e Sexo

A incidência da FA aumenta com a idade, dobrando a cada década.^27,37^ Alguns autores, entretanto, questionam essa relação, afirmando que, devido à maior longevidade populacional observada na atualidade, seria mais provável a detecção dessa arritmia nos indivíduos mais idosos. Além disso, a maior procura por atendimento médico devido à preocupação com a saúde e a popularização dos métodos de detecção de arritmias de caráter intermitente seriam outros fatores a serem considerados.^5^ A maioria dos estudos epidemiológicos sobre a FA baseia-se em informações obtidas de dados hospitalares ou bancos de dados eletrônicos de serviços de seguro médico, sujeitas a vieses, levando a uma ampla variação nas estimativas de incidência e prevalência dessa arritmia. Falta um estudo populacional com seguimento por tempo prolongado para confirmar a relação entre idade avançada e FA.

Conceitos epidemiológicos a parte, há dados clínicos que sugerem a associação do envelhecimento e FA. A influência da idade como determinante de risco pode estar relacionada ao maior tempo de exposição aos fatores de risco cardiovascular, que, de alguma maneira, comprometem a atividade elétrica atrial. O sexo masculino é 1,5 vezes mais propenso a apresentar FA. Depois dos 60 anos, entretanto, homens e mulheres apresentam a mesma incidência.^35^

##### 4.2.1.2. Polimorfismos Genéticos

As junções comunicantes, ou junções *gap*, formadas por proteínas transmembrana estruturais denominadas conexinas constituem vias intercelulares responsáveis pela rápida transmissão do potencial de ação e acoplamento elétrico do coração. O acoplamento elétrico anormal ocasionado pela alteração na distribuição de conexinas foi associado a maior suscetibilidade a FA. Mutações ou polimorfismos em quatro isoformas de conexinas já foram identificados como causadores de FA.^38^ Fato é que mais de 160 mutações já foram implicadas na gênese da FA nas últimas décadas. Não apenas mutações em proteínas estruturais, mas também mutações nos canais iônicos ou fatores de transcrição.^39^

A lipoproteína A, um fator de risco reconhecido para a doença aterosclerótica cardiovascular independente da dieta, também foi recentemente implicada como um possível mediador de FA, sugerindo uma ação deletéria mais ampla dessa lipoproteína e corroborando os achados de vulnerabilidade genética para o desenvolvimento da FA.^40^

#### 4.3.2. Comorbidades

##### 4.3.2.1. Hipertensão Arterial Sistêmica

A hipertensão arterial sistêmica (HAS) é talvez o fator de risco populacional mais importante para FA devido à sua elevada prevalência na população geral. O risco relativo (RR) para o surgimento de FA em hipertensos varia entre 20 e 50%. Um estudo indicou que a HAS isoladamente é responsável por 14% de todas as causas de FA.^41^ A pressão diferencial (pressão de pulso) e os valores pressóricos sistólicos levemente aumentados (entre 130 e 139 mmHg de pressão sistólica) estão mais associados a FA do que pressão arterial sistólica (PAS) abaixo de 120 mmHg.^42^ O risco de arritmia se eleva em 26% para cada aumento de 20 mmHg da pressão sistólica. Isso tem grande importância clínica, indicando que, para o seu tratamento, é fundamental o rigoroso controle da pressão arterial. Além disso, deve-se destacar que as recorrências da FA aumentam quando a pressão arterial se eleva pelo não tratamento adequado.

Estudos clínicos indicam que o tratamento efetivo da pressão arterial associado à redução da hipertrofia ventricular esquerda avaliada pelo critério de Cornell do ECG reduz a incidência de FA.^43-45^ Dados do ensaio clínico SPRINT (*Systolic Blood Pressure Intervention Trial*) apontam que o tratamento intensivo da HAS (PAS-alvo < 120mmHg) foi associado à redução de 26% do risco de FA em relação ao tratamento padrão (PAS-alvo < 140 mmHg).^46^ No Japão, o declínio das taxas de HAS associou-se à redução significativa da incidência de FA, particularmente em idade mais avançada.^47^ Esses achados indicam que o controle da HAS e a consequente redução de lesão em órgão-alvo, como a hipertrofia ventricular esquerda, reduzem o risco de FA. Além disso, o ECG pode ser uma ferramenta útil, pela análise dos critérios de Sokolow e Cornell de hipertrofia ventricular esquerda, para se estabelecer melhora clínica.

##### 4.3.2.2. Insuficiência Cardíaca

A IC aumenta em 4 a 6 vezes o risco de surgimento de FA.^27,35^ A maior incidência relaciona-se com a piora do grau funcional e com a sintomatologia apresentada pelo paciente, estando presente em 5 a 10% dos indivíduos com classe funcional I da New York Heart Association (NYHA), em 10 a 26% entre aqueles nas classes II e III e em 40 a 50% dos pacientes na classe funcional IV.^41,48^ Esses dados indicam que pacientes mais descompensados, com piores graus de disfunção ventricular sistólica, átrios aumentados e maior grau de efeito simpático são os mais afetados.^49^

A disfunção diastólica isolada também está associada ao aumento da incidência de FA, possivelmente refletindo fatores de risco compartilhados como idade avançada e HAS.^50^ Nessa situação, o aumento da pressão ventricular final reflete-se nos átrios, causando sua distensão e dilatação.^51^

A FA na IC ocorre secundariamente a diversos fatores, incluindo desde aumento da frequência de ectopias atriais, dilatação atrial, hipervolemia e aumento do tônus autonômico (hiperatividade simpática). O fato de ocorrer em pacientes mais descompensados e, portanto, com maior efeito de atividade adrenérgica (fator instabilizador do substrato) fornece suporte a esse raciocínio. Por essa razão, a compensação clínica do paciente pode melhorar as condições hemodinâmicas e restabelecer o ritmo cardíaco normal. Conhecendo-se o fato de que o surgimento da FA ocorre com a piora clínica da IC, sua prevenção deve ser feita tratando agressivamente o quadro congestivo. Nesse caso, a prevenção primária da arritmia passa a ter um papel preponderante.

##### 4.3.2.3. Diabetes Melito

Em um estudo realizado nos Estados Unidos, foi demonstrado que o diabetes é um fator de risco independente para o desenvolvimento de FA, com risco duas vezes maior em indivíduos diabéticos mais jovens.^52^ Além disso, a presença de diabetes dobra o risco de FA independentemente da presença de outras comorbidades.^53^ Os efeitos adversos do diabetes em vários sistemas também podem contribuir para um risco maior de FA, cuja ocorrência chega a 40% dos pacientes com níveis glicêmicos elevados. Não somente sua maior incidência, mas também o maior tempo de duração do diabetes associa-se com maior risco de FA, conforme demonstrado pela presença de níveis elevados de hemoglobina glicada.^54^ Nesse caso, pode-se concluir que, além da presença da doença, níveis glicêmicos elevados por muito tempo devem ser os principais fatores de risco para o surgimento de FA nessa endocrinopatia. O diabetes frequentemente se associa a outros fatores de risco para FA, como obesidade, apneia do sono e HAS, o que agrava sua repercussão sobre os pacientes afetados.

O surgimento de disfunção autonômica no diabetes melito, conhecida como neuropatia autonômica cardíaca, aumenta o risco de arritmias atriais. Ela se caracteriza por aumento da atividade simpática e redução da atividade parassimpática. A morbidade relacionada com essa neuropatia varia entre 34 e 44% dos pacientes com diabetes tipo II, conforme demonstrado por meio da análise da variabilidade do intervalo RR.^54^

A manutenção de uma glicemia normal associada a baixos níveis de hemoglobina glicada pode diminuir o risco de surgimento de FA, particularmente por reduzir o risco de neuropatia autonômica cardíaca e o processo inflamatório sistêmico causado pelo diabetes.

##### 4.3.2.4. Refluxo Gastroesofágico

Essa é uma condição clínica que se associa à FA devido à irritação inflamatória direta do esôfago sobre o AE.^55,56^ Além disso, reflexos vagais locais parecem ser outro fator predisponente à essa taquiarritmia, particularmente em indivíduos com coração normal e atletas (maratonistas). Relatos isolados de casos demonstram que o tratamento com bloqueadores de bombas de próton pode reduzir o risco de FA em pacientes acometidos.^57^

A hérnia de hiato pode ser um fator deflagrador de episódios de FA devido ao seu efeito mecânico comprimindo a parede atrial e gerando extrassístoles. Como essa condição é mais frequente em pacientes idosos, outros fatores de confusão (HAS, obesidade, diabetes) podem predispor à FA além da hérnia hiatal propriamente. No entanto, algumas publicações demonstram a redução de episódios de FA após a correção cirúrgica da hérnia, sugerindo, assim, uma relação direta de causa e efeito.^57^

##### 4.3.2.5. Infecções Sistêmicas

Estudos demonstram a associação entre infecções sistêmicas (gastrointestinais, respiratórias, urinárias) com maior risco de FA, além de maior risco de complicações futuras durante a internação e após a alta hospitalar.^58,59^ Essa é uma condição clínica que predispõe ao desencadeamento de episódios agudos de FA, provavelmente em indivíduos já predispostos, mas ressalta uma importância clínica extraordinária devido à gravidade da evolução dos pacientes após a alta. Vale lembrar que, nos quadros infecciosos, o estresse oxidativo e as citocinas inflamatórias deflagram a instabilidade do circuito arritmogênico em AE já remodelado, dando origem à FA.

##### 4.2.2.6. Outras Condições

**Figura 9:**
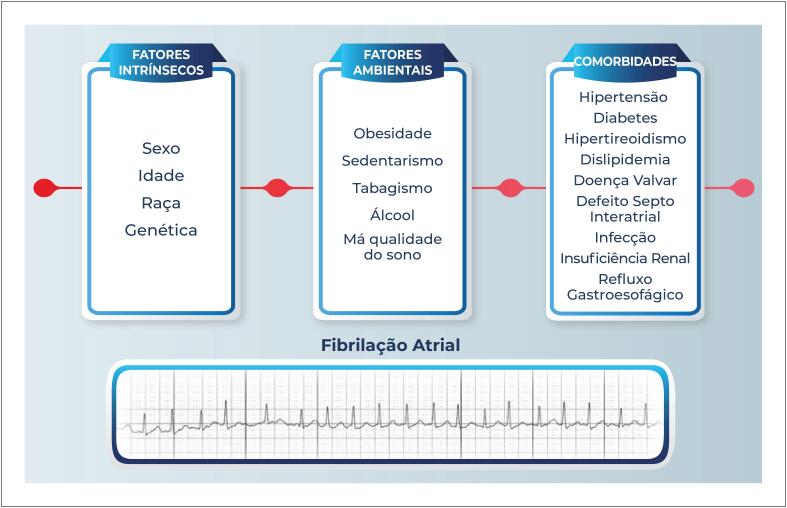
Fatores de risco para fibrilação atrial.

Inúmeras outras condições podem predispor à FA. A doença valvar mitral, especialmente a estenose mitral moderada ou grave, é uma condição que frequentemente acompanha alterações do ritmo cardíaco. Outras condições associadas ao hiperfluxo pulmonar com os defeitos do septo interatrial também se associam a maior ocorrência de FA quando comparadas com a população geral. Mesmo após o tratamento, a ocorrência de *shunt* residual também se associa a maior probabilidade de FA.^60^ Outras condições não cardíacas como hipertireoidismo, insuficiência renal e doença pulmonar obstrutiva crônica (DPOC) também favorecem a FA (Figura 9).

### 4.4. Hábitos de Vida

O alto número de casos de FA observados na prática clínica não pode ser explicado exclusivamente pelo envelhecimento populacional. Outros fatores de risco desempenham um importante papel no desenvolvimento dessa arritmia. Recentemente, fatores comportamentais associados ao estilo de vida foram implicados na pior evolução da FA, oferecendo novas oportunidades de melhor controle clínico com mudanças relacionadas ao estilo de vida.

#### 4.4.1. Obesidade

A ocorrência de obesidade aumentou de forma significativa no Brasil e no mundo. A prevalência aumentou de 11,8 para 20,3% entre os anos de 2006 e 2019. Infelizmente, a prevalência projetada para 2030 no Brasil é de 68,1% para sobrepeso e de 29,6% para obesidade.^61^ Uma importante metanálise incluindo 626.603 indivíduos demonstrou um aumento no risco de FA em 29% para cada aumento de 5 unidades no índice de massa corporal (IMC).^62^ Além disso, uma subanálise do estudo RE-SPECT ESUS (*Randomized, Double-Blind, Evaluation in Secondary Stroke Prevention Comparing the Efficacy and Safety of the Oral Thrombin Inhibitor Dabigatran Etexilate Versus Acetylsalicylic Acid in Patients With Embolic Stroke of Undetermined Source*), um estudo randomizado, duplo-cego, para prevenção secundária de AVC em pacientes com AVC embólico de origem indeterminada, demonstrou que o indivíduo com FA e obeso apresenta um maior risco de AVC, tromboembolismo e morte.^63^ A genética também parecer justificar essa associação. Um estudo com mais de 50.000 indivíduos demonstrou que as variantes genéticas associadas ao IMC alto se correlacionam com a incidência de FA, sugerindo uma relação causal entre as duas condições.^64^

Vários estudos prospectivos foram conduzidos para avaliar o efeito da redução do peso na ocorrência de FA.^65-69^ O estudo LEGACY (*Long-Term Effect of Goal directed weight management on Atrial Fibrillation Cohort: A 5 Year follow-up study*) incluiu 355 pacientes que foram acompanhados por 4 anos e divididos em três grupos de acordo com a perda de peso no final do estudo. Uma probabilidade seis vezes maior de estarem livres de anormalidades do ritmo foi observada nos participantes com perda e manutenção de peso superior a 10% do peso corporal quando comparado com o grupo que perdeu menos de 3% ou ganhou peso no período.^66^ Em outro estudo prospectivo e observacional, com 149 pacientes com IMC superior a 27kg/m^2^ submetidos a ablação de FA, os pacientes foram submetidos a um programa de redução de peso presencial, demonstrando maior tempo de sobrevida livre de eventos arrítmicos quando comparado com o grupo-controle.^65^ Dessa forma, manter um peso saudável parece contribuir para a prevenção e redução da recorrência de FA; todavia, vale apontar que alguns estudos mostram que a baixa massa magra pode se associar a FA^70^ e talvez apenas o emagrecimento sem uma visão integrada do controle de fatores de risco pode ser ineficiente para prevenção de recorrências.^71^

#### 4.4.2. Sedentarismo

O estudo CARDIO-FIT (*Evaluating the Impact of a Weight Loss on the Burden of Atrial Fibrillation [AF] in Obese Patients*) avaliou o impacto da perda de peso na capacidade cardiorrespiratória na ocorrência de FA em pacientes obesos e com sobrepeso. A cada equivalente metabólico adquirido durante o protocolo, houve 9% de redução na recorrência da arritmia mesmo após a correção para o peso e outros fatores de risco.^72^ Além disso, a prática regular de atividade física melhora a capacidade ao esforço, a QV e a fração de ejeção do ventrículo esquerdo (FEVE) em pacientes portadores de FA.^73^ Mais recentemente, foi demonstrado que 1 ano de treinamento aeróbico melhora a função endotelial e está associado à redução de marcadores trombogênicos e pró-inflamatórios no paciente portador de FA.^74^

Por outro lado, a relação entre atividade física e FA parece não ser linear, e sim uma curva em "U", ou seja, os extremos, seja o sedentarismo ou a prática extenuante de exercícios, aumentam o risco de FA.^75,76^ Vale lembrar que se trata da prática de exercícios em doses muito altas que excedem a recomendação e correspondem a uma porcentagem muito pequena da população; entretanto, conhecer o limite entre o saudável e o prejudicial ainda é um dilema na literatura.^77^ Um estudo clínico em andamento para avaliar o destreino de atletas de alto desempenho com FA poderá contribuir para o entendimento da relação entre exercício físico e FA.^78^

#### 4.4.3. Má Qualidade de Sono

Em um estudo epidemiológico nacional, a ocorrência de arritmia cardíaca noturna foi mais frequente nos pacientes portadores de apneia obstrutiva do sono (AOS) grave, definida como índice de apneia/hipopneia (IAH) superior a 30 eventos/hora.^11^ Vários estudos revelam que a ocorrência de AOS em pacientes com FA é muito alta, variando entre 60 e 82%.^79,80^ De fato, tratam-se de condições que compartilham fatores de risco, e a demonstração de casualidade é árdua na literatura científica. Todavia, em um estudo prospectivo, com pacientes referidos para a cardioversão elétrica de FA/FLA atrial, observou-se 82% de recorrência nos pacientes com AOS sem tratamento adequado e 42% de recorrência nos pacientes tratados (p = 0,013).^81^ Além disso, no grupo de pacientes não tratados, a recorrência foi ainda maior entre os que apresentavam maior queda na saturação de oxigênio durante o evento de apneia (p = 0,034). O tratamento da AOS reduz o risco de recorrência de FA não somente em pacientes submetidos a cardioversão elétrica, mas também após ablação por cateter. Em um estudo observacional com 426 pacientes submetidos ao isolamento elétrico das veias pulmonares, 62 pacientes apresentaram AOS confirmada pela polissonografia, sendo 32 pacientes usuários de pressão positiva contínua nas vias aéreas (CPAP) e 30 pacientes sem tratamento. O uso de CPAP foi associado a uma maior taxa de sobrevida livre de FA quando comparado ao grupo sem o uso de CPAP (71,9% vs. 36,7%; p = 0,01). Os autores concluíram que o tratamento com CPAP em pacientes portadores de AOS submetidos a tratamento percutâneo da FA melhora a recorrência da arritmia, e, nos casos de AOS sem tratamento adequado, o isolamento elétrico tem pouco valor clínico.^82,83^ Uma metanálise foi realizada para determinar o papel da AOS no paciente portador de FA submetido a ablação por cateter, concluindo-se que a presença de AOS está associada a maior risco de recorrência de FA após ablação (RR 1,25; p = 0,003).^84,85^

Por outro lado, um pequeno estudo randomizado, com o objetivo de avaliar a recorrência e a carga de FA após isolamento elétrico das veias pulmonares, não demonstrou benefício no uso de CPAP em comparação com o grupo-controle.^86^ Esta diretriz recomenda a adequada avaliação do sono em pacientes com FA; entretanto, estudos randomizados são necessários para esclarecer o papel da CPAP na prevenção e no tratamento da FA.^87^

#### 4.4.4. Uso de Álcool

Os efeitos do consumo de álcool no remodelamento atrial e no sistema nervoso autônomo podem, em parte, justificar o aumento na recorrência de FA. Um dado interessante é que a abstinência de álcool se relaciona à redução da recorrência da arritmia.^88^ Uma metanálise com nove estudos observacionais incluindo 5.436 pacientes submetidos à ablação por cateter para tratamento de FA demonstrou que pacientes que consomem doses moderadas a altas de álcool apresentam maior risco de recorrência.^89^ Em um estudo multicêntrico, prospectivo e randomizado realizado nos hospitais da Austrália, foram selecionados pacientes com consumo superior a 10 doses semanais com FA paroxística ou permanente e que estavam em ritmo sinusal na avaliação basal. O grupo foi selecionado 1:1 para continuar com o uso habitual e abstinência de álcool. Um total de 140 pacientes foram inclusos. A recorrência de FA ocorreu em 53% dos pacientes do grupo de abstinência e em 73% no grupo-controle. O tempo para a primeira recorrência foi maior no grupo de abstinência, e o número total de eventos após 6 meses de acompanhamento foi significativamente menor nos que pararam com o uso em comparação aos controles.^90^

#### 4.4.5. Tabagismo

O uso de tabaco foi recentemente associado ao desenvolvimento de FA (*hazard ratio* [HR], 1,51-2,05), com um efeito dose-resposta (maior risco no tercil mais alto, > 675 anos-cigarro).^91-93^ O abandono do hábito de fumar reduz o risco de FA. Em um estudo que envolveu 15.792 pacientes, aqueles que conseguiram parar de fumar diminuíram o risco dessa arritmia em 36%.^91^

Os mecanismos envolvidos na gênese da FA provavelmente incluem o papel inflamatório da nicotina; o aumento da atividade simpática; a elevação aguda da pressão arterial; a hipoperfusão miocárdica secundária à doença arterial coronariana (DAC); e doença pulmonar obstrutiva crônica (DPOC). O hábito persistente de fumar também está associado à maior recorrência da FA após a ablação por cateter das veias pulmonares, e a inflamação causada pela nicotina pode estar envolvida nesse processo.^94^

O abandono do hábito de fumar pode reduzir os efeitos deletérios da nicotina sobre a atividade elétrica atrial e melhorar os desfechos da FA.^95^

#### 4.4.6. Consumo de Cafeína

A relação entre FA e o consumo de café está em investigação e, até o momento, não há estudos que suportem o malefício da cafeína na FA. Pelo contrário, uma análise de duas coortes na Espanha demonstrou que usuários intermediários de cafeína (1 a 7 xícaras/semana) apresentaram redução do risco de FA.^96^ Resultado semelhante foi demonstrado por um estudo prospectivo com 18.960 participantes, em que a ingesta de uma a três xicaras de café/dia foi associada à redução no risco de FA.^97^ Uma recente metanálise sobre o assunto falhou em demonstrar o prejuízo do consumo de café na ocorrência de FA.^98^

## 5. Diagnóstico de Fibrilação Atrial

O diagnóstico da FA depende do registro eletrocardiográfico de uma arritmia que se caracteriza por ondas P sinusais não discerníveis, ciclos RR irregulares (com condução AV preservada), atividade elétrica atrial não coordenada e ciclos entre as deflexões correspondentes à despolarização atrial (ondas f) usualmente < 200 ms.^16^ As características da FA que tornam o seu diagnóstico ocasionalmente difícil são o seu caráter muitas vezes assintomático e intermitente. Nem sempre a arritmia é detectada em sucessivos ECG de consultório ou de serviços de urgência. Portanto, monitorizações ambulatoriais prolongadas do ECG são necessárias sempre que o diagnóstico for suspeitado. As tecnologias disponíveis para triagem de FA são o Holter de 1 a 7 dias, monitores externos de eventos tipo Loop (monitorizações de 1 a 4 semanas), dispositivos vestíveis como *smartwatches* e aplicativos de celulares, além dos dispositivos cardíacos eletrônicos implantáveis (DCEI), como o monitor de eventos e os dispositivos de estimulação cardíaca artificial.^20^ Vale lembrar que, quando um ritmo atrial rápido é documentado, se faz necessário o registro eletrocardiográfico da arritmia, validado por um médico, convencionando-se que o episódio de FA deva durar ao menos 30 segundos para ser considerado FA clínica (ver Item 3.2).

**Figura 10:**
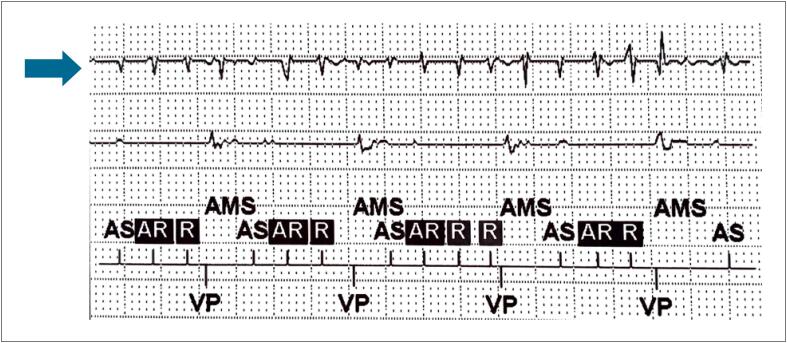
Registro de episódio de ritmo atrial acelerado em análise de dispositivo cardíaco eletrônico implantável. O canal atrial (seta) mostra eletrogramas atriais muito rápidos, irregulares e compatíveis com fibrilação atrial. Figura cedida pelo Dr Thiago da Rocha Rodrigues.

A FA subclínica corresponde a episódios assintomáticos com observação de ritmo atrial acelerado em DCEI. Com o objetivo de reduzir a presença de artefatos, normalmente considera-se um ritmo atrial acelerado os episódios com frequência ≥ 175 bpm avaliados e atestados por um especialista, como exibido na Figura 10.

Define-se como "carga" de FA, ao longo de um monitoramento contínuo do ECG, o tempo total de detecção de FA durante um período específico. A maioria dos estudos considera o período de monitorização de 24 horas como base do cálculo.^99^

### 5.1. Manifestações Clínicas

Quando a FA se manifesta clinicamente, um amplo espectro de apresentações pode ocorrer (Tabela 3). Alguns sintomas são sutis e facilmente ignorados, enquanto outros podem afetar significativamente a QV do paciente e representar sérios riscos à saúde. Os pacientes com FA paroxística tendem a relatar mais sintomas (80%) do que aqueles com FA permanente (51%), sendo que, nesse último perfil, há relato com mais frequência de dispneia, fadiga e intolerância ao esforço.^100^

**Tabela 3:** Resumo das manifestações clínicas relacionadas à fibrilação atrial

Palpitações
Fadiga
Dispneia e insuficiência cardíaca
Desconforto precordial e/ou precordialgia
Tontura e síncope
Acidente vascular cerebral e tromboembolismo sistêmico
Intolerância ao exercício
Ansiedade e depressão
Declínio cognitivo e risco de demência
Hospitalização e piora da qualidade de vida
Aumento da mortalidade
Outras manifestações (poliúria, nictúria, sintomas relacionados a outras comorbidades – hipertireoidismo/apneia do sono/doenças autoimunes, sistêmicas etc.)

As palpitações são os sintomas mais comuns nos indivíduos com FA.^101^ Os pacientes geralmente descrevem uma pulsação rápida e irregular na região precordial, às vezes como "sensação de algo balançando no peito", que pode ser intermitente ou persistente. As palpitações podem estar associadas a ansiedade, tontura e dispneia.^101,102^ Nos pacientes que não se queixam de palpitações, em geral os sintomas da FA costumam ser mais sutis, principalmente em idosos com disfunção do sistema de condução e FA com baixa resposta ventricular.^103^ A FA assintomática tem sido associada a um prognóstico menos favorável. Esse achado, aparentemente paradoxal, deve-se ao fato de que o reconhecimento da arritmia permite a adoção de medidas terapêuticas que possibilitam o seu melhor controle clínico, bem como o menor índice de complicações.^104^

Fadiga e fraqueza generalizada também são frequentes em pacientes com FA. Tanto a irregularidade do ritmo quanto a alta resposta ventricular podem diminuir o débito cardíaco, levando à redução do fornecimento de oxigênio aos tecidos e a uma sensação de cansaço.

A FA pode causar dispneia, principalmente durante o esforço físico. A irregularidade das contrações atriais compromete o enchimento ventricular efetivo, levando à diminuição do débito cardíaco. Os pacientes podem apresentar dispneia, de forma insidiosa e progressiva chegando a mínimos esforços. Cerca de 30 a 40% dos pacientes com FA podem desenvolver IC em algum momento do diagnóstico ou tratamento, do mesmo modo que em torno de 30 a 40% dos pacientes com IC podem evoluir com quadro de FA.^104,105^ A incidência de IC com fração de ejeção preservada (ICFEp) foi duas vezes maior em pacientes com FA do que em pacientes sem FA.^106^ Os níveis elevados de peptídeo atrial natriurético, frequentemente encontrados na FA, podem refletir a coexistência de ICFEp.^107^ Por outro lado, a FC cronicamente elevada pode desencadear um quadro de piora da função sistólica do ventrículo esquerdo (VE) conhecido como taquicardiomiopatia. Inicialmente essa disfunção pode ser assintomática, mas a persistência da elevação da FC promove piora progressiva da FEVE, levando a quadros de IC e miocardiopatia dilatada. É importante o diagnóstico diferencial entre FA secundária à taquicardiomiopatia e a FA no contexto de uma IC preexistente. De um modo geral, a taquicardiomiopatia apresenta normalização da FEVE após a reversão da arritmia. Além disso, a elevação de biomarcadores como o BNP é mais comum nos pacientes com IC primária.

Desconforto no peito ou angina podem ocorrer em alguns pacientes com FA. Embora a arritmia em si não seja diretamente responsável pela isquemia miocárdica, a coexistência com DAC subjacente pode contribuir para a dor torácica.^108^ Além disso, frequências ventriculares rápidas associadas à FA podem aumentar a demanda por oxigênio do miocárdio, exacerbando os sintomas de angina.

Episódios de tontura ou pré-síncope são manifestações comuns de FA. O débito cardíaco reduzido resultante de ritmos cardíacos irregulares e rápidos pode levar à hipoperfusão cerebral, causando tontura. Em casos graves, o fluxo sanguíneo inadequado para o cérebro pode resultar em síncope, especialmente nos pacientes com disfunção diastólica. Além disso, a associação de FA com disfunção sinusal ou distúrbio de condução AV pode ocasionar bradiarritmias significativas, com longas pausas após a reversão dos episódios na FA paroxística relacionada à doença do nó sinusal (síndrome taqui-bradi), ou FC extremamente baixa nos bloqueios AV de grau avançado ou total, muitas vezes associados ao uso de medicamentos antiarrítmicos.

Uma preocupação na FA é o aumento do risco de AVC e tromboembolismo sistêmico periférico. A atividade atrial caótica promove a estase sanguínea nos átrios, predispondo à formação de trombos que podem ser fonte emboligênica, em especial para o cérebro, causando o AVC isquêmico (AVCi). Cerca de 15 a 30% de todos os AVCi têm como causa a FA. Os AVCs em pacientes com FA costumam ser mais graves e debilitantes do que os de outra etiologia.^109^ Uma metanálise que avaliou os eventos TE relacionados à FA demonstrou que a incidência de tromboembolismo sistêmico foi inferior à da embolia cerebral (0,24 contra 1,92/100 pessoas-ano), compreendendo 12% de todos os eventos TE clínicos, mas com um risco comparável de morte, como também de AVCi durante o seguimento. Quanto à localização, cerca de 60% desses eventos envolveu as extremidades inferiores, 30%, os sistemas viscerais/mesentéricos e apenas 11%, as extremidades superiores.^110^

Os pacientes com FA podem apresentar tolerância reduzida ao exercício. Esse sintoma é particularmente referido em atletas que desenvolvem FA,^111^ em especial os de alto rendimento. O ritmo cardíaco irregular e sua resposta ineficiente podem limitar a capacidade do coração de responder às demandas metabólicas aumentadas durante a atividade física. Consequentemente, os indivíduos com FA podem sentir-se rapidamente fatigados ou incapazes de realizar exercícios que anteriormente toleravam, quase sempre relatando sintomas como "perda de rendimento". Outra situação comum é o aumento desproporcional da FC durante o esforço decorrente da facilitação da condução AV relacionada ao maior estímulo adrenérgico.

A natureza crônica da FA, junto com seus sintomas associados e possíveis complicações, pode ter um impacto significativo no bem-estar mental do paciente.^112^ Ansiedade e depressão são comumente observadas em indivíduos com FA, muitas vezes devido à natureza disruptiva da arritmia, medo de complicações e limitações impostas nas atividades diárias. Além disso, pode ocorrer declínio cognitivo e demência. Estudos apontam que a hipoperfusão cerebral crônica, microembolias e/ou microsangramentos, bem como inflamação e lesões de substância branca, são fatores responsáveis pelos quadros demenciais relacionados à FA. O declínio cognitivo pode ocorrer independentemente da história de AVC clínico pregresso. Por outro lado, não é incomum nesses pacientes antecedentes familiares de quadros demenciais de surgimento mais precoce.^113,114^

A FA pode, ocasionalmente, estar associada a quadro de poliúria e nictúria, principalmente após a reversão da arritmia, devido à liberação do peptídeo atrial natriurético.

### 5.2. Impacto na Qualidade de Vida e Hospitalização

A presença de FA está associada a um aumento significativo nas taxas de hospitalização em todo o mundo,^115^ e QV reduzida foi associada a riscos mais elevados para hospitalização.^116^ O impacto clínico da FA pode variar do indivíduo totalmente assintomático àquele extremamente limitado com comprometimento na QV semelhante a outras cardiopatias. Dorian et al. demonstraram em todos os domínios do questionário Short-Form Health Survey 36 (SF-36) a pior QV em pacientes com FA quando comparados com indivíduos saudáveis, pacientes após angioplastia e infarto do miocárdio.^117^ Além disso, características psicológicas, DAC e diabetes podem desempenhar um papel importante na identificação de indivíduos com maior risco de comprometimento da QV e sintomas mais graves relacionados à FA.^118^ Outro ponto a ser considerado são os fatores psicossociais como ansiedade, distúrbios do sono, estresse relacionado ao trabalho e nível de ruído como contribuintes para a gravidade dos sintomas relacionado à FA.^119^

Um questionário específico de QV em FA foi desenvolvido e validado no Brasil para avaliar, especificamente, pacientes com FA abordando as peculiaridades de sintomas e tratamento dessa condição.^120,121^ É recomendado que a QV também seja considerada entre os objetivos terapêuticos na FA, além da redução da morbimortalidade.

### 5.3. Avaliação Clínica do Paciente com Fibrilação Atrial

O paciente com suspeita ou diagnóstico de FA deve ser avaliado clinicamente com o intuito de caracterizar a apresentação da arritmia, estimativa do seu tempo de início, frequência e duração dos episódios, comportamento da FC, sintomas associados e consequente impacto na QV e nas funções cotidianas. Além disso, a ocorrência de outros sintomas de natureza cardiovascular como dispneia, dor torácica, pré-síncope ou síncope, associados ou não à arritmia, podem revelar sua repercussão clínica, bem como levantar a suspeita da associação de outras doenças cardíacas relacionadas. A identificação de fatores predisponentes para a FA, como HAS, IC, diabetes melito, DAC, obesidade, AOS, sedentarismo, consumo de álcool, hipertiroidismo, entre outros, é de fundamental importância para a determinação posterior das estratégias terapêuticas a serem implementadas. Os antecedentes cardiovasculares e a identificação de doenças cardíacas estruturais auxiliam na definição do substrato fisiopatológico da arritmia e seu tratamento adequado. A pesquisa de antecedentes TE, especialmente o AVCi, fatores de risco e hemorrágico são pilares fundamentais na avaliação inicial.

O exame físico deve ser orientado para a pesquisa de sinais de alterações cardiovasculares, como a presença de turgência jugular, edema periférico, congestão pulmonar, pulso irregular, alteração nas bulhas cardíacas, sopros, hipotensão ou HAS. O ECG de 12 derivações é recomendado em todos os pacientes para estabelecer o diagnóstico e avaliar frequência ventricular, distúrbios de condução, isquemias ou outros sinais de doença cardíaca estrutural.

O paciente recém-diagnosticado deve realizar exames laboratoriais básicos que incluem avaliação da glicemia, função renal, perfil lipídico, eletrólitos, hormônios tireoidianos e hemograma com contagem de plaquetas. No ecocardiograma transtorácico, devem ser observadas as dimensões das câmaras cardíacas, função do VE e ventrículo direito (VD), alterações da contratilidade segmentar, possíveis doenças de depósito, diâmetro e volume dos átrios direito (AD) e AE e presença de doenças valvares. Outros exames complementares específicos podem ser utilizados de acordo com o contexto clínico individual dos pacientes. Os exames recomendados para avaliação inicial do paciente com FA estão apresentados no Quadro 3.

**Quadro 3:** Exames recomendados para avaliação inicial do paciente com fibrilação atrial (FA)

Recomendação para avaliação inicial no paciente com FA	Classe de recomendação	Nível de evidência
Avaliação clínica inicial deve incluir história clínica detalhada, eletrocardiograma de 12 derivações e exames laboratoriais (glicemia, função renal, perfil lipídico, eletrólitos, hormônios tireoidianos e hemograma com contagem de plaquetas)	**I**	**A**
Ecocardiograma transtorácico deve ser realizado na primeira avaliação	**I**	**A**

**Quadro 4:** Recomendação para investigação do paciente com fibrilação atrial (FA)

Recomendação para investigação do paciente com FA	Classe	Nível de evidência
A triagem oportunista com a palpação de pulso ou ECG de 12 derivações é recomendada para pacientes acima de 65 anos	**I**	B
A avaliação do paciente com DCEI deve incluir a busca de ritmo atrial acelerado	**I**	B
ECG e Holter de 24 a 72 h podem ser utilizados na avaliação de pacientes com AVCi ou embolia sistêmica que tenham fatores de risco para FA	**I**	B
A triagem sistemática para busca ativa de FA deve ser individualizada de acordo com a suspeita clínica e perfil de risco do paciente	**IIa**	**B**
Monitorização prolongada com monitor de eventos externo ou implantável pode ser utilizada em pacientes diagnosticados com AVCi criptogênico	**IIa**	**B**

AVCi: acidente vascular cerebral isquêmico; ECG: eletrocardiograma; DCEI: dispositivo cardíaco eletrônico implantável.

### 5.4. Triagem no Paciente com Risco de Fibrilação Atrial

A detecção da FA em pacientes com suspeita clínica depende, primordialmente, do tempo de monitorização eletrocardiográfica. A escolha da ferramenta apropriada é de grande importância, de acordo com as características de cada paciente. Os altos custos médico-hospitalares relacionados à FA justificam as várias estratégias propostas para sua detecção e tratamento precoces, especialmente em populações de maior risco.^122^ A triagem diagnóstica da FA pode ser feita de forma oportunista ou sistemática de acordo com as faixas etárias, especialmente nos indivíduos com 65 anos ou mais e naqueles portadores de fatores de risco já mencionados anteriormente.^123,124^

A triagem oportunista é feita por profissional de saúde, através da palpação do pulso ou realização de ECG, durante o atendimento de rotina ou avaliação de algum sintoma. Por outro lado, na triagem sistemática, programas específicos estimulam a observação do comportamento do ritmo cardíaco de forma disciplinada que pode ser realizada pelo próprio paciente, com avaliações periódicas utilizando dispositivos de detecção não invasiva ou programas específicos para busca ativa de FA. Uma abordagem custo-efetiva foi baseada na palpação de pulso ou no uso de dispositivos portáteis de ECG. Tanto a triagem oportunista quanto a sistemática são mais custo-efetivas do que a avaliação clínica de rotina para pacientes com mais de 65 anos. Campanhas comunitárias para palpação de pulso, com orientação do paciente de como realizar essa manobra simples, são uma opção para a população geral.^125-127^

Em nosso meio, entre as formas não invasivas de triagem diagnóstica, além da palpação periódica do pulso periférico, dispomos do ECG intermitente, do sistema Holter (entre 24 h e 7 dias) e dos monitores de eventos (*loop recorders)* externos, que monitoram o ECG continuamente por 2 semanas ou mais, dependendo da tolerância do paciente. Estudos demonstram que a realização frequente do ECG de 12 derivações aumentou em quatro vezes a detecção da arritmia, quando comparada a gravação realizada em avaliação clínica anual de rotina. Gravações repetidas do ECG num período de duas semanas, detectou FA assintomática em 7,4% dos indivíduos com dois ou mais fatores de risco para AVC.^125^

**Figura 11:**
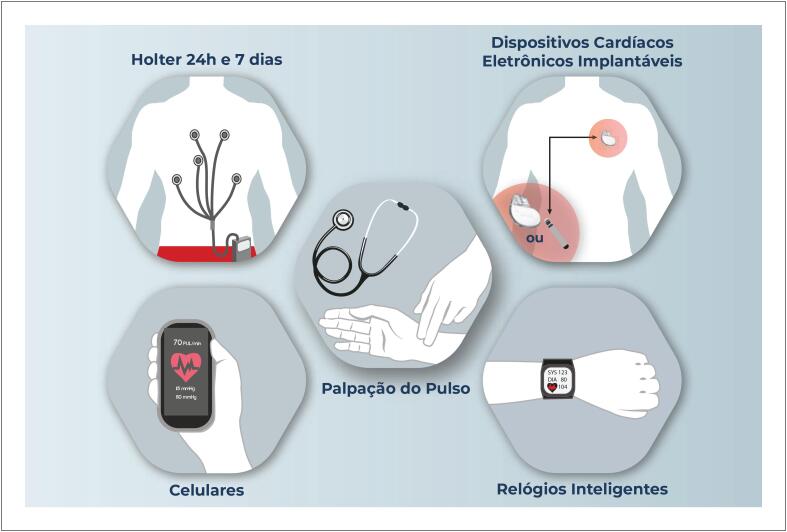
Ferramentas utilizadas para triagem de fibrilação atrial/flutter atrial.

Os dispositivos de avaliação não invasiva do ritmo cardíaco vêm se tornando cada vez mais populares. Os aparelhos portáteis ou vestíveis que detectam o pulso por meio de pletismografia (oxímetros de pulso, aparelhos aferidores de pressão arterial automáticos, pulseiras, relógios inteligentes) são formas simples e acessíveis para a identificação de pulsos irregulares compatíveis com FA.^128^ O registro eletrocardiográfico domiciliar por meio de dispositivos não invasivos como os relógios inteligentes ou interfaces-eletrodos para registro do ECG através de aplicativos em dispositivos móveis é capaz de fornecer um traçado com uma ou mais derivações e definição suficiente para o diagnóstico preciso, que deve ser validado por médico com conhecimento na área de ECG. Estudos prévios com elevado número de pacientes já demonstraram a acurácia dos traçados eletrocardiográficos obtidos por relógios inteligentes na detecção de FA.^13,129^ É importante salientar que nem todos os dispositivos estão homologados pela Agência Nacional de Vigilância Sanitária (ANVISA) para uso no território brasileiro, sendo essa regulamentação necessária para a sua validação e utilização clínica. A Tabela 4 traz o resumo da sensibilidade e especificidade dos principais métodos utilizados na detecção de FA. A Figura 11 demonstra as principais ferramentas utilizadas para triagem de FA.

**Tabela 4:** Sensibilidade e especificidade das formas de triagem para detecção de fibrilação atrial (considera-se o eletrocardiograma de 12 derivações como padrão-ouro)

Tipo de monitorização	Especificidade	Sensibilidade
Palpação do pulso	87-97%	70-81%
Monitores de pressão arterial	93-100%	86-92%
Eletrocardiograma	98%	76-95%
Aplicativos para smartphone	91,5-98,5%	91,4-100%
Relógios Inteligentes	99%	83-94%

Adaptado de Hindricks et al.^17^

### 5.5. Monitorização Eletrocardiográfica Prolongada

Em pacientes que utilizam algum tipo de DCEI, a avaliação periódica desses dispositivos deve incluir a pesquisa dos eventos de ritmo atrial acelerado que caracterizam a FA subclínica. O estudo ASSERT (*Asymptomatic Atrial Fibrillation and Stroke Evaluation in Pacemaker Patients and the Atrial Fibrillation Reduction Atrial Pacing Trial*) que avaliou 2.580 pacientes acima de 65 anos com DCEI demonstrou incidência de ritmo atrial acelerado com duração > 6 minutos de 10,1%, com aumento no risco de detecção de FA clínica e AVCi nessa população.^130^

A FA responde por cerca de 15% de todos os AVCi.^130^ Um problema frequente na prática clínica é a pesquisa de FA em indivíduos que apresentaram AVCi criptogênico, principalmente os não lacunares atribuídos a provável causa TE de fonte desconhecida, que correspondem a cerca de 20 a 40% dos casos de AVCi.^131^ Uma eventual detecção de FA nesses pacientes pode determinar a necessidade de anticoagulação oral em substituição à antiagregação plaquetária. O estudo EMBRACE (*30-Day Cardiac Event Monitor Belt for Recording Atrial Fibrillation After a Cerebral Ischemic Event: A Randomized Controlled Trial*) mostrou que quanto maior o tempo de monitorização eletrocardiográfica num período de até 30 dias após o AVCi ou ataque isquêmico transitório, maior a possibilidade de detecção de FA. A utilização do gravador de *loop* por 30 dias (com detecção automática de FA) foi superior ao Holter para a detecção de FA com duração > 30 segundos (16,1% contra 3,2%; p < 0,001) em pacientes com AVCi ou ataque isquêmico transitório nos últimos 6 meses.^131^ No estudo CRYSTAL AF (*CRYptogenic STroke And underLying AF Trial*), a monitorização com *loop* implantável por 12 meses detectou FA em 12,4% dos pacientes com AVCi criptogênico contra 2,0% naqueles com monitorização convencional (ECG e Holter), com RR de 7,3 (p < 0,001).^132^

## 6. Fibrilação Atrial e Fenômenos Tromboembólicos

### 6.1. Algoritmos para Avaliação do Risco de Fenômenos Tromboembólicos

A FA é a principal causa de eventos TE de origem cardíaca.^133^ O risco de AVC na vigência de FA dobra a cada década de vida após os 55 anos de idade, sendo a sua incidência 25% maior em pacientes acima de 80 anos. A FA é responsável por quase um terço de todos os eventos isquêmicos cerebrais, sendo o principal mecanismo a cardioembolia.^134^

Fatores locais como a redução da velocidade do fluxo no AAE, dilatação, lesão endocárdica, inflamação e fibrose atrial participam do mecanismo fisiopatológico da formação do trombo na FA^135^ e, desde que demonstrou-se a capacidade dos anticoagulantes orais de ação direta (ACOD) em reduzir a ocorrência de eventos TE, os escores de risco têm sido elaborados, validados e amplamente utilizados na decisão quanto à introdução da terapêutica anticoagulante em pacientes com FA. O mais utilizado é o escore CHA_2_DS_2_-VA, anteriormente chamado de CHA2DS2-VAsC (Tabela 5). A iniciativa da Sociedade Europeia de Cardiologia para a mudança da nomenclatura do escore, abolindo o sexo feminino do sistema de pontuação, reside no fato de ser um fator modificador de risco, além de excluir um grupo grande de pacientes com identidade não binária, transgêneros ou em uso de terapia hormonal. Essa modificação foi endossada pelo seu caráter inclusivo, e o termo CHA_2_DS_2_-VA será utilizado neste documento.

**Tabela 5:** Escore de CHA_2_DS_2_-VA

CHA_2_DS_2_-VA	Pontuação
*Congestive heart failure/left ventricular dysfunction* (insuficiência cardíaca congestiva/disfunção ventricular esquerda)	1
*Hypertension* (hipertensão arterial)	1
*Age ≥ 75 yrs* (idade ≥ 75 anos)	2
*Diabetes mellitus* (diabetes melito)	1
*Stroke/transient ischaemic attack/ Thrombo-embolism* (histórico de AVC/AIT/embolia sistêmica)	2
*Vascular disease* (*prior myocardial infarction, peripheral artery disease or aortic plaque*) (doença vascular)	1
*Age 65–74 yrs* (idade)	1

AVC: acidente vascular cerebral; AIT: ataque isquêmico transitório.

O escore CHA_2_DS_2_-VA apresenta alta sensibilidade e valor preditivo negativo para definir, principalmente, a população de baixo risco, porém, com baixa especificidade para detectar pacientes de alto risco.^136-138^

A anticoagulação oral é recomendada para pacientes com escore CHA_2_DS_2_-VA ≥ 2. Pacientes com escore zero, o risco é considerado baixo (1,3% ao ano), e a terapia anticoagulante deve ser individualizada de acordo com o perfil de risco, sangramento e preferência do paciente. A anticoagulação em paciente com CHA_2_DS_2_-VA = 0 não é necessária. A forma de apresentação e a carga da FA têm demonstrado implicações como fator de risco adicionais para eventos TE, com maior risco em pacientes com FA persistente e com carga maior de FA.^139^

Outros escores como o ABC-AF-stroke/bleeding (*age, biomarkers and clinical history*)^140^ e o Atria (*Anticoagulation and Risk Factors in Atrial Fibrillation*)^141^ incluem o uso de biomarcadores (peptídeo natriurético pró-tipo B N-terminal, troponina-T cardíaca e fator de diferenciação de crescimento 15) e presença de disfunção renal, respectivamente. Já o Garfield-AF Risk Tool baseia-se em um modelo de predição de risco gerado por computador (*machine learning*) que prevê mortalidade por todas as causas, AVCi e hemorrágico e embolia sistêmica em pacientes com FA.^142^ Apesar de esses escores discriminarem bem os pacientes com baixo e alto risco de AVC e sangramento, a praticidade e o custo adicional ainda restringem seu uso na prática clínica.

### 6.2. Algoritmos para Avaliação do Risco de Sangramento

Diversos fatores que determinam maior risco TE associam-se a maior ocorrência de eventos hemorrágicos. Portanto, a correção ou o ajuste dos fatores modificáveis, quando possível, é fundamental no balanço entre o risco e o benefício da terapia com anticoagulante.

Em uma revisão sistemática com 38 estudos, o escore HAS-BLED apresentou melhor evidência na predição de risco de sangramento (Tabela 6).^143^ Valores ≥ 3 indicam alto risco, mas não constituem contraindicação à terapia anticoagulante. Nesses, o melhor controle da HAS, evitar o uso concomitante de anti-inflamatórios ou álcool e substituir a varfarina por um ACOD são condutas sugeridas para mitigar o risco.^134,144^

**Tabela 6:** Escore HAS-BLED para avaliação de risco de sangramento

Risco HAS-BLED	Pontuação
*Hypertension* (hipertensão arterial) – PAS >160 mmHg	1
*Abnormal renal or liver function* (alteração da função renal ou hepática) *Clearance* de creatinina ≤ 50 mL/min ou creatinina ≥ 2,26 mg/dL ou hemodiálise ou transplante renal; bilirrubina ≥ 2x VN + TGO ou TGP ou FA ≥ 3x VN ou cirrose hepática	1 ou 2
*Stroke* (AVC prévio)	1
*Bleeding* (sangramento prévio ou predisposição a sangramentos)	1
*Labile INRs* (INR lábil ou < 60% do tempo na faixa terapêutica)	1
*Elderly* (idade > 65 anos)	1
*Drugs or alcohol* (uso de drogas [AINE/AINH, antiplaquetários] ou abuso de álcool [> 20 U por semana])	1 ou 2

PAS: pressão arterial sistólica; AVC: acidente vascular cerebral; VN: valores normais; TGO: transaminase glutâmico oxalacética; TGP: transaminase glutâmico pirúvica; FA: fosfatase alcalina; AINE: anti-inflamatórios não esteroides; AINH: anti-inflamatórios não hormonais; INR: razão normalizada internacional. Adaptado de Steffel et al.^144^

O escore ORBIT, que considera fatores como idade avançada, baixos valores de hemoglobina, antecedente de sangramento, disfunção renal e uso concomitante de antiplaquetário,^145^ não foi melhor do que o escore HAS-BLED na predição de eventos hemorrágicos maiores em pacientes anticoagulados por FA.^146^

O risco de sangramento é dinâmico, e a reavaliação periódica dos fatores de risco é fundamental. No estudo mAFA-II, o monitoramento dinâmico prospectivo com a reavaliação usando a pontuação HAS-BLED, juntamente com o gerenciamento holístico baseado em aplicativo, foi associado a menores taxas de eventos hemorrágicos graves e ao aumento do uso de anticoagulantes comparado com o grupo com cuidados habituais aos 12 meses de seguimento.^147^

As recomendações para prevenção de fenômenos TE em pacientes com FA não valvar estão sumarizadas no Quadro 5.

**Quadro 5:** Recomendações para prevenção de fenômenos tromboembólicos na fibrilação atrial (FA) não valvar

Recomendação para prevenção de fenômenos tromboembólicos na fibrilação atrial não valvar	Classe de recomendação	Nivel de evidência
O escore CHA_2_DS_2_-VA deve ser empregado em todos os pacientes	**I**	**A**
Em pacientes com escore CHA_2_DS_2_-VA ≥ 2, é recomendada a terapia antitrombótica	**I**	**A**
Pacientes de baixo risco, com CHA_2_DS_2_-VA = 0, não têm indicação para terapia antitrombótica	**I**	**A**
Em pacientes com escore CHA_2_DS_2_-VA = 1, a terapia antitrombótica pode ser instituída, levando-se em consideração o benefício clínico, o risco de sangramento, a forma de apresentação da FA e a preferência do paciente	**IIa**	**B**
Anticoagulação oral é recomendada para todos os pacientes com FA e miocardipatia hipertrófica, amiloidose cardíaca e hipertireoidismo a despeito do escore CHA_2_DS_2_-VA, para a prevenção dos eventos embólicos	**I**	**B**
A reavaliação periódica do risco tromboembólico e de sangramento é recomendada nos pacientes com FA	**I**	**B**
Para avaliação do risco de sangramento, o escore HAS-BLED pode ser considerado para auxiliar na identificação dos fatores modificáveis em pacientes de alto risco	**IIa**	**B**
Em pacientes elegíveis a terapia antitrombótica, os ACOD são preferíveis aos AVK, exceto em pacientes com válvula cardíaca mecânica ou estenose mitral moderada a grave	**I**	**A**
Para pacientes em uso de AVK, o RNI deve ter como alvo 2,0-3,0 e deve permanecer mais que 70% do tempo em faixa terapêutica	**I**	**B**
Terapia com antiagregantes plaquetários isolados ou em combinação não é recomendada para prevenção de fenômenos tromboembólicos em pacientes com FA	**III**	**A**
A estimativa do risco de sangramento, na ausência de contraindicações absolutas, não deve guiar a decisão da terapia antitrombótica	**III**	**A**
A anticoagulação oral em pacientes com FA subclínica pode ser considerada com edoxabana ou apixabina, levando-se em consideração risco, benefício e preferência do paciente	**IIa**	**B**

AVK: antagonistas da vitamina K; ACOD: anticoagulantes orais diretos; INR: razão normalizada internacional; AVC: acidente vascular cerebral.

### 6.3. Prevenção de Fenômenos Tromboembólicos

#### 6.3.1. Anticoagulantes Disponíveis na Prática Clínica

Os ACOD são a terapia de primeira linha para a prevenção de tromboembolismo em pacientes com FA, e seu uso é orientado por estimativa do risco de AVC. Em comparação com controle ou placebo, a terapia com antagonista da vitamina K (AVK) reduziu o risco de AVC em 64% e a mortalidade em 26%.^148^

Os AVK são eficazes na prevenção de eventos TE, mas apresentam uma série de limitações na prática clínica como janela terapêutica estreita, farmacocinética e farmacodinâmicas variáveis, grande variedade de interações medicamentosas e alimentares, além da necessidade de monitoramento e ajustes regulares.^134^

Vários ensaios clínicos randomizados estabeleceram a não inferioridade e a superioridade dos ACOD em comparação com a varfarina na prevenção de AVC e embolia sistêmica em pacientes com FA não valvar.^149-152^

Em uma metanálise incluindo os grandes estudos pivotais, os ACOD foram associados à redução de 19% no risco de AVC/embolia sistêmica e 51% no risco de AVC hemorrágico. Além disso, a terapia com ACOD associou-se a uma redução significativa de 10% na mortalidade por todas as causas, com redução de 14% no risco de sangramento maior e de 52% no sangramento intracraniano às custas de um aumento de 25% nas taxas de sangramento gastrointestinal quando comparado com AVK.^153^

Da mesma forma, a análise de dados individuais de pacientes oriundos de ensaios randomizados (COMBINE AF [*Controlled Trials of Non-Vitamin K Antagonist Oral Anticoagulants in Patients with Atrial Fibrillation*]) demonstrou menor risco de AVC ou embolia sistêmica entre os pacientes que receberam ACOD comparados com aqueles tratados com varfarina. O risco de sangramento intracraniano foi reduzido em 55% com o uso de ACOD. As taxas de mortalidade por todas as causas e sangramento maior também foram reduzidas em 8 e 14%, respectivamente. Em relação a sangramento gastrointestinal, os ACOD aumentaram em 31% o RR quando comparados à varfarina.^154^

Há grande quantidade de dados publicados que confirmam, na prática clínica, que os ACOD são pelo menos tão seguros e eficazes quanto a varfarina.^155^ Com quatro ACOD disponíveis em diferentes dosagens para diferentes indicações e com diferentes critérios de redução de dose, adotar uma abordagem personalizada pode ser a melhor estratégia para o manejo clínico do paciente.

#### 6.3.2. Individualização do paciente para decisão da melhor estratégia de anticoagulação

Apesar de os ACOD contribuírem muito para o sucesso da terapia antitrombótica, vários fatores precisam ser levados em consideração na escolha da melhor estratégia de anticoagulação. A varfarina permanece como primeira linha de tratamento para pacientes com FA e estenose mitral moderada e grave de origem reumática ou pacientes com prótese mecânica cardíaca. Nesses pacientes, o RNI-alvo deve ser em torno de 2 a 3. Por outro lado, outras formas de doença valvar, como insuficiência ou estenose aórtica, insuficiência mitral, biopróteses ou plastia valvar, foram incluídas nos estudos pivotais com ACOD, e uma revisão sistemática com esses pacientes demonstrou a eficácia e segurança no uso dessas medicações.^156^

A função renal constitui fator relevante na decisão sobre qual estratégia de anticoagulação utilizar. Pacientes com insuficiência renal facilmente preenchem critérios para anticoagulação; entretanto, paradoxalmente também estão sujeitos a alto risco de sangramento. Além disso, esse grupo de pacientes é frequentemente excluído dos grandes estudos clínicos, tornando ainda mais difícil a escolha sobre qual anticoagulante utilizar. As evidências do uso de anticoagulação na insuficiência renal estágio 4 (taxa de filtração glomerular [TFG] de 15 a 29 mL/min/1,73 m^2^) são em grande parte embasadas em estudos observacionais. Uma análise dos pacientes com baixa depuração de creatinina (*clearance* de creatinina [ClCr] entre 25 e 30 mL/min) do estudo ARISTOTLE (*Apixaban for the Prevention of Stroke in Subjects With Atrial Fibrillation*) demonstrou que apixabana foi eficaz em prevenir AVC e ainda mais segura do que a varfarina quando comparada à segurança da apixabana em pacientes com ClCr maior que 30 mL/min. Portanto, a apixabana demonstra um perfil de segurança único para pacientes com doença renal mais avançada não dialítica.^157^ Pacientes em estágio terminal (ClCr < 15 mL/min) ou que estejam em terapia substitutiva são um desafio na prática clínica. Metanálises de estudos observacionais não conseguiram demonstrar eficácia da varfarina para esses doentes, tendo aumento exponencial do risco de sangramento. Além disso, o controle da RNI é uma tarefa árdua nessa população. Por fim, a varfarina também leva a calcificações patológicas em doentes com função renal terminal. Dessa forma, não se sabe qual a real eficácia da varfarina em doentes com função renal terminal. Um estudo retrospectivo nessa população não encontrou diferenças entre as taxas de eventos TE; entretanto, demonstrou um número menor de sangramentos em pacientes tratados com apixabana *versus* varfarina.^158^ O estudo RENAL-AF (*Renal Hemodialysis Patients Allocated Apixaban Versus Warfarin in Atrial Fibrillation*) tentou avaliar a segurança da apixabana em doentes dialíticos, mas infelizmente o estudo foi interrompido precocemente e passou a ser apenas exploratório, demonstrando eficácia e segurança semelhantes à varfarina nessa população. Assim sendo, não se sabe se há ou não benefício em se anticoagular doentes em diálise. Em casos em que se opta por anticoagulação nessa população, a apixabana parece ser uma opção razoável, embora precisemos de dados mais definitivos. Para maiores informações, ver tabela FA em situações especiais.

Idosos frágeis também apresentam peculiaridades que devem ser levadas em consideração. Um estudo recente avaliou a segurança de trocar a varfarina por um ACOD em pacientes idosos com FA e que vivem com fragilidade (idade acima de 75 anos e escore de fragilidade de Groningen superior a 3). Os autores demonstraram que a troca para um ACOD foi associada a maior risco de sangramento sem redução adicional no risco de fenômenos TE.^159^ No entanto, existem questões metodológicas relevantes que não nos permitem aplicar seus resultados na prática clínica, sendo prudente aguardar novos estudos nessa população. Além de tratar-se de um estudo pequeno e terminado precocemente, os desfechos foram aferidos pelo médico do estudo, o que, potencialmente, é um motivo de viés. O aumento de 69% de sangramento com ACOD em relação à varfarina não possui plausibilidade biológica compatível com a literatura atual. Além disso, 75% dos doentes usaram rivaroxabana, edoxabana e dabigatrana; drogas que já mostraram sangrar mais no trato gastrointestinal (TGI) do que a varfarina, especialmente em idosos. Em resumo, pacientes estáveis em uso de varfarina podem ser mantidos em uso de varfarina, mas, particularmente em pacientes idosos e frágeis, o uso de ACOD pode constituir alternativa segura e eficaz.

A escolha e a dose do anticoagulante devem ser baseadas no perfil geral do paciente, e essa tomada de decisão deve ser revisitada a cada avaliação clínica durante todo o seguimento clínico, seguindo os modelos de ajustes de doses realizados nos estudos pivotais de cada medicamento a fim de se reduzir o risco de AVC ao máximo e minimizar o risco de sangramento.^143^

As características farmacocinéticas e farmacodinâmicas dos ACOD e as doses recomendadas estão representadas nas Tabelas 7 e 8.

**Tabela 7:** Características farmacocinéticas e farmacodinâmicas dos ACOD

	Dabigatrana	Rivaroxabana	Apixabana	Edoxabana
**Posologia**	2x/dia	1x/dia	2x/dia	1x/dia
**Metabolismo por CYP450**	Nulo	32%	15%	< 4%
**Meia-vida (horas)**	12-17	5-13	12	10-14
**Horas até C_máx._**	1-3	2-4	3-4	1-2

9 ACOD: anticoagulantes orais de ação direta; Cmáx.: concentração máxima. Adaptada de Steffel et al.^144^

**Tabela 8:** Doses recomendadas para o uso de ACOD

	Dabigatrana	Rivaroxabana	Apixabana	Edoxabana
**Dose padrão**	150 mg 2x ao dia	20 mg 1x ao dia	5 mg 2x ao dia	60 mg 1x ao dia
**Dose ajustada**	110 mg 2x ao dia*	15 mg 1x ao dia	2,5 mg 2x ao dia	30 mg 1x ao dia
**Critério para mudança de dose**	– Idade igual ou superior a 80 anos – Uso concomitante de verapamil – Risco aumentado para sangramentos	ClCr entre 15 e 49 mL/min	Pelo menos dois dos três critérios: – Idade maior ou igual a 80 anos, – Peso corporal menor ou igual a 60 kg – Creatinina maior ou igual a 1,5 mg/dL	Qualquer dos seguintes critérios: – TFG 15–50 mL/min – Peso corporal menor ou igual a 60 kg – Uso concomitante de dronedarona, ciclosporina, eritromicina ou cetoconazol

Adaptada de Hindricks et al.^17^

* A dose de 110 mg de dabigatrana não é uma dose ajustada, e sim uma dose reduzida testada no estudo RE-LY sem a presença de critérios para ajuste. Uma vez que apresentou menor taxa de sangramento em relação à varfarina e com eficácia semelhante, recomenda-se usá-la em pacientes com risco aumentado de sangramento. ClCr: clearance de creatinina pela fórmula Cockcroft-Gault; kg: quilograma; TFG: taxa de filtração glomerular; ACOD: anticoagulantes orais de ação direta.

### 6.4. Orientações aos Pacientes em Uso de Anticoagulação

Apenas metade dos pacientes com FA e fatores de risco para AVC é tratada com anticoagulantes.^17^ Assim, se faz necessário o desenvolvimento de intervenções que possam aumentar a proporção de pacientes adequadamente tratados. No estudo IMPACT AF (*Clinical Trial to Improve Treatment With Blood Thinners in Patients With Atrial Fibrillation*), uma intervenção personalizada, multifacetada e multinível envolvendo a educação de pacientes com FA e seus provedores, com acompanhamento regular, resultou em aumento significativo na proporção de pacientes tratados com anticoagulação (12% no grupo intervenção contra 3% no grupo-controle, p = 0,0002). Apesar desse aumento no uso de ACOD, não se observou aumento nas taxas de sangramentos, e menores taxas de AVC foram reportadas no grupo intervenção (1% contra 2%, p = 0,043).^160^

A aderência ao tratamento com ACOD é crucial, considerando que o efeito anticoagulante diminui dentro de 12–24 horas após a última ingestão. Embora as taxas de descontinuação sejam significativamente mais baixas com os ACOD comparadas com AVK em decorrência do melhor perfil farmacocinético e segurança e eficácia favoráveis dessa classe de medicamentos, a descontinuação ainda é uma questão relevante.^161^ Idade mais jovem, disfunção renal, consumo excessivo de álcool, escores de CHA_2_DS_2_-VA mais baixos e alto custo constituem importantes causas de descontinuação do tratamento.^162^ Logo, a orientação em relação à aderência é de suma importância e deve ser minuciosamente discutida com o paciente e os familiares no início da terapia anticoagulante e a cada consulta subsequente.

Apesar dos ACOD terem menor interação medicamentosa, recomenda-se a avaliação do potencial de interação com os fármacos que utilizam as vias do citocromo P450 e CYP3A4. Além disso, a monitorização da função renal deve ser criteriosamente avaliada periodicamente em pacientes com função renal reduzida. Quanto menor a TFG, mais frequente deve ser essa avaliação.

Envolver o paciente no processo de decisão e discutir as opções de anticoagulação, em um processo de tomada de decisão compartilhada, são pontos-chave a fim de avaliar adequadamente a necessidade de cada paciente.

Os quesitos importantes para o acompanhamento do paciente em terapia de anticoagulação estão listados na Tabela 9.

**Tabela 9:** Fatores necessários para o acompanhamento do paciente em uso de anticoagulante

	Intervalo	Comentário
**Adesão**	Toda visita	Educação médica Considerar preferência 1x ou 2x dia
**Tromboembolismo**	Toda visita	Avaliação clínica e de imagem quando necessário
**Sangramento**	Toda visita	
**Eventos adversos**	Toda visita	Avaliar criteriosamente a relação com ACOD: troca da medicação ou interrupção temporária.
**Medicações concomitantes**	Toda visita	Interações medicamentosas. Considerar também as de uso temporário.
**Laboratório**	Utilizar fórmula*	Se ClCr < 60 mg/kg/24 h
	4 meses	> 75 anos ou fragilidade
	Anual	Se nenhuma das condições acima
**Fatores modificáveis de sangramento**	Toda visita	Hipertensão não controlada (> 160 mmHg), uso de aspirina e anti-inflamatórios não hormonais; consumo de álcool excessivo
**ACOD e perfil do paciente**	Toda visita	ACOD de acordo com perfil do paciente Dose apropriada para condição clínica

ACOD: anticoagulantes de ação direta; ClCr: clearance de creatinina.

* Para pacientes com ClCr inferior a 60 mg/kg/24 horas, o intervalo de solicitação de exames deve seguir a fórmula: intervalo em meses = ClCr/10. Adaptado de Steffel et al.^144^

#### 6.4.1. Orientações nos Casos de Sangramento com os ACOD

Embora os ACOD tenham mudado a abordagem terapêutica da FA não valvar na prevenção de acidentes TE, as complicações hemorrágicas relacionadas ao uso desses fármacos podem representar uma limitação. A incidência de sangramento maior em pacientes em uso de ACOD é de 2-3% ao ano, sendo a incidência de AVC hemorrágico de 0,1-0,5%.^163^ Dada a meia-vida curta, sangramentos de pequena intensidade podem ser solucionados com a suspensão medicamentosa.

A avaliação do paciente com sangramento ativo em uso de ACOD envolve a detecção do local da hemorragia, a avaliação da gravidade e a informação sobre a última dose do fármaco. Fatores que influenciam o risco de sangramento, como álcool, uso concomitante de aspirina, uso de antitrombóticos e disfunção renal, devem ser explorados. Testes de coagulação mais específicos, como a atividade anti-Xa, podem ser realizados; entretanto, nem sempre estão disponíveis e muitas vezes são desnecessários para o controle do sangramento. Na maioria dos casos, a tomada de decisão clínica adequada está atrelada à ingestão da última dose do ACOD e à avaliação da função renal, hemoglobina, hematócrito e plaquetas.

As características farmacocinéticas são distintas entre os diversos ACOD, e tais peculiaridades podem determinar mudanças na conduta. A dabigatrana, por exemplo, apresenta fraca ligação às proteínas plasmáticas, sendo potencialmente removida por hemodiálise. Rivaroxabana e apixabana, por outro lado, não são substâncias dialisáveis, dada a forte ligação proteica no plasma.^164^

Eventos hemorrágicos menores devem ser tratados com medidas de suporte como compressão mecânica ou pequena cirurgia para obter hemostasia. Os ACODs apresentam meia-vida plasmática curta, e a hemostasia pode ser esperada dentro de 12 a 24 h após uma dose omitida. Por outro lado, o tratamento de eventos hemorrágicos moderados pode exigir transfusões de sangue. Se a última ingestão do ACOD for inferior a 2 a 4 h do início do sangramento, a administração de carvão ativado e/ou lavagem gástrica pode ser considerada para reduzir a exposição adicional. É conveniente salientar que o uso de carvão ativado é contraindicado na vigência de hemorragia digestiva. Ele está disponível sob a forma de pó e pode ser administrado diluído em água ou suco, se o paciente estiver acordado, ou por sonda nasogástrica, na dose de 1 g por kg de peso corporal.

Intervenções específicas para identificação e controle da causa do sangramento (por exemplo, endoscopia digestiva) devem ser realizadas imediatamente. A diálise é eficaz na redução da concentração de dabigatrana e tem sido associada à redução da duração e/ou gravidade do sangramento.

Sangramento grave ou com risco de morte requer reversão imediata do efeito antitrombótico. O uso de antídotos pode ser considerado nesses casos. O idarucizumabe é um fragmento de anticorpo monoclonal que se liga à dabigatrana, com afinidade 350 vezes maior que a observada com a trombina, resultando na neutralização do efeito anticoagulante. A segurança do uso deste medicamento foi demonstrada em diversos estudos incluindo pacientes idosos e com insuficiência renal. No estudo RE-VERSE AD (*A Study of the RE-VERSal Effects of Idarucizumab on Active Dabigatran*), o reversor utilizado em situações emergenciais neutralizou os efeitos anticoagulantes da dabigatrana com segurança e rapidez, sem efeitos pró-coagulantes, em um grupo de 503 pacientes. A reversibilidade do efeito anticoagulante foi obtida em 100% dos pacientes, cerca de 1,6 e 2,5 horas após a administração. Eventos TE ocorreram em 6,3 a 7,4% dos pacientes.^165^

O andexanete é uma proteína recombinante inativa que se liga aos inibidores do fator X ativado (rivaroxabana, apixabana e edoxabana), revertendo o efeito anticoagulante. Reversão satisfatória foi descrita com a administração intravenosa.^166^ No entanto, seu uso está frequentemente associado à subsequente não reiniciação do ACOD e, portanto, a possível aumento nas taxas de eventos trombóticos.

Por fim, o ciraparantag, ainda não oficialmente aprovado, é uma droga sintética que inibe diretamente os inibidores diretos do fator Xa e a enoxaparina por meio de ligações de hidrogênio não covalentes, removendo essas drogas de seu local-alvo com reversão dos efeitos anticoagulantes.^167^

A administração de plasma fresco congelado pode ser realizada, mas, como medida de suplementação de fatores de coagulação, apresenta concentrações muito inferiores às observadas nos concentrados de complexos protrombínicos. A indicação desses concentrados é razoável em situações de hemorragias graves.^168,169^ Embora os antídotos possam ser efetivamente administrados em casos de sangramento grave ou cirurgia de emergência, seu uso é relativamente raro na prática clínica.

O risco de morte após sangramento grave relacionado ao ACOD ainda é significativo, apesar da alta taxa de hemostasia eficaz com antídotos. A falha em atingir hemostasia efetiva correlaciona-se fortemente com desfecho desfavorável e óbito. Uma metanálise reportou taxas de tromboembolismo particularmente elevadas com uso de andexanete.^170^ Portanto, novos estudos, especialmente focando em estratégias de implementação e sistematização do atendimento de pacientes com sangramentos graves, são necessários para entender melhor o uso e o real papel dos antídotos nos diferentes cenários clínicos.

**Figura 12:**
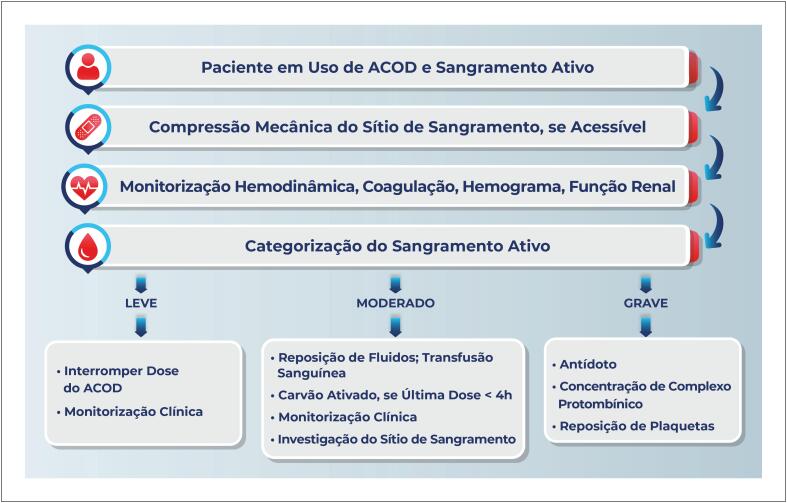
Algoritmo de abordagem do paciente com sangramento ativo em uso de anticoagulante oral de ação direta (ACOD).

A Figura 12 sumariza o manejo do sangramento em pacientes em uso de ACOD. As indicações para o uso de antídoto nos casos de sangramento secundário ao uso de ACOD estão apresentadas na Tabela 10. O resumo dos principais estudos relacionados ao uso de antídotos em sangramentos secundários ao uso ACOD está descrito na Tabela 11.

**Tabela 10:** Indicações para o uso de antídotos em pacientes em uso de anticoagulantes de ação direta (ACOD)

Sangramento com risco de morte: sistema nervoso central, hemorragia incontrolável
Sangramento em cavidade ou órgão críticos: intraespinal, intraocular, pericárdico, retroperitoneal, intramuscular com síndrome compartimental
Sangramento maior apesar de medidas de hemostasia local ou risco de sangramento recorrente
Cirurgia ou intervenção de emergência em pacientes com alto risco de sangramento: neurocirurgia, punção lombar, cirurgia cardíaca, vascular e hepática

**Tabela 11:** Antídotos utilizados para os anticoagulantes de ação direta (ACOD)

Antídoto	Alvo	Descrição	Estudo principal	Critério de inclusão	Resultados principais
**Idarucizumabe**	Dabigatrana	Fragmento de anticorpo monoclonal	RE-VERSE AD^165^	Hemorragia incontrolável (GI ou SNC) Cirurgia urgente	Sangramento cessou em média 2,5 h Reversão completa da dabigatrana em 97,5% dos pacientes
**Andexanete**	Inibidor direto do fator Xa	Molécula recombinante derivada do fator Xa humano	ANNEXA-4^171^	Sangramento maior (trato GI e SNC, 18 h após ACOD)	Hemostasia efetiva em 12 h em 82% dos pacientes Redução de 92% da atividade da apixabana e rivaroxabana
**Ciraparantag** *	Inibidor direto do fator Xa	Molécula sintética, reverte inibidores diretos orais do fator Xa	Ansell et al.^167^ Medicamento em investigação		Reversão total da ação anticoagulante em até 30 min

* Não provado para uso na prática clínica. GI: gastrointestinal; SNC: sistema nervoso central.

O uso de concentrados de complexo protrombínico de quatro fatores pode ser considerado como uma alternativa de tratamento para reverter o efeito anticoagulante da rivaroxabana, apixabana e edoxabana, embora as evidências científicas sejam muito limitadas nesse contexto. As recomendações para o manejo de sangramento ativo em pacientes em uso de ACOD estão representadas no Quadro 6.

**Quadro 6:** Recomendações para o manejo de sangramento ativo em pacientes em uso de anticoagulantes de ação direta (ACOD)

Recomendações para o manejo de sangramento ativo em pacientes em uso de ACOD	Classe de recomendação	Nível de evidência
Interromper o uso do ACO até identificação e tratamento do sangramento leve, moderado ou grave	**I**	**C**
Realizar intervenções diagnósticas para identificação do sítio de sangramento em pacientes sem causa identificada	**I**	**C**
Uso de idarucizumab em pacientes com sangramento grave e risco de morte secundário ao uso de dabigatrana	**IIa**	**B**
Uso de andexanet em pacientes com sangramento grave e risco de morte secundário ao uso de anticoagulanetes inibidores fator Xa	**IIa**	**B**
Uso de carvão ativado e lavagem gástrica nas primeiras 4 h após o uso do ACOD em paciente com sangramento moderado ou grave	**IIa**	**C**
Concentrado de complexo protrombínico na vigência de sangramento moderado ou grave sem resposta ao antídoto	**IIa**	**C**
Uso de plasma fresco congelado ou concetrado de plaquetas em pacientes com sangramento moderado sem disponibilidade de outras terapias	**IIa**	**C**
Uso de carvão ativado em casos de hemorragia digestiva	**III**	**C**

### 6.5. Uso dos Anticoagulantes em Situações Especiais

#### 6.5.1. Cardioversão Elétrica

Pacientes submetidos à cardioversão elétrica devem ser avaliados quanto ao risco de eventos TE. A duração da arritmia (< 24 horas ou ≥ 24 horas) tem sido utilizada para determinar o momento da cardioversão. Quando inferior a 24 horas, deve ser precedida por anticoagulação com ACOD (dabigatrana, rivaroxabana, apixabana ou edoxabana), heparina subcutânea (enoxaparina 1 mg/kg) ou heparina endovenosa em *bolus* (60-70 U/kg).^17^ Nos casos em que o início é superior a 24 horas ou desconhecido, o ecocardiograma transesofágico pode ser realizado para detecção de trombo. Na ausência de trombo, a cardioversão pode ser realizada após anticoagulação com heparina (endovenosa em *bolus* ou subcutânea) ou ACOD.^17^ Se a realização do ecocardiograma transesofágico não for possível ou houver presença de trombo, deve-se iniciar anticoagulação oral com ACOD ou varfarina (alvo de INR 2,0-3,0) por 3 semanas antes da cardioversão.^17^ Vale lembrar que as 3 semanas de varfarina são contadas a partir da primeira INR em nível terapêutico. Já com os ACOD, é imprescindível assegurar o uso correto da medicação. Se houver dúvidas quanto à adesão ao tratamento, deve-se considerar a realização do ecocardiograma transesofágico.^172^

Inicialmente, o uso dos ACOD no cenário da cardioversão tinha seu uso respaldado nas análises de subgrupos dos grandes estudos fase 3: RE-LY (*Randomized Evaluation of Long Term Anticoagulant Therapy*); ENGAGE AF-TIMI (*Effective Anticoagulation With Factor Xa Next Generation*); ARISTOTLE (*Apixaban for Reduction in Stroke and Other Thromboembolic Events in Atrial Fibrillation*); e ROCKET-AF (*An Efficacy and Safety Study of Rivaroxaban With Warfarin for the Prevention of Stroke and Non-Central Nervous System Systemic Embolism in Patients With Non-Valvular Atrial Fibrillation*).^152,173-175^ Posteriormente, estudos direcionados para a avaliação de eficácia e segurança dos ACOD na cardioversão de FA foram conduzidos, fornecendo evidências de eficácia e segurança similar aos AVK (Tabela 12).^176-178^ Além disso, algumas análises inclusive demonstram vantagem com o uso dos ACOD.^179^ As recomendações para os usos de anticoagulantes na cardioversão elétrica são apresentadas no Quadro 7.

**Tabela 12:** Estudos clínicos com o uso dos ACOD para cardioversão de FA

	X-VeRT	ENSURE-AF	EMANATE
**Anticoagulante**	Rivaroxabana	Edoxabana	Apixabana
**Número de pacientes**	1.504	2.199	1.500
**Desenho do estudo**	Randomizado	Randomizado	Randomizado
**Duração de FA**	≥ 48 h	≥ 48 h	< 48 h e ≥ 48 h
**Comparador**	AVK	AVK/heparina de baixo peso molecular	AVK/heparina
**Estratégia**	ETE + rivaroxabana por pelo menos 4 horas antes da CVE	ETE + edoxabana por pelo menos 2 horas antes da CVE	Apixabana por pelo menos 2 horas antes da CVE ou após 5 doses
**Anticoagulação após CVE**	42 dias	28 dias	30 dias
**Desfecho eficácia**	Composto por AVC, AIT, embolia sistêmica, infarto ou morte cardiovascular	Composto por AVC, AIT, embolia sistêmica, infarto ou morte cardiovascular	AVC, embolia sistêmica, mortalidade
**Resultado ACOD & AVK**	5 (0,5%) vs. 5 (1,0%)	5 (0,5%) vs. 11 (1,0%)	0 (0%) vs. 6 (0,8%)
**Desfecho de segurança**	Sangramento maior	Sangramento maior ou clinicamente relevante	Sangramento maior ou clinicamente relevante
**Resultado ACOD & AVK**	6 (0,6%) vs. 4 (0,8%)	16 (1,5%) vs. 11 (1,0%)	14 (1,9%) vs. 19 (2,5%)

Adaptado de Lucà et al.^180^ FA: fibrilação atrial; AVK: antagonistas da vitamina K; ETE: ecocardiograma transesofágico; CVE: cirurgia vascular endovascular; AVC: acidente vascular cerebral; AIT: acidente isquêmico transitório; ACOD: anticoagulantes orais de ação direta.

**Quadro 7:** Recomendações para uso de anticoagulantes na cardioversão elétrica

Recomendações para prevenção de eventos tromboembólicos na cardioversão da fibrilação atrial (FA)	Classe de recomendação	Nível de evidência
A cardioversão elétrica em paciente com FA com duração superior a 24 horas deve ser realizada após anticoagulação por, no mínimo 3 semanas, com AVK ou ADOC e mantida por, pelo menos, 4 semanas	**I**	**B**
O uso do eco transesofágico é recomendado como alternativa à anticoagulação oral prévia de 3 semanas	**I**	**B**
O uso do anticoagulante é recomendado por, no mínimo, 4 semanas após a cardioversão em todos os pacientes independente do escore CHA_2_DS_2_-VA. O uso indefinidamente deve ser baseado no perfil de risco do paciente	**I**	**C**
A importância da adesão aos anticoagulantes deve ser enfatizada para todos os pacientes	**I**	**C**
A terapia com antiagregante plaquetário isolada ou em combinação não é indicada para cardioversão de FA	**III**	**B**

Além da duração da FA, o risco embólico deve ser considerado na programação da cardioversão. Pacientes com FA < 12 horas e CHA_2_DS_2_-VA = 0 em homens e 1 em mulheres apresentam baixo risco. Já pacientes com FA de etiologia valvar (estenose mitral grave ou prótese valvar mecânica) ou evento embólico prévio apresentam risco elevado, devendo sempre ser tratados com a mesma estratégia para duração superior a 24 horas.^179^ Essa abordagem também se aplica aos pacientes com escore maior ou igual a 2 em homens e maior ou igual a 3 em mulheres cuja FA tenha duração entre 12 e 24 horas.^179^

**Figura 13:**
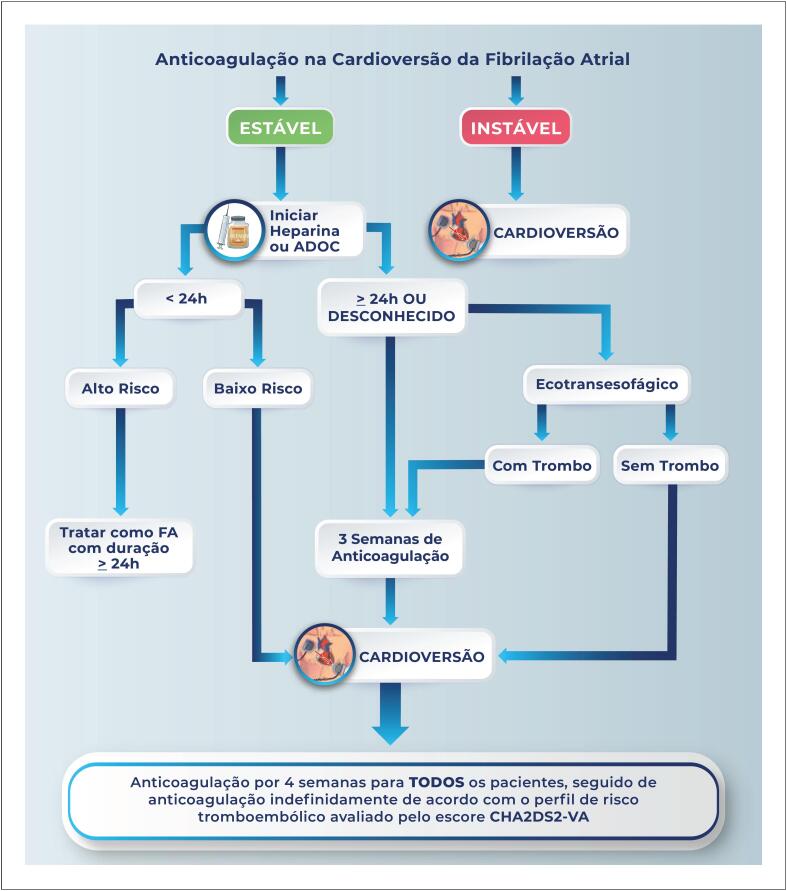
Manejo de pacientes com indicação de cardioversão na FA. ACOD: anticoagulantes orais de ação direta.

A anticoagulação oral deve ser continuada por, pelo menos, 4 semanas após a cardioversão em todos os pacientes independentemente da sua duração e perfil de risco.^179^ Após esse período, a manutenção da anticoagulação oral depende do risco tromboembólico: CHA_2_DS_2_-VA ≥ 2 devem ser anticoagulados indefinidamente. Em pacientes com escore = 0, a anticoagulação pode ser interrompida. Nos pacientes com risco intermediário (CHA_2_DS_2_-VA = 1), a decisão deve ser individualizada. A aspirina nesse contexto é contraindicada. O algoritmo para tomada de decisão em pacientes submetidos à cardioversão de FA estável é ilustrado na Figura 13. As recomendações para prevenção de eventos TE na cardioversão da FA estão no Quadro 7. A FA que cursa com instabilidade hemodinâmica deve ser submetida a cardioversão elétrica com introdução do anticoagulante o mais rápido possível.

Pacientes com trombo detectado no ecocardiograma transesofágico devem ser anticoagulados por, pelo menos, 3 semanas antes da cardioversão. De maneira geral, não se recomenda repetir o ecocardiograma ao final das 3 semanas de anticoagulação, assumindo que esta tenha sido eficaz e, portanto, novos trombos não tenham sido formados.

#### 6.5.2. Fibrilação Atrial Valvar

As doenças cardíacas valvares representam fator de risco independente para a ocorrência de FA. Estima-se que mais de um terço dos pacientes com FA apresente alguma forma de doença valvar. Pacientes com doença valvar e FA apresentam maior risco de eventos embólicos quando comparados àqueles com FA e sem doença valvar.^181^

Na presença de doença valvar, a decisão pelo tipo de anticoagulante deve levar em consideração a etiologia, a valva acometida e o grau de comprometimento, além da presença e do tipo de prótese valvar. Assim, torna-se necessária a individualização na escolha do ACO em pacientes com FA e doença cardíaca valvar.

##### 6.5.2.1. Anticoagulação Oral em Pacientes com Doenças Cardíacas Valvares em Válvulas Nativas

Apesar de os grandes estudos pivotais em anticoagulação excluírem portadores de estenose mitral moderada a grave e prótese mecânica, muitos pacientes com doença cardíaca valvar estavam contemplados. Dos 71.531 pacientes avaliados, 13.585 (19%) apresentavam algum tipo de valvopatia. A regurgitação mitral foi a mais prevalente, acometendo 10.633 pacientes (14,9% do total). As patologias da valva aórtica ocorreram em 3.794 pacientes, sendo 2.559 (3,6% do total) com regurgitação aórtica e 1.235 (1,7% do total) com estenose aórtica. A regurgitação tricúspide ocorreu em 4,6% dos pacientes, aproximadamente 1% dos pacientes apresentavam cirurgia/reparo valvar prévios e 0,5% tinham estenose mitral leve.^182-185^

Pacientes com doença valvar eram mais velhos e apresentavam maior número de comorbidades, além de taxas mais elevadas de AVC, embolia sistêmica e sangramentos maiores. Ainda assim, os ACOD demonstraram eficácia e segurança comparáveis à varfarina, não havendo, de maneira geral, diferença entre os grupos. A única exceção foi o maior risco de sangramento nos pacientes em uso de rivaroxabana.^182,185-187^ Assim, os ACOD constituem os fármacos de escolha em pacientes com FA e doenças valvares que incluem a regurgitação mitral, regurgitação aórtica e estenose aórtica, principalmente quando a etiologia é degenerativa.

##### 6.5.2.2. Anticoagulação Oral em Pacientes com Estenose Mitral Moderada a Grave

Recentemente publicado, o INVICTUS (*INVestIgation of rheumatiC AF Treatment Using Vitamin K Antagonists, Rivaroxaban or Aspirin Studies*) foi o primeiro estudo randomizado que comparou a eficácia e a segurança da rivaroxabana frente aos AVK em 4.531 pacientes com FA e estenose mitral moderada a grave de etiologia reumática. Desfechos primários (composto de AVC, embolia sistêmica, infarto do miocárdio, morte por causa vascular ou desconhecida) ocorreram mais comumente no grupo rivaroxabana (8,21%/ano vs. 6,5%/ano). Quando os componentes foram analisados de maneira isolada, observaram-se maiores taxas de morte bem como de AVCi no grupo rivaroxabana. Em relação às taxas de sangramento, não houve diferença entre os grupos.^188^ Em andamento, o estudo DAVID-MS (*DAbigatran for Stroke PreVention In Atrial Fibrillation in MoDerate or Severe Mitral Stenosis*) terá como objetivo avaliar os efeitos da dabigratana em pacientes com estenose mitral moderada ou grave comparada à varfarina.^189^ Diante das atuais evidências, pacientes com FA e estenose mitral moderada a grave de etiologia reumática elegíveis para anticoagulação devem ser anticoagulados com AVK.

##### 6.5.2.3. Anticoagulação Oral em Pacientes com Próteses Valvares

Desenvolvido no Brasil, o RIVER (*RIvaroxaban for Valvular Heart diseasE and atRial Fibrillation Trial*) foi um estudo randomizado que analisou a eficácia e a segurança da rivaroxabana em 1.005 pacientes com FA e prótese biológica em posição mitral; a rivaroxabana foi não inferior à varfarina quanto ao desfecho primário de morte, eventos cardiovasculares maiores e sangramento maior. O estudo demonstrou ainda uma redução numérica nas taxas de morte cardiovascular e de sangramento maior, além da redução nas taxas de AVC no grupo tratado com rivaroxabana. Além disso, paciente incluídos nos 3 primeiros meses após a troca valvar (N = 189 pacientes) no grupo rivaroxabana apresentaram redução significativa no desfecho primário do estudo (6,4% contra 18,9%).^190^ Assim, os ACOD representam alternativa segura e eficaz para anticoagulação de pacientes com FA submetidos a troca valvar mitral por bioprótese.

Em relação aos efeitos dos ACOD em pacientes submetidos à implantação transcateter da válvula aórtica (TAVI, de *transcatheter aortic valve implantation*), resultados de estudos observacionais e registros iniciais foram discordantes ao comparar os ACOD à varfarina.^191,192^ Recentemente, os resultados de dois estudos clínicos randomizados foram publicados. No ATLANTIS (*Anti-Thrombotic Strategy to Lower All Cardiovascular and Neurologic Ischemic and Hemorrhagic Events after Trans-Aortic Valve Implantation for Aortic Stenosis*), 1.451 pacientes submetidos a TAVI foram divididos em dois grupos com e sem FA. No grupo com FA, a ocorrência do desfecho primário (morte, AVC, infarto agudo do miocárdio [IAM], embolia sistêmica e trombose valvar) foi semelhante entre os grupos que receberam apixabana ou varfarina. O mesmo ocorreu com as taxas de sangramento. No grupo sem FA, no qual se comparou apixabana à terapia padrão (92% com terapia antiplaquetária isolada), a apixabana aumentou o risco do desfecho primário, embora tenha reduzido o risco de trombose valvar clínica ou subclínica.^193^

O ENVISAGE-TAVI AF (*Edoxaban Compared to Standard Care After Heart Valve Replacement Using a Catheter in Patients with Atrial Fibrillation*) comparou edoxabana com AVK em 1.426 pacientes com FA submetidos a TAVI. Pacientes randomizados para o tratamento com edoxabana apresentaram resultados semelhantes àqueles tratados com varfarina em relação ao desfecho composto primário de morte, AVC, embolia sistêmica, IAM, trombose da válvula ou sangramento importante. Contudo, maiores taxas de hemorragia gastrointestinal ocorreram nos pacientes em uso de 60 mg de edoxabana e naqueles em uso concomitante de antiplaquetários.^194^

A segurança da associação entre anticoagulantes e antiplaquetários, em pacientes com FA submetidos a TAVI, foi avaliada no POPular TAVI *Trial*. Esse estudo incluiu 313 pacientes (mais de 94% apresentavam FA e aproximadamente 30% utilizavam ACOD). A anticoagulação isolada resultou em menos eventos hemorrágicos, sem o aumento concomitante de eventos isquêmicos, sugerindo que a terapia anticoagulante isolada possa ser suficiente para o tratamento desses pacientes.^195^

Baseado nesses dados, os ACOD representam uma opção terapêutica eficaz e segura para pacientes com FA submetidos a TAVI e elegíveis para tratamento anticoagulante. A associação com antiplaquetário deve ser individualizada e, em geral, reservada para pacientes que tenham sido submetidos à intervenção coronária percutânea recente (< 3 meses).

**Figura 14:**
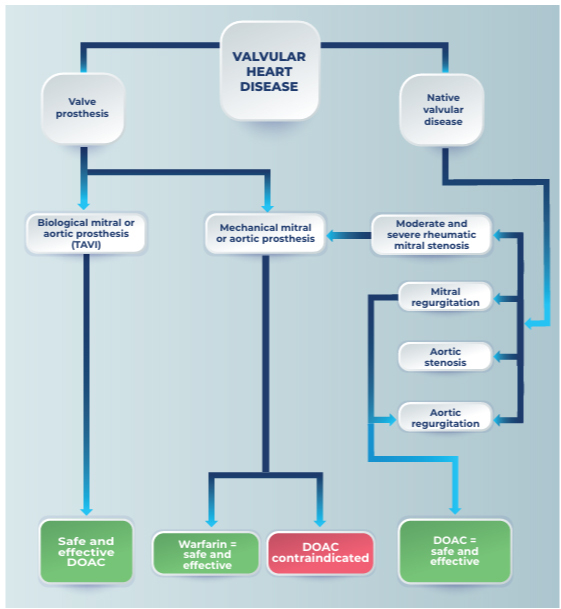
Fluxograma para anticoagulação oral em pacientes com doenças cardíacas valvares e fibrilação atrial. ACOD: anticoagulantes orais de ação direta; TAVI: implantação transcateter da válvula aórtica.

A anticoagulação com ACOD em pacientes com FA e próteses valvares mecânicas foi avaliada no estudo RE-ALIGN (*Dabigatran Etexilate in Patients With Mechanical Heart Valves*), que comparou a dabigatrana à varfarina. O estudo planejava a inclusão de 405 pacientes portadores de próteses valvares mecânicas nas posições aórtica ou mitral. Entretanto, foi interrompido precocemente, após a inclusão de 252 pacientes, dadas as maiores taxas de AVC e sangramento maior no grupo dabigatrana.^196^ Da mesma forma, o estudo PROACT-Xa (*A Trial to Determine if Participants with an On-X Aortic Valve Can be Maintained Safely on Apixaban*) foi desenhado com o objetivo de incluir 1.000 pacientes submetidos ao implante de prótese mecânica em posição aórtica (válvula On-X), pelo menos 3 meses antes da randomização, e avaliar a não inferioridade da apixabana comparada à varfarina quanto à incidência do desfecho primário composto de trombose valvar ou evento TE. O estudo foi interrompido precocemente, após a inclusão de 863 participantes, dada a maior ocorrência de eventos TE no grupo apixabana. Não houve diferença significativa entre os grupos em relação às taxas de sangramento.^197^ Diante disso, estabelece-se que pacientes portadores de próteses mecânicas devem ser anticoagulados com a varfarina. A Figura 14 representa um fluxograma para anticoagulação oral em pacientes com doença cardíaca valvar e FA.

As recomendações para anticoagulação de pacientes com doença cardíaca valvar e FA elegíveis para anticoagulação estão representadas no Quadro 8.

**Quadro 8:** Recomendações para anticoagulação de pacientes com doença cardíaca valvar e fibrilação atrial elegíveis para anticoagulação

Recomendações para anticoagulação de pacientes com doença cardíaca valvar e fibrilação atrial elegíveis para anticoagulação	Classe de recomendação	Nível de evidência
Em pacientes com próteses valvares mecânicas, a varfarina é o fármaco de escolha.	**I**	**A**
Em pacientes com estenose aórtica, regurgitação aórtica e regurgitação mitral, os ACOD são recomendados preferencialmente à varfarina.	**I**	**A**
Em pacientes com estenose mitral moderada e grave, a varfarina é o fármaco de escolha.	**I**	**B**
Os ACOD representam alternativa segura e eficaz para anticoagulação de pacientes com FA submetidos a troca valvar mitral por bioprótese.	**IIa**	**B**
Os ACOD representam alternativa segura e eficaz para anticoagulação de pacientes com FA submetidos a bioprótese aórtica (TAVI).	**IIa**	**B**
Os ACOD são contraindicados em pacientes com estenose mitral moderada a grave.	**III**	**A**
Os ACOD são contraindicados em pacientes com próteses valvares mecânicas.	**III**	**A**

ACOD: anticoagulantes orais de ação direta; TAVI: implantação transcateter da válvula aórtica.

#### 6.5.3. Anticoagulação na Síndrome Coronariana Aguda

Em pacientes com síndrome coronariana aguda (SCA) e FA em uso de anticoagulação oral, é necessária adequada consideração entre o tempo de intervenção e o risco de sangramento. O uso do ACOD é uma contraindicação relativa à trombólise, devendo esses pacientes ser triados preferencialmente à estratégia de angioplastia primária.^198^ A via de acesso radial deve ser priorizada dado o menor risco de sangramento, assim como deve-se evitar o uso de inibidores da P2Y12 mais potentes, como prasugrel e ticagrelor.^198,199^

Pacientes com SCA com supradesnível do segmento ST, assim como SCA sem supradesnível do segmento ST de alto risco, devem receber dose adicional de anticoagulante parenteral (por exemplo, heparina não fracionada 60 UI/kg ou enoxaparina 0,5 mg/kg), independentemente da última dose do ACO.^200^ Em casos de SCA sem supradesnível do segmento ST e sem critérios para intervenção coronária percutânea de emergência, recomenda-se a suspensão do ACOD (até RNI < 2 se uso de varfarina, por 24 h se ACOD e TFG > 30 mL/min e por 36 h se ACOD e TFG < 30 mL/min) associada à anticoagulação parenteral até o momento do procedimento.^201^ As recomendações para o uso de anticoagulantes na fase aguda da SCA estão no Quadro 9.

**Quadro 9:** Recomendações para uso de anticoagulantes na fase aguda da síndrome coronariana aguda (SCA)

Recomendações para o uso de anticoagulantes na fase aguda da SCA	Classe de recomendação	Nível de evidência
Pacientes anticoagulados devem ser encaminhados preferencialmente à estratégia de angioplastia primária	**IIa**	**C**
Pacientes com SCA com supradesnivelamento do segmento ST e anticoagulação oral devem receber dose usual de anticoagulação parenteral	**IIa**	**C**
Pacientes com SCA sem supradesnivelamento do segmento ST e sem critérios de alto risco devem ter seu anticoagulante oral suspenso e transição realizada para anticoagulante parenteral	**IIa**	**C**

O uso do anticoagulante após intervenção coronária percutânea também foi motivo de estudos clínicos, uma vez que a combinação de FA e DAC crônica não é um cenário incomum. Estima-se que cerca de 10 a 15% dos pacientes portadores de FA são submetidos a angioplastia em algum momento da vida. Sabe-se que a terapia anticoagulante por si só é insuficiente na prevenção de trombose por *stent*, assim como o uso de antiplaquetários isoladamente não previne eventos trombóticos na FA. Logo, a combinação entre as duas classes de medicamentos impõe um dilema: proteção contra eventos cardiovasculares e aumento demasiado no risco de sangramento.^202^ Nesse cenário, cinco ensaios clínicos randomizados (quatro com o uso de ACOD e um com o uso de varfarina) avaliaram o uso de tripla terapia com ácido acetilsalicílico (AAS), inibidor da P2Y12 (majoritariamente clopidogrel), anticoagulante contra dupla terapia com inibidor da P2Y12 (majoritariamente clopidogrel) e anticoagulante em pacientes portadores de FA com SCA recente ou pós-angioplastia eletiva (Tabela 13).^203-207^

**Tabela 13:** Resumo dos principais estudos clínicos que avaliaram a terapia antitrombótica com ACOD em pacientes com FA e doença arterial coronariana

Estudo	Ano de publicação	N	Tratamento	Desfecho primário de segurança	Tempo até descalonamento (dias)	Seguimento (meses)	Conclusão
**PIONEER AF-PCI**	2016	1.389	Terapia dupla (clopidogrel + rivaroxabana) vs. 8 meses terapia tripla (AAS + clopidogrel + varfarina)	Sangramento TIMI maior ou menor ou sangramento necessitando atendimento médico	3	12	Menores taxas de sangramento clinicamente relevante com terapia dupla.
**RE-DUAL PCI**	2017	2.725	Terapia dupla (clopidogrel + dabigatrana 110 mg ou 150 mg 2x/d) vs. 2,7 meses terapia tripla (AAS + clopidogrel + varfarina)	Sangramento maior ISTH ou sangramento clinicamente relevante não maior	5	14	Menores taxas de sangramento com terapia dupla. Dupla terapia foi não inferior à tripla terapia em eventos trombóticos.
**AUGUSTUS**	2019	4.614	Terapia dupla (clopidogrel + apixabana 5 mg 2x/d/ varfarina) vs. 6 meses terapia tripla (AAS + clopidogrel + apixabana / varfarina)*	Sangramento maior ISTH ou sangramento clinicamente relevante não maior	6	6	Terapia dupla com clopidogrel e apixabana, sem aspirina, resultou em menos sangramento sem aumento na taxa de eventos isquêmicos com varfarina, aspirina ou ambos.
**ENTRUST-AF PCI**	2019	1.506	Terapia dupla (clopidogrel + edoxabana 60 mg/d) vs. 3 meses terapia tripla (AAS + clopidogrel + varfarina)**	Sangramento maior ISTH ou sangramento clinicamente relevante não maior	2	12	Dupla terapia foi não inferior à tripla terapia com varfarina, sem um aumento significativo no número de eventos isquêmicos.

* Apixabana 2,5 mg duas vezes ao dia se ao menos dois dos três fatores: > 80 anos, peso < 60 kg ou creatinina > 1,5 mg/dL.

** Edoxabana 30 mg/d se algum dos seguintes presente: clearance de creatinina 15-50 mL/min, peso < 60 kg ou uso concomitante de inibidores da glicoproteína-P. ACOD: anticoagulantes orais de ação direta; FA: fibrilação atrial; AAS: ácido acetilsalicílico; TIMI: trombólise em infarto do miocárdio; ISTH: International Society for Thrombosis and Haemostasis.

A despeito de algum grau de heterogeneidade, todos de maneira geral demonstraram redução nas taxas de sangramento com a dupla terapia sem aumento de eventos isquêmicos. Tais estudos, entretanto, não possuíam poder suficiente para avaliação de desfechos isquêmicos. Metanálises e subanálises subsequentes demonstraram redução de sangramento com dupla terapia, apesar de uma tendência de aumento do risco de eventos isquêmicos, principalmente nos primeiros 30 dias.^208-211^

No estudo AUGUSTUS (*Open-Label, 2×2 Factorial, Randomized, Controlled Clinical Trial to Evaluate the Safety of Apixaban vs. Vitamin K Antagonist and Aspirin vs. Placebo in Patients With Atrial Fibrillation and Acute Coronary Syndrome and/or Percutaneous Coronary Intervention*), o tratamento com ACOD na terapia dupla (sem AAS) apresentou melhor perfil de segurança, com redução de até 78% nas taxas de sangramento relevante comparado com estratégia tripla (AAS mais clopidogrel mais varfarina). Essa estratégia dupla com apixabana mais clopidogrel por 6 meses representou um número necessário para tratar (NNT) de 9 para evitar sangramento relevante. Além disso, a terapia com apixabana reduziu o risco de morte e hospitalização.^209^ Finalmente, o estudo ENTRUST (*Edoxaban Treatment Versus Vitamin K Antagonist in Patients With Atrial Fibrillation Undergoing Percutaneous Coronary Intervention*) mostrou não inferioridade da terapia dupla com edoxabana (60 mg 1 x/dia) + um inibidor de P2Y12 comparado com a terapia tripla clássica (AVK + dupla antiagregação plaquetária) quanto ao desfecho composto de sangramento maior ou clinicamente relevante não maior em 12 meses e sem diferenças em desfechos trombóticos.^207^

Recomenda-se a tripla terapia com a escolha do clopidogrel como inibidor da P2Y12 e preferencialmente com o uso de ACOD nos primeiros 7 dias, seguida por dupla terapia antiagregante com clopidogrel por 1 ano em pacientes com SCA prévia e por 6 meses pós-angioplastia eletiva. Após esse período, o anticoagulante deve ser mantido isoladamente.

A tripla terapia, com o uso de apixabana associada a aspirina e clopidogrel, pode ser estendida por até 1 mês, de maneira individualizada, em pacientes de alto risco de trombose de *stent,* tais como lesão em tronco ou bifurcações, malha de *stent* > 60 mm, hipoexpansão do *stent*, intervenção em oclusão crônica, trombose prévia de *stent* em adequado tratamento antiplaquetário, angioplastia em contexto de SCA, diabetes descompensado e doença renal crônica. Tal decisão deve ser sempre acompanhada de uma avaliação quanto ao maior risco de sangramento com essa estratégia.

**Figura 15:**
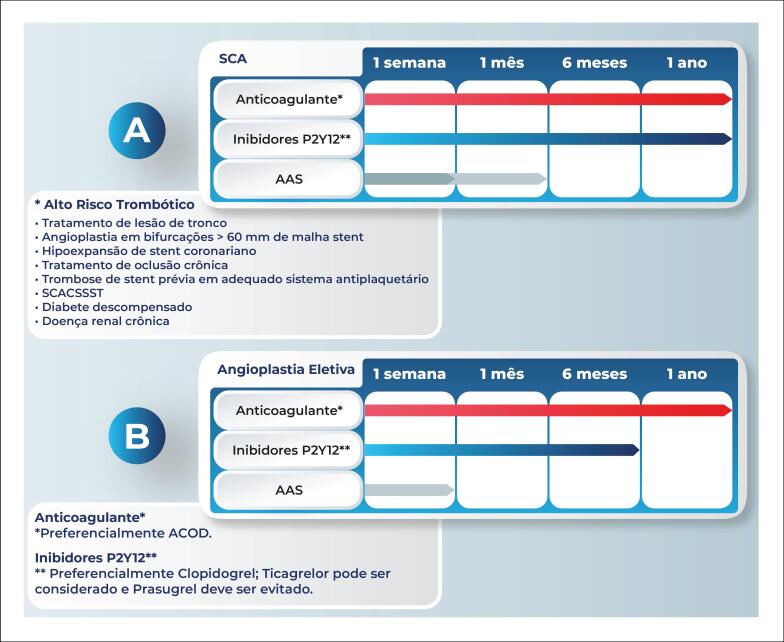
Representação esquemática do tempo de uso de dupla e tripla terapia antitrombótica no paciente com doença arterial coronariana e fibrilação atrial nos diferentes cenários clínicos. (A) pacientes com síndrome coronária aguda ou com alto risco trombótico; (B) pacientes submetidos a intervenção percutânea eletiva. AAS: ácido acetilsalicílico; SCACSSST: síndrome coronariana aguda sem supra do segmento ST; SCA: síndrome coronariana aguda.

O Quadro 10 sumariza as recomendações quanto ao uso de anticoagulantes e antiplaquetários em pacientes com DAC. A Figura 15 demonstra o uso da terapia antitrombótica nos diferentes cenários da DAC.

**Quadro 10:** Recomendações quanto ao uso de anticoagulantes e antiplaquetários em pacientes com DAC após intervenção coronária percutânea

Recomendações quanto ao uso de anticoagulantes e antiplaquetários em pacientes com DAC após intervenção coronária percutânea	Classe	Nível de evidência
Pacientes com necessidade de anticoagulação por FA submetidos à angioplastia coronária devem fazer uso de tripla terapia por, pelo menos, 1 semana, seguido de dupla terapia	**I**	**A**
Pacientes com necessidade de anticoagulação por FA internados por SCA em estratégia de tratamento clínico devem fazer uso de tripla terapia por, pelo menos, 1 semana, seguido de dupla terapia	**I**	**A**
Dupla terapia com antiplaquetário e anticoagulante oral deve ser mantida por 6 meses em pacientes pós-angioplastia eletiva e por 1 ano após SCA	**I**	**A**
Tripla terapia pode ser estendida por até 1 mês em casos individualizados	**IIa**	**C**
Deve-se priorizar o uso de clopidogrel como antiplaquetário em associação aos anticoagulantes orais	**IIa**	**A**
O uso de ACOD é superior à varfarina quando associado a antiplaquetários pós-intervenção coronária	**I**	**A**
O controle dos fatores de risco modificáveis para sangramento deve ser realizado.	**I**	**B**

DAC: doença arterial coronariana; FA: fibrilação atrial; SCA: síndrome coronariana aguda; ACOD: anticoagulantes orais de ação direta.

Recomenda-se o uso de inibidores da bomba de prótons em todos os pacientes submetidos à dupla terapia antitrombótica, salvo contraindicações.^212,213^ Da mesma maneira, é fortemente recomendado um posicionamento ativo por parte do médico em relação aos fatores de risco de sangramento modificáveis, com um adequado controle pressórico, incentivo à diminuição do consumo de álcool e otimização da função renal.^214^ As recomendações quanto à anticoagulação associada ao uso de antiagregante plaquetário estão representadas no Quadro 10.

#### 6.5.4. Manejo da Anticoagulação no Perioperatório de Ablação e Cirurgia Cardíaca

Embora exista alguma variação no manejo da terapia anticoagulante em pacientes submetidos à ablação percutânea da FA, há uma tendência para a estratégia de realização do procedimento sem a interrupção da anticoagulação oral, tanto da varfarina (desde que a INR esteja dentro da faixa terapêutica) quanto dos ACOD. Em pacientes que não estejam sob anticoagulação por algum motivo, sugere-se o início da terapia oral por 3 a 4 semanas previamente ao procedimento.^215^ Em uma metanálise com 12 estudos, a anticoagulação ininterrupta utilizando ACOD para ablação por cateter de FA associou-se a menores taxas de AVC e acidente isquêmico transitório (AIT) (0,08% *versus* 0,16%) e taxas similares de eventos embólicos sistêmicos silenciosos (8,0% *versus* 9,6%) quando comparadas com AVK. As taxas de sangramentos maiores foram significantemente reduzidas com continuidade do ACOD (0,9%) comparativamente a AVK (2,0%).^216^

De modo geral, a não interrupção de ACOD ocasiona a menor incidência de AVC e AIT com significativa redução em sangramentos maiores em pacientes submetidos à ablação por cateter de FA. No RE-CIRCUIT (*Uninterrupted Dabigatran Etexilate in Comparison to Uninterrupted Warfarin in Pulmonary Vein Ablation*), o maior estudo randomizado periprocedimento que comparou ACOD *versus* AVK, a incidência de sangramento maior nas primeiras 8 semanas após ablação foi significativamente menor com dabigatrana comparada com AVK (1,6% *versus* 6,9%).^217^ Outros estudos randomizados, como o VENTURE-AF (*A Study Exploring Two Treatment Strategies in Patients With Atrial Fibrillation Who Undergo Catheter Ablation Therapy*) com rivaroxabana,^218^ AXAFA-AFNET 5 (*Anticoagulation using the direct factor Xa inhibitor apixaban during Atrial Fibrillation catheter Ablation: comparison to VKA therapy*) com apixabana^219^ e ELIMINATE-AF (*A Prospective, Randomized, Open-Label, Blinded Endpoint Evaluation [PROBE] Parallel Group Study Comparing Edoxaban vs. VKA in Subjects Undergoing Catheter Ablation of Non-valvular Atrial Fibrillation*) com edoxabana,^220^ também mostraram taxas de eventos similares com o uso de ACOD e AVK.

Por outro lado, a ponte com heparina aumenta a incidência de sangramentos e deve ser evitada. Na prática clínica, o termo anticoagulação ininterrupta usualmente se refere a descrição de regimes, nos quais até uma ou duas doses de ACOD podem ser omitidas pré-ablação. Entretanto, a administração de ACOD foi verdadeiramente ininterrupta nos estudos randomizados, e não há razão para a recomendação de omissão de doses previamente ao procedimento.

Após o procedimento de ablação, a primeira dose deve ser administrada no período da tarde ou na manhã seguinte, caso corresponda ao momento da próxima dose de acordo com o regime de anticoagulação previamente utilizado.^218^

Cirurgias cardíacas eletivas em pacientes sob uso de ACO são consideradas procedimentos de alto risco de sangramentos. Pacientes em uso de ACOD devem suspender o fármaco por pelo menos 48 horas antes do procedimento, porém suspensões por períodos mais prolongados, como 72-96 horas, podem ser consideradas em pacientes com risco de acúmulo do fármaco, como idosos e portadores de doença renal crônica.^221^ Já o AVK deve ser suspenso cerca de 5 dias antes do procedimento, e considera-se segura a realização da cirurgia cardíaca quando INR < 1,5.^222^ De forma geral, como na maioria das situações, a ponte com heparina não é recomendada de rotina para pacientes anticoagulados em condição de cirurgia eletiva.^221^ A Tabela 14 resume o tempo recomendado de suspensão do anticoagulante de acordo com a função renal.

**Tabela 14:** Tempo para interrupção de anticoagulantes orais de ação direta (ACOD) previamente à cirurgia cardíaca de acordo com a função renal

Valor de depuração de creatinina	Dabigatrana	Rivaroxabana Apixabana Edoxabana
ClCr > 80 mL/min	≥ 48 hs	≥ 48 hs
ClCr 50-79 mL/min	≥ 72 hs	≥ 48 hs
ClCr 30-49 mL/min	≥ 96 hs	≥ 48 hs
ClCr 15-29 mL/min		≥ 48 hs

ClCr: clearance de creatinina.

Após cirurgia cardíaca, o reinício da anticoagulação depende de fatores como sangramento e possibilidade de intervenções adicionais. Heparina em dose profilática é recomendada desde o período pós-operatório precoce por cerca de 12-24 horas pós-procedimento se hemostasia adequada, seguida de anticoagulação plena após cerca de 36 horas de pós-operatório assumindo a ausência de sangramentos ativos.^222^ No momento em que hemostasia adequada tenha sido atingida, os drenos tenham sido retirados e o paciente esteja clinicamente estável sem planejamento de intervenções invasivas, a transição de heparina para anticoagulação oral plena é recomendada. As recomendações para o uso de terapia antitrombótica em procedimentos de ablação e cirurgia cardíaca estão apresentadas no Quadro 11.

**Quadro 11:** Recomendações para uso de terapia antitrombótica em procedimentos de ablação e cirurgia cardíaca

Recomendações para uso de terapia antitrombótica em procedimentos de ablação e cirurgia cardíaca	Classe	Nível evidência
Em pacientes com FA e fatores de risco tromboembólicos que não estão em uso de anticoagulantes orais pré-ablação, recomenda-se o início da anticoagulação 3 semanas antes do procedimento	**I**	**C**
Alternativamente, ecocardiograma transesofágico ou angiotomografia cardíaca pode ser realizado para exclusão da presença de trombo intracavitário atrial	**IIa**	**C**
Em pacientes que serão submetidos a ablação de FA por cateter e estão em uso de AVK ou ACOD, recomenda-se não suspender a anticoagulação oral antes do procedimento	**I**	**A**
Após ablação por cateter, é recomendada anticoagulação oral com AVK ou ACOD continuamente por pelo menos 2 meses em todos os pacientes e indefinidamente no paciente de alto risco	**I**	**C**
Pacientes em uso de ACOD devem suspender o fármaco por, pelo menos, 48 horas antes de cirurgia cardíaca	**I**	**C**
Pacientes com alto risco de sangramento, como idosos e portadores de insuficiência renal, podem suspender o uso do anticoagulante 72 horas antes da cirurgia cardíaca	**IIa**	**C**

FA: fibrilação atrial; AVK: anticoagulantes antagonistas da vitamina K; ACOD: anticoagulantes orais de ação direta.

#### 6.5.5. Anticoagulação na Fibrilação Atrial Subclínica

A FA subclínica é caracterizada por episódios assintomáticos de curta duração, geralmente inferior a 24 horas, normalmente detectados por dispositivos cardíacos implantáveis.^223^ Essa forma de FA pode estar presente em até um terço dos pacientes com dispositivos implantáveis como marca-passos e cardiodesfibrilador implantável (CDI). O risco de AVC nessa população parece ser mais baixo do que nos pacientes com FA clínica.^223-225^ Portanto, o benefício da anticoagulação oral nessa população ainda não está totalmente estabelecido. Recentemente, o estudo NOAH-AFNET 6 (*Non-vitamin K antagonist Oral anticoagulants in patients with Atrial High rate episodes*) randomizou 2.536 pacientes com episódios ritmo atrial acelerado detectados em dispositivos implantáveis e risco aumentado de AVC para o uso de edoxabana ou placebo. Vale lembrar que 56% dos pacientes receberam aspirina em vez de placebo.^224^ O estudo demonstrou ausência de benefício no desfecho composto de eficácia de AVC, embolia sistêmica ou morte cardiovascular associado ao aumento de sangramento maior em pacientes randomizados para o grupo edoxabana. É importante ressaltar que esse estudo foi interrompido precocemente, e, portanto, não tem poder estatístico adequado para detectar um efeito protetor da anticoagulação oral caso existisse, em especial para o AVC, o desfecho mais temido da FA.

O estudo ARTESiA (*Apixaban for the Reduction of Thrombo-Embolism in Patients With Device-Detected Sub-Clinical Atrial Fibrillation*), que randomizou mais de 4.000 pacientes com FA subclínica e risco aumentado para AVC, claramente demonstrou uma redução de RR de AVC e embolia sistêmica em 37% em pacientes randomizados para apixabana quando comparado com os pacientes que foram randomizados para aspirina.^226^ Importantemente, o risco de AVCs fatais e mais graves, de acordo com a escala de Rankin modificada, foi reduzido em 49% no grupo apixabana. Dos AVCs que ocorreram no grupo aspirina, 45% foram graves ou fatais. Conforme esperado, houve aumento de sangramento maior no grupo apixabana, mas a grande maioria (85%) foi tratada clinicamente e sem necessidade de medidas mais agressivas para seu controle. Vale ressaltar que não houve diferença entre apixabana e aspirina nas taxas de sangramentos fatais, intracranianos ou que levaram à instabilidade hemodinâmica.

**Figura 16:**
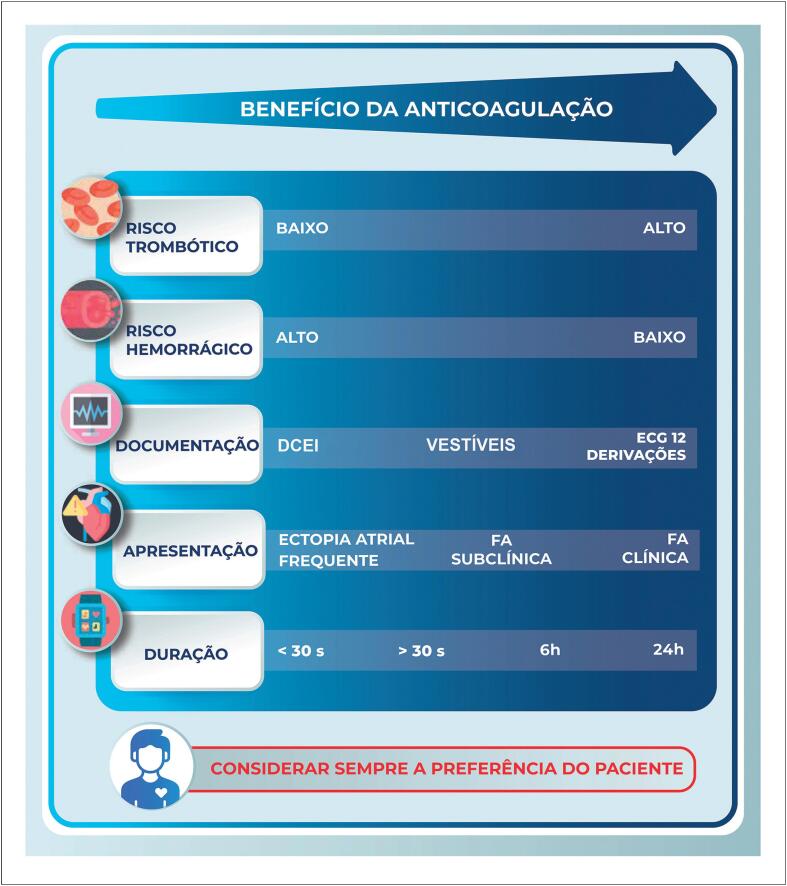
Representação esquemática dos fenótipos da fibrilação atrial (FA) com suas intersecções. Duração, presença de sintomas, risco de fenômenos tromboembólicos, risco de sangramento, forma de identificação da arritmia, evidência de benefício para anticoagulação e preferência do paciente. Itens que necessitam ser levados em consideração para melhor uso da terapia antitrombótica em uma decisão compartilhada. DCEI: dispositivos cardíacos eletrônicos implantáveis; ECG: eletrocardiograma.

Os resultados dos estudos NOAH-AFNET 6 e ARTESiA podem parecer diferentes; entretanto, não são. Apesar de o estudo NOAH-AFNET 6 não ter poder estatístico para demonstrar benefício da anticoagulação oral na população de FA subclínica, a tendência dos resultados foi semelhante aos do estudo ARTESiA. Particularmente, quando observamos os resultados dos dois estudos em relação a AVC e embolia sistêmica, ambos demonstram uma redução no RR de AVC em 32%.^227^ Portanto, quando analisamos a totalidade dos dados sobre o papel da anticoagulação oral em pacientes com FA subclínica, a anticoagulação oral com apixabana ou edoxabana pode ser considerada, e a decisão terapêutica deve ser individualizada e compartilhada levando-se em consideração vários fatores (Figura 16). Deve-se atentar que ao redor de um quarto dos pacientes evolui para FA subclínica com mais de 24 hs de duração ou até mesmo para FA clínica;^130^ portanto, a monitorização cuidadosa deve ser recomendada nos casos em que a anticoagulação não tenha sido iniciada.

### 6.6. Oclusão do Apêndice Atrial Esquerdo e Anticoagulação

#### 6.6.1. Contraindicações ao Uso de Anticoagulantes

São poucas as contraindicações absolutas ao uso de anticoagulantes orais: sangramentos agudos graves, comorbidades associadas (por exemplo, trombocitopenia grave < 50.000/µL, anemia grave sob investigação) e sangramento grave recente (por exemplo, hemorragia intracraniana). Nessas situações, nenhuma opção de terapia anticoagulante deve ser considerada. Entretanto, assim que essas condições de gravidade estejam resolvidas, o tratamento anticoagulante deve ser considerado.^228^

#### 6.6.2. Oclusão Percutânea do Apêndice Atrial Esquerdo

Estima-se que mais de 90% dos trombos em pacientes com FA não valvar originam-se no AAE.^134,229^ O procedimento percutâneo de oclusão percutânea do AAE é uma intervenção minimamente invasiva que visa ocluir o AAE com dispositivos dedicados. A eficácia e a segurança da oclusão do AAE foram demonstradas em vários estudos não randomizados e dois ensaios clínicos randomizados: PREVAIL (*A Safety and Efficacy Study of Oral MDV3100 in Chemotherapy-Naive Patients With Progressive Metastatic Prostate Cancer*) e PROTECT AF (*Watchman Left Atrial Appendage Closure Technology for Embolic Protection in Patients With Atrial Fibrillation*).^230-232^

Em um outro estudo de mundo real com 106 pacientes com FA, demonstrou-se que o procedimento é seguro e eficaz. A taxa de eventos TE foi de 3,3%/ano, com benefícios na redução de eventos esperados com base nos escores CHADS_2_ (redução relativa de 66%) e CHA_2_DS_2_-VA (redução de 59%).^233^ Resultados similares foram obtidos em outro estudo não randomizado e multicêntrico, que avaliou 150 pacientes com FA sem doença cardíaca valvar com alto risco de AVC e contraindicação à terapia com ACO. Nesse estudo, a taxa de AVCi observada de foi 1,7%/ano, que representa 77% menos eventos do que o esperado com base no escore CHADS_2_.^234^ Em outro estudo retrospectivo conduzido em Taiwan com pacientes submetidos ao implante de dispositivos Watchman ou Amplatzer Amulet/Amulet, o procedimento também foi seguro e tecnicamente viável, com resultados clínicos satisfatórios em longo prazo, com seguimento médio de 28±14 meses.^235^

Em uma metanálise de dados dos estudos randomizados PREVAIL e PROTECT AF com seguimento de 5 anos, a oclusão percutânea do AAE com dispositivo Watchman resultou em taxas de AVC, embolia sistêmica ou morte cardiovascular/inexplicada semelhantes às da varfarina. Por outro lado, os pacientes submetidos à oclusão do AAE apresentaram menos eventos hemorrágicos, incapacidade ou morte comparados com a varfarina.^236^

A oclusão do AAE para pacientes com FA também foi avaliada por revisões sistemáticas. Al-Abcha et al. concluíram que os dispositivos de OPAAE (Watchman, Watchman-FLX, Amulet e AMPLATZER AMULET) proporcionam menores taxas de AVC, embolia sistêmica e morte cardiovascular quando comparados com o tratamento medicamentoso (varfarina, ACOD e AVK). Além disso, as taxas de mortalidade por todas as causas, mortalidade cardiovascular, AVC hemorrágico, sangramento maior e sangramento maior não relacionado ao procedimento foram significativamente menores com o implante de dispositivo. Entretanto, o risco para todos os tipos de AVC, AVCi ou embolia sistêmica foram semelhantes entre os dois grupos avaliados.^237^ Dessa forma, a oclusão percutânea do AAE pode ser considerada na prevenção de tromboembolismo em pacientes com FA não associada a doença valvar e contraindicação e/ou falha à terapia com ACOD. Estudos randomizados e adequadamente delineados comparando a oclusão do AAE diretamente com os ACOD deverão fornecer dados relevantes para a prática clínica e sobre o papel da OPAAE em pacientes com FA.

#### 6.6.3. Oclusão Cirúrgica do Apêndice Atrial Esquerdo

A oclusão do AAE tem como objetivo reduzir o risco de eventos cardioembólicos e sangramento em pacientes com FA não valvar, alto risco de tromboembolismo e contraindicação para o uso crônico de ACOD.^238-241^ No estudo randomizado LAAOS III (*Left Atrial Appendage Occlusion Study III*), pacientes submetidos à oclusão cirúrgica do AAE associada a terapia anticoagulante tiveram menores taxas de AVC ou embolia sistêmica quando comparados com aqueles tratados apenas com terapia anticoagulante oral.^242^

Evidências atuais apontam que pacientes com histórico de FA submetidos à cirurgia cardíaca por outra causa devem realizar concomitantemente a oclusão do AAE.^242^ Prasad et al., em uma metanálise, demonstraram taxas de AVC no pós-operatório significativamente menores nos pacientes submetidos a oclusão do AAE, tanto precocemente (30 dias após o procedimento) como em longo prazo (> 2 anos).^241^ A abordagem cirúrgica também pode ser considerada em pacientes com episódios repetitivos de trombo e que não apresentam indicação para intervenção percutânea.^243^

Desde a primeira intervenção cirúrgica para oclusão do AAE descrita em 1949, diversas abordagens foram propostas e incluem a amputação do AAE, a exclusão com grampeador cirúrgico (Endo GIA II, EZ45), a exclusão com sutura, a oclusão com *patch*, além de dispositivos como AtriClip^®^.^239,243,244^ A indicação e a estratégia utilizada baseiam-se nas características clínicas e cirúrgicas individuais de cada paciente (Tabela 15).

**Tabela 15:** Abordagens cirúrgicas e perfil do paciente para indicação

Abordagem cirúrgica	Características clínicas e cirúrgicas
Exclusão com grampeador ou amputação do apêndice	Cirurgias cardíacas que não envolvam incisão do átrio esquerdo (revascularização de coronárias com CEC, cirurgia de válvula aórtica)
Ligadura com sutura ou oclusão com *path*	Cirurgia cardíaca concomitante que envolvam a incisão do átrio esquerdo (cirurgia da válvula mitral, Cox-Maze) Apêndice com tecido frágil Reabordagem cirúrgica Alto risco de sangramento Pacientes com contraindicação ou falha do tratamento percutâneo
Exclusão com uso de dispositivo	Cirurgias cardíacas que não envolvam incisão do átrio esquerdo (revascularização de coronárias com CEC, cirurgia de válvula aórtica) Alto risco de sangramento Reabordagem cirúrgica Pacientes com contraindicação ou falha do tratamento percutâneo

22 CEC: circulação extracorpórea.

Embora estudos comparando as diferentes técnicas cirúrgicas sejam escassos,^242,245^ alguns autores sugerem que a amputação do AAE é superior às outras técnicas sem dispositivos. Isso porque, com o tempo, quando o apêndice está intacto, as suturas ou grampos que fecham o orifício vão corroendo gradativamente as paredes do AAE, fazendo com que ele reabra.^246^ Oclusões incompletas do AAE aumentam em cinco vezes o risco de AVC ou embolia sistêmica.^247^

Os dispositivos de clipagem epicárdica como o AtriClip^®^ têm mostrado resultados promissores. Um estudo randomizado demonstrou segurança e eficácia desse método, com taxas de sucesso superiores a 95%.^248^ O clipe epicárdico para oclusão do AAE pode ser utilizado em procedimentos com esternotomia mediana, em procedimentos minimamente invasivos^249^ ou naqueles assistidos por robótica.^250^ Recentemente, Franciulli et al. avaliaram os efeitos da oclusão do AAE com o AtriClip^®^ via toracoscopia em pacientes com alto risco de sangramento e demonstraram taxas de sucesso do procedimento em 100% dos casos, sem eventos adversos cerebrais ou mortalidade em curto prazo (6 meses).^251^ Resultados semelhantes foram obtidos por Branzoli et al.^252^ Logo, a intervenção é uma opção em pacientes com contraindicação para terapia com anticoagulante ou dupla antiagregação plaquetária.

Uma nova técnica foi proposta por Ghinescu et al. durante cirurgias cardíacas robóticas em câmara direita. Nela, o AAE é invaginado no AE, excisado completamente na base com uma tesoura, e o coto é, então, fechado por dentro com uma sutura em *loop*. Até o momento, apenas 20 pacientes foram submetidos à técnica, e todas foram realizados com sucesso (ressecção completa do AAE sem coto residual).^253^ Apesar dos excelentes resultados iniciais, a técnica padrão-ouro para a oclusão do AAE ainda é incerta. Estudos randomizados comparando a técnica cirúrgica com a terapia anticoagulante são necessários. As recomendações da oclusão percutânea e cirúrgica do AEE estão demonstradas no Quadro 12.

**Quadro 12:** Recomendações da oclusão percutânea e cirúrgica do apêndice atrial esquerdo (AAE) em pacientes com fibrilação atrial (FA)

Recomendações da oclusão percutânea e cirúrgica do AAE no paciente com FA	Classe de recomendação	Nível de evidência
A indicação de fechamento ou exclusão cirúrgica do AAE deve ser considerada em pacientes com FA submetidos à cirurgia cardíaca	**I**	**B**
A indicação de fechamento ou exclusão percutânea do AAE deve ser considerada em pacientes com FA de alto risco tromboembólico e contraindicação absoluta ou falha da terapia anticoagulante	**IIa**	**B**
A suspensão da anticoagulação em pacientes submetidos à exclusão cirúrgica do AAE tem benefício incerto	**IIb**	**A**

#### 6.6.4. Anticoagulação após Oclusão do Apêndice Atrial Esquerdo

##### 6.6.4.1. Anticoagulação após Oclusão Percutânea do Átrio Esquerdo

O selamento da prótese é o resultado do crescimento do endotélio normal na superfície do dispositivo. A endotelização parcial ou completa ocorre usualmente dentro de 30 a 90 dias após a oclusão percutânea do AAE.^254^ Nesse período, o uso de antitrombóticos é recomendado para evitar a ocorrência de trombose do dispositivo.

Uma metanálise que incluiu mais de 10.000 pacientes mostrou trombose de prótese em 3,8% dos casos. Nesses, a ocorrência de eventos isquêmicos cerebrais foi significativamente maior do que naqueles sem trombose do dispositivo.^255^ Fatores como estado de hipercoagulabilidade, efusão pericárdica, disfunção renal, profundidade do implante superior a 10 mm e FA não paroxística estiveram associados a maior ocorrência de trombose de prótese.^256^

Os estudos pivotais PROTECT-AF e PREVAIL utilizaram protocolos desenhados para minimizar o risco de trombose relacionada ao procedimento e tromboembolismo incluindo exames de imagem, seguimento clínico e terapia antitrombótica. Tais protocolos, entretanto, não foram avaliados de maneira randomizada, e estudos de mundo real mostraram que apenas cerca de 12% dos pacientes recebem o regime aprovado pela Food and Drug Administration (FDA) na prática clínica. O uso de AVK ou ACOD parece favorável quando usados isoladamente com taxas extremamente baixas de trombose do dispositivo e fenômenos TE. Estudos em andamento como o ADALA (*Apixaban vs Dual Antiplatet Therapy Study After Left Atrial Appendage Occlosure*), ANDES (*Short-Term Anticoagulation Versus Antiplatelet Therapy for Preventing Device Thrombosis Following Left Atrial Appendage Closure*), APPROACH (*Multimodal Approach in Patients With mHSPC. Randomized Trial of APA+ADT vs APA-ADT and Local Treatment*), DEA-LAA (*Efficacy of Short Term Dabigatran Etexilate Followed by Aspirin Monotherapy After Left Atrial Appendage Device Closure*) e FADE-DRT (*Efficacy of Different Anti-Thrombotic Strategies on Device-Related Thrombosis Prevention After Percutaneous Left Atrial Appendage Occlusion*) avaliarão, de maneira randomizada, os efeitos dos ACOD após a exclusão percutânea do AAE.

O uso de antiplaquetários nesse cenário também é empírico, e são altas as taxas de descontinuação durante o seguimento clínico. Em andamento, o estudo ASPIRIN-LAAO (*Aspirin Discontinuation After Left Atrial Appendage Occlusion in Atrial Fibrillation*) avaliará os efeitos da descontinuação da terapia com aspirina 6 meses após o implante.

A ocorrência de vazamentos no dispositivo é comum e apresenta incidência variável. Em uma grande coorte nacional que avaliou mais de 50.000 pacientes, pequenos vazamentos (1-5 mm) estiveram presentes em cerca de 25% dos pacientes, enquanto vazamentos > 5 mm foram raros (< 1%).^257^ Vazamentos superiores a 5 mm representam oclusão incompleta e requerem continuação ACO ou seu fechamento.

Dispositivos com melhor selamento, uso de bainhas deflectíveis que permitam o alinhamento coaxial com AAE e planejamento da escolha do tipo e tamanho do dispositivo com exame de imagem são fatores importantes para minimizar o risco de trombose relacionada ao dispositivo. Em pacientes com risco muito alto de sangramento, o uso de oclusores com acesso epicárdico sem necessidade de terapia antitrombótica pode ser considerado.

Os estudos em andamento OPTION, CHAMPION-AF e CATALYST avaliarão os efeitos de próteses WATCHMAN Flex e AMULET em pacientes elegíveis a ACOD em comparação ao uso de ACOD. Portanto, de maneira geral, enquanto não tivermos os resultados dos inúmeros ensaios clínicos randomizados em andamento, a terapia antitrombótica nesse cenário continuará empírica.

##### 6.6.4.2. Terapia Antitrombótica após Oclusão Cirúrgica do Apêndice Atrial Esquerdo

O estudo LAAOS III comparou a oclusão cirúrgica do AAE vs. não oclusão em pacientes com FA, com escore CHA_2_DS_2_-VA ≥ 2 e que foram submetidos à cirurgia cardíaca. Esse estudo, com quase 5 mil pacientes randomizados, demonstrou redução de 33% nas taxas de AVCi no grupo submetido à oclusão sem aumento na ocorrência de eventos adversos, sugerindo efeitos benéficos adicionais à anticoagulação.^242^ Não é estabelecido que a suspensão da ACOD nesses casos é segura, e a terapia anticoagulante oral em longo prazo deve ser mantida com base no risco TE individual (escore CHA_2_DS_2_-VA) mesmo em casos onde a oclusão cirúrgica foi com sucesso.

### 6.7. Estratégias para Minimizar o Risco de Sangramento

A morte decorrente de hemorragia é um problema que acomete cerca de 1,9 milhão de pessoas no mundo anualmente, sendo 1,5 milhão decorrente de trauma físico. A terapia anticoagulante é um dos principais fatores de risco para sangramento, sendo a maioria dos pacientes atendida em serviços de emergência nos Estados Unidos. Esses pacientes têm maior morbimortalidade que os pacientes que não estão em uso dessas medicações.^258^ Portanto, antes de iniciar o tratamento com ACOD, é fundamental a avaliação das condições clínicas do paciente, bem como o conhecimento dos mecanismos de ação dos anticoagulantes e a fisiopatologia da hemorragia a fim de minimizar o risco e tratar os eventos decorrentes do tratamento, caso ocorram.

Os ACOD são superiores à varfarina quanto ao risco de hemorragia intracraniana, e seu uso tem crescido progressivamente a cada ano. Entretanto, o risco persiste e é preciso considerar a possibilidade de traumas e do desenvolvimento de outras condições mórbidas que aumentam a incidência de sangramento, sobretudo no paciente idoso, como a insuficiência renal e a desidratação.^17^

A primeira etapa na avaliação é utilizar um escore de risco de sangramento e, a partir daí, considerar a relação risco/benefício. O HAS-BLED apresenta a melhor evidência na predição de risco de sangramento.^17^ Muitos dos parâmetros utilizados no escore CHA_2_DS_2_-VA e HAS-BLED são superponíveis, e escore de sangramento elevado não deve ser contraindicação ao uso de anticoagulantes.^134^

O antecedente de quedas, sobretudo em pacientes mais idosos, usualmente é visto com preocupação na decisão de iniciar o tratamento; entretanto, não é um preditor independente de sangramento nos pacientes em uso de ACOD. Além disso, os ACOD exercem efeito protetor nos pacientes acometidos por queda quando comparados com aqueles em uso de varfarina.^259^ É importante ressaltar que história de quedas não é um preditor independente de sangramento em pacientes em uso de ACOD. Dados estimam que 295 quedas ao ano seriam necessárias para que os benefícios da redução do AVCi com uso de ACOD fossem superados pelo potencial de sangramento grave.

A estratégia mais eficiente para diminuir o risco de sangramento em pacientes em uso de ACOD é a correta avaliação dos fatores de risco, que podem ser agrupados nas categorias de fatores não modificáveis, potencialmente modificáveis e modificáveis (Tabela 16, Quadros A-B-C). Embora de menor aplicabilidade clínica, alguns biomarcadores parecem ser úteis na estratificação do risco de sangramento (Tabela 16, Quadro D).^17^

**Tabela 16:** Fatores de risco para sangramento no tratamento com anticoagulantes orais e antiplaquetários. Adaptado de Hindricks et al.^17^

Não modificáveis - Quadro A
Idade > 65 anos
Antecedente de sangramento maior
Insuficiência renal grave (em diálise ou transplantado renal)
Insuficiência hepática grave (cirrose)
Doença maligna
Fatores genéticos (por exemplo, polimorfismos CYP 2C9)
AVC prévio, doença de pequenos vasos etc.
Diabetes melito
Deficiência cognitiva/demência
Potencialmente modificáveis - Quadro B
Fragilidade extrema ± risco excessivo de quedas
Anemia
Redução do número ou função plaquetária
Insuficiência renal com ClCr < 60 mL/min
Estratégia de manejo de antagonistas da vitamina K
Modificáveis - Quadro C
Hipertensão/pressão sistólica elevada
Uso concomitante de antiplaquetário/anti-inflamatórios não hormonais
Ingestão excessiva de álcool
Não adesão aos ACOD
Diversões/ocupações perigosas
Terapia de "ponte" com heparina
Controle de RNI (alvo: 2,0-3,0)
TTR > 70%
Escolha apropriada e dose correta do ACOD
Biomarcadores - Quadro D
GDF-15
Cistatina C/CKD-EPI
cTnT-hs
Fator von Willebrand (+ outros marcadores de coagulação)

AVC: acidente vascular cerebral; ClCr: clearance de creatinina; ACOD: anticoagulantes orais de ação direta; INR: razão normalizada internacional; TTR: tempo no intervalo terapêutico; GDF-15: fator de diferenciação e crescimento 15; CDK-EPI: colaboração para epidemiologia da doença renal crônica; cTnT-hs: troponina cardíaca T de alta sensibilidade.

A identificação dos fatores modificáveis e potencialmente modificáveis é de fundamental importância para que possam ser adotadas medidas capazes de revertê-los e, assim, tornar possível e mais segura a terapia anticoagulante.

Fatores como HAS não controlada, uso abusivo de bebidas alcóolicas ou uso de anti-inflamatórios não hormonais ou aspirina, por exemplo, podem ser modificados, deixando de ser incluídos como fatores de risco de sangramento nesses pacientes. Para alcançar esses objetivos, faz-se necessária uma boa relação médico-paciente, para que seja obtida a adesão do paciente às orientações médicas baseadas sobretudo em informações sobre os riscos e benefícios do tratamento.

É preciso também considerar que os fatores de risco de sangramento são dinâmicos, e sua presença pode variar ao longo do tempo, sendo recomendado que os pacientes que não tiveram o seu tratamento com ACOD iniciado devido ao risco de sangramento sejam periodicamente reavaliados para que o tratamento seja instituído assim que esses fatores estejam superados.^134^

### 6.8. Escolha do Anticoagulante Oral

Nenhum estudo clínico comparou de forma direta os ACOD. Logo, não é possível determinar se um ACOD é superior ou mais seguro que o outro. Perfis clínicos individuais apresentam efeitos mais favoráveis ao uso específico de um ACOD e estão sumarizados na Tabela 17.

**Tabela 17:** Sugestões para o uso de anticoagulantes orais de ação direta (ACOD) de acordo com diferentes situações clínicas em pacientes com FA

Situação clínica	Anticoagulante oral sugerido
Idoso	Apixabana ou edoxabana
Múltiplas comorbidades	Apixabana ou edoxabana
Alto risco de AVCi com baixo risco de sangramento	Dabigatrana
Alto risco de sangramento	Apixabana ou edoxabana
Alto risco de sangramento gastrointestinal	Apixabana
Disfunção renal	Apixabana ou edoxabana
Estenose mitral moderada/grave ou prótese valvar mecânica	Varfarina
Bioprótese valvar	Rivaroxabana
Síndrome coronariana aguda	Apixabana + clopidogrel (ticagrelor pode ser considerado. Evitar prasugrel)
Síndrome coronariana crônica	Qualquer ACOD sem antiagregante plaquetário
Comodidade posológica	Edoxabana ou rivaroxabana

AVCi: acidente vascular cerebral isquêmico; FA: fibrilação atrial. Obs: Não há estudos com comparação direta entre os diferentes ACOD. Essas sugestões são baseadas em análises de subgrupos dos múltiplos estudos pivotais e não indicam que outros ACOD não possam ser utilizados. Cada paciente deve ser individualizado, e os riscos de AVC e sangramento devem ser discutidos com o paciente e familiares para uma decisão compartilhada.

## 7. Tratamento do Ritmo ou da Frequência Cardíaca na Fibrilação Atrial

A estratégia de tratamento da FA se baseia no controle do ritmo, ou seja, estratégia de manutenção do ritmo sinusal, e no controle da resposta ventricular, mantendo o paciente em FA.^17,260^ A escolha de uma ou outra estratégia dependerá das características clínicas de cada paciente.

### 7.1. Fármacos Utilizados no Controle do Ritmo

Os medicamentos antiarrítmicos estão divididos em quatro classes distintas, de acordo com a classificação proposta por Vaughan-Williams.^261^ A classe I é composta pelos bloqueadores dos canais de sódio, a classe II é composta pelos betabloqueadores, a III, pelos bloqueadores dos canais de potássio e a classe IV, pelos bloqueadores dos canais de cálcio não diidropiridínicos. No Brasil, poucos desses agentes estão disponíveis comercialmente, sobre os quais vamos nos concentrar no escopo desta Diretriz.

#### 7.1.1. Propafenona

A propafenona é um bloqueador dos canais rápidos de sódio (classe IC de Vaughan-Williams). Sua utilização para manutenção de ritmo sinusal deve ser recomendada em pacientes sem isquemia miocárdica ou cardiopatia estrutural, sendo a disfunção sistólica do VE uma contraindicação para o seu uso, uma vez que fármacos dessa classe, como a flecainida, estão relacionados ao aumento da mortalidade em pacientes com essas características, conforme demonstrado no estudo CAST (*Cardiac Arrhythmia Suppression Trial*).^262^ Sua eficácia também já foi comprovada na reversão aguda dos episódios de FA, modalidade "*pill in the pocket*", na dose de 450 a 600 mg em uma tomada, podendo ser acrescida de doses complementares de 300 mg a cada 8 horas.^263^

Após a absorção por via oral, a propafenona tem uma meia-vida curta (cerca de 5 a 8 h), requerendo no uso contínuo duas ou três tomadas diárias. A dose usual varia de 150 mg em duas tomadas diárias até 300 mg em três tomadas diárias.

Os efeitos adversos da propafenona ocorrem em maior frequência no início do tratamento e nos pacientes que utilizam doses mais elevadas do medicamento. As bradiarritmias sinusais ou por bloqueios AV estão associadas ao uso concomitante de outros fármacos cronotrópicos negativos ou em pacientes suscetíveis. O efeito adverso mais preocupante com o uso da propafenona, principalmente quando utilizada em dose alta, inclusive na estratégia "*pill-in-the-pocket*", é o *flutter* tipo IC, pois pode estar associada a condução AV 1:1 e alta resposta ventricular, podendo muitas vezes ser mal tolerados.^264^

Queixas relativamente comuns incluem sensação de gosto metálico e sintomas neurológicos como tontura, cefaleia, insônia, visão turva e pesadelos. Pacientes com antecedentes de broncoespasmo podem ter exacerbação das crises devido ao seu efeito betabloqueador. Outros efeitos raros incluem a ocorrência de síndrome semelhante ao lúpus, agranulocitose e disfunção hepática.^265^

#### 7.1.2. Amiodarona

A amiodarona é a droga antiarrítmica (DAA) frequentemente utilizada para controle do ritmo na FA. Seu efeito predominante é de bloqueio dos canais de potássio, característico de classe III. No entanto, suas propriedades antiarrítmicas extrapolam esse efeito, tendo ação no bloqueio dos canais de sódio e de cálcio e no bloqueio não competitivo dos receptores beta e alfa-adrenérgicos, o que lhe confere características de todas as classes de antiarrítmicos. Apesar do potencial risco de aumento do intervalo QT e efeito pró-arrítmico, a incidência de *Torsades de Pointes* é baixa, estando mais relacionada à administração de doses elevadas, à ocorrência de distúrbios eletrolíticos, principalmente a hipocalemia, e ao uso concomitante de drogas que contribuem para aumento do intervalo QT, como outros agentes antiarrítmicos, fenotiazinas, antidepressivos tricíclicos, diuréticos tiazídicos, entre outros.

A administração da amiodarona pode ser por via intravenosa ou oral. Embora a primeira também seja utilizada no tratamento do episódio agudo da FA, o índice de reversão para RS nesta situação é relativamente baixo. O uso oral, por sua vez, requer maior tempo de administração para obtenção do efeito farmacológico máximo, geralmente observado após alguns meses do início da terapêutica. Por ser uma droga lipossolúvel, o acúmulo nos tecidos favorece a manutenção de níveis terapêuticos da droga, observados mesmo após alguns meses da suspensão do uso. Por outro lado, a afinidade por determinados tecidos, como o hepático e o pulmonar, podem ser responsáveis por sua toxicidade relacionada a esses órgãos.^266^

A amiodarona é a droga mais eficaz no controle do ritmo, chegando a ser duas vezes mais efetiva que a propafenona e o sotalol na prevenção da recorrência dos episódios.^267^ A grande vantagem da amiodarona é poder ser utilizada nos pacientes com cardiopatia estrutural e disfunção sistólica do VE, tanto para a manutenção do RS quanto no controle de FC – neste último, em situações excepcionais após a utilização de betabloqueadores e digital.

A maior limitação do uso crônico da amiodarona está relacionada a efeitos adversos que levam à descontinuidade do tratamento em cerca de 15% dos pacientes. Esse fato pode ser minimizado pelo uso de doses inferiores a 200 mg/d, que costumam manter a eficácia em uma parcela considerável de pacientes. As taxas de efeitos adversos variam de 6% em doses de 200 mg a 2% em doses mais baixas.^268^ Em cerca de 1% dos pacientes, pode surgir hipertireoidismo, situação de mais difícil controle, muitas vezes obrigando a descontinuidade da droga. A toxicidade pulmonar gerando fibrose pulmonar ocorre em cerca de 3% dos pacientes.^269^ Esse quadro é geralmente relacionado ao uso de doses elevadas, em alguns casos podendo ser reversível com a retirada dessa medicação. A impregnação corneana é comum, mas raramente se traduz em diminuição da acuidade visual. Os pacientes em uso crônico de amiodarona devem realizar periodicamente avaliação da função renal e hepática, dosagem de hormônios tireoidianos, radiografia de tórax e avaliação oftalmológica, além de outros exames específicos dependendo da suspeita do acometimento de outros órgãos.^270^

#### 7.1.3. Sotalol

O sotalol é uma molécula constituída por uma mistura racêmica entre os isômeros d-sotalol e o l-sotalol. A porção d (dextrógira) apresenta propriedades antiarrítmicas de classe III (bloqueio dos canais de potássio), e a porção l (levógira) tem efeito betabloqueador predominante. A eliminação desse fármaco é totalmente renal, devendo haver redução da dose em pacientes com ClCr abaixo de 60 mL/min e contraindicação ao seu uso no caso de ClCr menor que 40 mL/min.

Em pacientes com FA, o sotalol apresenta eficácia semelhante à propafenona e inferior à amiodarona. É particularmente útil em pacientes com cardiopatia isquêmica e FEVE acima de 40%, não sendo recomendado em pacientes com disfunção sistólica grave em razão do aumento de mortalidade nessa população, observada no estudo SWORD (*Survival With Oral d-Sotalol*), e deve ser evitada em pacientes como hipertrofia miocárdica importante.^271^ A dose recomendada varia entre 160 e 320 mg/d dividida em duas tomadas diárias. Os efeitos adversos extracardíacos são comuns aos demais betabloqueadores, sendo esse fármaco geralmente bem tolerado quando utilizado em doses usuais.

### 7.2. Fármacos Utilizados no Controle da Frequência Cardíaca

#### 7.2.1. Betabloqueadores

Os betabloqueadores, antiarrítmicos de classe II na classificação de Vaughan-Williams, são os agentes preferenciais utilizados para o controle de FC, tanto na fase aguda como no acompanhamento ambulatorial. Esses fármacos bloqueiam predominantemente os receptores beta-adrenérgicos, embora possam exercer bloqueio alfa-adrenérgico causando vasodilatação periférica, efeito menos pronunciado quanto maior a cardiosseletividade de cada agente. Os receptores β1 encontrados no coração, em maior número no nó sinusal e no nó AV, exercem papel importante na modulação do RS e da condução AV. O bloqueio β2 adrenérgico promove broncoespasmo, efeito limitante em indivíduos com hiperreatividade brônquica como os asmáticos e portadores de DPOC. Embora o bisoprolol, o metoprolol e o atenolol sejam cardiosseletivos, essa propriedade é mais pronunciada nos dois primeiros, nessa ordem, sendo os agentes com menor potencial para efeitos adversos extracardíacos. Tanto o bisoprolol, o carvedilol e o metoprolol podem ser utilizados em pacientes com disfunção sistólica do VE.

Na FA persistente/permanente, os betabloqueadores são os agentes mais amplamente utilizados para reduzir a resposta ventricular.^272^ Apesar da sua reconhecida eficácia no controle da FC, seu impacto na redução de mortalidade e hospitalizações e na melhora na QV dos pacientes vem sendo motivo de grande discussão nos últimos anos. Os benefícios dos betabloqueadores são mais pronunciados nos pacientes com IC e após IAM, principalmente em relação a menor incidência de FA.^273^ Contudo, a esperada redução na mortalidade não é observada naqueles pacientes que já desenvolveram a arritmia.^274^ Evidências mais consistentes são necessárias no sentido de comprovar a superioridade de alguma classe específica de medicamentos na estratégia de controle da FC na FA.

Dependendo da sensibilidade individual de cada paciente, efeitos cardiovasculares indesejados, como bradicardia significativa e bloqueios AV, podem ocorrer, obrigando a redução da dose ou mesmo a suspensão do medicamento. Efeitos extracardíacos são descritos em um percentual razoável de pacientes e são menos comuns quanto maior a cardiosseletividade do agente. Entre os efeitos adversos mais relatados, além do broncoespasmo já descrito, estão fadiga, tontura, insônia, pesadelos e disfunção erétil. Em pacientes diabéticos, os sinais de hipoglicemia podem ser mascarados.

#### 7.2.2. Bloqueadores do Cálcio

Os bloqueadores dos canais de cálcio não diidropiridínicos, como o verapamil e o diltiazem, têm efeitos farmacológicos semelhantes aos betabloqueadores, diminuindo o automatismo sinusal e a condução AV. Esse efeito ocorre por bloqueio da corrente lenta de cálcio, atuando de forma predominante nas células de resposta lenta do nó sinusal e do nó AV, sendo classificados como antiarrítmicos de classe IV. São eficazes no controle da FC em pacientes com FA,^275^ podendo ser utilizados como agente de primeira linha ou como alternativa aos betabloqueadores, principalmente nos pacientes que apresentam contraindicação ou efeitos adversos relacionados a esses últimos. Devido ao seu efeito inotrópico negativo, não devem ser utilizados em pacientes com FEVE < 40%.

#### 7.2.3. Digoxina

A recomendação atual do uso da digoxina deve ser restrita a pacientes cujo controle de FC permaneça inadequado, já em uso de betabloqueadores ou bloqueadores de cálcio ou com intolerância a esses fármacos.^276^ O uso da digoxina em pacientes com doença renal avançada deve ser evitado. A associação com amiodarona, quando necessária, deve ser feita com cautela devido ao risco de toxicidade.

A Tabela 18 traz informações úteis no manejo dos antiarrítmicos utilizados no tratamento da FA.

**Tabela 18:** Aspectos práticos relacionados aos antiarrítmicos utilizados no tratamento da fibrilação atrial (FA)

Fármaco	Via de eliminação	Posologia	Reações adversas extracardíacas	Principais interações medicamentosas
Propafenona	Hepática/renal	150 a 300 mg (2-3x/d)	Broncoespasmo, tontura, turvação visual, gosto metálico, cefaleia, insônia	Varfarina, dabigatrana, edoxabana, cetoconazol, fluoxetina, paroxetina
Amiodarona	Hepática	100 a 400 mg (1x/d)	Hipotireoidismo, hipertiroidismo, fibrose pulmonar, neuropatia, fotossensibilidade, hepatotoxicidade	Digoxina, diltiazem, verapamil, varfarina, dabigatrana, edoxabana, fenitoína, ciclosporina, estatinas, sildenafila, antidepressivos tricíclicos
Betabloqueadores				
Atenolol	Hepática/renal	25 a 100 mg (1-2x/d)	Broncoespasmo, fadiga, tontura, insônia, pesadelos, extremidades frias, náusea, constipação, disfunção erétil, ganho de peso, hiperglicemia, atenuação dos sinais de hipoglicemia	Amiodarona, fluoxetina, paroxetina, digoxina, clonidina, fenobarbital, AINEs
Metoprolol	Hepática/renal	25 a 200 mg (1-2x/d)		
Propranolol	Hepática/renal	40 a 240 mg (2-3x/d)		
Bisoprolol	Hepática/renal	1,25 a 10 mg (1x/d)		
Carvedilol	Hepática	3,125 a 50 mg (2x/d)		
Sotalol	Renal	80 a 160 mg (2-3x/d)		
Bloqueadores de cálcio				
Verapamil	Hepática/renal	80 a 160 mg (2-3x/d)	Fadiga, tontura, constipação, cefaleia, náusea, edema em tornozelo, constipação, hiperplasia gengival (verapamil)	Digoxina, ivabradina, colchicina, carbamazepina, fenitoína, fenobarbital, ciclosporina, estatinas, dabitrana, edoxabana
Diltiazem	Hepática/renal	30 a 120 mg (2-3x/d)		
Digoxina	Renal	0,125 a 0,25 mg (1x/d)	Vertigem, turvação visual, vômito, diarreia	Alprazolam, ciclofosfamida, doxorrubicina, metotrexato, cetoconazol, betabloqueadores, amiodarona, verapamil, diltiazem

AINES: anti-inflamatórios não esteroides.

### 7.3. Escolha entre Controle do Ritmo ou Controle da Frequência Cardíaca

O tratamento antiarrítmico da FA deve ter como objetivo o controle dos sintomas e a prevenção de complicações cardiovasculares. Duas estratégias terapêuticas são possíveis nesse contexto: o controle do ritmo (mantendo o paciente em RS) ou o controle da FC (sem priorizar a reversão da arritmia). A escolha da melhor estratégia para o tratamento antiarrítmico foi objeto de controvérsia há mais de 20 anos.^277,278^

Do ponto de vista fisiológico, o RS apresenta inúmeras vantagens em relação à FA. A manutenção da contração atrial e da estrutura atrial normal, do sincronismo AV, da resposta fisiológica da FC diante das diferentes demandas, da regularidade do intervalo RR, entre outros são fatores que estão relacionados a menor trombogenicidade, à diminuição de sintomas e à preservação da função sistólica do VE, diminuindo o risco de eventos que impactam no prognóstico.

Atualmente, sabemos que a manutenção do RS é superior ao controle de FC em pacientes com FA. O estudo EAST AFNET 4 (*Early Therapy of Atrial Fibrillation for Stroke Prevention Trial*) apresentou menor mortalidade, menor incidência de eventos TE e hospitalização em pacientes em que se objetivava o controle do ritmo.^279^ Esses dados foram confirmados por metanálise publicada em 2023, onde foi demonstrada confirmando uma redução superior a 20% em desfechos de mortalidade, incidência de AVCi e hospitalização por IC.^280^

O controle do ritmo deve ser uma estratégia prioritária em pacientes com maior chance de manutenção de RS (idade < 65 anos, com remodelamento atrial discreto a moderado). Da mesma forma, o controle do ritmo é preferencial em situações com relevante impacto na redução da morbimortalidade a médio e longo prazos, como: pacientes sintomáticos, com difícil controle da FC, taquicardiomiopatia, IC e nos pacientes com alto risco tromboembólico.

O controle da FC pode ser uma estratégia em situações de pacientes muito idosos ou assintomáticos com função ventricular normal. As recomendações para o controle do ritmo ou da FC em pacientes com FA estão apresentadas no Quadro 13.

**Quadro 13:** Recomendações para controle do ritmo da frequência cardíaca (FC) no paciente com fibrilação atrial (FA)

Recomendação	Classe de recomendação	Nível de Evidência
O controle do ritmo está recomendado como estratégia de tratamento preferencial.	**I**	**A**
A estratégia de controle de FC pode ser utilizada em pacientes assintomáticos ou com baixa probabilidade de manutenção do ritmo sinusal.	**IIa**	**A**

### 7.4. Controle da Frequência

O controle da frequência ventricular durante a FA é importante para evitar o desenvolvimento de taquicardiomiopatia ou sintomas em médio a longo prazo. Algumas condições, como tônus vagal aumentado e uso de fármacos que lentificam a condução AV, reduzem a resposta ventricular durante FA. Por outro lado, condições como hipertireoidismo, excesso de catecolaminas ou síndrome de Wolff-Parkinson-White podem ser responsáveis por frequências ventriculares mais elevadas.

Para controle farmacológico da frequência ventricular, os fármacos mais usados são os antagonistas dos canais de cálcio (diltiazem, verapamil), betabloqueadores, digoxina e eventualmente amiodarona. A ablação da junção AV com implante de marca-passo é uma opção não farmacológica para controle de frequência.

#### 7.4.1. Meta Terapêutica

Quando o controle da FC é o tratamento empregado, o alvo da FC de repouso deve ser observado, sendo a monitorização ambulatorial (sistema Holter) uma ferramenta importante na quantificação da FC média em 24 h. O controle da FC deve objetivar FC em repouso abaixo de 80 bpm, porém o controle mais leniente da FC (FC em repouso abaixo de 110 bpm) em pacientes assintomáticos não foi associado a um aumento de eventos cardiovasculares.^281^

#### 7.4.2. Controle Agudo

A presença de FA aguda e instabilidade hemodinâmica geralmente tem indicação para cardioversão elétrica, mas ocorrem situações em que isso não é possível ou que não se obtém sucesso. Nessas situações, assim como naquelas em que a resposta ventricular é elevada, mas não há instabilidade hemodinâmica, o controle da FC de forma aguda é uma opção adequada. Devemos sempre afastar possíveis causas secundárias para a instabilidade, que precisam ser descartadas e tratadas, como desidratação, sangramento, anemia ou infecção, principalmente no pronto-socorro.

Em relação aos fármacos, os mais utilizados são os betabloqueadores, como metoprolol ou esmolol. Como existe a chance de ocorrer hipotensão arterial, o betabloqueador mais indicado é o esmolol, por sua meia-vida mais curta. Uma metanálise comparativa recente aponta para resultados mais efetivos com diltiazem do que com metoprolol no controle agudo da FC,^282^ entretanto os antagonistas do cálcio endovenosos têm disponibilidade reduzida no Brasil.

Quando não se obtém o controle adequado com esses fármacos, a opção seguinte é acrescentar digitálico intravenoso, sendo a última opção farmacológica a amiodarona intravenosa.

O sulfato de magnésio intravenoso associado a drogas que bloqueiam o nó AV também tem um papel no controle agudo da resposta da FA. O mecanismo mais provável é o bloqueio da entrada dos canais lentos de cálcio do nó AV.^283^ A principal vantagem dessa droga é a segurança.^283-286^ Em uma metanálise recente, o uso do magnésio endovenoso foi superior em comparação à estratégia convencional na obtenção de controle de resposta ventricular (63% *versus* 40%; *odds ratio* [OR], 2,49 intervalo de confiança [IC] 1,80-3,45) e modestamente efetivo na conversão para ritmo sinusal (21% *versus* 14%; OR, 1,75 [IC, 1,08-2,84]).^285^ A ivabradina também pode ter um papel no controle da resposta ventricular.^287,288^

A transição das doses via intravenosa para a via oral é feita avaliando a dose total diária intravenosa e utilizando a mesma dose via oral nas 24 horas, tomada em uma ou mais vezes, de acordo com a formulação dos comprimidos.

A Tabela 19 apresenta as drogas utilizadas para controle agudo da FC

**Tabela 19:** Doses intravenosas usadas para controle agudo da frequência cardíaca

Droga	Dose
**Esmolol**	500 microgr/kg em bolus por 1 min; manutenção 10-40 microgr/kg/min (1-10 em casos de disfunção ventricular)
**Metoprolol tartarato**	2,5-5 mg intravenoso em bolus; no máximo 4 doses
**Amiodarona**	300 mg diluídos em soro glicosado a 5% por 30-60 min; manutenção 900-1.200 mg intravenoso em 24 horas; via mais central possível
**Sulfato de magnésio**	Dose baixa: 4,5 g diluídos em 100 mL infundidos em 30 minutos Dose alta: 9 g diluídos em 100 mL infundidos em 30 minutos
**Deslanosídeo**	Dose: Digitalização rápida (24 h) em casos de urgência: 0,8-1,6 mg, endovenosa ou intramuscular, fracionados em 1-4 administrações. Manutenção: 0,2-0,6 mg/dia, intramuscular ou endovenosa.

#### 7.4.3. Controle em Longo Prazo

O controle crônico da FC é geralmente obtido com o uso de betabloqueadores ou antagonistas dos canais de cálcio, medicações que funcionam tanto em repouso como durante o esforço. A digoxina, que tem menos efeito durante momentos de alta carga adrenérgica, e a amiodarona também são alternativas eventualmente usadas.^289^ Em uso isolado ou associado de fármacos, o controle da frequência é obtido em mais de 80% dos pacientes, mas isso deve ser feito com cuidado e acompanhamento.

Os betabloqueadores podem ser usados em pacientes com função sistólica normal ou reduzida e são especialmente indicados em pacientes com cardiopatia isquêmica e em cargas adrenérgicas elevadas. No caso de disfunção ventricular, são mais indicados o succinato de metoprolol, carvedilol ou bisoprolol.

Os antagonistas dos canais de cálcio reduzem o inotropismo cardíaco e não devem ser usados em disfunção sistólica. O verapamil tem melhor efeito na redução da resposta ventricular do que o diltiazem, mas também tem maior efeito inotrópico negativo. Geralmente é a opção preferencial em pacientes com doenças broncopulmonares e contraindicação aos betabloqueadores.

A digoxina tem maior indicação em pacientes com disfunção ventricular sistólica, mas é realmente mais usada como terapia combinada aos betabloqueadores ou antagonistas dos canais de cálcio. Por perder efeito em exercício, é pouco usada como monoterapia, especialmente em pacientes jovens, mesmo que um trabalho recente mostre bons resultados na comparação com bisoprolol em relação à qualidade de vida em pacientes com IC.^290^ O uso da digoxina requer cuidado, já que a faixa terapêutica não é distante da faixa da intoxicação, e trabalhos descrevem aumento de mortalidade, especialmente com concentração de digoxina sérica > 1,2 ng/mL.^291^

A amiodarona via oral pode eventualmente ser usada no contexto de controle da resposta ventricular, mas isso é bastante incomum, devido ao seu elevado perfil de efeitos colaterais no longo prazo. A Tabela 20 apresenta as drogas e doses utilizadas para controle crônico da resposta ventricular em pacientes com FA

**Tabela 20:** Doses via oral diárias para controle de frequência cardíaca em longo prazo

Droga	Dose
**Diltiazem**	180-360 mg
**Verapamil**	80-480 mg
**Atenolol**	25-100 mg
**Bisoprolol**	1,25-20 mg
**Carvedilol**	6,25-100 mg
**Metoprolol succinato**	50-400 mg
**Metoprolol tartarato**	50-200 mg
**Nebivolol**	2,5-10 mg
**Digoxina**	0,0625-0,25 mg
**Amiodarona**	100-400 mg (geralmente 200 mg 5-7x/semana)

#### 7.4.4. Ablação do Nó Atrioventricular

A ablação do nó AV e o implante de marca-passo definitivo é uma alternativa extremamente eficiente quando a estratégia de controle medicamentoso da resposta ventricular falhou ou não foi tolerada. A principal vantagem dessa estratégia é que, embora invasiva, é extremamente eficiente, simples e de baixo risco, levando a uma melhora nos sintomas, QV e capacidade ao exercício.^17,292,293^

Essa estratégia pode ser empregada em pacientes que já são portadores de DCEI implantados, seja por doença do nó sinusal, pacientes com ressincronização cardíaca ou pacientes portadores de CDI.^294^ Nos pacientes sem dispositivo cardíaco, o implate deve ser realizado anteriormente e, algumas semanas após, a ablação do nó AV pode ser realizada.

Para a realização da ablação, o dispositivo deve ser inibido a fim de que seja possível o mapeamento do eletrograma do His. O local da ablação geralmente é a região do nó AV à direita, em local mais proximal com eletrograma de átrio, His e ventrículo, pois, dessa maneira, é obtido um bloqueio mais proximal e, no caso de ocorrer acidentalmente a perda de captura do marca-passo, um escape juncional pode ser melhor tolerado.^295^ Em algumas situações onde não é possível o bloqueio nesta região, pode-se realizar na região do His mais distal ou, em pacientes com bloqueio de ramo direito, existe a necessidade de abordagem da região do His no VE.

Um ponto extremamente importante é que, nas primeiras semanas após a ablação do nó AV, os pacientes devem ser mantidos sob estimulação com frequência de 80-90 bpm,^17^ pois existem relatos de casos de taquicardia ventricular polimórfica espontânea após ablação do nó AV, e essa estratégia pode prevenir essa complicação.^296,297^

Em pacientes com ressincronização cardíaca e FA, a estratégia de ablação do nó AV a fim de garantir maior taxa de estimulação pode ser essencial em algumas situações.^298,299^ No estudo clínico CERTIFY (*Cardiac Resynchronization Therapy in Atrial Fibrillation Patients Multinational Registry*)^300^, a mortalidade total (6,8 contra 6,1 por 100 pessoas-ano) e cardíaca (4,2 contra 4,0) foi semelhante nos pacientes com terapia de ressincronização com FA e ablação do nó AV e os pacientes que estavam em ritmo sinusal.

Recentemente, foram implementadas técnicas de estimulação fisiológica no His e do ramo esquerdo.^294^ Tanto os pacientes com estimulação do His^301,302^ quanto aqueles com estimulação no ramo esquerdo,^303^ a realização de ablação do nó AV é possível, segura e efetiva.

Poucos estudos compararam a estratégia de controle do ritmo através da ablação por cateter de FA e controle da resposta ventricular com a ablação do nó AV. O estudo PABA-CHF (*Randomized Controlled Trial of Pulmonary Vein Antrum Isolation vs. AV Node Ablation With Bi-Ventricular Pacing for Treatment of Atrial Fibrillation in Patients With Congestive Heart Failure*) avaliou pacientes com IC (classes II e III da NYHA) e disfunção ventricular (FEVE igual ou menor a 40%), demonstrando melhora na FE, QV e teste de caminhada no grupo submetido a ablação de FA em comparação a ablação do nó AV com implante de marca-passo em um seguimento de 6 meses.^304^ Em um registro alemão com 4.444 pacientes submetidos a ablação de FA e 234 submetidos a ablação do nó AV, os pacientes submetidos a ablação do nó AV eram em média 10 anos mais velhos (71±10 contra 61±10 anos, p < 0,001), apresentavam mais comorbidades cardiovasculares (44% contra 7%, p < 0,001), e maior mortalidade em 1 ano de seguimento (9,8% contra 0,5%), porém em ambos os grupos houve melhora nos sintomas. As hospitalizações foram menores nos pacientes submetidos a ablação do nó AV (31% contra 18%, p < 0,001).^305^

O Quadro 14 apresenta as recomendações de ablação do nó AV.

**Quadro 14:** Recomendações para procedimentos de ablação do nó atrioventricular (AV)

Recomendações	Classe de recomendação -+.	Nível de evidência
A ablação do nó AV está recomendada para pacientes com cardiodesfibrilador implantável com terapias inapropriadas por fibrilação atrial (FA) ou flutter atrial com alta resposta ventricular e que não são elegíveis para a ablação por cateter.	**I**	**B**
Em pacientes com FA e resposta ventricular elevada submetidos a ablação do nó AV, a frequência cardíaca (FC) inicial de estimulação do marca-passo deve ser mantida entre 80 e 90 bpm para reduzir o risco de morte súbita.	**I**	**C**
A ablação do nó AV está recomendada para pacientes com terapia de ressincronização por estimulação biventricular, His ou ramo esquerdo que apresentem FA ou flutter atrial com baixa taxa de estimulação e que não são elegíveis para a ablação por cateter	**I**	**B**
A ablação do nó AV deve ser considerada para o controle da FC em pacientes portadores de marca-passo definitivo que não responderam ou são intolerantes às estratégias de controle e ritmo ou da FC	**IIa**	**B**
A ablação do nó AV deve ser considerada para o controle da FC em pacientes que não responderam ou são intolerantes a estratégia de controle do ritmo ou da FC e não são elegíveis a ablação por cateter de FA, aceitando-se que esses pacientes serão dependentes de marca-passo	**IIb**	**B**

### 7.5. Controle do Ritmo – Situações Especiais

#### 7.5.1. Cardioversão Elétrica

A FA é assintomática ou causa sintomas leves na maioria dos pacientes. Entretanto, se houver instabilidade hemodinâmica, a cardioversão elétrica sincronizada deve ser realizada de imediato (200 J bifásico).^17,306^ Nos casos estáveis, a cardioversão elétrica restaura o ritmo sinusal de forma mais rápida e efetiva do que os fármacos antiarrítmicos, reduzindo o tempo de hospitalização, porém necessita de jejum adequado e sedação. O posicionamento anteroposterior das pás promove passagem de maior energia pelo AE. Entretanto, em uma metanálise recente, não houve diferença na reversão para ritmo sinusal quando comparados os posicionamenteos anteroposterior e anterolateral.^307^ O pré-tratamento com DAA ajuda a otimizar a cardioversão, principalmente prevenindo as recorrências imediatas. Um ensaio randomizado recente demonstrou que a aplicação de choques com energia máxima é mais eficaz do que o aumento escalonado da carga, podendo ser alternativa em casos refratários às doses habituais (200 J). A compressão ativa durante a cardioversão elétrica também é uma medida efetiva no aumento de obtenção de RS.^308^

#### 7.5.2. Cardioversão Química

A utilização de DAA para reversão ao RS é bastante frequente dada sua praticidade. Contudo, os antiarrítmicos podem acarretar efeitos adversos graves, como a *torsades de pointes*, especialmente em pacientes idosos, com múltiplas comorbidades e alterações eletrolíticas (hipocalemia). Além disso, a eficácia das DAA na reversão da FA é relativamente pequena em comparação ao placebo, em parte porque a reversão espontânea dessa arritmia é alta, especialmente nas formas paroxísticas. Em recente ensaio clínico randomizado, a conduta expectante (apenas controle da FC e cardioversão, se necessário, em 48 horas) foi equivalente à cardioversão imediata em pacientes com FA paroxística.^309^ Essa estratégia pode ser considerada em pacientes com FA de início recente, embora seja de difícil aplicação em nosso meio. As drogas disponíveis no Brasil para a cardioversão farmacológica se restringem à amiodarona e à propafenona, sendo que apenas a amiodarona pode ser usada em pacientes com cardiopatia estrutural. As doses recomendadas para a cardioversão farmacológica, assim como seus principais efeitos colaterais, estão demonstradas na Tabela 21.

**Tabela 21:** Dosagens dos fármacos utilizados na reversão da fibrilação atrial e manutenção do ritmo sinusal

Fármaco	Reversão	Manutenção do ritmo	Eventos adversos
**Amiodarona**	EV 150-300 mg diluída em SG 5% (infundir em 15-30 min) seguida de 900 mg nas 24 horas	Ataque: 600 a 800 mg/dia em doses divididas até o total de 10 g. Manutenção 200-400 mg 1 vez ao dia	Bradicardia, TdP, fotossensibilidade,
			Hipo/hipertiroidismo
			Toxicidade pulmonar, hepática, polineuropatia
**Propafenona**	450-600 mg dose única oral ou EV 1,5 a 2,0 mg/kg em 10 a 20 min	150-300 mg 3 vezes ao dia	Bradicardia, pró-arritmia, distúrbios gastrointestinais, cefaleia
**Sotalol**	Não indicado	80-160 mg 2 vezes ao dia	Bradicardia, TdP, broncoespasmo

EV: endovenoso; SG: soro glicosado; TdP: torsades de pointes.

A cardioversão eletiva (elétrica ou química) deve ser precedida de avaliação do risco embólico, abordada na Seção 6.5.1

##### 7.5.2.1. Uso do Esquema "Pill-in-the-pocket"

A FA paroxística é por definição autolimitada, entretanto, os esquemas *pill-in-the-pocket* têm sido utilizados para abreviar a duração da FA e reduzir as visitas ao pronto-socorro, especialmente nos pacientes com crises sintomáticas e infrequentes. Da mesma forma, esses esquemas podem ser utilizados em FA persistente de início recente (< 7 dias), pois é sabido que quanto mais precoce for seu uso, melhores são os resultados de reversão ao RS. O sucesso da reversão para RS varia de 56 a 94%.^263,310^ Os esquemas "*pill-in-the-pocket*" devem ser utilizados somente em pacientes com coração estruturalmente normal ou com mínimas alterações, devendo ser evitados em pacientes com hipertrofia ventricular (> 13 mm), disfunção ventricular ou miocardiopatia isquêmica.

Uma vez definida a escolha por esse esquema, deve ser administrada uma dose de betabloqueador ou antagonista do cálcio anteriormente à DAA, visando evitar a ocorrência de FLA 1:1, o que pode acontecer em 3 a 7% dos casos.^311^ Sendo assim, deve ser sempre utilizada pela primeira vez em ambiente hospitalar para avaliação da segurança dessa estratégia. A propafenona é a medicação utilizada na dose de 600 mg (dois comprimidos) em dose única, podendo ser reduzida para 450 mg em pessoas com menos de 70 kg.^263^

A associação de doses adicionais de propafenona visando o tratamento "*pill-in-the-pocket*" em pacientes que fazem uso crônico de tal droga foi estudada em pequenas séries de casos, sempre buscando-se respeitar a dose máxima total de 900 mg/dia. Se o paciente já faz uso de 600 mg/dia, adicionar somente 300 mg no esquema obteve sucesso na reversão de 77%.^312^

### 7.6. Tratamento Farmacológico para Manutenção do Ritmo Sinusal

Apesar das limitações, o tratamento farmacológico continua sendo um alicerce no tratamento da FA. As DAA comumente utilizadas na manutenção do RS disponíveis em nosso meio são propafenona, sotalol e amiodarona.

A escolha da DAA para a manutenção do RS deve respeitar as características individuais dos pacientes, como fatores de risco e comorbidades. É importante lembrar que essas drogas têm potenciais efeitos colaterais e contraindicações (Tabela 21).

**Figura 17:**
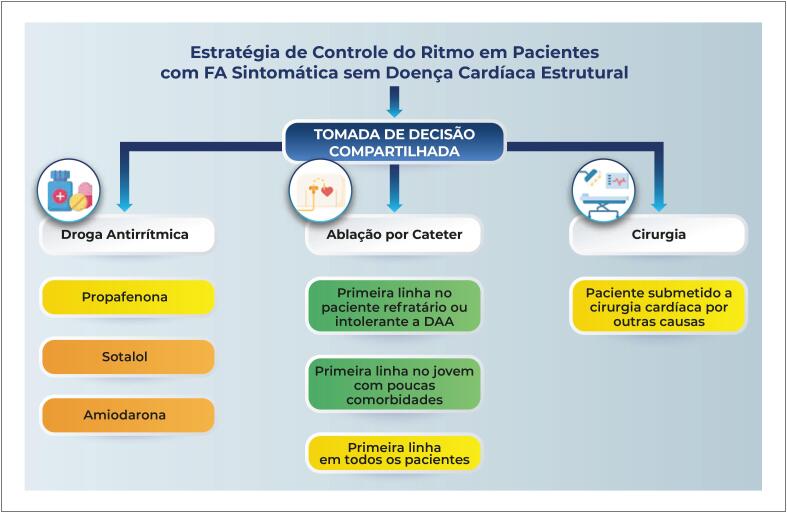
Estratégias de controle do ritmo em pacientes com fibrilação atrial (FA) sintomática sem doença cardíaca estrutural. DAA: droga antiarrítmica.

As Figuras 17 e 18 demonstram as estratégias de controle do ritmo em pacientes com FA sintomática sem doença cardíaca estrutural e em pacientes com insuficiência cardíaca, respectivamente

**Figura 18:**
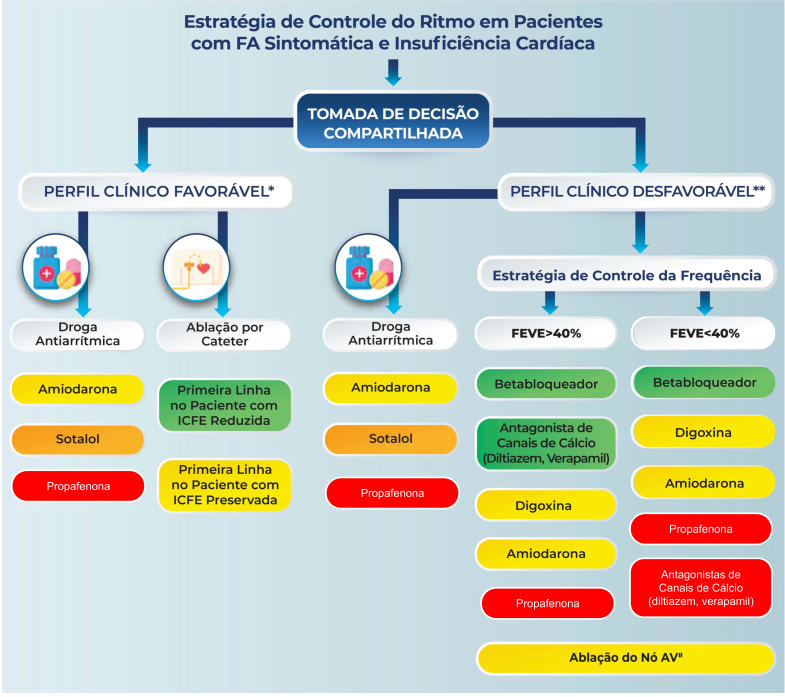
Estratégias de controle do ritmo em pacientes com fibrilação atrial (FA) sintomática e insuficiência cardíaca. ICFE: Insuficiência cardíaca com fração de ejeção; FEVE: fração de ejeção do ventrículo esquerdo; AV: atrioventricular. *Taquicardiomiopatia, jovens com poucas comorbidades, átrio pouco dilatado, formas paroxísticas ou persistente recente, insuficiência cardíaca inicial, ventrículo com pouca fibrose. **Insuficiência cardíaca avançada, fibrose ventricular, cardiomiopatia fibrótica atrial avançada, recorrência após ablação, muito idoso com várias comorbidades, fA de longa duração. ^#^Ablação do nó AV em casos de falha no controle da frequência e ritmo.

A propafenona tem sido utilizado como fármaco de primeira linha em indivíduos com coração estruturalmente normal, e seu uso é contraindicado nos pacientes com doença cardíaca estrutural (DAC e IC) devido ao risco de indução de arritmias ventriculares e aumento de mortalidade.^17,22^ O controle do ritmo da FA a longo prazo com a propafenona é considerado satisfatório. Em um estudo com pacientes tratados com propafenona de liberação sustentada 325 mg duas vezes ao dia, a taxa de recorrência em 1 ano foi de 41% em comparação com 68% naqueles tratados com placebo.^313^

O sotalol possui taxa de eficiência equivalente à da propafenona na manutenção do RS.^267^ Contudo, seu uso é mais restrito, e esse fato é atribuído principalmente ao risco de proarritmias (*torsades de pointes*). Esse fármaco possui capacidade de prolongar a refratariedade atrial e ventricular e, consequentemente, o aumento do intervalo QT. Os fatores de risco incluem bradicardia, sexo feminino, comprometimento renal e hipertrofia ventricular esquerda.^314^ Sendo assim, nos pacientes com FA tratados com sotalol, é recomendado o monitoramento do intervalo QT, níveis séricos de potássio, ClCr e outros riscos de proarritmia. O uso do sotalol é contraindicado em pacientes com IC de fração de ejeção reduzida (ICFEr).^17^

A amiodarona tem demonstrado ser a DAA mais eficaz na manutenção do RS;^267,315^ contudo, é associada ao risco de complicações cardíacas e não cardíacas. A amiodarona pode ser utilizada para a manutenção do RS em todos os pacientes com FA, entretanto, pelo risco de eventos adversos, tem sido recomendada preferencialmente aos pacientes com doença cardíaca estrutural (DAC, doença valvar e ICFEr).^267,316^

## 8. Ablação de Fibrilação Atrial

### 8.1. Tecnologias de Mapeamento e Ablação

#### 8.1.1. Sistemas de Mapeamento Tridimensional

Após a descoberta de Haïssaguerre et al.^317^ sobre o papel de focos arritmogênicos no interior das veias pulmonares no início e manutenção da FA, o isolamento elétrico das veias pulmonares se consolidou como o pilar do tratamento da arritmia.^318,319^ Com o objetivo de identificar corretamente o substrato anatômico e facilitar a navegação no interior do AE, foram incorporadas técnicas auxiliares de imagem como mapeamento eletroanatômico 3D e ecocardiograma intracardíaco. Hoje, o uso dessas tecnologias para guiar a ablação é considerado o tratamento padrão, reduzindo tempo de procedimento e exposição à fluoroscopia.^320,321^

Os sistemas de mapeamento auxiliam na visão direta do cateter, ao navegar em uma anatomia tridimensional, orientação que apresenta visualização limitada pela radioscopia utilizada tradicionalmente nos laboratórios de eletrofisiologia. Os três sistemas mais utilizados no Brasil são CARTO (Biosense Webster), EnSite (Abbott Medical) e Rhythmia (Boston Scientific). A tecnologia é baseada na reconstrução tridimensional da cavidade atrial e veias pulmonares através da criação de um campo eletromagnético (CARTO e Rhythmia) ou de alterações na voltagem e impedância tecidual (NavX), definindo com precisão a anatomia, o substrato funcional e a ativação elétrica, além de marcar as lesões de radiofrequência no mapa criado, reduzindo a complexidade da técnica e necessidade de fluoroscopia.^322^

#### 8.1.2. Eco Intracardíaco na Ablação de Fibrilação Atrial

O papel do ecocardiograma intracardíaco (EIC) como uma modalidade de imagem para auxílio à ablação de FA teve sua utilização, no início,^323^ focada na punção transeptal guiada pelo eco, através da identificação precisa da fossa oval e determinação do melhor sítio de punção, permitindo um acesso seguro para o AE sem necessidade de contraste iodado.^324,325^ Uma utilidade mais recente do EIC durante todas as etapas do procedimento de ablação, principalmente quando associado aos sistemas de mapeamento eletroanatômico, é a de reduzir e até mesmo eliminar a necessidade do uso da fluoroscopia.^326^

**Figura 19:**
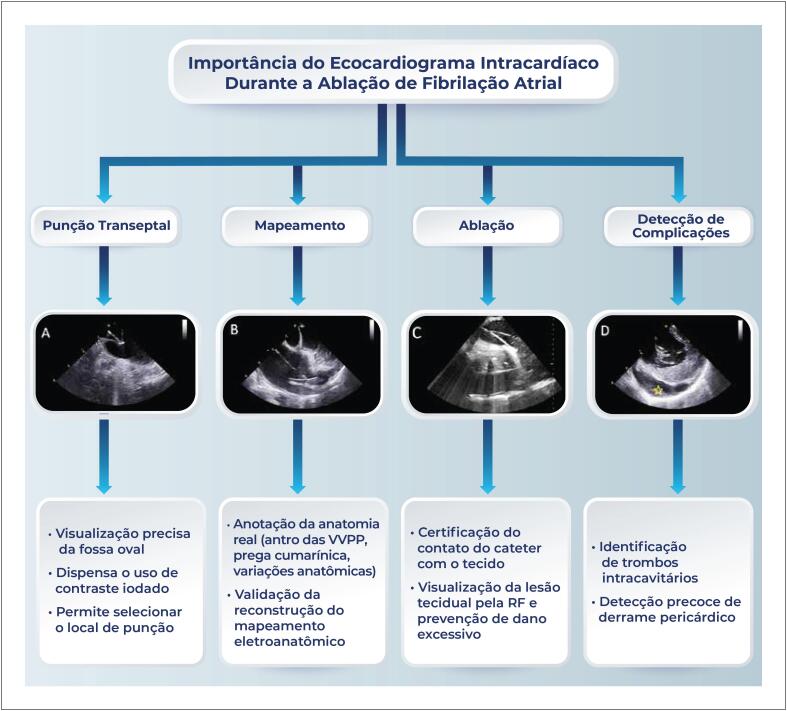
Uso do ecocardiograma intracardíaco em um procedimento de fibrilação atrial. A: Visualização da agulha na fossa oval durante a punção transeptal. B: Com a sonda posicionada no ventrículo direito, uma imagem do átrio esquerdo permite a delineação da prega cumarínica e do apêndice atrial esquerdo. C: Aplicação de radiofrequência (RF) próximo à veia pulmonar inferior esquerda. Uma área hiperrefringente no tecido adjacente ao cateter corresponde a uma aplicação efetiva, motivando a movimentação do cateter para outra região, evitando lesão excessiva. D: Derrame pericárdico identificado através do posicionamento da sonda de ecocardiograma intracardíaco no ventrículo direito no início do procedimento. Foi possível a condução segura do procedimento através da comparação de imagens no início e no fim da ablação. VVPP: veias pulmonares.

Durante a criação do mapa eletroanatômico intracavitário, o EIC é de extrema utilidade para delinear a anatomia em tempo real, conferindo ao mapa informações extremamente relevantes como a localização real dos óstios e antros das veias pulmonares, bem como de regiões de difícil delimitação como a prega cumarínica (limites entre o apêndice atrial esquerdo e a região anterior das veias pulmonares esquerdas) (Figura 19B) ou identificação de veias pulmonares supranumerárias ou de drenagem anômala. O esôfago também pode ser visualizado pelo ecocardiograma intracardíaco,^327^ permitindo a identificação de lesões com potencial de lesão esofágica.^328^

Durante o isolamento das veias pulmonares, uma adequada aplicação de RF depende diretamente do tempo de contato do cateter de ablação com o tecido atrial, e um posicionamento instável do cateter pode propiciar uma reconexão futura, devido a uma lesão não transmural. O EIC permite a monitorização do contato do cateter com o tecido em tempo real.

Por outro lado, uma aplicação de RF pode levar à formação de excessiva reação tecidual que é facilmente identificada pelo EIC^329^ como uma área hiperrefringente no tecido atrial (Figura 19C). Esse fenômeno usualmente é um precursor da formação de uma explosão tecidual ("*pop*"), com significativo potencial de complicações graves como a perfuração cardíaca. Portanto, a monitorização em tempo real da formação da lesão tecidual pelo ecocardiograma intracardíaco é útil para prevenir complicações nessa fase do procedimento. De fato, em um grande estudo recente envolvendo mais de 100.000 pacientes submetidos a ablação de FA, o não uso do EIC foi relacionado a um risco 4,8 vezes maior de perfuração cardíaca.^330^

Devido à capacidade de monitorização ultrassonográfica em tempo real, o EIC permite a detecção precoce de complicações graves como o derrame pericárdico (Figura 19D), permitindo o seu diagnóstico antes mesmo de ocorrer hipotensão secundária ao tamponamento cardíaco. A visualização do espaço pericárdico no início do procedimento e periodicamente durante toda a ablação é desejável, permitindo a comparação das imagens em diferentes momentos e a detecção de derrame pericárdico em suas fases iniciais, antes do aparecimento de suas complicações hemodinâmicas. Com isso, medidas terapêuticas (reversão da anticoagulação, drenagem percutânea) podem ser tomadas precocemente para evitar o colapso hemodinâmico.

A formação de trombos intracardíacos durante a ablação por cateter também é uma condição associada a alto risco embólico, e EIC é a ferramenta ideal para sua rápida detecção e adequado tratamento.^331-333^ Também no cenário pré-procedimento, o EIC realizado em sítios específicos do coração (seio coronário e artéria pulmonar) permite a visualização e a detecção dos trombos no AAE de forma equivalente ao ecocardiograma transesofágico.^334-336^ Quando utilizado para avaliação de pacientes com trombo no AAE detectado ao ecocardiograma transesofágico, o EIC realizado na artéria pulmonar foi capaz de detectar casos de "falsos-positivos".^337^

Todas essas características em conjunto conferem segurança ao procedimento de ablação e foram responsáveis pela rápida disseminação de seu uso. Em um estudo que avaliou apenas o acesso transeptal, o ecocardiograma intracardíaco foi um fator independente para a prevenção de complicações no acesso ao AE.^338^ Houve também redução significativa nas taxas de complicação e na emissão de fluoroscopia em procedimentos de ablação de FA em que foi usado o ecocardiograma intracardíaco.^339-341^ Embora exista um custo adicional associado ao seu uso,^342^ essa modalidade de imagem mostrou uma redução no tempo médio de permanência hospitalar em relação aos pacientes que não utilizaram esta tecnologia, o que impactou positivamente no custo hospitalar.^343^ A Figura 19 demonstra a importância do ecocardiograma intracardíaco na ablação da FA.

#### 8.1.3. Tecnologias de Ablação

Em mais de 20 anos de evolução, ocorreram inúmeros avanços na tecnologia dos sistemas de mapeamento, cateteres e formas de energia para lesão tecidual. A ablação ponto a ponto convencional por RF é a mais utilizada, sendo recomendado o uso de cateteres de ablação irrigados e com sensor de força de contato, aumentando eficácia no isolamento permanente e segurança das lesões térmicas no AE.^344-347^

Em relação à efetividade a longo prazo da ablação por RF, o maior entendimento fisiopatológico da formação da lesão levou ao aprimoramento dos cateteres e tecnologias com a criação de índices de qualidade de lesão que incorporam força de contato, potência e tempo de aplicação. O conhecimento da interação entre estes fatores otimizando a aplicação de RF é essencial na durabilidade das lesões, reduzindo reconexão das veias pulmonares e recorrência da arritmia.^348^

Outra tecnologia validada é o congelamento (crioablação), o qual utiliza um cateter-balão posicionado no antro das veias pulmonares sendo capaz de realizar toda a lesão simultaneamente na circunferência em contato com o tecido, o que diminui o tempo total do procedimento, porém com exposição maior à fluoroscopia. Estudos randomizados comparando as duas estratégias demonstraram resultados semelhantes, sendo a energia escolhida conforme a disponibilidade da tecnologia e experiência do eletrofisiologista.^348,349^

Atualmente, a utilização de sistemas eletroanatômicos utilizando força de contato se faz mandatória quando utilizadas tecnologias ponto a ponto, associadas se possível ao uso de ecocardiografia intracardíaca, visando a uma ablação mais efetiva, melhorando resultados e reduzindo complicações.^321,322,340-342^ A crioablação não necessita de associação aos sistemas eletroanatômicos, entretanto sua associação com EIC aumenta a segurança e reduz a exposição ao raio X.

Mais recentemente, tem sido estudada uma nova modalidade de energia baseada em impulsos elétricos de alta energia e curta duração aplicados no tecido-alvo ("eletroporação") ou ablação por campo pulsado ("*pulsed field ablation*"), levando a apoptose celular. Como os tecidos têm diferentes limiares para destruição induzida por campos elétricos, essa tecnologia visa atingir seletivamente a célula cardíaca, evitando, assim, lesões no esôfago ou nervo frênico.^350^ Os ensaios clínicos iniciais e registros apresentam resultados promissores em relação ao sucesso no isolamento elétrico completo das veias, durabilidade das lesões e recorrência clínica em 1 ano de acompanhamento.^351-357^ Reddy et al. demonstraram no ADVENT trial (*The FARAPULSE ADVENT PIVOTAL Trial PFA System vs SOC Ablation for Paroxysmal Atrial Fibrillation*), um estudo de não inferioridade, que a ablação de campo pulsado foi equivalente à ablação térmica para um desfecho combinado de eficácia que inclui falha no isolamento das veias pulmonares, arritmia atrial com duração maior que 30 segundos, uso de DAA das classes I e III e desfecho de segurança.^356^ Entretanto, ainda faltam dados mais robustos em relação ao protocolo ideal de energia, número de pulsos, frequência, orientação e configuração do cateter, para garantir eficácia sem complicações como excessiva contração muscular, aquecimento tecidual ou formação de bolhas levando a fenômenos embólicos.

Como conclusão, as ferramentas disponíveis são cada vez maiores na prática clínica e cada tecnologia apresenta seus prós e contras. A escolha por qual recurso utilizar deve ser norteada pelo custo, disponibilidade e experiência do centro e equipe médica.

### 8.2. Indicações para a Realização de Ablação de Fibrilação Atrial

A experiência clínica adquirida e o avanço tecnológico têm proporcionado maiores efetividade e segurança no tratamento da FA. O tratamento da FA através da ablação apresenta maior taxa de sucesso se comparado ao tratamento com fármacos antiarrítmicos.^358,359^ Ainda não existem evidências claras de que a intervenção seja superior às DAA na redução de mortalidade.^360^ Uma metanálise de cinco ensaios clínicos com 994 pacientes demonstrou menor recorrência de taquicardias atriais e de FA sintomática e menor taxa de internação hospitalar nos pacientes tratados com ablação por cateter em comparação a aqueles tratados com DAA.^361^ A decisão de indicar a ablação para um paciente deve levar em consideração a taxa de recorrência do procedimento e as taxas de complicações. Vários fatores foram identificados como preditores de recorrência de FA após ablação por cateter. Estudo com 702 pacientes submetidos ao primeiro procedimento de ablação por RF ou por crioablação identificaram que a síndrome metabólica e a perda de função renal são preditores independentes de menor eficácia da ablação.^362^ Essa análise deu origem ao escore ALARMEc (tipo de FA, tamanho do AE, insuficiência renal, síndrome metabólica e cardiomiopatia), testado posteriormente como preditor de recorrência em 213 pacientes, porém, na análise isolada, somente o tipo de FA (não paroxística) e o AE aumentado foram preditores independentes.^363^ Um estudo brasileiro com 95 pacientes identificou maior diâmetro anteroposterior do AE como preditor de recorrência (HR 2,58 para cada milímetro ajustado, IC95% 1,36-4,89).^364^ Já no estudo que gerou o escore CAAP-AF, de fácil aplicação, foram identificados seis preditores de recorrência: presença de DAC, diâmetro aumentado do AE, idade avançada, FA persistente ou persistente longa, falha terapêutica a fármacos antiarrítmicos e sexo feminino. Com pontuação entre 0 e 13 pontos, os autores observaram sobrevida livre de recorrência de FA em 2 anos de seguimento na coorte de desenvolvimento de 100% se escore = 0 até 29,1% se escore > 10 pontos. Os resultados foram replicados na coorte de validação, com sobrevida livre de FA em 2 anos de 100% se escore = 0 até 51,3% se escore > 10 pontos.^365^ Recentemente, foi publicado um escore baseado em um registro europeu de ablação de FA que utilizou mecanismos de inteligência artificial para identificação de preditores de recorrência pós-procedimento, com 3.128 pacientes divididos em coortes de derivação (80% da amostra) e validação (20% da amostra). Esse escore apresentou boa acurácia para avaliação de recorrência em 1 ano (área sob a curva [AUC] 0,72), sendo incluídas no modelo final variáveis clínicas como idade, gênero, comorbidades e classificação da FA (paroxística ou persistente) e dados ecocardiográficos de volume diastólico do VE, FEVE, diâmetro anteroposterior do AE e escore CHAD_2_DS_2_-VA.^366^

Ainda não há escore ideal para a predição de recorrência, sendo que a melhor indicação da ablação deve considerar uma avaliação mais intensa dos fatores de risco e ajustar esses fatores à situação individual do paciente. O que se observa é que a presença de FA persistente, o diâmetro ou volume aumentados do AE e a presença de comorbidades estão relacionados a maiores taxas de recorrência.

As indicações de ablação de FA estão apresentadas no Quadro 15 e nas Figuras 17 e 18.

**Quadro 15:** Indicações de ablação de fibrilação atrial (FA)

Recomendações	Classe de recomendação	Nível de evidência
Considerações Gerais		
Antes de considerar a ablação por cateter de FA, é recomendado que o paciente seja orientado em relação aos riscos e desfechos do procedimento.	**I**	**C**
A repetição da ablação deve ser considerada em pacientes que recorreram arritmia, com melhora inicial dos sintomas após a ablação e dados do procedimento prévio sugerem chance de manutenção do ritmo sinusal.	**IIa**	**B**
Ablação por Cateter após Falha de Medicação		
A ablação por cateter de FA é recomendada em pacientes portadores de FA paroxística ou persistente, sintomáticos e refratários ou intolerantes a pelo menos um antiarrítmico da classe I ou III.	**I**	**A**
A ablação por cateter de FA é recomendada em pacientes portadores de FA paroxística ou persistente, sintomáticos e refratários ou intolerantes a betabloqueador.	**IIa**	**B**
A ablação por cateter de FA deve ser considerada em pacientes portadores de FA paroxística com pausas prolongadas no momento do término da FA, com vistas a evitar implante de marca-passo definitivo	**IIa**	**C**
Ablação por Cateter como Tratamento de Primeira Linha		
A ablação por cateter de FA deve ser considerada como terapia de primeira linha em pacientes portadores de FA paroxística sintomática em caso de escolha do paciente	**I**	**B**
A ablação por cateter de FA pode ser considerada como terapia de primeira linha em pacientes portadores de FA persistente sintomática em caso de escolha do paciente	**IIa**	**B**
A ablação por cateter de FA é recomendada como terapia de primeira linha em pacientes portadores de FA suspeita de induzir taquicardiomiopatia (portadores de ICFEr), independentemente de sintomas.	**I**	**B**
A ablação por cateter de FA deve ser considerada como terapia de primeira linha em pacientes portadores de ICFEr visando melhora de mortalidade e redução de hospitalização por insuficiência cardíaca	**IIa**	**A**

ICFEr: Insuficiência cardíaca com fração de ejeção reduzida.

#### 8.2.1. Estudos Clínicos em Ablação de Fibrilação Atrial

Inúmeros estudos clínicos randomizados foram realizados nas diversas populações de pacientes com FA com desfechos variando de QV, recorrência de FA e redução de mortalidade principalmente nos pacientes com IC. Além disso, também foram testadas tecnologias diferentes.

Nos pacientes com FA paroxística, o isolamento antral das veias pulmonares proporciona um índice livre de recorrências que varia entre 60 e 79%,^349,367,368^ podendo atingir patamares de 77% em seguimentos de longo prazo.^369^ Por outro lado, a ablação da FA persistente tem tido resultados menos favoráveis, e modificações adicionais do substrato arritmogênico, além do isolamento das veias pulmonares, têm sido recomendadas, mas ainda sem grandes impactos nos benefícios clínicos dessa terapia.^370,371^ De fato, os resultados de longo prazo na ablação de FA persistente têm sido inferiores aos da FA paroxística, e novos estudos são necessários para o melhor entendimento dos mecanismos arritmogênicos envolvidos. Também é importante destacar que muitos pacientes requerem sessões adicionais de ablação, e recorrências tardias não são infrequentes, particularmente da FA persistente.^372,373^

A abordagem clínica mais precoce tem sido estudada com objetivo de evitar a progressão da cardiomiopatia atrial e ter, como consequência, um cenário clínico mais favorável na manutenção do RS. De fato, os resultados dos ensaios clínicos EARLY-AF (*Early Aggressive Invasive Intervention for Atrial Fibrillation*)^374^ e EAST-AFNET 4 (*Early Treatment of Atrial Fibrillation for Stroke Prevention Trial*)^279^ indicam que uma intervenção mais precoce pode levar a resultados clínicos mais favoráveis, possivelmente por conta da contenção e desaceleração do progressivo remodelamento patológico e deletério do AE. O estudo ATTEST (*The Randomized Controlled Atrial Fibrillation Progression Trial*) demonstrou que a ablação por RF foi superior às DAA no retardo da progressão da FA paroxística para a sua forma persistente, em um seguimento médio de 3 anos.^375^ Do mesmo modo, o estudo EARLY-AF demonstrou a mesma vantagem da crioablação como estratégia inicial em comparação com a terapia farmacológica em pacientes com FA paroxística, com seus resultados reconfirmados em recente publicação de seguimento clínico médio de 3 anos.^375^ Além disso, em revisão sistemática e metanálise de estudos observacionais, pode-se também afirmar que um período mais curto entre o diagnóstico da FA e a ablação aumenta as chances no sucesso dessa terapêutica.^376^ Finalmente, uma metanálise de Han et al.^280^ demonstrou que a estratégia de um controle do ritmo de forma precoce pode ser mais benéfica do que o controle da frequência nos pacientes com FA. Os autores incluíram nesta metanálise sete estudos: dois ensaios clínicos randomizados, uma análise retrospectiva de ensaio clínico randomizado e quatro estudos observacionais. O tempo médio de seguimento variou de 1 a 5 anos. A análise agrupada demonstrou que a estratégia precoce no controle do ritmo obteve menor índice de mortalidade por todas as causas (RR 0,76; IC95% 0,69-0,83; p < 0,00001); menor mortalidade cardiovascular (RR 0,68; IC95% 0,63-0,74; p < 0,00001) e menor risco de AVCi (RR 0,77; IC95% 0,67-0,87; p < 0,0010; I 2 = 64%).^280^ Portanto, as evidências atuais demonstram fortemente que o controle precoce do ritmo é superior à estratégia do controle da frequência e que, portanto, pacientes com FA, mesmo aqueles assintomáticos em sua apresentação clínica inicial, deveriam receber uma abordagem inicial com o objetivo de manter o RS, e não apenas controlar a FC.

A ablação por cateter como terapia de primeira escolha na FA paroxística também tem sido amplamente estudada, com resultados favoráveis confirmados em recente estudo metanalítico que incluiu 994 pacientes de cinco ensaios clínicos que testaram a ablação por RF (três estudos) e a crioablação (dois estudos) comparadas com as DAA. O tempo médio de seguimento foi de 5 anos, e os autores concluíram que a ablação por cateter é superior às drogas na recorrência da FA sintomática (OR 0,32; IC95% 0,18-0,57; p < 0,001), recorrência de TA (OR 0,36; IC95% 0,25-0,52; p < 0,001) e índice de hospitalização (OR 0,25; IC95% 0,15-0,42; p < 0,001).^361^ Do mesmo modo, outra metanálise, com seis ensaios clínicos, também testando a ablação por RF e a crioablação, obteve resultados semelhantes. O estudo reuniu 1.212 pacientes, sendo 609 pacientes no braço da ablação e 603 no braço das DAAs. O seguimento clínico variou de 1 a 2 anos. A análise agrupada dos resultados demonstrou que a ablação por cateter (RF ou crioenergia) obteve menor recorrência de taquiarritmias atriais (RR 0,63; IC95% 0,55-0,73; p < 0,00001) e de taquiarritmias atriais sintomáticas (RR 0,53; IC95% 0,32-0,87; p = 0,01), sem apresentar diferença estatística para quaisquer eventos adversos (RR 0,93; IC95% 0,68-1,27; p = 0,64) ou eventos adversos cardiovasculares (RR 0,90; IC95% 0,56-1,44; p = 0,65).^377^

A melhora da QV com a ablação já foi comprovada tanto na FA paroxística quanto persistente.^378^ O estudo CABANA tentou demonstrar superioridade da ablação de FA em desfechos duros em relação aos fármacos antiarrítmicos em 2.204 indivíduos. Apesar de também evidenciar melhora sintomática nos pacientes submetidos à ablação, não houve diferença entre as estratégias no desfecho composto morte, acidente vascular encefálico incapacitante, sangramento grave ou parada cardiorrespiratória (HR 0,86; IC95% 0,65-1,15). Este estudo, porém, foi prejudicado pelo alta taxa de "*crossover*", em que 27,5% dos pacientes alocados para o grupo antiarrítmicos acabaram sendo submetidos à ablação.^360,379^ Após o resultado do CABANA, ainda não surgiram evidências robustas de redução de mortalidade ou AVC com a ablação na população geral, sendo a indicação de ablação de FA ainda colocada como manejo sintomático.

Já na população portadora de ICFEr, testada no CASTLE-AF (*Catheter Ablation vs. Standard Conventional Treatment in Patients With LV Dysfunction and AF*) com 363 indivíduos com FEVE < 35%, a ablação se mostrou benéfica tanto no desfecho primário composto por morte por todas as causas ou hospitalização por piora da IC (HR 0,62; IC95% 0,43-0,87) como no desfecho secundário isolado de morte por todas as causas (HR 0,53; IC95% 0,32-0,86).^380^ O CASTLE-AF foi um importante divisor de águas na indicação da ablação como primeira opção nos pacientes portadores de ICFEr, principalmente quando há maior probabilidade de que a taquicardia seja um importante componente da perda de função ventricular (taquicardiomiopatia). Com o controle da FA, haveria a restauração da função ventricular.

Outra população na qual a ablação de FA pode ser benéfica é a de pacientes portadores de FA paroxística relacionada à síndrome taquicardia-bradicardia. Uma análise retrospectiva de 57 pacientes que apresentavam indicação de implante de marca-passo por pausas prolongadas no momento do término dos episódios de FA submetidos à ablação comparados a 43 pacientes submetidos a implante de marca-passo e terapia antiarrítmica demonstrou que, após seguimento médio de 20 meses, 95% do grupo ablação não necessitou mais de implante de dispositivo de estimulação.^381^ Além do controle sintomático, esse grupo de pacientes também poderia se beneficiar da não necessidade de terapia de estimulação.

Dessa forma, as indicações para a ablação podem ser separadas em ablação de FA paroxística e FA persistente, sendo consideradas no grupo persistente tanto as persistentes como as persistentes de longa duração, pois não há dados referentes ao período arbitrário de FA com duração maior de 12 meses.

Em pacientes com FA paroxística e persistente, diversos estudos demonstraram eficácia semelhante de técnicas ponto a ponto utilizando RF e a crioablação na manutenção de RS.^349,382,383^ Em pacientes com TA e *flutter* atípicos associados a FA, a utilização de técnicas ponto a ponto com RF se faz mandatória.

Existe sólida evidência de diferentes taxas de sucesso e complicações quando comparados operadores de baixo (< 50), médio (50-100) e alto volume (> 100 procedimentos ano) de ablação de FA. Operadores de alto volume têm menores taxas de complicação e maiores taxas de sucesso para manutenção de RS. Pacientes que realizam procedimentos em hospitais de alto volume de ablação têm uma menor taxa de mortalidade.^384^

### 8.3. Complicações

Os procedimentos de ablação percutânea para o tratamento de FA foram hoje incorporados à prática rotineira da quase totalidade dos laboratórios de eletrofisiologia, e o domínio das técnicas para realização deste tipo de intervenção é exigência dos programas de formação dos novos especialistas. Isso faz com que as taxas de complicações clinicamente relevantes relativas a esse tipo de ablação sejam hoje realmente baixas. Em uma publicação recente de um grande registro multicêntrico norte-americano envolvendo cerca de 76 mil pacientes tratados por ablação entre os anos de 2016 e 2020, a prevalência de qualquer tipo de complicação foi de 2,5%, sendo que complicações maiores foram observadas em apenas 0,9% dos casos, com mortalidade intra-hospitalar de 0,05%.^385^ Entre os anos de 2000 e 2010, período em que os procedimentos de ablação para tratamento de FA ganhavam credibilidade nos diversos centros, a prevalência global de complicações atingia taxa superior a 5%, com mortalidade intra-hospitalar em torno de 0,5%.^386-388^ Dados de 37 mil ablações para tratamento de FA realizadas na Austrália e Nova Zelândia entre 2008 e 2017 indicaram um incremento de cinco vezes no número de procedimentos realizados e um aumento na complexidade dos pacientes submetidos ao tratamento, porém com uma redução de 30% na taxa de complicações entre os dois extremos de tempo.^389^

Um ponto essencial relacionado às complicações da ablação de FA está na experiência do centro. Um registro europeu incluiu 3.368 pacientes submetidos a ablação em 91 centros, sendo divididos os centros de alto volume quando for maior que 180 procedimentos por ano, volume médio entre 74 e 179 e baixo volume abaixo de 73. Foi observado um aumento na taxa de complicações cardiovasculares e AVC em centros de baixo volume em comparação aos de alto (RR: 1,6; p = 0,039) e médio (RR: 1,2; p = 0,008) e menor taxa de sucesso (p < 0,001).^390^ Uma metanálise que incluiu 14 estudos e 315.120 pacientes indentificou que dois terços dos procedimentos foram realizados em centros de baixo volume. Os centros com volume maior que 50 e 100 procedimentos por ano apresentaram menor taxa de complicações (OR: 0,58; p < 0,001) (OR: 0,62; p < 0,001), respectivamente em comparação aos centros de baixo volume (< 50 procedimentos por ano). A mortalidade também foi menor nos hospitais de maior volume (OR: 0,33; p < 0,001), e uma relação semelhante na incidência de complicações também foi observada com operadores que faziam mais de 50 procedimentos por ano (OR: 0,25; p < 0,001).^391^

As tentativas de tornar a ablação mais eficaz naturalmente aumentam a agressividade das intervenções, com aumento da extensão das ablações dos AE e AD com as proposições de novas técnicas. Isso gerou mudanças de cenários em termos de prevalência e tipos de complicações nos procedimentos de ablação. O isolamento de AAE, por exemplo, proposto por alguns grupos como técnica adicional ao isolamento das veias pulmonares, agregou ao procedimento uma importante variável de alteração mecânica na contração do AAE com aumento da probabilidade pós-operatória de eventos cardioembólicos, tornando necessárias medidas agressivas de proteção antitrombótica indo desde o uso sistemático e permanente de terapia anticoagulante até a própria oclusão do AAE por dispositivo oclusor.^384^ O direcionamento de lesões para a parede posterior do AE nos procedimentos em "*box*" agregou a maior probabilidade da ocorrência de lesões térmicas esofágicas, exigindo adoção de medidas intraoperatórias e pós-operatórias para prevenção, diagnóstico precoce e tratamento.^392^

A crioablação por cateter-balão dos antros das veias pulmonares introduzida na prática clínica em 2007^393,394^ trouxe o benefício da simplificação das intervenções, proporcionando um isolamento venoso com uma única aplicação, sendo na prática clínica atual uma modalidade de ablação utilizada como primeira alternativa terapêutica por muitos laboratórios de eletrofisiologia, especialmente na FA paroxística. A efetividade é similar à observada com a ablação por RF, com procedimentos mais rápidos, porém sem agregar vantagens em termos de redução de complicações. Em uma grande metanálise de 15 estudos controlados comparando as duas tecnologias, envolvendo cerca de 2.700 pacientes, não se conseguiu demonstrar diferenças significativas entre ambas no que diz respeito à ocorrência de complicações.^395^

Neste documento, são abordadas as principais complicações observadas nos procedimentos de ablação percutânea para tratamento de FA, com exploração dos critérios de diagnóstico e medidas preventivas e terapêuticas. As informações são resumidas na Tabela 22.

**Tabela 22:** Principais complicações em procedimento de ablação percutânea para tratamento de fibrilação atrial pelas técnicas disponíveis em uso clínico

Complicação	Incidência	Diagnóstico	Tratamento	Prevenção	Prognóstico
**Lesão térmica esofágica**	Até 60%	Fortuito (subclínico)	Dieta fria líquido-pastosa	Redução de potência em parede posterior	Bom
		Manifestações clínicas	Inibidor da bomba de prótons	Limitação de força de contato em parede posterior	
		Endoscopia digestiva alta	Sucralfato	Desvio esofágico	
				Desvio de linhas de ablação	
**Fístula atrioesofágica**	0,01 a 0,03%	Manifestações clínicas	Stent esofágico	Mesmas da lesão térmica	Ruim
		Tomografia computadorizada	Cirurgia		Mortalidade > 50%
		Ressonância magnética			
**Alterações da motilidade gastroesofágica**	Até 74%	Manifestações clínicas	Dieta fria líquido-pastosa em baixo volume	Mesmas da lesão térmica	Bom
		Endoscopia digestiva alta	Metoclopramida		
		Tomografia computadorizada	Suporte nutricional nas formas graves		
			Cirurgia		
**Tamponamento cardíaco**	< 1%	Manifestações clínico-hemodinâmicas	Drenagem por punção sub-xifoide	Apoio de ecocardiografia intracardíaca	Bom
		Fluoroscopia	Cirurgia		
		Ecocardiografia intracardíaca			
		Ecocardiografia transtorácica			
**Acidente vascular cerebral isquêmico**	< 1% (forma clínica)	Manifestações clínicas	Terapia trombolítica	Pesquisa de trombos por método de imagem	Dependente da extensão
		Tomografia computadorizada	Angioplastia	Anticoagulação ininterrupta	
		Ressonância magnética		Anticoagulação operatória precoce com heparina	
		Angiografia cerebral		Aspiração e lavagem cuidadosa de bainhas	
Embolia aérea	< 1%	Suspeita por imagem transoperatória	Medidas de suporte respiratório e hemodinâmico	Manipulação cuidadosa de bainhas e cateteres com luz	Dependente da extensão
		Manifestações clínicas	Posição inclinada para baixo de cabeça		
		Tomografia computadorizada cerebral	Oxigenação hiperbárica		
		Ressonância magnética cerebral			
**Estenose de veias pulmonares**	< 1% (forma clínica)	Manifestações clínicas	Angioplastia	Evitar lesões no interior das veias pulmonares	Bom
		Tomografia computadorizada	Stent		
		Ressonância magnética			
Paralisia frênica permanente	< 1%	Observação radiológica operatória	Medidas de suporte	Estimulação frênica operatória	Dependente da magnitude de acometimento ventilatório
		Manifestações clínicas		Monitoramento de atividade elétrica do nervo frênico	
**Complicação vascular**	0,2 a 1,5%	Manifestações clínicas	Conservador	Técnica adequada de acesso vascular	Bom
		Ultrassonografia vascular	Embolização	Punção guiada por ultrassom	
			Cirurgia		

### 8.4. Principais Complicações

#### 8.4.1. Lesão Térmica Esofágica

A lesão térmica esofágica é uma frequente complicação dos procedimentos de ablação percutânea por corrente de RF ou crioablação, sendo observada em até 60% dos pacientes. A quase totalidade dessas lesões é transitória, com cicatrização completa nos primeiros dias em seguida ao procedimento. Na maioria das vezes, se restringe a eritema e úlceras superficiais, podendo também ocorrer sob a forma de úlceras profundas de cicatrização mais lenta.^396^ As úlceras parecem ser menos comuns pela crioablação do que pela RF.^397^ A grande preocupação na sua ocorrência advém do fato de que elas presumivelmente são as lesões precursoras da fístula atrioesofágica.^398^ Normalmente são assintomáticas, descobertas em endoscopia digestiva realizada no pós-operatório, adotada por alguns grupos como prática rotineira de segurança. Diversas medidas preventivas têm sido empregadas, não havendo consenso sobre sua eficácia,^399^ como redução da potência e da força de contato nas lesões da parede posterior, monitoramento da temperatura esofágica por termômetro pré-moldado multipolar com interrupção das lesões frente a eventuais elevações, desvio mecânico esofágico por sonda de ecocardiografia ou por dispositivos específicos,^400-404^ modificações na dinâmica de produção das lesões visando evitar aquecimento aditivo^405^ e desvio de linha de ablação baseado na definição pré-operatória da posição do esôfago por método de imagem.^406^ A realização de endoscopia digestiva alta rotineira nas primeiras 24 a 48 horas em seguida à ablação é utilizada como rotina por alguns grupos.^407^ O intuito nesses casos é o diagnóstico precoce, a vigilância e o tratamento, com vistas a prevenir evolução das lesões para uma eventual fístula atrioesofágica. Tal prática não é consensual, visto que a quase totalidade dessas lesões, quando presentes, apresenta resolução espontânea precoce. A utilização de inibidor de bomba de prótons é uma prática rotineira no pós-operatório, com o objetivo de reduzir a secreção cloridro-péptica do estômago, evitando agressão esofágica na eventualidade da presença de refluxo gastroesofágico.^319^ Existem fortes indícios de que a lesão térmica esofágica promova dano ao plexo vagal anterior do esôfago, contribuindo para exacerbação de refluxo preexistente ou para o surgimento de refluxo inexistente antes da ablação. Entretanto, não há evidências consistentes de que o uso rotineiro desses fármacos promova redução na chance de ocorrência de fístula atrioesofágica. O tratamento da lesão térmica esofágica é intuitivo.^408^ Habitualmente, é instituída dieta líquido-pastosa fria, com o intuito de evitar agressão mecânica ou térmica adicional sobre uma lesão já estabelecida. A utilização de inibidor de bomba de prótons é rotineira, como comentado anteriormente. Na presença de úlcera diagnosticada, muitos grupos utilizam sucralfato sob a forma de flaconetes ou comprimidos mastigáveis visando à formação de película protetora sobre a erosão, composta por complexo formado pelo fármaco, em combinação com o exsudato da própria erosão.

#### 8.4.2. Fístula Atrioesofágica ou Esôfago-pericárdica

A fístula é a complicação mais temida nos procedimentos de ablação baseada em lesões térmicas.^409^ É rara, ocorrendo em proporção de 1 para cada 1.000 a 4.000 pacientes.^410^ É uma complicação dramática, que frequentemente cursa com embolia aérea, mediastinite, pericardite e septicemia. A mortalidade é elevada, com taxas superiores a 50% em pacientes tratados por cirurgia ou endoscopia e em torno de 90% em pacientes não tratados.^411^ Normalmente tem manifestação tardia, geralmente 2 a 3 semanas após a ablação, e os sintomas mais comuns são febre, queda do estado geral, dor torácica e manifestações sistêmicas embólicas, em particular o AVC por embolia séptica. O diagnóstico é realizado por tomografia computadorizada ou ressonância magnética. A endoscopia digestiva alta deve ser evitada diante da suspeita clínica pela possibilidade de precipitação de embolia gasosa. O tratamento deve ser imediato. Existem evidências de que a conduta conservadora está associada com piores desfechos. Não é consensual se a cirurgia é superior ao implante de *stent* esofágico; contudo, o tratamento cirúrgico parece ter melhores resultados.^412^ Deve ser envolvida equipe multidisciplinar nas tomadas de decisão. O tratamento cirúrgico deve ser compartilhado por equipes de cirurgia torácica e cardíaca. Algumas técnicas endoscópicas para fechamento da fístula têm sido usadas recentemente, principalmente quando a fístula ainda não está estabelecida.^413^

#### 8.4.3. Alterações da Motilidade Gastroesofágica

Como já comentado anteriormente, a ação térmica das ablações na parede posterior do AE pode causar lesão ao plexo vagal anterior do esôfago, implicando em alterações de motilidade esofágica e gástrica, traduzidas clinicamente por refluxo gastroesfágico e gastroparesia. Tais alterações são normalmente transitórias, relativamente comuns e frequentemente passam despercebidas.^414,415^ Uma anamnese dirigida no pós-operatório frequentemente identifica sintomas como sensação de plenitude gástrica, náuseas e eructações, que se confundem com os sintomas naturais de recuperação pós-operatória. A gastroparesia severa não é comum, porém pode ser limitante e de tratamento difícil.^416^ Nas formas mais leves sintomáticas, a utilização de dieta líquido-pastosa de fácil digestão administrada em volumes pequenos e intervalos curtos e a utilização de metoclopramida e bromoprida são suficientes. Nas formas severas, o envolvimento de equipe multiprofissional faz-se necessário, com linhas de conduta mais agressivas que podem envolver às vezes a necessidade de suporte nutricional por linhas alternativas, restabelecimento do peristaltismo normal e até marca-passo gástrico ou tratamento cirúrgico.

#### 8.4.4. Tamponamento Cardíaco

As perfurações miocárdicas com consequente hemopericárdio e tamponamento cardíaco permanecem sendo uma das principais complicações dos procedimentos de eletrofisiologia e da ablação para tratamento de FA. Contudo, diante dos domínios de técnica de punção transeptal e de instrumentação do AE, da utilização da ecocardiografia intracardíaca e da utilização dos cateteres terapêuticos com monitoramento de força de contato, a prevalência dessa complicação declinou substancialmente, hoje para valores em torno de 0,4%.^385^ A redução da movimentação da silhueta cardíaca na fluoroscopia, a própria demonstração direta de derrame pericárdico por ecocardiografia intracardíaca ou a presença de instabilidade hemodinâmica permitem o seu diagnóstico. O acesso facilitado à ecocardiografia transtorácica na ausência de ecocardiografia intracardíaca é uma exigência de segurança em um laboratório de eletrofisiologia. O tratamento consiste em obtenção de drenagem percutânea por punção sub-xifoide com aspiração até formação de pressão negativa no saco pericárdico, o que permite controle da situação na quase totalidade dos casos.^417^ A reversão do efeito da heparina deve ser realizada com sulfato de protamina após o esvaziamento do derrame pericárdico. Em pacientes sob uso de anticoagulantes de ação direta, existe a possibilidade de inativação do seu efeito com idarucizumabe naqueles pacientes sob uso de dabigatrana ou adexanet-alfa nos que estão sob uso de inibidores de fator XA.^418,419^ Raramente frente à persistência de sangramento torna-se necessária intervenção cirúrgica para rafia da perfuração. Raramente casos de tamponamento tardios podem ser identificados após ablação de FA, podendo os pacientes se apresentarem com sintomas inespecíficos incluindo dispneia e IC direita.^420^

#### 8.4.5. Acidente Tromboembólico Sistêmico

Os acidentes embólicos sistêmicos manifestos sob a forma de AVC ou embolias sistêmicas têm uma prevalência baixa, com incidência inferior a 0,2% de acordo com dados recentes.^385^ Contudo, as chamadas embolias cerebrais assintomáticas são frequentes e têm sido demonstradas em até 87% dos pacientes submetidos a ablação para tratamento de FA.^421-423^ Esses microinfartos regridem em sua maioria algumas semanas após sua detecção, não tendo sido associados a alterações neurocognitivas. Os acidentes embólicos estão relacionados potencialmente à formação de trombos sobre guias de punção transeptal, dentro ou fora das bainhas transeptais, na superfície de cateteres, ao fenômeno de formação de crosta na ponta do cateter de ablação durante aplicação de RF, à fragmentação de trombo preexistente dentro do AAE, ao "atordoamento" mecânico atrial em seguida à cardioversão elétrica realizada no procedimento, à presença de trombofilias ou a combinação de fatores. As interrupções da terapia anticoagulante com utilização de ponte com heparina de baixo peso molecular parecem estar relacionadas a uma maior chance de ocorrência de eventos embólicos, de tal modo que hoje a terapia ininterrupta com anticoagulantes de ação direta é recomendada, não havendo impacto em termos do aumento da prevalência de complicações hemorrágicas.^424^ A investigação pré-operatória por método de imagem de trombos em átrio ou AAE, envolvendo a ecocardiografia transesofágica e a tomografia computadorizada cardíaca,^425^ é sugerida logo antes do procedimento em pacientes em uso de anticoagulante por, pelo menos, 3 semanas, especialmente em pacientes com CHA2DS2-VA ≥ 2, e recomendada nas 48 horas que antecedem o procedimento naqueles pacientes sem uso de anticoagulante ou em quem a anticoagulação tenha sido instituída há menos de 3 semanas.^319^ Pode ser utilizada também a EIC no momento do procedimento em pacientes em ritmo sinusal e/ou com efetiva anticoagulação por 3 semanas para exclusão de trombos.^334^

A anticoagulação sistêmica transoperatória com heparina deve ser iniciada imediatamente antes ou em seguida à punção transeptal e mantida de forma ininterrupta durante a intervenção para manter um teste de coagulação ativado entre 300 e 350 segundos. As bainhas transeptais devem ser lavadas com solução fisiológica heparinizada após sua introdução no AE; entretanto, a irrigação contínua com solução heparinizada contínua é adotada por muitos grupos, mas não tem uma indicação consensual. Nas ablações por RF, o emprego de cateteres terapêuticos com irrigação aberta é sempre recomendado, pela minimização da formação de crostas em ponta. O tratamento dos acidentes TE sistêmicos naturalmente deve envolver equipes de radiologia intervencionista, inclusive na área neurológica. Isso significa que procedimentos desse porte devem sempre ser realizados em instituições que ofereçam retaguarda multidisciplinar adequada. O AVC significativo deve prontamente ser abordado por intervenção neurovascular, com terapia trombolítica, aspiração e angioplastia. As embolias cerebrais assintomáticas são de diagnóstico fortuito e normalmente não são abordadas.

#### 8.4.6. Embolia Aérea

A embolia aérea em procedimentos de ablação para tratamento de FA é uma consequência da entrada de ar nos elementos de comunicação entre o meio exterior e o AE, em especial as bainhas transeptais. A importância clínica do problema está inteiramente ligada ao montante de ar introduzido no sistema. Uma manifestação comum da embolia aérea é a isquemia e a lesão da parede inferior do VE. A posição mais superior do óstio da coronária direita faz dele uma porta de entrada preferencial para bolhas. Essas alterações costumam ser transitórias e podem cursar com bloqueio AV total necessitando estimulação cardíaca artificial temporária. Embolias maciças cursam com colapso hemodinâmico, hipoxemia e lesões cerebrais irreversíveis.^426^ O tratamento envolve maximização da oxigenação, hidratação, tratamento do choque e, quando possível, aspiração do ar infundido.^427^ A inclinação inferior da cabeça é uma manobra benéfica. O tratamento com oxigenação hiperbárica pode reverter a condição. A prevenção da complicação envolve um cuidado minucioso com a remoção de bolhas em bainhas, equipos e cateteres com luz e maior atenção quando se utiliza múltiplas vias de irrigação. Um cuidado especial deve ser dado na introdução de balão para crioablação.

#### 8.4.7. Estenose de Veias Pulmonares

A estenose de veias pulmonares é uma complicação tanto das ablações por RF^428^ como das crioablações.^429,430^ Estenoses severas, com redução de diâmetro superior a 70%, clinicamente relevantes, são atualmente raras em procedimentos de isolamento das veias pulmonares, com incidência inferior a 1%.^431^ Nas primeiras ablações por RF, quando se realizavam isolamentos mais profundos, direcionados para os óstios das veias pulmonares, a ocorrência dessa complicação era frequente; entretanto, hoje em dia, com o uso de estratégias mais antrais, essa complicação se tornou rara.^318,431,432^ Estenoses graves podem cursar com dispneia, hemoptise, tosse, pneumonia e dor torácica, com ocorrência tardia, geralmente semanas após o procedimento.

A suspeita clínica sempre deve ocorrer frente à ocorrência desse tipo de sintomatologia. A investigação deve ser realizada mediante angiotomografia ou ressonância magnética.^433^ O tipo de tratamento depende da importância clínica e da magnitude da estenose observada. A angioplastia é o tratamento de eleição para as formas graves, e o implante de *stent* sempre deve ser considerado, implicando em menor chance de reestenose.^434^

#### 8.4.8. Paralisia Frênica

A paralisia frênica permanente hoje é de ocorrência quase exclusiva dos procedimentos de crioablação.^435,436^ O mecanismo está associado à proximidade anatômica do nervo frênico direito com o antro das veias pulmonares direitas. A sua ocorrência em forma permanente é rara, com incidência em torno de 0,3%.^394,436^ A manobra usual para evitar essa complicação é realizar a aplicação sob estimulação frênica, utilizando cateter posicionada na veia cava superior. Utiliza-se estimulação local com energia superior a 10 mA e observa-se a efetividade da estimulação pela observação visual ou palpatória do movimento involuntário diafragmático a cada pulso de marca-passo. Os pacientes devem estar sem efeito do bloqueador neuromuscular nas aplicações nas veias direitas. A redução ou perda de movimento significa esfriamento do frênico e deve implicar em interrupção imediata da aplicação de crioenergia. Existem técnicas de monitoramento direto da atividade elétrica do nervo frênico que podem ser empregadas, porém de uso pouco comum na prática diária considerando a dificuldade técnica para obtenção e ausência de vantagens significativas sobre o método de avaliação indireta. A paralisia frênica usualmente é uma complicação transitória, com resolução completa nos primeiros 3 meses em seguida à ablação. Não existe tratamento específico para a paralisia frênica permanente.

#### 8.4.9. Complicação Vascular

As complicações vasculares são inerentes às punções para acesso venoso. A maioria dos laboratórios de eletrofisiologia utiliza acesso exclusivo venofemoral para realização dos procedimentos. As principais complicações são hematoma inguinal, fístula AV e pseudoaneurisma de artéria femoral.^318,387,437^ Tais complicações usualmente são consequentes a punções arteriais inadvertidas e hemostasia inadequada ao final do procedimento. As manifestações clínicas envolvem tumoração sobre a área de punção e dor local de graus variáveis. A suspeita clínica de complicação maior sempre deve existir na presença de dor importante no pós-operatório, devendo-se realizar ultrassom vascular quando existe suspeita de complicação vascular. Uma situação extrema e consequentemente mais grave corresponde ao hematoma retroperitoneal, consequente a punções mais altas, e o pseudoaneurisma associado a punção arterial acidental. A conduta terapêutica exige o envolvimento da radiologia intervencionista e vai desde uma conduta conservadora à embolização ou ao tratamento cirúrgico. O uso de punção guiada por ultrassom deve ser estimulado por ser capaz de reduzir a incidência desse tipo de complicação.^438^

#### 8.4.10. Outras Complicações

Algumas complicações mais raras têm sido descritas em situação de pós-operatório de ablação para tratamento de FA. A síndrome do AE não complacente originalmente descrita após cirurgia da valva mitral tem sido observada em pós-ablação por RF de FA.^438,440^ Caracteriza-se clinicamente por um quadro de IC por aumento das pressões de enchimento do AE, cursando com dispneia e hipertensão arterial pulmonar, na ausência de disfunção ventricular esquerda, respondendo bem a terapia diurética e uso de anti-inflamatórios. Casos de dispneia leve e tosse pós-ablação não são raros, sendo em muitos centros utilizado de rotina diurético nos primeiros dias após a ablação. A síndrome de Takotsubo também tem sido raramente descrita em pacientes submetidos a ablação percutânea para tratamento de FA, em uma incidência estimada em 0,04%.^441^

### 8.5. Crioablação

Diversos estudos randomizados e observacionais relatam eficácia e segurança similares entre as ablações realizadas com RF e crioenergia.^349,436,442-448^ Alguns estudos demonstraram redução de hospitalização e menor taxa de complicações com a crioablação.^449^

O principal mecanismo destrutivo da crioablação é a lise celular causada pela formação de gelo no meio intra e extracelular, acarretando morte celular por necrose e por apoptose e ocasionando uma menor resposta inflamatória e, consequentemente, menor edema, um dos fatores apontados como responsáveis pela reconexão das veias. Além disso, a criotermia não promove desnaturação de proteínas, preservando o colágeno e a elastina do tecido conjuntivo e, consequentemente, preservando a matriz extracelular e minimizando o risco de formação de trombos, de estenose das veias e de lesão no esôfago.^450^

Os parâmetros importantes de injúria tecidual relacionada à crioterapia são a velocidade de resfriamento, a duração do resfriamento, o tempo de reaquecimento e a repetição do ciclo resfriamento-aquecimento-resfriamento.^451^ Alguns parâmetros aferidos durante o procedimento, como o contato adequado do balão no antro das veias pulmonares antes do início do resfriamento, o registro dos potenciais das veias pulmonares para determinação do tempo do início do resfriamento até o isolamento elétrico da veia (TTI) e o tempo de reaquecimento tecidual, são importantes indicadores para avaliação de lesão transmural eficaz.^394,452^

#### 8.5.1. Técnica de Crioablação de Fibrilação Atrial

O contato do criobalão com o tecido cardíaco é um importante critério para formação de lesão e isolamento da veia pulmonar (IVP). A oclusão da veia pulmonar é fundamental para o uso bem-sucedido do criobalão. A confirmação de uma oclusão adequada pode ser feita através da observação na fluoroscopia da retenção de contraste entregue através do ponta do balão na veia pulmonar ou através da observação de vazamento pelo ecocardiograma intracardíaco ou transesofágico.^453,454^

O IVP pode ser monitorado em tempo real usando o cateter de mapeamento. O TTI é o tempo decorrido desde o início da crioablação até a observação do isolamento da veia. Diversos protocolos atuais incorporam a avaliação do TTI durante o procedimento de crioablação.^455-457^ Estudos que avaliaram TTI e recorrência de FA identificaram uma tendência maior de recorrência com tempos mais longos para atingir o isolamento da veia.^35-39^ O isolamento, quando atingido nos primeiros 60 segundos da aplicação da crioterapia, está associado a uma menor incidência de reconexão das veias pulmonares e manutenção de longo prazo,^451,458,459^ com uma tendência à ausência de reconexão com isolamento alcançado em até 40 segundos. O TTI e a dosagem são parâmetros importantes associados à eficácia da crioablação; entretanto, congelamento de bônus é apropriado quando o TTI não é visualizado ou é maior ou igual a 60 segundos. Recomenda-se que seja confirmado o IVP testando o seu bloqueio de entrada e saída.

A temperatura do balão é um parâmetro importante que deve ser monitorado durante o procedimento, devendo-se ficar atento a temperaturas abaixo de −55 °C durante tempo prolongado e evitando-se temperaturas abaixo de −60 °C.^451^

Recomenda-se a aferição do tempo entre o final da crioterapia até a temperatura do balão alcançar 0 °C, com a intenção de avaliar a qualidade da lesão e consequentemente a durabilidade do IVP.

Estudos iniciais de crioablação utilizaram aplicações de 240 a 300 segundos de duração, seguidos de uma aplicação adicional de bônus.^460^ Atualmente, o tempo de aplicação foi reduzido para 180 segundos e foi eliminada a obrigatoriedade de uma aplicação adicional de bônus, sem alterar a eficácia do procedimento a longo prazo. ^461,462^

### 8.6. Ablação por Cateter para Fibrilação Atrial em Pacientes com Insuficiência Cardíaca

A FA e a IC são condições clínicas frequentemente coexistentes; pelo menos 50% dos pacientes com IC apresentam FA e 30% dos pacientes com FA desenvolvem IC. A FC elevada associada à perda da sístole atrial e à irregularidade temporal da sístole ventricular são alterações fisiopatológicas presentes nos pacientes com FA que podem induzir disfunção ventricular ou agravar a ICFEr preexistente. Paralelamente, as alterações hemodinâmicas e hormonais envolvidas na ICFEr criam condições favoráveis para a ocorrência e manutenção da FA.^106,463^

Até recentemente, não havia evidência clínica de que a restauração e manutenção do RS fosse benéfica para esse perfil de pacientes,^277,463^ contudo, entre 2008 e 2014, quatro estudos randomizados envolvendo 224 pacientes demonstraram efeitos hemodinâmicos e clínicos benéficos possivelmente relacionados com a ablação.^304,465-467^ Em três desses estudos, a FA era persistente e envolvia pacientes com IC classe funcional entre II-III com fração de ejeção média de 26%. Os pacientes eram na sua maioria homens (89%), com idades variando entre 57 e 63 anos, predominando a cardiopatia isquêmica como etiologia da ICFEr. A ablação da FA melhorou significantemente a QV, o consumo de oxigênio e a distância de caminhada de 6 minutos em comparação com os pacientes em controle clínico. No subgrupo de FA persistente, houve aumento significativo da FEVE nos pacientes submetidos a ablação de FA, e os efeitos adversos não foram significativamente diferentes.^465^

O estudo CAMERA-MRI (*Catheter Ablation Versus Medical Rate Control in Atrial Fibrillation and Systolic Dysfunction*) foi um ensaio clínico randomizado multicêntrico que envolveu pacientes com FA persistente e cardiomiopatia idiopática (FEVE ≤ 45%). Todos os pacientes foram submetidos ao controle da FC e à ressonância magnética cardíaca (RMC) para avaliar a FEVE e o realce tardio ventricular, a fim de avaliar extensão da fibrose, antes da randomização para ablação por cateter ou tratamento clínico com controle da FC. Entre 2013 e 2016, 66 pacientes foram randomizados, 33 para cada braço. A FEVE absoluta melhorou em 18±13% no grupo ablação em comparação com 4,4±13% no grupo clínico (p < 0,0001) com normalização da fração de ejeção (≥ 50%) em 58% versus 9%, respectivamente (p = 0,0002). Naqueles submetidos à ablação por cateter, a ausência de realce tardio previu melhor a recuperação da FEVE em 6 meses (73% vs. 29%; p = 0,0093).^468^

O AATAC (*Ablation vs Amiodarone for Treatment of AFib in Patients With CHF and an ICD*) foi o primeiro estudo multicêntrico e randomizado a apontar possível benefício da ablação na redução de mortalidade. Foram incluídos 203 pacientes com FA e ICFEr (NYHA II a III e FEVE < 40%), portadores de CDI de câmara dupla ou terapia de ressincronização cardíaca. Os pacientes foram randomizados para ablação por cateter (n = 102) ou tratamento médico com amiodarona (n = 101). Durante um seguimento mínimo de 24 meses, a taxa livre de recorrência de FA foi de 70% nos pacientes do grupo ablação, em comparação com 34% no grupo amiodarona (p < 0,001). A taxa de internação foi de 31% no grupo ablação e 57% no grupo amiodarona (p < 0,001). De forma importante, a mortalidade (objetivo secundário do estudo) foi significativamente menor no grupo de ablação por cateter (8% vs. 18%, p = 0,03).^469^

No estudo CASTLE-AF, Marrouche et al.^380^ confirmaram as observações do estudo AATAC, demostrando que a ablação por cateter da FA reduz significativamente a mortalidade de pacientes com ICFEr, em comparação com o controle da FC. Nesse estudo, 263 pacientes com FA paroxística ou persistente sintomática foram randomizados, 179 pacientes para ablação por cateter da FA e 183 para tratamento clínico, com controle da frequência ou do ritmo. Todos os pacientes estavam em IC classe II, III ou IV da NYHA, FEVE de 35% ou menos e desfibrilador implantado. O desfecho primário, a combinação de morte por qualquer causa e hospitalização por piora da IC, foi obtido após um seguimento médio de 37,8 meses, favorecendo a ablação por cateter em comparação com a terapia médica. No grupo ablação, 63% dos pacientes estavam em ritmo sinusal aos 60 meses contra 22% no grupo de terapia medicamentosa. O desfecho primário composto ocorreu em 51 (28,5%) pacientes no grupo de ablação e em 82 (44,6%) pacientes no grupo de terapia médica (HR = 0,62; p = 0,007). Houve uma redução significativa da mortalidade por todas as causas no grupo de ablação (13,4% vs. 25,0%, HR = 0,53, p = 0,01) e por causas cardiovasculares (11,2% vs. 22,3%; FC = 0,49; p = 0,009). Além disso, os pacientes submetidos à ablação por cateter apresentaram redução da taxa de internação por piora da IC (20,7%) em relação ao tratamento médico (35,9%, HR = 0,56, p = 0,004). Adicionalmente, a ablação por cateter reduziu a carga de FA, aumentou a distância percorrida em 6 minutos e melhorou a FEVE (8%).^380^ A principal limitação desse estudo foi a alta seleção dos pacientes incluídos, que representaram 10% dos pacientes avaliados.

A tentativa de generalização dos resultados do CASTLE-AF em mundo real confirmou que menos de 10% da população geral apresentava características semelhantes àquelas do estudo clínico, e a redução de risco da ablação para morte e internação hospitalar foi inferior à observada previamente (RR de 18% comparado com 40% de RR no CASTLE-AF).^470^

No estudo CASTLE-HTx com IC terminal, em que os pacientes apresentavam fração de ejeção média de 29±6% no grupo ablação e 25±6%no grupo medicamentoso, em um seguimento mediano de 18 meses, o desfecho primário composto que incluía morte, implante de assistência circulatória ou transplante cardíaco de urgência foi reduzido no grupo de pacientes que foram submetidos ablação de FA (8% no grupo ablação e 30% no grupo tratamento clínico otimizado). Ocorreu um incremento na fração de ejeção de 7,8±7,6% no grupo ablação e uma redução na carga de FA de 31,4±33,3%.^471^

Adicionalmente, o estudo AMICA (*Atrial Fibrillation Management in Congestive Heart Failure With Ablation*) incluiu 140 pacientes (65±8 anos, 90% homens com FA persistente/de longa duração e FEVE ≤ 35%) alocados aleatoriamente para ablação por cateter ou tratamento clínico. Apesar de os pacientes do grupo ablação permanecerem mais tempo em RS do que os pacientes do grupo clínico, o desfecho primário do estudo (aumento absoluto da FEVE) foi observado em 8,8% no grupo de ablação e em 7,3% no grupo clínico, sem diferença estatística ao longo de 1 ano. Os desfechos secundários incluíram teste de caminhada de 6 minutos, QV e peptídeo natriurético tipo B N-terminal (NT-proBNP) que também não foram significativos entre os grupos de tratamento.^472^ Vale destacar que os pacientes do estudo AMICA aparentemente apresentavam IC mais avançada, pois apresentavam a média da função ventricular ao ecocardiograma mais deprimida em relação ao estudo CASTLE-AF (27% vs. 32%), maior número de pacientes em classe funcional III-IV (100% vs. 70%) e padrão de FA persistente ou prolongada mais frequente (60% vs. 31%), embora a dimensão do AE não fosse diferente entre as duas populações. Esses dados podem sugerir que o efeito hemodinâmico da restauração do RS pode ser menos evidente em pacientes com ICFEr mais avançada; entretanto, o aumento da FEVE em 1 ano não foi diferente entre o AMICA (8,8%), o CASTLE-AF (7,0%) e o AATAC (9,6%). A ausência de diferença entre os resultados da ablação e tratamento clínico no AMICA na verdade se deveu a ótima recuperação da fração de ejeção no grupo clínico do AMICA (7,3%) em comparação ao CASTLE-AF e AATAC (2,0% e 4,2%), respectivamente, que poderia ser explicado pelo maior número de pacientes com ressincronizadores no estudo AMICA. É interessante que as análises de subgrupo do desfecho primário em CASTLE-AF sugerem que os benefícios da ablação ocorreram nos pacientes com sintomas de IC classe funcional II da NYHA, bem como pacientes com FEVE > 25%, mas não foram evidentes naqueles com classe funcional III e fração de ejeção < 25%. Provavelmente, o fator mais importante para a resposta clínica favorável da restauração e manutenção do ritmo sinusal em pacientes com ICFEr, seja ela feita pelo manejo clínico ou pela ablação, esteja relacionado ao grau de taquicardiomiopatia induzido pela FA.

Pacientes com IC têm potencial maior de complicações durante e após a ablação e preferencialmente devem ser tratados em hospitais de médio e alto volume de ablações e por profissionais com médio e alto volume de ablação. A utilização de um *Heart Team* incluindo médico especialista em IC deve ser recomendada. As recomendações de ablação de FA em pacientes com IC estão apresentadas no Quadro 16.

**Quadro 16:** Recomendações de ablação de fibrilação atrial (FA) em pacientes com insuficiência cardíaca (IC)

Recomendações	Classe de recomendação	Nível de evidência
A utilização de um Heart Team incluindo médico com experiência em IC deve ser recomendada nos pacientes com IC em avaliação para ablação de FA	**I**	**B**
Ablação de FA é recomendada para pacientes com ICFEr com alta probabilidade de taquicardiomiopatia induzida pela FA	**I**	**B**
Ablação de FA é recomendada para pacientes com ICFEr e FA para diminuição de internação hospitalar e redução da mortalidade	**IIa**	**B**
Ablação de FA é recomendada para pacientes com FA e FC controlada (FC em repouso < 80 bpm) e IC classe funcional IV ou FE < 20%	**IIb**	**B**

27 ICFEr: insuficiência cardíaca com fração de ejeção reduzida; FC: frequência cardíaca.

## 9. Tratamento Cirúrgico da Fibrilação Atrial

A cirurgia de Cox-Maze, descrita em 1987 pelo Dr. James Cox, persistiu durante anos como a única forma de tratamento invasivo da FA.^473^ Com o objetivo de interromper os circuitos de macro reentrada atrial, uma sequência específica de "corte-sutura" era realizada criando cicatrizes transmurais e formando barreiras à condução do estímulo nos átrios que, desta forma, interrompiam a manutenção da FA. A associação da ressecção do AAE resultava em redução também da ocorrência de eventos tromboembólicos (TE).^473-475^

Sua versão final, a Cox-Maze III, tornou-se o tratamento cirúrgico padrão da FA ao apresentar taxas de sucesso na manutenção do RS a longo prazo, variando entre 73 e 97%.^476^ Entretanto, a necessidade de toracotomia e a alta complexidade do procedimento com considerável índice de complicações e morbidades relacionadas limitaram e restringiram seu uso aos pacientes com FA refratária ao tratamento clínico e que apresentavam indicação para toracotomia pela necessidade de outras cirurgias cardíacas, em geral, para tratamento de valvopatia mitral.^477^

O desenvolvimento tecnológico e o acesso a novas ferramentas invasivas, utilizando fontes de energia como a criotermia e a RF, permitiu a substituição do tradicional "corte-sutura" pelas ablações por cateter que ainda, que, adicionadas à utilização de pinças de RF bipolar, trouxeram um grande avanço na abordagem da FA,^478^ desta vez, através de acessos menos invasivos e com coração "batendo", sem a necessidade de circulação extracorpórea (CEC) e com produção de lesões lineares transmurais pelo epicárdio, tornou-se padrão-ouro no tratamento cirúrgico da FA.^479^

Em 2002, Gaynor et al. ampliaram significativamente as indicações do tratamento cirúrgico da FA ao redor do mundo ao descrever a cirurgia de Cox-Maze IV que consistia na combinação de ablação por RF bipolar e crioenergia replicando as incisões da cirurgia de Cox-Maze III.^480^ A técnica minimamente invasiva utilizando pinças de RF bipolar tornou o tratamento que era restrito aos casos combinados com outras cirurgias cardíacas factível para o tratamento cirúrgico isolado da FA. A evidente expansão do tratamento por essa técnica foi demonstrada pela publicação dos registros da Society of Thoracic Surgeons, mostrando um aumento de 3.987 procedimentos realizados em 2004 para 12.737 no ano seguinte.^481^ As evoluções das técnicas cirúrgicas são apresentadas na Tabela 23.

**Tabela 23:** Modificações da técnica de Cox-Maze

Procedimento	Modificação da versão anterior	Limitações do procedimento
**Cox-Maze I**	NA	Incapacidade de produzir taquicardia sinusal apropriada
		Disfunção atrial esquerda pós-operatória
**Cox-Maze II**	Átrio esquerdo: atriotomia transversa através da cúpula do átrio esquerdo movida posteriormente	Condução intra-atrial prolongada
		Deve transecionar completamente a VCS para obter exposição atrial esquerda
	Atrial direito: eliminação da VCS para lesão atrial direita	
**Cox-Maze III**	Átrio direito: colocação da incisão septal posterior ao orifício da VCS	Tempo prolongado de CEC e dificuldade técnica
**Cox-Maze IV**	Combinação de ablação por RF bipolar e crioablação	Necessidade de CEC
	Átrio esquerdo: lesão em caixa ao redor do átrio esquerdo posterior	

VCS: veia cava superior; CEC: circulação extracorpórea; RF: radiofrequência.

Entretanto, algumas limitações técnicas na abordagem de regiões críticas no tratamento da FA e do FLA persistiam, tornando necessária em algumas situações a ablação híbrida, que associa a cirurgia minimamente invasiva e a ablação por cateter em um único momento ou sequencialmente, permitindo testar e comprovar o isolamento das áreas ablacionadas cirurgicamente, assim como completar a ablação de regiões inatingíveis pela técnica cirúrgica.^482^

O Cox-Maze (CM) IV é atualmente o tratamento cirúrgico padrão-ouro para FA,^483-487^ com sucesso de 93%, também com a vantagem de ser necessário menor tempo de clipagem aórtica, sendo de 47±26 min. para o CM IV e de 93±34 min. para o CM III. Uma metanálise recente demonstrou que o CM IV isolado via minitoracotomia é tão eficaz quanto via esternotomia, com menor tempo de internação. Os autores ressaltam que a abordagem minimamente invasiva deve ser o procedimento de escolha, quando possível.^488^ O procedimento CM IV associado a outros procedimentos concomitantes também tem demonstrado excelentes resultados, como melhora da QV, aumento da sobrevida a longo prazo e menor risco de AVC a longo prazo.^486,489^

Dados de estudos randomizados e metanálises reportaram a superioridade do tratamento por procedimento híbrido quando comparado à ablação por cateter na manutenção do ritmo sinusal em pacientes com FA persistente e persistente de longa duração, a despeito de maior índice de complicações.^476-478^

A ressecção ou isolamento (sutura ou clipagem) do AAE, para prevenção de fenômenos tromboembólicos, geralmente se associa a essas técnicas ou pode eventualmente ser realizada isoladamente. O procedimento CM IV associado a outros procedimentos concomitantes também têm demonstrado excelentes resultados, como melhora da qualidade de vida, aumento da sobrevida a longo prazo e menor risco de AVC a longo prazo.^486,489^

### 9.1. Indicações para o Tratamento Cirúrgico da Fibrilação Atrial

#### 9.1.1. Tratamento Cirúrgico da Fibrilação Atrial Concomitante a Outra Cirurgia Cardíaca

A maioria dos resultados reportados são em cirurgias combinadas para correção de valvopatia mitral (troca ou plastia) mas também pode ser associado a revascularizações do miocárdio.^492,493^ Uma metanálise publicada em 2022 incluindo três estudos randomizados com cirurgias concomitantes de valva mitral demonstrou que o tratamento cirúrgico com técnica de Cox-Maze (III ou IV) resultou em maior sobrevida livre de FA quando comparado a apenas ao isolamento das veias pulmonares.^494^ O benefício da associação da técnica de Cox-Maze para abordagem da FA concomitante ao tratamento valvar mitral se deu não somente nas taxas de mortalidade, mas também na melhora na QV.

Entretanto, questões relacionadas à segurança (morbidade e mortalidade) ainda precisam de maior esclarecimento e permanecem como as principais limitações. Dados recentes de revisão sistemática e metanálises reportaram que a ablação cirúrgica concomitante ao tratamento valvar mitral resultou em menor taxa de recorrência da FA em 12 meses, sem aumentar mortalidade em 30 dias ou eventos cardiovasculares maiores.^495,496^

#### 9.1.2. Tratamento Cirúrgico Isolado da Fibrilação Atrial

O tratamento cirúrgico isolado da FA é destinado aos portadores da arritmia sem doença cardíaca estrutural e pode ter como objetivo adicional o fechamento do AAE e a promoção de ablação por RF utilizando a toracoscopia como acesso.

Uma metanálise incluindo três estudos randomizados e controlados mostrou resultado favorável da ablação toracoscópica quando comparada à ablação por cateter, demonstrando maior eficácia no controle de taquiarritmia atrial e menor necessidade de repetição do procedimento ablativo tanto para FA paroxística quanto para a persistente.^497^

O estudo FAST (*Ablation or Surgery for Atrial Fibrillation Treatment*) randomizou pacientes com maior chance de insucesso na ablação de FA por cateter e demonstrou recorrência menor após ablação por toracoscopia em comparação com ablação por cateter (56% vs. 87%) no acompanhamento a longo prazo (média de 7 anos); a hospitalização foi mais longa e as taxas de complicações da ablação cirúrgica foram maiores em comparação com a ablação por cateter.^498^

A ablação por toracoscopia mostrou ser mais eficaz no controle do ritmo do que ablação por cateter, porém, é um procedimento mais invasivo, com maiores taxas de complicações e hospitalização mais longa, fazendo com que seja indicada preferencialmente em pacientes com falha anterior na ablação por cateter ou com alto risco de falha na ablação por cateter.

As recomendações para ablação cirúrgica estão apresentadas no Quadro 17

**Quadro 17:** Recomendações para ablação cirúrgica e híbrida

Recomendações	Classe	Nível de evidência
Pacientes com FA sintomática, recorrente e refratária (ao menos uma droga antiarrítmica), que serão submetidos a cirurgia de válvula mitral, a ablação cirúrgica concomitante da FA deve ser considerada. Deve-se ponderar a experiência do grupo e os benefícios da manutenção do ritmo sinusal sobre os riscos cirúrgicos relacionados	**I**	**B**
Pacientes com FA sintomática, recorrente e refratária (ao menos uma droga antiarrítmica), que serão submetidos a cirurgia não envolvendo a válvula mitral, a ablação cirúrgica concomitante da FA pode ser considerada. Deve-se ponderar a experiência do grupo e os benefícios da manutenção do ritmo sinusal sobre os riscos cirúrgicos relacionados	**IIa**	**B**
A ablação híbrida deve ser considerada em pacientes com FA paroxística ou persistente, sintomática e refratária às drogas antiarrítmicas e a ablação endocárdica isolada, com a tomada de decisão conjunta entre eletrofisiologista e cirurgião cardíaco.	**IIb**	**B**

### 9.2. Resultados a Longo Prazo e Complicações

Há décadas o tratamento cirúrgico da FA é uma opção de tratamento eficaz para a manutenção a longo prazo do RS. A cirurgia de Cox-Maze apresenta uma notável taxa de sucesso livre de FA e redução na prevalência de AVC tardio.^473,474,499^

Pacientes submetidos ao Cox-Maze apresentam maior sobrevida quando comparados aos pacientes com FA não tratada.^500^ O Cox-Maze IV é superior aos procedimentos de ablação por cateter ou ablação por cirurgia, principalmente em pacientes com FA persistente ou de longa duração.^486^ Uma revisão sistemática comparou os resultados clínicos a médio prazo entre essas duas técnicas de ablação cirúrgica (Cox-Maze e IVP) concomitante à troca valvar mitral, mostrando menor recorrência de FA em 12 meses nos pacientes submetidos ao CM.^494^

Evidências a longo prazo também demonstram excelentes resultados. Segundo Weimar et al., a ausência de FA sintomática em 10 anos foi de 85%.^501^ Apenas um AVC tardio foi descrito, e 80% dos pacientes não precisaram de terapia anticoagulante durante o período de seguimento.^501^ Os resultados semelhantes foram descritos por Khiabani et al., com taxa de pacientes livres de FA em 1, 5 e 10 anos de 92%, 84% e 77%, respectivamente.^483^

O Cox-Maze IV isolado também demonstrou relevância em pacientes com FA persistente, com baixa morbidade e ausência de FA sintomática em 88% após 7 anos do procedimento.^485^

O procedimento de labirinto realizado através de uma minitoracotomia direita é tão eficaz quanto a esternotomia.^502,503^ Essa abordagem está associada a menos complicações, menor taxa de mortalidade e diminuição do tempo de internação na unidade de terapia intensiva e na permanência hospitalar.^502^

As principais complicações associadas ao CM IV são o aumento da incidência de implante de marca-passo e a maior taxa de lesão renal aguda. A taxa de implante de marca-passo varia de 21,5% a 6,3%.^484,492^ A maior incidência de lesão renal é observada nos pacientes submetidos ao CM IV concomitante (32% contra 16%), devido ao maior tempo de CEC.^504^

Até o momento, ainda não há um estudo robusto que verifique os eventos adversos associados ao CM. Fatores como a variabilidade técnica e tecnológica, associados ou não a outro procedimento cirúrgico, idade avançada e a presença de comorbidades, dificultam a análise de complicações diretamente relacionadas ao procedimento de labirinto.

### 9.3. Procedimentos Híbridos

Os procedimentos híbridos para a ablação da FA se iniciaram em 2009^505^ e consistem em uma abordagem cirúrgica epicárdica inicial (toracoscopia uni/bilateral ou laparoscopia pela região sub-xifoide), com capacidade de englobar a parede posterior do AE, plexos autonômicos, ligamento de Marshall e, finalmente, a exclusão do AAE. A transmuralidade pode ser checada e complementada com mapeamento eletroanatômico e ablação endocárdicos, realizados de forma sequencial concomitante ou depois de alguns meses do procedimento inicial.

O estudo randomizado CONVERGE (*Convergence of Epicardial and Endocardial Ablation for the Treatment of Symptomatic Persistent AF*) foi o primeiro a incluir um grande número de pacientes com FA persistente de longa duração (duração média FA = 4,4 meses), envolvendo 153 pacientes randomizados na razão 2:1 (102 tratamentos híbridos e 51 endocárdicos), demonstrando maior efetividade no grupo submetido ao tratamento híbrido (67% contra 50%, p = 0,03) em 1 ano de evolução.^490^

Uma metanálise incluindo 16 estudos e 1.242 pacientes (apenas um randomizado) demonstrou que a técnica híbrida permite oferecer resultados com ausência de recorrência da FA em 78%, 75% e 73% após 1, 2 e 3 anos de seguimento ambulatorial, respectivamente.^506^

É importante salientar que a indicação de tais procedimentos deve ser realizada por equipes multidisciplinares ("*Heart Team*") envolvendo cirurgião cardíaco e eletrofisiologista que comandam a equipe médica e paramédica para os cuidados gerais dos pacientes submetidos a esse tipo de intervenção.^507^

As bases anatômicas que favorecem e fundamentam o procedimento híbrido justificam a necessidade de maior desenvolvimento tecnológico e mais estudos clínicos para verificar a custo-efetividade, a carga de FA, a QV e a melhora da função ventricular dos pacientes submetidos a esses procedimentos, parâmetros frequentemente comprometidos nos pacientes com FA persistente. São necessários também estudos que consigam identificar as variações técnicas, como a concomitância ou não das abordagens epicárdicas e endocárdicas, o isolamento do AAE e as estratégias e energias utilizadas no endocárdio que podem influenciar significantemente na efetividade e principalmente na segurança dos procedimentos.

## 10. Abordagem Multidisciplinar da Fibrilação Atrial

### 10.1. Implementação de Programa de Mudança de Qualidade de Vida

Hendriks et al. demonstraram que pacientes tratados em clínicas multidisciplinares dedicadas ao tratamento da FA, lideradas por enfermeiras e supervisionadas por cardiologistas, apresentaram redução de 35% no desfecho combinado de internação hospitalar e mortalidade cardiovascular em comparação com pacientes tratados de forma convencional pelo cardiologista.^508^ O tratamento integrado com uma equipe multiprofissional incluindo profissionais da área de saúde, pacientes e familiares pode melhorar desfechos clínicos e melhorar a adesão ao tratamento.^509,510^

Younis et al. investigaram o papel dos programas de clínicas de reabilitação especializados em 292 pacientes com FA no período de 2009 a 2015 para o desfecho primário combinado de internação por causa cardiovascular e mortalidade global. Os autores observaram que pacientes com melhor desempenho cardiopulmonar apresentaram significativamente melhor evolução (RR 0,40; p = 0,001). Os pacientes que conseguiram melhorar o desempenho cardiopulmonar também apresentaram redução significativa de eventos (RR 0,83; p = 0,04) em comparação com aqueles que não melhoraram.^511^

Como o substrato da FA envolve múltiplas comorbidades e diferentes cenários clínicos, uma abordagem multidisciplinar que envolve médico generalista, cardiologista, arritmologista, endocrinologista, clínicas de sono, clínicas de reabilitação cardíaca e profissionais de enfermagem, educação física e fisioterapia é recomendada para a melhora de desfechos clínicos, redução de recorrência da FA e da progressão para formas persistentes da arritmia.

## 11. Fibrilação Atrial em Situações Especiais

### 11.1. Hemorragia Intracraniana

A ocorrência de hemorragia intracraniana (HIC) é uma das situações mais delicadas no manuseio de pacientes com FA. Por ser uma condição de alto potencial letal que normalmente ocorre em pacientes em uso dos anticoagulantes orais ou antiagregantes plaquetários, existe grande resistência em se reiniciar a anticoagulação nesta população, embora também sejam pacientes de alto risco para AVCi.^512^ A decisão de se reintroduzir a anticoagulação nesses pacientes deve ser feita por uma equipe multidisciplinar, envolvendo cardiologista, neurologista, neurocirurgião, hematologista, além do próprio paciente, familiares e responsáveis. Após a ocorrência de HIC, tomar a decisão de anticoagular não é simples, uma vez que esses pacientes foram excluídos de todos os estudos de prevenção de AVC em pacientes com FA. Uma análise cuidadosa do caso, avaliando riscos e benefícios, além da realização de exames de imagem cerebral auxiliarão na tomada de decisão da equipe.^221^

Fatores de risco para recorrência do HIC devem ser avaliados e divididos em dois grupos: os modificáveis e os não modificáveis (Tabela 24). Quando prevalece a presença de fatores de risco passíveis de modificação, sem outros fatores que contraindiquem a anticoagulação, e a relação risco/benefício é considerada favorável, a reintrodução dela pode ser instituída (Figura 19). O momento ideal para o início do uso do anticoagulante não está bem estabelecido, mas deve ser introduzido após a fase aguda, aguardando-se em geral um mínimo de 4 semanas. Embora não haja estudos que tenham comparado ACOD contra varfarina em pacientes com HIC, os ACOD devem ser preferidos devido à redução de aproximadamente 50% na ocorrência de HIC, nos estudos clínicos randomizados.^153^

**Tabela 24:** Fatores de risco para recorrência de hemorragia intracraniana

Fatores modificáveis	Fatores não modificáveis
HAS descontrolada	Idade avançada
Baixos níveis de LDL e triglicérides	Sexo masculino
Consumo excessivo de álcool	Etnia asiática
Uso de ACOD	Insuficiência renal crônica
Drogas simpatomiméticas*	Angiopatia amiloide cerebral
	Doença cerebral de pequenos vasos

* Cocaína, Heroína, Anfetamina, Efedrina. HAS: hipertensão arterial sistêmica; LDL: lipoproteína de baixa densidade; ACOD: anticoagulantes orais de ação direta.

**Figura 20:**
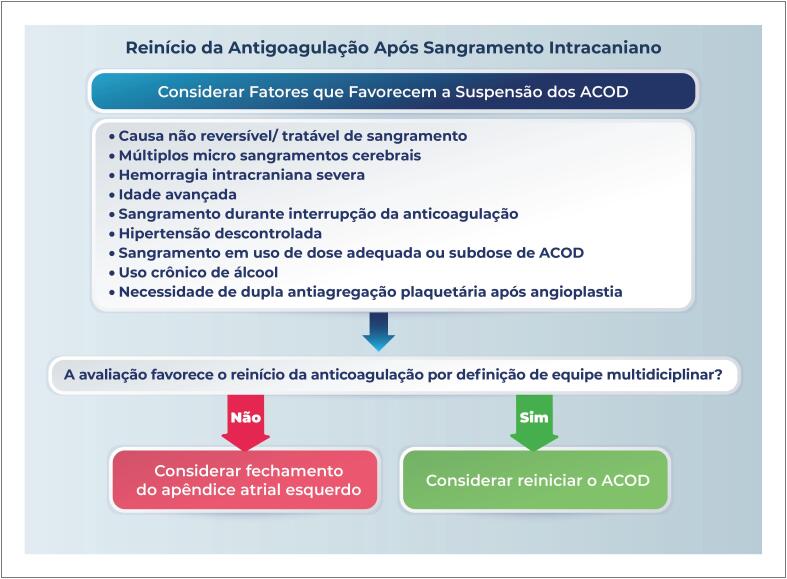
Reintrodução da anticoagulação após sangramento intracraniano. Figura adaptada 2021 EHRA Practical Guide on the use of DOACs(2). DOAC: Anticoagulante de ação direta; a: Sem evidência de estudos clínicos randomizados; b: Estudo de Imagem é mandatório antes de reiniciar o DOAC.

Nos pacientes com causas irreversíveis de HIC ou com fatores de risco que contraindiquem a anticoagulação devido ao alto risco de recorrência, a utilização de oclusão de AAE torna-se uma opção,^512^ apesar de não existirem estudos específicos para essa população. A Figura 20 demostra os fatores que devem ser considerados para o reinício da anticoagulação após sangramento intracraniano.

### 11.2. População Muito Idosa

O diagnóstico da fibrilação atrial (FA) em idosos pode ser desafiador. Nessa população a FA pode ser assintomática ou apresentar sintomas atípicos o que pode levar ao retardo no diagnóstico. Além disso, a coexistência de várias comorbidades e seus respectivos tratamentos farmacológicos podem mascarar os sintomas da FA ou tornar o diagnóstico mais complexo. A dificuldade em diferenciar a FA de outras arritmias cardíacas, como a TA, também representa um desafio na assistência à população geriátrica.

As alterações que ocorrem com a idade na estrutura, contratilidade ou mudanças eletrofisiológicas que afetam os átrios podem predispor a FA. A presença de ICFEp comum nessa faixa etária também pode favorecer a anormalidade do ritmo.

Nos pacientes idosos, sintomáticos ou oligossintomáticos, a presença de extrassístoles atriais frequentes e taquicardias atriais é indicadora de maior instabilidade elétrica atrial e marcador de cardiomiopatia atrial, com consequente maior risco de FA. O Copenhagen Holter Study avaliou por meio do Holter de 48 horas 678 indivíduos sem doença cardiovascular conhecida ou FA prévia e mostrou uma forte associação entre extrassístoles atriais ≥ 30/hora e/ou taquicardias atriais de ≥ 20 batimentos, com AVCi.^513^

A associação entre atividade ectópica supraventricular excessiva e uma carga maior de doença cerebrovascular subclínica também foi avaliada em um estudo transversal que incluiu 462 homens (idade média de 68,1 anos) sem AVC aparente nem FA submetidos a ECG, Holter de 24 horas e ressonância magnética cerebral. A presença de extrassístoles atriais foi independentemente associada a maiores cargas de hiperintensidade de sinal na substância branca, um marcador de doença de pequenos vasos cerebrais, e de estenose aterosclerótica intracraniana, ambos fortes preditores de AVCi.^514^

### 11.3. Grupos Étnicos Minoritários

Apesar do reconhecimento das diferenças étnicas na doença cardiovascular e AVC, a literatura mundial na epidemiologia clínica da FA em grupos não brancos é escassa. Sarraju et al., pesquisando os trabalhos incluídos nas Diretrizes de FA da Sociedade Americana de Cardiologia, encontraram que menos da metade apresentavam dados étnicos raciais.^515^ Já Khan et al. revisaram 134 estudos clínicos randomizados, no qual apenas 12,7% apresentavam tal informação.^516^

Na Ásia, a prevalência de FA é em torno de 1%, menor que nos países ocidentais. No entanto, devido ao tamanho dessa população, estima-se que a FA irá acometer cerca de 60-70 milhões de asiáticos até 2050. Na África, a real prevalência de FA não é conhecida. Estudos pequenos revelam prevalência de 0,7% a 5,5%, sendo que alguns consideram que, em torno de 2050, a prevalência de FA na África será a maior do mundo.^517-519^

Estudos epidemiológicos têm mostrado que a FA é mais prevalente em pacientes brancos do que em outros grupos raciais e etnias. Em estudo recente com 330 milhões de pacientes portadores de FA que tiveram alta hospitalar na Califórnia, foi observado FA mais em brancos quando comparados com negros (11,3% vs 4,6%, p < 0,001). Essa diferença ficou mais significante em pacientes com idade maior que 75 anos (31,4% brancos contra 18,2% negros, p < 0,001).^520^ Dessa forma, parece que indivíduos negros apresentam prevalência mais baixa de FA quando comparados a brancos, entretanto, apresentam uma maior prevalência de fatores de risco tradicionais, o que foi chamado de o "paradoxo da FA".^35, 521-523^ Tal paradoxo também é notado nos asiáticos do sul que possuem taxas ajustadas à idade maiores para diabetes, hipertensão e doença coronária.^524^

Estudos realizados nos últimos 15 anos têm mostrado que descendentes europeus apresentam nove *loci* genéticos associados a risco de FA.^525^ Pacientes negros e hispânicos com FA de início precoce têm apresentado maiores chances de apresentar um familiar de primeiro grau com FA do que os brancos, sugerindo uma predisposição genética que deve ser explorada.^526^

Os sintomas também apresentam diferenciação de acordo com a raça. Com base na Associação Europeia de Arritmia, pacientes negros reportam sintomas mais severos e debilitantes (20%) quando comparados a brancos (16,4%) e hispânicos (8,5%). Além disso, pacientes negros apresentam pior QV em 2 anos de seguimento.^527^

A evolução clínica também apresenta algumas diferenças. Um estudo com mais de 500.000 pacientes acima de 65 anos de idade com FA mostrou que negros e hispânicos apresentavam um maior risco de morte (46% e 11%, respectivamente) e de AVC (66% e 21%) quando comparado aos brancos.^528^ As incidências de AVCi e hemorrágico são mais altas em pacientes negros não hispânicos e asiáticos numa subanálise do estudo ROCKET AF, que utilizou rivaroxabana.^529^ No estudo ARISTOTLE, que utilizou apixabana como anticoagulante, alguns pacientes asiáticos apresentaram maior risco para ambas as formas de AVC, isquêmico e hemorrágico.^517^

Portanto, as disparidades raciais afetam a evolução dos cuidados de saúde, mesmo após o ajuste dos fatores socioeconômicos. O desafio é a criação de novos planos de cobertura de saúde e fluxos de trabalho clínico customizados para aumentar o acesso e a qualidade do cuidado para as minorias étnicas e raciais.^530^

### 11.4. Insuficiência Renal

A insuficiência renal crônica está presente em mais de 40% dos pacientes com FA e é um dos fatores de aumento de mortalidade.^531,532^ Por outro lado, a FA pode acelerar a progressão da doença renal.^532^ Em relação ao acompanhamento dos pacientes com a associação de insuficiência renal e FA, o melhor preditor independente de complicações embólicas ou hemorrágicas é o ClCr.^532^

A insuficiência renal, por si, é uma condição protrombótica e pró-hemorrágica, e, por essa razão, os principais estudos randomizados de prevenção de embolia cerebral com o uso dos ACOD corrigiam a dose das drogas quando o ClCr estava abaixo de 50%.^149-152^ Os estudos dos ACODs, quando compararam pacientes com insuficiência renal leve a moderada com pacientes sem insuficiência renal, apresentaram eficácia e segurança semelhantes.^533-536^ Desse modo, a utilização dos ACODs nos pacientes com disfunção renal deve levar em consideração os fatores de risco demonstrados na Tabela 24 desta Diretriz. Em relação aos pacientes com ClCr menor que 15 mL/min ou em terapia dialítica, os estudos observacionais questionam o benefício do uso de anticoagulantes orais.^17^ Uma redução de sangramento foi observada com uso dos ACODs quando comparado aos cumarínicos,^537^ mas uma metanálise demonstrou que não havia benefício na redução de AVCi ou embolia sistêmica com uso dos anticoagulantes orais.^538^

Um estudo multicêntrico e randomizado (RENAL-AF) que incluiu 154 pacientes com insuficiência renal crônica e tratamento dialítico, foi interrompido prematuramente devido a dificuldades na inclusão de pacientes, e não apresentou poder estatístico para concluir que a apixabana ou varfarina foram diferentes em relação a sangamentos.^539^ No entanto, o número de sangramentos relevantes foi dez vezes maior do que o de eventos isquêmicos, seja AVCi ou embolia sistêmica. O estudo AXADIA-AFNET 8 randomizou 97 pacientes com FA, insuficiência renal crônica e tratamento a longo prazo com hemodiálise. O objetivo foi demonstrar a não inferioridade da apixabana em relação aos inibidores de vitamina K quanto à sua eficácia e segurança. A conclusão foi que não houve diferença na segurança ou eficácia; no entanto, os pacientes permaneceram em alto risco dos eventos cardiovasculares, com alta mortalidade e sangramento.^540^ Portanto, até o presente momento, não está claro o benefício do uso do anticoagulante oral no grupo de pacientes com FA associada a insuficiência renal crônica em terapia renal substitutiva.

### 11.5. Fibrilação Atrial na Gestação

As doenças cardiovasculares são a principal causa de mortalidade materna, sendo as arritmias a principal complicação cardíaca observada na gestação.^541^ Em gestantes, a incidência de FA é relativamente baixa e está mais relacionada à presença de cardiopatias preexistentes, como as cardiopatias congênitas, as miocardiopatias e principalmente a valvopatia mitral, com alta prevalência no nosso meio, especialmente de etiologia reumática. Em mulheres grávidas sem cardiopatia estrutural, a obesidade e a idade avançada são os principais fatores de risco para a arritmia. A incidência de FA é cerca de cinco vezes maior em mulheres que engravidam após os 35 anos de idade quando comparadas com as de menos de 25 anos.^542^

A repercussão clínica da FA em mulheres grávidas é influenciada pelas mudanças fisiológicas cardiovasculares no organismo materno, com aumento significativo do volume plasmático e do débito cardíaco a partir do segundo trimestre da gestação e da FC, principalmente no terceiro trimestre, efeito bastante relacionado ao aumento do tônus adrenérgico. O aumento da volemia, associado ao estado hiperadrenérgico, proporciona um maior estresse de parede atrial, causando o aumento de atividade automática e de atividade deflagrada nos miócitos atriais. Devido à alta demanda hemodinâmica, a presença de FA na gestante tende a ser menos tolerada e pode ser fator de descompensação de cardiopatia preexistente. Por mecanismos não claramente compreendidos, a FA está relacionada ao maior risco de complicações obstétricas como pré-eclâmpsia, eclampsia e prematuridade.^543^

Em gestantes muito sintomáticas, o controle do ritmo pode ser uma estratégia que apresenta melhor resposta clínica. A cardioversão elétrica pode ser feita com segurança de modo semelhante à realizada fora da gestação, com risco mínimo de repercussão elétrica no coração fetal, que deve ser monitorizado adequadamente. O tratamento farmacológico antiarrítmico em pacientes assintomáticas e estáveis clinicamente deve ser evitado no primeiro trimestre da gestação, principalmente no período de organogênese fetal entre a quinta e décima semanas. A partir do segundo trimestre, a propafenona e o sotalol apresentam perfil aceitável de segurança (classe C da FDA – efeitos adversos no feto em estudos animais, sem estudos em humanos, porém os potenciais benefícios do tratamento justificam o seu uso) com objetivo de manter o ritmo sinusal. A amiodarona não deve ser usada pelo maior risco de reações adversas significativas relacionadas ao feto.^544^ A ablação por cateter pode ser realizada em situações de maior gravidade, havendo o cuidado para mínima exposição radiológica no feto, ou utilização de técnicas sem auxílio da fluoroscopia.^545^

Caso a manutenção do ritmo sinusal não seja viável, o controle da FC deve ser instituído preferencialmente com betabloqueadores cardiosseletivos (bisoprolol ou metoprolol) ou bloqueadores de canais de cálcio não diidropiridínicos (verapamil ou diltiazem). A digoxina é considerada droga de segunda linha neste contexto e pode ser utilizada no caso de controle insatisfatório dos sintomas, isoladamente ou em associação com os agentes preferenciais descritos, observando-se as interações medicamentosas. Embora os efeitos adversos com os betabloqueadores, como o menor crescimento intrauterino e a hipoglicemia fetal, sejam característicos desta classe de medicamentos, o atenolol está relacionado com maior risco de reações adversas, não sendo recomendado para uso durante a gestação.^546,547^ As recomendações para controle do ritmo e da FC nas pacientes estão apresentadas no Quadro 18.

**Quadro 18:** Recomendações para controle de ritmo na gestante

Recomendação	Classe	NE
A anticoagulação com heparina de baixo peso molecular ou AVK (exceto no primeiro trimestre e após a 36° semana de gestação) é recomedada para gestantes com risco tromboembólico alto.	**I**	**C**
Os betabloqueadores (exceto atenolol) é recomendado para o controle da FC durante a gestação para reduzir os sintomas e melhorar os desfechos materno e fetal.	**I**	**C**
Cardioversão elétrica para restauração do ritmo sinusal na presença de instabilidade hemodinâmica ou sintomas limitantes, observando a obrigatoriedade da monitorização fetal.	**I**	**C**
A digoxina é recomendada para o controle da FC durante a gestação para reduzir os sintomas e melhorar os desfechos materno e fetal, nos casos de intolerância ou falta de resposta aos betabloqueadores	**IIa**	**C**
Propafenona ou sotalol utilizado para a manutenção do ritmo sinusal a partir do segundo trimestre da gestação, em casos de insucesso no controle da frequência.	**IIb**	**C**
Ablação por cateter utilizada em casos selecionados com refratariedade ao tratamento e sintomas limitantes, observando-se mínima ou nenhuma exposição fluoroscópica fetal.	**IIb**	**C**
ACOD utilizado para anticoagulação em gestantes	**III**	**B**
Amiodarona oral para o controle do ritmo ou controle de FC em qualquer fase da gestação.	**III**	**B**

Apesar de a gravidez proporcionar um estado de hipercoagulabilidade, o papel da anticoagulação na prevenção de TE não está bem estabelecido. O escore de CHA_2_DS_2_-VA não foi validado em gestantes, embora esse parâmetro seja aceito na identificação de pacientes de maior risco. Na presença de valvopatia mitral significativa, principalmente de etiologia reumática, a anticoagulação deve ser avaliada já no primeiro trimestre, sendo a alternativa mais segura neste período a utilização de heparina de baixo peso molecular. A varfarina pode ser administrada com relativa segurança a partir do segundo trimestre, devendo ser trocada por heparina de baixo peso molecular no período periparto. Não há informações consistentes acerca dos efeitos dos ACODs durante a gestação, podendo inclusive essa classe de drogas estar associada a risco de abortos espontâneos e anormalidades na gravidez.^548^

### 11.6. Fibrilação Atrial, Sistema Nervoso Parassimpático e Cardioneuroablação

A inervação vagal contribui para a indução e manutenção FA ao influenciar a atividade elétrica dos átrios. A redução do período refratário das paredes atriais, aumenta a dispersão da refratariedade e do transiente de cálcio sustentando a FA. Além disso, pode influenciar nos disparos das veias pulmonares podendo deflagrar a FA.^549^ A atividade vagal pode influenciar o remodelamento atrial, resultando em alterações estruturais e elétricas que contribuem para FA.^550^ Estes mecanismos demonstram a influência vagal na FA, observada em jovem sem cardiopatia significativa. No entanto, é importante considerar que a FA é uma condição complexa com múltiplos fatores contribuintes e a inervação vagal é apenas um dos vários mecanismos envolvidos.

Sharifov et al.^551^ demonstraram que a infusão direta de acetilcolina na artéria do nódulo sinusal induz FA em 100% dos cães. A FA mediada por acetilcolina é facilitada por isoproterenol, que diminuiu a concentração limiar de acetilcolina necessária para a indução de FA além de prolongar os episódios de FA. A infusão isolada de isoproterenol na artéria do nó sinusal também provoca FA. Entretanto, se o efeito vagal é previamente bloqueado com atropina o isoproterenol não induz a FA, mostrando que as catecolaminas, isoladamente, sem o efeito vagal são pouco indutoras de FA.

Na década de 90, utilizando análise espectral, foi verificado que áreas do miocárdio atrial com grande inervação apresentam alterações características no eletrograma que podem ser detectadas por análise espectrais utilizando a transformada rápida de Fourier.^552,553^ Nesses locais, a interpolação de fibras neurais reduz a conexão elétrica laterolateral das células miocárdicas, permitindo a micro-reentrada e a manutenção da FA. Parte da inervação vagal entra nos átrios pelas veias pulmonares em cujo antro encontra-se grande quantidade da inervação parassimpática. A ablação destes locais nas paredes atriais esquerda e direita provoca intensa denervação vagal e estabiliza eletricamente as paredes atriais, tornando os átrios mais resistentes à estimulação atrial progressiva sem reindução de FA.

A cardioneuroablação se baseia no mapeamento e na ablação desses plexos ganglionados. Trata-se de uma técnica que permite a denervação vagal através de ablação endocárdica por RF nas paredes atriais. Desta forma, a cardioneuroablação pode ser sobreposta à ablação de FA com isolamento das veias pulmonares, podendo ter aplicação nessa população.

Uma metanálise de ablação de FA associada a cardioneuroablação anatômica empírica demonstrou melhor resultado no grupo paroxístico com coração estruturalmente normal tratado com denervação parassimpática concomitante ao isolamento das veias pulmonares, entretanto, não foi evidenciado benefício no grupo com FA persistente.^554^

Recentemente, algumas técnicas de ablação poupam a inervação vagal, como a ablação com campo pulsado (PFA), dessa forma, parte do efeito benéfico dessa tecnica poderá ser perdido.^555^ Osório et al, demonstraram que a crioablação reduz, porém preserva considerável efeito vagal.^556^

### 11.7. Fibrilação Atrial no Atleta

A atividade física diminui o risco de doenças cardiovasculares, promove envelhecimento saudável e participa ativamente do bem-estar físico e emocional. Inclusive reduz a ocorrência de FA e deve ser recomendada nesses pacientes.^72^ Entretanto, foi demonstrado que homens praticantes de esportes de alto desempenho apresentavam aumento no risco dessa anormalidade do ritmo,^557^ sugerindo que a relação entre FA e esporte é uma curva em "U".^558^ Esportes como corrida e ciclismo estão associados ao maior risco.^75^

Os mecanismos envolvidos no aumento no risco de FA em atletas profissionais são complexos e incluem: dilatação atrial, estimulação adrenérgica, alteração no tônus vagal, inflamação, fibrose e alterações nas propriedades eletrofisiológicas.^559,560^ Vários outros mecanismos ainda estão em investigação.

Os pacientes mais propensos são do sexo masculino, de meia-idade, estatura mais alta e tempo de prática mais prolongado. Não há estudos randomizados que respaldem a conduta em pacientes atletas; entretanto, esportes com contato corporal direto precisam ser evitados nos pacientes elegíveis a anticoagulação. Devido à bradicardia sinusal frequente nessa população, o tratamento medicamentoso é dificultado, e a ablação por cateter normalmente é preferível.

### 11.8. Síndromes Arrítmicas

#### 11.8.1. Síndrome Arrítmica em Jovens sem Aparente Cardiopatia Estrutural

A FA em jovens sem aparente cardiopatia estrutural pode ser considerada uma doença mais rara, com prevalência menor que 1%, uma condição anteriormente chamada de "FA isolada".^561^ Atualmente, esse termo tem sido menos utilizado, devido à compreensão em constante evolução da complexidade da doença e de seus mecanismos moleculares subjacentes.

Atualmente, foram observados dois padrões familiares relacionados à FA como uma doença monogênica, com herança mendeliana: (1) FA sem cardiopatia estrutural, relacionada a canalopatias atriais; e (2) FA familial no contexto de outra cardiomiopatia hereditária, com sinais ainda incipientes da cardiopatia ventricular.^562^ Até o momento, mais de 30 genes foram associados à FA; a maioria deles codifica canais iônicos, proteínas envolvidas no metabolismo do cálcio, bem como genes associados à fibrose, a doença de condução e a inflamação, atualmente mais conhecida como cardiomiopatia atrial. Apesar do aumento da conscientização sobre os aspectos genéticos da FA, o teste genético tem recomendação restrita, e até o momento há poucos genes definitivamente associados.^563^

Pacientes com canalopatias, como a síndrome de Brugada, têm uma maior prevalência de FA, e, em algumas coortes, a presença de FA foi considerada um fator adicional de risco para morte súbita. Na síndrome do QT curto (SQTC), a FA é um critério diagnóstico e está presente em metade dos pacientes.^564^ Embora seja mais rara, também podemos observar FA em pacientes com síndrome do QT longo (SQTL) e taquicardia ventricular polimórfica catecolaminérgica (TVPC).^565^

Recentemente, a FA tem sido observada como uma manifestação inicial de algumas cardiomiopatias hereditárias, mesmo antes da ocorrência de sobrecarga hemodinâmica. Isso sugere que a FA pode ser causada por uma cardiomiopatia específica que afeta primariamente o átrio e que pode ter um caráter progressivo.^566^

Um ponto importante na prática clínica para o reconhecimento da FA de origem genética está na terapêutica antiarrítmica. Na síndrome de Brugada, os medicamentos da classe I são contraindicados (www.brugadadrugs.org), o que proíbe o uso de propafenona como tratamento agudo (esquema "*pill in the pocket*"). Na síndrome do QT longo, muitos medicamentos são contraindicados devido ao aumento do risco de prolongamento do intervalo QT e *torsades de pointes* (http://www.crediblemeds.org/). Além disso, a ablação em jovens com canalopatias ou cardiomiopatias atriais pode não ser tão eficaz quanto o esperado nessa faixa etária.

Assim, a FA em jovens, anteriormente considerada uma arritmia de fácil controle e com poucas implicações prognósticas relevantes, passou por uma mudança de paradigma com o aumento da disponibilidade dos testes genéticos e o reconhecimento de padrões familiares. Os diagnósticos genéticos têm impacto em diversos aspectos do manejo clínico, incluindo o rastreamento e aconselhamento familiar, a prescrição de exercícios, a terapia farmacológica e a indicação de cardiodesfibriladores. Dessa forma, é razoável utilizar a avaliação genética da FA na prática clínica em casos selecionados, especialmente em pacientes mais jovens (<60 anos), onde exista a suspeita de FA familiar estabelecida com base na análise do histórico clínico do paciente, história familiar e características do ECG. As análises devem incluir os genes SCN5A, LMNA, KCNQ1, MYL4 e TTN.

### 11.9. Arritmias atriais em pacientes adultos com Cardiopatia Congênita

As taquicardias atriais reentrantes, incluindo o FLA típico e atípico e a FA, são as taquiarritmias sustentadas mais comuns na população adulta portadora de cardiopatia congênita. Em uma coorte retrospectiva com 482 com cardiopatia congênita, as taquicardias atriais foram encontradas em 61,6%, enquanto a FA esteve presente em 28,8%. A prevalência da FA aumenta com a idade, sendo mais comuns acima dos 50 anos (51,2% vs. 44,2%).^567^

O substrato arritmogênico da arritmia atrial envolve a presença de cicatriz atrial e material protético, sendo que a complexidade da cardiopatia congênita, a incisão atrial e o número de cirurgias cardíacas prévias são fatores de risco para a sua ocorrência. A prevalência das TA também aumenta após a cirurgia de Fontan^568^ e em portadores de tetralogia de Fallot corrigida, com insuficiência tricúspide grave.^569^ Por outro lado, o substrato arritmogênico da FA inclui também mecanismos de remodelamento elétrico e estrutural, além de fatores de risco como idade e hipertensão arterial que estão associados à ocorrência de FA em adultos com cardiopatia congênita.^567,570^ A presença de um defeito do septo interatrial não corrigido ou corrigido tardiamente tem sido associada a uma maior incidência de FA,^571^ e o fechamento cirúrgico ou percutâneo está relacionado à redução na prevalência de FA.^572^

Não obstante ao aumento de sobrevida da população adulta portadora de cardiopatia congênita, as evidências disponíveis para guiar o tratamento das arritmias atriais nessa população são predominantemente derivadas de estudos observacionais ou extrapoladas de grandes ensaios clínicos.

A indicação de anticoagulação deve ser considerada levando em conta a complexidade da cardiopatia congênita. É recomendada a anticoagulação para todos os portadores de TA ou FA e cardiopatia congênita de complexidade moderada a severa, incluindo aqueles com correção cirúrgica intracardíaca, cardiopatia cianogênica, ventrículo direito sistêmico ou pós-cirurgia de Fontan. Nos portadores de TA/FA e cardiopatia congênita de baixa complexidade, a anticoagulação é indicada se houver um ou mais fatores de risco tromboembólico (escore CHADS-VA ≥ 1).^17,570,573^ Entretanto, a validade dos escores CHADS-VA e HAS-BLED nessa população é incerta; portanto, a aplicação e a interpretação desses escores apresenta algumas limitações. O uso de ACODs na população adulta com cardiopatia congênita parece ser seguro e eficaz na ausência de prótese valvar mecânica ou estenose mitral moderada a grave.^574-576^

A manutenção do ritmo sinusal é desejável nos pacientes com cardiopatia congênita. Assim, o controle de ritmo é geralmente considerado como a primeira abordagem terapêutica para TA ou FA em portadores de cardiopatia congênita de complexidade moderada a severa.^574,577^ O controle da FC permanece como estratégia potencialmente útil naqueles com cardiopatia congênita simples ou naquelas mais complexas quando há falha das estratégias de controle de ritmo.^577^ Nos casos em que há a presença FA e defeito do septo interatrial, é importante considerar a cirurgia para FA, como a técnica de CM ou a ablação por cateter durante o procedimento de fechamento do defeito do septo interatrial.

É importante destacar que os portadores de cardiopatia congênita com TA ou FA frequentemente apresentam disfunção do nó sinusal, aumentando o risco de pausa sinusal ou bradicardia sinusal após a reversão aguda da arritmia. Portanto, é fundamental ter disponível um marca-passo temporário durante a tentativa de cardioversão.^570^

As drogas antiarrítmicas da classe IC, como a propafenona, devem ser utilizadas com cautela devido ao risco de alentecimento da taquicardia atrial (TA) resultando em condução AV 1:1.^578^ Além disso, é importante ressaltar que a presença de fibrose miocárdica, disfunção ventricular ou hipertrofia é comum em adultos com cardiopatia congênita. Portanto, é recomendado evitar o uso de antiarrítmicos da classe IC em pacientes com disfunção ou hipertrofia do ventrículo sistêmico.

A amiodarona pode ser uma opção para evitar a recorrência de TA ou FA em pacientes cardiopatia congênita que apresentam disfunção ou hipertrofia do ventrículo sistêmico, especialmente quando a ablação por cateter não foi eficaz.^579,580^ Devido ao perfil de efeitos colaterais da amiodarona, é necessário ter cautela com o seu uso em pacientes com cardiopatia congênita cianogênica, baixo peso, doenças hepáticas, pulmonares ou da tireoide, bem como na presença de intervalo QT prolongado. Essas condições podem aumentar o risco de complicações relacionadas à medicação.^581,582^

A longo prazo, a ablação por cateter é geralmente considerada a primeira opção em pacientes TA e cardiopatia congênita, principalmente quando é tecnicamente viável e o substrato arritmogênico for bem definido. Entretanto, embora o sucesso agudo seja alto, as taxas de recorrência, principalmente nos casos de FLA, são altos nessa população, podendo chegar a 50%.^583-585^ Quando se programa uma ablação em um paciente com cardiopatia congênita complexa, especialmente nos casos de pacientes com Fontan ou anomalias complexas, é fundamental o conhecimento da anatomia através dos exames de imagem como tomografia computadorizada e também com os sistemas de mapeamento eletroanatômicos.^586-588^ Além disso, em alguns casos, é necessária a punção do tubo para acesso ao átrio venoso, devendo o eletrofisiologista estar habituado com esse acesso.^589,590^ Nos casos de pacientes submetidos a diferentes cirurgias, o conhecimento da técnica utilizada também é essencial quando se programa ablação nessa população (Quadro 19).

**Quadro 19:** Recomendações para o manejo de taquicardia atrial (TA) reentrante e fibrilação atrial (FA) em adultos com cardiopatia congênita

Recomendações	Classe de recomendação	Nível de evidência
A ablação por cateter deve ser realizada em pacientes com TA reentrante sintomática e recorrente nos pacientes com cardiopatia congênita de baixa complexidade, sendo preferível ao uso de drogas antiarrítmicas para controle de ritmo no longo prazo	**I**	**C**
A ablação por cateter pode ser considerada em pacientes com TA reentrante sintomática e recorrente nos pacientes com cardiopatia congênita de moderada a alta complexidade, desde que realizada em centros especializados	**IIa**	**C**
A ablação por cateter de FA ou cirurgia para FA (procedimento de Cox-Maze) devem ser consideradas em pacientes com doença do septo interatrial submetidos à correção percutânea ou cirúrgica, respectivamente	**IIa**	**C**
A anticoagulação oral deve ser considerada para todos os adultos com FA ou TA e cardiopatia congênita, na presença de cirurgia corretiva intracardíaca prévia, cianose, procedimento de Fontan prévio ou ventrículo direito sistêmico, independentemente do escore CHADS-VA. Em adultos com FA ou TA e outras cardiopatias congênitas, anticoagulação oral deve ser considerada se CHADS-VA ≥2.	**I**	**C**

### 11.10. Peculiaridades da Fibrilação Atrial em Mulheres

Estima-se que 29,4 milhões de mulheres tenham FA ao redor do mundo. Embora a incidência seja maior entre os homens, mulheres mais velhas têm mais FA, visto que a expectativa de vida nas mulheres é maior.^17,22^ Quando comparadas aos homens com FA, as mulheres são mais velhas, têm maior prevalência de hipertensão, doença valvar, ICFEp e baixa prevalência de doença cardíaca isquêmica.^591^ As mulheres apresentam preditores de risco peculiares para a FA. Um estudo com 34.221 mulheres (1,8% FA) e seguimento de 12,4 anos observou que a hipertensão, particularmente a sistólica, foi um preditor de risco de FA entre as mulheres de meia-idade que eram inicialmente saudáveis.^592,593^ O estudo LEGACY já havia demonstrado que a perda e a manutenção do peso estavam associadas a significante redução na carga de FA e manutenção do RS em ambos os sexos.^66^ O IMC esteve linearmente associado ao risco de incidência de FA em um grande grupo de mulheres estudadas, demonstrando que obesidade é outro marcador de risco importante entre as mulheres e que mudanças dinâmicas no peso também são deletérias.^594^ As mulheres que praticam atividade física vigorosa têm 28% de redução de incidência de FA; no entanto, foram poucas as que praticaram atividade física nesse nível em uma grande coorte. Dados prospectivos sugerem que quantidades modestas de atividade física regular estão associadas a um risco reduzido de FA em mulheres de meia-idade que eram inicialmente saudáveis, principalmente se ajustado pelo índice de massa corpórea.^595^ Entre mulheres saudáveis de meia-idade, foi demonstrada uma incidência aumentada de FA naquelas que consumiam duas ou mais doses por dia, sendo o consumo excessivo de álcool um preditor de FA entre as mulheres. Em um estudo prospectivo com 30.034 mulheres, a menopausa não foi significativamente relacionada à incidência de FA, enquanto o uso de monoterapia com estrogênio foi associado a aumento do seu risco, sugerindo uma ligação fisiopatológica entre a exposição ao estrogênio e a arritmia em mulheres. Em comparação as nulíparas, um número aumentado de gestações foi associado a um aumento linear no risco de FA em uma grande coorte de mulheres inicialmente saudáveis. A exposição repetitiva a mudanças hormonais, metabólicas e fisiológicas durante a gravidez pode predispor à FA mais tarde na vida.^596^ Um estudo com seguimento médio de 20,6 anos demonstrou que as mulheres que tiveram FA e desenvolveram IC tiveram um aumento da mortalidade por todas as causas, da mortalidade cardiovascular e do infarto do miocárdio. Em análise multivariada desta população, as mudanças nos fatores de risco hipertensão sistólica (PAS < 120 mmHg), obesidade (IMC < 30 kg/m^2^), tabagismo e diabetes melito foram associadas a redução progressiva no risco de IC nessas mulheres quanto maior o número de fatores controlados.^597^

Estudos histológicos mostram que as mulheres com FA de longa data têm uma fibrose aumentada quando comparada a mulheres sem FA, o que nem sempre é visto nos homens. Essa diferença entre os sexos se deve a diferentes expressões nos genes e proteínas que causam o remodelamento fibrótico.^598^ Um estudo de ressonância magnética com quantificação de fibrose pelo realce tardio confirmou que mulheres idosas têm mais fibrose que os homens na mesma faixa etária. Essas diferenças entre os sexos são ainda mais pronunciadas na presença de AVC, sugerindo o papel do sexo feminino em uma remodelação fibrótica atrial mais acentuada e subsequente AVC.^599^

Muitos estudos examinaram a associação entre a diferença de sexos e o risco de AVC na FA demonstrando que as mulheres têm 20 a 30% de excesso de risco contra os homens, mesmo depois de ajustado para fatores de risco para AVC, anticoagulação e idade. Entretanto, recentemente foi proposta, pela Sociedade Europeia, a retirada do sexo feminino no escore de CHADS-VA, pois os critérios de recomendação de anticoagulação não eram diferentes, sendo essa modificação também implementada nesta Diretriz.

Os estudos com ACODs contaram com 35 a 40% de mulheres. Uma metanálise que avaliou as diferenças de sexo no risco residual de AVC e sangramento maior em pacientes com FA não valvular tratados com ACODs demonstrou que mulheres têm mais risco de AVC e embolia sistêmica quando tratadas com varfarina e menor risco de sangramento maior com os ACODs quando comparadas com os homens. Não houve diferença significativa no risco de AVC e embolia sistêmica quando tratadas com ACODs e no risco de sangramento maior com uso da varfarina quando comparadas com os homens. Tais resultados sugerem um aumento no benefício clínico do ACOD comparado com a varfarina no tratamento de mulheres com FA.^600^ Além disso, as mulheres demonstraram redução de mortalidade por todas as causas e redução significativa no risco de hemorragia intracraniana com uso dos ACODs quando comparado com varfarina.^601^

As mulheres com FA têm mais sintomas e pior QV quando comparadas com os homens.^120^ Dados da subanálise de QV do estudo ORBIT com 4.293 pacientes (42% mulheres) demonstraram que as mulheres com FA eram mais velhas e tinham uma mediana maior no escores de CHA_2_DS_2_-VA e apenas 32,1% eram assintomáticas comparadas com 42,5% dos homens. Elas apresentaram piores escores de QV, incluindo maiores limitações nas suas atividades diárias e maior preocupação com o tratamento, muito embora a maioria delas estivesse em RS no momento da avaliação.^602^

A despeito de serem mais sintomáticas, as mulheres recebem menos controle de ritmo que os homens. As mulheres são menos submetidas a ablação e a cardioversão elétrica, enquanto são mais submetidas a ablação da junção AV e implante de marca-passo quando comparadas aos homens.^603^ Adicionalmente, são menos selecionadas para uso de DAA classe III, e, devido a características eletrofisiológicas próprias do sexo feminino que possui maior intervalo QTc de base, as mulheres toleram menos essas drogas que prolongam o intervalo QTc.^604^

A ablação por cateter da FA melhora sintomas relacionados à FA, tem efeitos positivos na redução da mortalidade por IC, reduz hospitalização e AVC, além de melhorar a função cognitiva em pacientes com FA. Em geral, mulheres têm menos indicação para ablação da FA e, quando esta acontece, é mais tardia,^605^ as mesmas apresentam achados clínicos pré-ablação mais complexos, há uma maior recorrência,^606^ maior prevalência de focos extraveias pulmonares^607^ e maiores taxas de complicações relacionadas ao procedimento.^608,609^ Em relação à mortalidade, o estudo de Framingham já havia demonstrado que a mortalidade com FA é maior em ambos, homens e mulheres, e não há diferença entre eles na presença da FA.^610^

Sendo assim, esta Diretriz recomenda que, independentemente do sexo, incluindo a diversidade de gênero, o tratamento deve ser direcionado para a redução no risco de fenômenos tromboembólicos, controle do ritmo e melhoria da QV. Estudos que melhor avaliem as diferenças sexuais na apresentação e tratamento da FA são necessários na literatura.

### 11.11. Fibrilação Atrial no Portador de Dispositivo Cardíaco Eletrônico Implantável

#### 11.11.1. Detecção

Os DCEI disponibilizam o armazenamento de dados através dos eletrogramas registrados pelos eletrodos e possuem algoritmos capazes de interpretação automática que permitem o diagnóstico de taquiarritmias. A detecção de FA no registro do DCEI pode estar ou não relacionada a sintomas, ao registro em ECG de 12 derivações ou a eventos embólicos prévios. No entanto, a detecção dessas taquiarritmias requer a avaliação criteriosa por parte do médico especializado para que se discriminem eventos falsos que podem ser registrados por *oversensing* no canal atrial. Uma vez confirmados os episódios registrados, pode-se quantificar a carga de FA e a duração dos episódios verdadeiros, que guardam relação com o risco global do paciente.^130^ Os estudos que avaliaram a relação entre a densidade de arritmias atriais detectadas no DCEI e eventos tromboembólicos apontam para maior risco em pacientes que apresentem eventos com duração superior a 5 minutos. A carga total registrada de maneira contínua guarda relação proporcional ao risco de eventos embólicos: quanto maior a duração registrada dos episódios de FA, maior o risco relativo.^611^

#### 11.11.2. Ajustes de Programação

A estimulação cardíaca artificial pode ser necessária em vários cenários no contexto dos pacientes com FA. Portadores de bradiarritmias com indicação de marca-passo podem evoluir com FA ao longo do seguimento, assim como as bradiarritmias podem estar presentes no paciente com FA, seja por disfunção do nó sinusal, do nó AV (FA de baixa resposta ventricular) ou por efeito cronotrópico negativo de eventuais tratamentos farmacológicos antiarrítmicos. Entretanto, a estimulação atrial não parece ter efeito, nem no aumento^612^ ou na redução da incidência de FA.^613^

Nos pacientes portadores de marca-passo dupla câmara, os modos deflagrados (DDD ou VDD) são inadequados durante episódios de FA por possibilitarem frequências elevadas para os ventrículos. Diante de episódios sustentados de FA, em pacientes com necessidade de suporte de frequência por bloqueio AV, deve se manter o suporte em modo inibitório, o que limita à frequência mínima de estimulação, sem acompanhar a frequência atrial elevada (VVI, DDI). O suporte otimizado em pacientes com episódios paroxísticos inclui a programação de mudança automática de modo (*Mode Switching*) que modifica para o modo DDI(R) assim que o algoritmo detecta frequência atrial persistentemente elevada. O algoritmo permite o retorno ao modo DDD após reversão da arritmia detectada pela monitorização da frequência atrial.^294^

A detecção inadequada de FA pode ser ocasionada por eventuais problemas de sensibilidade, de condução retrógrada ventrículo-atrial e de ajuste de período refratário atrial após a detecção do QRS. Além disso, a estimulação inadequada em período refratário atrial pode propiciar a deflagração de arritmias atriais por estimulação inadequada em período refratário atrial.^614^

Deve ser dada atenção aos portadores de distúrbio de condução interatrial, especialmente naqueles com IC e terapia de ressincronização. Geralmente, o intervalo AV sugerido para esses pacientes são curtos para promoção da estimulação biventricular sem possibilidade de fusão e, com isso, os portadores de atraso de condução pelo feixe interatrial de Bachmann atrasam a ativação do AE e com isso dissincronia AV à esquerda, aumentando, assim, a sobrecarga pressórica atrial e as chances de desenvolver FA. Nos portadores de terapia de ressincronização cardíaca com distúrbios de condução interatrial, a programação do intervalo AV otimizada pelo ecocardiograma poderá ser necessária.

#### 11.11.3. Cardioversão e Ablação

O portador de DCEI pode ser candidato a procedimento de cardioversão elétrica. A segurança do procedimento e a necessidade de ajuste e avaliação após a cardioversão são questões importantes nesse cenário. A disponibilidade de desfibriladores bifásicos impacta a eficácia da desfibrilação com menores energias e possivelmente com mais segurança para o portador de DCEI. Existem diversas séries de casos demonstrando a segurança do procedimento de desfibrilação no portador de DCEI. As preocupações incluem desgaste da bateria, indução de choque inapropriado no caso dos desfibriladores, inibição anormal, dano ou deslocamento dos eletrodos.^615^ Em uma análise retrospectiva baseada em extenso banco de dados nacional dinamarquês, Elgaard et al. avaliaram 2.582 portadores de DCEI submetidos a cardioversão elétrica e compararam com uma amostra pareada de mais de 12 mil pacientes. Nessa análise, foi encontrado um número maior de intervenções ao final de 1 ano para troca de gerador (HR 1,73) e revisão de eletrodo (HR 2,85). Apesar de ser uma análise retrospectiva e o tempo de latência para os eventos após a cardioversão ser longo, os autores alertam para a necessidade de atenção ao portador de marca-passo ou desfibrilador submetido a cardioversão elétrica.^616^

Nos portadores de DCEI que serão submetidos a ablação por cateter, é sugerido que seja realizada uma avaliação eletrônica antes e após o procedimento. Primeiro, na telemetria inicial, pode-se desligar os registros para não gravar desnecessariamente ruídos provenientes do procedimento e registrar os limiares de detecção e de captura para que o procedimento não possa ser imputado como causa de aumentos de limiares que possam estar presentes previamente. A avaliação do ritmo intrínseco de escape e risco de assistolias em casos de pacientes dependentes de estimulação cardíaca, a utilização de modo de estimulação assíncronos para o ventrículo permitem segurança para a aplicação de RF evitando inibições desnecessárias durante o procedimento. No entanto, a estimulação assíncrona no átrio deverá ser evitada pois pode ser prejudicial na construção dos mapas de ativação, além de poder inibir possíveis extrassístoles atriais. Nos casos dos portadores de CDI, as terapias para arritmia ventricular deverão ser desabilitadas, pois a fonte de energia poderá levar a detecção imprópria e o choque inapropriado. Ao término do procedimento, a nova telemetria trará à tona possíveis modificações estruturais e novos limiares de sensibilidade e captura das câmaras abordadas com a possibilidade de apagar possíveis eletrogramas gravados a partir do início do procedimento. A possibilidade de monitorização do ritmo cardíaco será fundamental para acompanhamento do resultado pós-procedimento, acompanhamento que poderá ser realizado através de monitoramento remoto que a maioria dos dispositivos realizam.

### 11.12. Fibrilação Atrial no Pós-operatório de Cirurgia Cardíaca e Não Cardíaca

A FA no pós-operatório é uma ocorrência específica, caracterizada por ocorrer no período pós-cirúrgico em pacientes sem o diagnóstico prévio da arritmia.^617-619^ Embora os mecanismos da FA no pós-operatório não sejam completamente conhecidos, acredita-se que a presença de gatilhos, por exemplo, das veias pulmonares, e substratos anatômicos e/ou eletrofisiológicos sejam responsáveis pelo início e manutenção da FA. Além disso, durante o período pós-operatório, existe um ambiente favorável para o desenvolvimento da arritmia, resultante do tônus simpático aumentado, presença de fatores inflamatórios, estresse oxidativo e distúrbios hidroeletrolíticos, em especial, hipomagnesemia.^620^

A despeito dos avanços tecnológicos atuais, a incidência de FA no pós-operatório permanece estável, tem impacto no prognóstico e no tempo de internação e gera custos adicionais. Os pacientes apresentam maior risco de AVC, infarto do miocárdio, internação por IC congestiva e mortalidade, tanto no período pós-operatório quanto no seguimento de 12 meses após a cirurgia.^621-623^ Aproximadamente 90% dos casos ocorrem em até 6 dias após a cirurgia, podendo ocorrer entre 5-10% em cirurgias não cardíacas e chegar a 55% em cirurgias cardíacas, dependendo do tipo de cirurgia e da definição de FA, pois alguns autores consideram como FA qualquer ocorrência com duração maior que 30 segundos, enquanto outros consideram apenas episódios sintomáticos e maiores que 10 minutos (Quadro 20).

**Quadro 20:** Recomendações para pacientes com dispositivos cardíacos eletrônicos implantáveis (DCEI)

Recomendações	Classe	Nível de evidência
Programação em AAI(R) é recomendada em pacientes com FA paroxística e doença do nó sinusal com condução AV preservada que cursam com resposta ventricular adequada durante os episódios de FA paroxística	**I**	**C**
Programação de mudança automática de MODO (Mode switching) é recomendada em pacientes com FA paroxística quando programado em modo DDD/VDD	**I**	**C**
O uso de algoritmos que promova a busca intrínseca da condução AV é recomendado em pacientes com FA paroxística com estimulação em modo DDD e condução AV preservada	**IIa**	**B**
Programação em modo VVI(R) deve ser evitada em pacientes com bradicardia sinusal ou FA paroxística com resposta ventricular adequada	**III**	**C**

#### 11.12.1. Fatores Preditores

Os preditores de FA no pós-operatório podem ser avaliados de acordo com dados clínicos (cardíacos e não cardíacos) e cirúrgicos (tipos de cirurgia).

#### 11.12.2. Comorbidades e Dados Epidemiológicos

A história clínica prévia é fundamental na avaliação de risco de FA no pós-operatório. A IC é um dos preditores mais importantes de FA no pós-operatório. Yamashita et al. relataram que a IC pode aumentar em até 56% a ocorrência de FA no pós-operatório de cirurgia cardíaca.^624^ Outros fatores importantes que resultam em aumento do risco de FA em ordem decrescente incluem DPOC (36%), hipertensão arterial (29%), história de infarto do miocárdio (18%) e diabetes (6%). Idade avançada, sexo masculino, obesidade e aumento de AE também são fatores correlacionados à FA no pós-operatório.

##### 11.12.2.1. Preditores Cirúrgicos

Entre os fatores cirúrgicos importantes na FA no pós-operatório, destacam-se: tipo de cirurgia (cardíaca, não cardíaca torácica e não cardíaca não torácica), tempo de cirurgia, necessidade de reposição volêmica e agressividade cirúrgica.

A incidência na cirurgia cardíaca também depende do tipo de cirurgia, podendo chegar a 20% após revascularização sem extracorpórea ou acima de 50% após troca valvar ou combinação de ambas.^625,626^ Os procedimentos cardiológicos por cateter, como a TAVI e o tratamento percutâneo da insuficiência mitral, como o MitraClip, que têm se tornado progressivamente rotineiros na prática clínica, também estão associados à ocorrência de FA no pós-operatório. Sua ocorrência é maior nos procedimentos valvares mitral e tricúspide quando comparado à valva aórtica.

Vários escores para identificar pacientes com maior risco de FA no pós-operatório de cirurgia cardíaca foram testados isoladamente ou em combinação.^627-629^

As cirurgias não cardíacas possuem menor risco de FA, ocorrendo entre 0,5% em cirurgias mais simples, como uma cesárea, até 30% em cirurgias mais complexas e invasivas, como cirurgia de colo retal.^630^ As cirurgias torácicas se encontram em um risco intermediário entre as não cardíacas e cardíacas, incidência de 10-20%, podendo ser maior em cirurgias mais extensas, como pneumectomia, transplante de pulmão e esofagectomia.

#### 11.12.3. Prevenção Pré-operatória

A prevenção da FA em pacientes que serão submetidos a cirurgia é parte fundamental do tratamento. Várias drogas, cardiológicas e não cardiológicas, foram testadas.

Os betabloqueadores são recomendados para redução da incidência de FA, porém, os dados disponíveis atuais mostram que em até 25% dos pacientes eles são suspensos, principalmente por efeitos colaterais, notadamente bradicardia e hipotensão, e devem ser utilizados em pacientes submetidos à cirurgia cardíaca com cautela.^631^ É importante notar que a redução na incidência de FA no pós-operatório com betabloqueadores profiláticos não se traduziu em redução de AVC, morte e tempo de internação em uma metanálise com 33 estudos randomizados.^632^ É importante mencionar que nesse estudo o melhor betabloqueador foi o carvedilol.

Por outro lado, em cirurgias não cardíacas, os betabloqueadores causam mais riscos do que benefícios e não devem ser utilizados profilaticamente. Em um estudo multicêntrico com mais 8 mil pacientes, pacientes randomizados para metoprolol, eles tiveram maior mortalidade (33%) e duas vezes mais AVC que o grupo placebo.^633^ Em uma metanálise de estudos randomizados com 14 mil pacientes, o uso de betabloqueadores reduziu a incidência de infarto e FA no pós-operatório, mas sem redução da mortalidade e AVC.^634^ Dessa forma, o seu uso não é recomendado como tratamento profilático para cirurgias não cardíacas (Quadro 21).

**Quadro 21:** Prevenção e tratamento de fibrilação atrial (FA) no perioperatório de cirurgia cardíaca e não cardíaca

Recomendação	Classe	NE
O uso de amiodarona no perioperatório é recomendado quando é necessária a prevenção de FA pós-operatoria em pacientes submetidos a cirurgia cardíaca.	**I**	**A**
Os betabloqueadores não devem ser utilizados de forma rotineira para a prevenção de FA em pacientes submetidos a cirurgia não cardíaca.	**III**	**C**

Existem evidências de benefício do uso de amiodarona profilática na redução de de FA no pós-operatório e tempo de internação em cirurgias cardíacas, entretanto, tais benefícios não se traduzem em redução de mortalidade e AVC.^635-637^ Seu uso é recomendado para cirurgias cardíacas e parece ter maior benefício na dose acumulada maior que 3.000 mg.^635^

A colchicina foi comparada em estudos randomizados e apresentou resultados controversos. O estudo END-AF (*Effect of Low Dose ColchiciNe on the InciDence of POAF*), que foi terminado prematuramente e com baixo poder de detecção, avaliando baixas doses de colchicina em cirurgia cardíaca, mostrou resultado negativo na redução da incidência de FA no pós-operatório de cirurgia cardíaca.^638^ Por outro lado, no estudo COPPS (*Colchicine for the Prevention of the Post-Pericardiotomy Syndrome*), 1 mês de colchicina reduziu a incidência de FA (12% contra 22%, p = 0,021) e tempo de internação em cirurgia cardíaca.^639^ Atualmente, a colchicina está sendo investigada no estudo multicêntrico COP-AF trial, e os pacientes submetidos à cirurgia torácica não cardíaca estão sendo randomizados. Seus resultados poderão fornecer informações mais precisas quanto ao seu benefício.^640^

#### 11.12.4. Tratamento da Fibrilação Atrial no Pós-operatório

Assim como em pacientes com FA não relacionada ao pós-operatório, pode se optar pelo controle da FC e anticoagulação ou pela reversão da arritimia, por cardioversão elétrica ou química. Em casos de instabilidade hemodinâmica, está recomendada a cardioversão elétrica imediata.^640^

Embora existam evidências de pior prognóstico a curto e longo prazo em pacientes que apresentam FA no pós-operatório, além de uma maior taxa de ocorrência de FA durante o seguimento clínico, os estudos que compararam controle do ritmo e o controle de frequência mostraram resultados semelhantes para ambos os tratamentos. Entretanto, mais de 95% dos pacientes recebem alta hospitalar em ritmo sinusal, o que mostra uma natureza transitória da FA, quando sua primeira ocorrência é detectada no pós-operatório. As opções para o controle de FC ou para a reversão da FA no pós-operatório seguem as mesmas recomendações indicadas para FA não relacionada ao período pós-operatório e são discutidos em outra seção desta Diretriz (Quadro 21).

## 12. Fibrilação Atrial e Alterações Cognitivas

Diversos estudos observacionais apontam a FA como um fator de risco isolado para o desenvolvimento de declínio cognitivo e demência.^641-645^ Esse acometimento neurocognitivo ocorre mesmo na ausência de um AVC clínico ou infartos cerebrais silenciosos detectados por neuroimagem.^646^

Outros mecanismos conhecidos são os microinfartos (causados por microembolia) e os microsangramentos, os quais não são vistos por técnica de imagem convencional. Além disso, a hipoperfusão cerebral por perda da contração atrial e por variabilidade da contração ventricular está relacionada ao desenvolvimento de demência, assim como o estado pró-inflamatório dos pacientes com FA e o uso de betabloqueadores.^646^

Além da demência vascular, a FA está relacionada com um maior risco do desenvolvimento de outros tipos de demência como a demência de Alzheimer^647^ e a demência senil.^644,647^ Uma metanálise demonstrou que a FA também está associada ao surgimento de demência de início precoce, antes dos 70 anos de idade.^647^ O início mais precoce da FA foi associado a um maior risco de demência, demência de Alzheimer e vascular.^648^

Alguns estudos sugerem que a anticoagulação é capaz de reduzir a incidência de demência na população com FA.^649^ Os ACODs parecem ser superiores à varfarina na prevenção da ocorrência de déficit cognitivo,^113,650^ fato possivelmente explicado pelas questões posológicas e segurança. O efeito protetor da varfarina está intimamente relacionado ao tempo na faixa terapêutica (TTR), enquanto TTRs abaixo de 75% ou RNI supraterapêutica estão associados a um maior risco de demência.^651^

**Figura 21:**
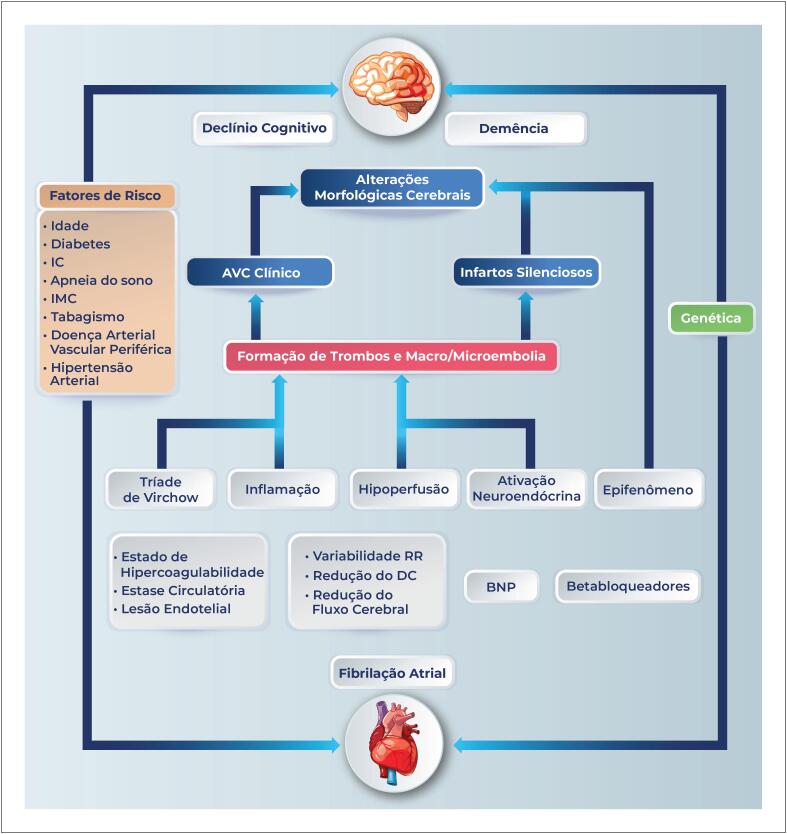
Associação entre fibrilação atrial e alterações cognitivas. Adaptado de Diener et al.^646^ IC: insuficiência cardíaca; IMC: índice de massa corporal; DAOP: doença arterial obstrutiva periférica; AVC: acidente vascular cerebral.

Com relação às estratégias de controle de ritmo, os dados são conflitantes. A ablação por cateter parece promissora em relação à redução importante do declínio cognitivo e desenvolvimento de demência.^652-654^ A cardioversão elétrica se mostrou capaz de promover uma melhora do fluxo cerebral,^655^ no entanto, mais estudos randomizados são necessários para entender o real papel do controle de ritmo na prevenção de demência e declínio cognitivo. A Figura 21 demonstra a associação entre a FA e alterações cognitivas.

## 13. Importância de Ações Educacionais no Manejo do Paciente com Fibrilação Atrial

A visão holística e multidisciplinar no tratamento do paciente com FA é fundamental para que melhores desfechos clínicos possam ser atingidos. Nesse sentido, a educação dos pacientes, seus familiares, médicos e profissionais de saúde torna-se determinante para o sucesso do tratamento.

O estudo randomizado e internacional IMPACT-AF^160^ com 2.281 pacientes demonstrou que intervenções educacionais multifacetadas e multiníveis levam a um aumento de mais de três vezes o uso adequado de ACOD em pacientes com FA e risco de aumento para AVC e embolia sistêmica. Além disso, esse estudo de maneira única demonstrou que tais intervenções se associaram em uma redução de 52% nas taxas de fenômenos tromboembólicos, independentemente do anticoagulante utilizado. Além de diminuir os eventos isquêmicos, a intervenção educacional não elevou as taxas de sangramento a despeito do aumento significativo na proporção de pacientes anticoagulados. Desse modo, fica claro que intervenções passivas e ativas para educação continuada de todos os envolvidos são necessárias para que as barreiras à anticoagulação possam ser superadas e a inércia terapêutica combatida, culminando com o tratamento de excelência nesses pacientes.

## 14. Análise de Custo-benefício e Uso Adequado de Recursos em Fibrilação Atrial

O financiamento da saúde é um tópico desafiador em todas as áreas da medicina. No cenário da FA, arritmia cardíaca mais comum e que tem efeitos devastadores em milhões de pessoas em todo o mundo, há necessidade de análise cuidadosa de custo-benefício e de uso eficiente dos recursos disponíveis. As análises devem abranger as diversas etapas do manejo da FA: 1) a triagem e o diagnóstico preciso, campo que conta com ampla e contínua evolução tecnológica (principalmente com os DCEI, métodos diagnósticos vestíveis etc.); 2) a prevenção de eventos tromboembólicos, que inclui a discussão da incorporação dos ACODs e das próteses de oclusão de apêndice; 3) as estratégias de controle de ritmo ou controle de FC com todas as suas possibilidades terapêuticas (DAA, ablação em suas diversas formas e implante de DCEI); 4) as estratégias de controle de fatores de risco, tarefa que envolve ações de educação em saúde. Infelizmente, diversas etapas não possuem estudos de custo efetividade disponíveis no cenário mundial e nacional.

No que diz respeito à triagem da FA, o estudo STROKESTOP (*Systematic ECG Screening for Atrial Fibrillation Among 75 Year Old Subjects in the Region of Stockholm and Halland, Sweden*) randomizou 27.975 pacientes com seguimento médio de 6,9 anos. O objetivo do estudo foi estimar o custo e a efetividade de investigação de FA populacional usando desfechos clínicos. O estudo demonstrou uma efetividade alta e economia com probabilidade de 92,7%, denotando que uma estratégia populacional ampla de investigação de FA em idosos é custo efetiva e esforços devem ser implemenados para maior abrangência diagnóstica.^656^ Ressalta-se que esse estudo utilizou dispositivo com dois registros diários de ECG por 14 dias. Hoje já existem diversas tecnologias vestíveis ou implantáveis que necessitam de avaliação específica de custos tanto em relação ao benefício da triagem quanto em sua efetividade.^13,20^

A capacidade de predizer eventos tromboembólicos foi testada recentemente através da medicina de precisão, que combina variáveis clínicas (idade, AVC prévio e sangramento) com biomarcadores (troponina ultrassensível, NT-proBNP, DGF-15 e hemoglobina) *versus* o tratamento habitual numa perspectiva de 20 anos de utilização do sistema de saúde canadense. Houve decréscimo de custo geral de 7% e aumento nos anos de anos de vida ajustados pela qualidade (QALY) de 12%. Os autores concluíram que a medicina de precisão é uma estratégia promissora e projetada para a redução de custos.^657^

A anticoagulação baseada no escore de risco CHA_2_DS_2_-VA é a conduta preventiva eficaz para evitar eventos tromboembólicos da FA. Diversos estudos em diferentes países compararam os antagonistas de vitamina K com os ACODs, sendo os ACODs na sua grande maioria custo-efetivos. No entanto, não há análise para o nosso país, e o cenário atual de quebra de patente com consequente redução de custo da medicação tem tornado mais acessível a aquisição da medicação pela população. Estudos de custo-efetividade são importantes para garantir no futuro o acesso universal pelo Sistema Único de Saúde brasileiro.

No arsenal do tratamento da FA relacionado ao controle de ritmo ou da frequência, há diversas possibilidades, o que dificulta a realização de estudos de custo-efetividade específicos. A ablação é mais eficaz no controle do ritmo do que as medicações antiarrítmicas, sendo uma estratégia que pode ser utilizada como primeira linha de tratamento. Um estudo coreano, baseado em avaliação econômica de mundo real, concluiu que a estratégia de controle de ritmo com ablação para pacientes com FA abaixo de 75 anos de idade foi considerada uma terapêutica custo-efetiva, com um incremento de custo-efetividade de $7,913/QALY.^658^ O estudo CABANA demonstrou que a ablação de FA é mais efetiva em melhorar sintomas, e sua análise de custo-efetividade demonstrou um incremento de aproximadamente 58.000$ por QALY ganho no grupo da ablação comparado ao grupo de DAA, o que seria economicamente atrativo nos Estados Unidos.^659^ Uma subanálise de custo-efetividade do estudo alemão EAST-AFNET 4 demonstrou que o controle precoce de ritmo é benéfico para a saúde a um custo adicional razoável.^660^ No entanto, somente 25% dos pacientes foram submetidos a ablação, e os autores ressaltam que são necessários estudos com uma perspectiva de custo mais ampla, avaliando os diferentes subgrupos clínicos, os tipos de tratamento e as diferentes regiões, para elucidar a questão. Portanto, no cenário brasileiro, em que a ablação da FA é utilizada preferencialmente na saúde suplementar, análises de custo-efetividade são importantes e devem ser encorajadas.

Um estudo brasileiro de custo-efetividade da ablação de FA no sistema de saúde privado com 83 pacientes demonstrou que, em um seguimento de 10,7±5,4 meses, a taxa livre de recorrência foi de 83,6%, refletindo em uma redução do custo mensal mediano de 63,5% (p < 0,001), caindo de R$ 286,00 para R$ 104,00 (Q1: R$ 57,00; Q3: 232,00). Essa redução foi por diminuição de atendimentos de emergência e ambulatoriais.^661^

## Abreviaturas

– AAE: Apêndice atrial esquerdo
– ACOD: Anticoagulantes orais de ação direta
– AIT: Ataque isquêmico transitório
– AVC: Acidente vascular cerebral
– AVCh: Acidente vascular cerebral hemorrágico
– AVCi: Acidente vascular cerebral isquêmico
– AVK: Anticoagulantes antagonistas da vitamina K
– ClCr: Clearance de creatinina
– DAC: Doença arterial coronariana
– DCEI: Dispositivos cardíacos eletrônicos implantáveis
– ECG: Eletrocardiograma
– FA: Fibrilação atrial
– FC: Frequência cardíaca
– FEVE: Fração de ejeção do ventrículo esquerdo
– FLA: Flutter atrial
– IC: Insuficiência cardíaca
– ICC: Insuficiência cardíaca congestiva
– ICFEp: Insuficiência cardíaca com fração de ejeção preservada
– ICFEr: Insuficiência cardíaca com fração de ejeção reduzida
– QV: Qualidade de vida
– SCA: Síndrome coronariana aguda
– TAVI: Implante transcateter de válvula aórtica
– TES: Tromboembolismo sistêmico periférico
– TFG: Taxa de filtração glomerular
