# Supplementary material for: Diretriz Brasileira de Fibrilação Atrial – 2025
Source: Arq Bras Cardiol. 2025 Sep 16;122(9):e20250618. [Article in Portuguese] doi: 10.36660/abc.20250618 (PMC13400077; doi:10.36660/abc.20250618)
Supplement: Translation

# Translation: Brazilian Atrial Fibrillation Guidelines – 2025

**DOI:** 10.36660/abc.20250618i

**Authors:** Fatima Dumas Cintra, Cristiano Faria Pisani, André Gustavo da Silva Rezende, Benhur Davi Henz, Luciana Vidal Armaganijan, Maurício Pimentel, Renato D. Lopes, Thais Aguiar do Nascimento, Adalberto Menezes Lorga, Afonso Luiz Tavares de Albuquerque, Alexsandro Fagundes, Almir Alamino Lacalle, Ana Luisa Calixto Rodrigues, Andre d’Avila, Angelo Amato Vincenzo De Paola, Anibal Pires Borges, Carlos Antônio Abunader Kalil, Carlos Eduardo de Souza Miranda, Carlos Eduardo Duarte, Carlos Manuel de Almeida Brandão, Dalmo Antonio R. Moreira, Dário Celestino Sobral, Denise Tessariol Hachul, Eduardo Benchimol Saad, Elerson Arfelli, Enrique Indalécio Pachón Mateo, Érika Olivier Vilela Bragança, Fernando Ribeiro de Moraes, Francisco Carlos da Costa Darrieux, Guilherme Fenelon, Gustavo Glotz de Lima, Jacob Atié, João Carlos Ferreira Leal, Jose Carlos Moura Jorge, José Carlos Pachón Mateos, José Marcos Moreira, José Tarcísio Medeiros de Vasconcelos, Leandro Ioschpe Zimerman, Luciana Sacilotto, Luiz Pereira de Magalhães, Luiz Roberto Leite da Silva, Marcelo Garcia Leal, Marcio Augusto Silva, Marcio Jansen de Oliveira Figueiredo, Martha Valéria Tavares Pinheiro, Mauricio Ibrahim Scanavacca, Olga Ferreira de Souza, Ricardo Alves da Costa, Ricardo Ryoshim Kuniyoshi, Rui Manuel de Sousa Sequeira Antunes de Almeida, Silvia Helena Cardoso Boghossian, Tan Chen Wu, Thiago da Rocha Rodrigues, Veridiana Silva de Andrade

**Affiliations:** 1 São Paulo SP Brazil Universidade Federal de São Paulo, Escola Paulista de Medicina, São Paulo, SP – Brazil; 2 São Paulo SP Brazil Hospital das Clínicas da Faculdade de Medicina da Universidade de São Paulo (HCFMUSP), São Paulo, SP – Brazil; 3 Recife PE Brazil PROCAPE (Pronto Socorro Cardiológico Universitário de Pernambuco Prof. Luiz Tavares), da Universidade de Pernambuco (UPE) Recife, PE – Brazil; 4 Brasília DF Brazil Instituto Brasília de Arritmia Cardíaca, Brasília, DF – Brazil; 5 Brasília DF Brazil Hospital do Coração do Brazil, Brasília, DF – Brazil; 6 São Paulo SP Brazil Instituto Dante Pazzanese de Cardiologia, São Paulo, SP – Brazil; 7 Porto Alegre RS Brazil Hospital de Clínicas de Porto Alegre, Porto Alegre, RS – Brazil; 8 Durham EUA Duke University Medical Center, Durham – EUA; 9 Salvador BA Brazil Hospital Santa Izabel, Salvador, BA – Brazil; 10 Salvador BA Brazil Hospital São Rafael, Salvador, BA – Brazil; 11 Salvador BA Brazil Hospital Ana Nery, Salvador, BA – Brazil; 12 São José de Rio Preto SP Brazil Instituto de Moléstias Cardiovasculares (IMC), São José de Rio Preto, SP – Brazil; 13 São José de Rio Preto SP Brazil Hospital de Base da Faculdade de Medicina de S. J. Rio Preto – FAMERP, São José de Rio Preto, SP – Brazil; 14 Salvador BA Brazil Universidade do Estado da Bahia, Salvador, BA – Brazil; 15 Ribeirão Preto SP Brazil Hospital das Clínicas da Faculdade de Medicina da Universidade de São Paulo de Ribeirão Preto (HCRPUSP), Ribeirão Preto, SP – Brazil; 16 Votuporanga SP Brazil Santa Casa de Votuporanga, Votuporanga, SP – Brazil; 17 Belo Horizonte MG Brazil Hospital Felício Rocho, Belo Horizonte, MG – Brazil; 18 Florianópolis SC Brazil Hospital SOS Cardio, Florianópolis, SC – Brazil; 19 Porto Alegre RS Brazil Santa Casa de Misericórdia de Porto Alegre, Porto Alegre, RS – Brazil; 20 Porto Alegre RS Brazil Hospital de Clínicas de Porto Alegre, Porto Alegre, RS – Brazil; 21 Belo Horizonte MG Brazil Hospital Madre Teresa, Belo Horizonte, MG – Brazil; 22 São Paulo SP Brazil Hospital Beneficência Portuguesa de São Paulo, São Paulo, SP – Brazil; 23 São Paulo SP Brazil Instituto do Coração (Incor) do Hospital das Clínicas da Faculdade de Medicina da Universidade de São Paulo (HCFMUSP), São Paulo, SP – Brazil; 24 Recife PE Brazil Universidade de Pernambuco, Recife, PE – Brazil; 25 Rio de Janeiro RJ Brazil Hospital Samaritano, Rio de Janeiro, RJ – Brazil; 26 São Paulo SP Brazil Hospital do Coração (HCor), São Paulo, SP – Brazil; 27 São José dos Campos SP Brazil RitmoCheck, São José dos Campos, SP – Brazil; 28 Recife PE Brazil Universidade Federal de Pernambuco, Recife, PE – Brazil; 29 São Paulo SP Brazil Hospital Israelita Albert Einstein, São Paulo, SP – Brazil; 30 Porto Alegre RS Brazil Instituto de Cardiologia do Rio Grande do Sul, Porto Alegre, RS – Brazil; 31 Rio de Janeiro RJ Brazil Universidade Federal do Rio de Janeiro (UFRJ), Rio de Janeiro RJ – Brazil; 32 São José do Rio Preto SP Brazil Faculdade Estadual de Medicina de São José do Rio Preto (FAMERP), São José do Rio Preto, SP – Brazil; 33 São José do Rio Preto SP Brazil Hospital Beneficência Portuguesa de São José do Rio Preto, São José do Rio Preto, SP – Brazil; 34 Curitiba PR Brazil Pontifícia Universidade Católica do Paraná, Curitiba, PR – Brazil; 35 São Paulo SP Brazil SEMAP Arritmias Dr. Pachón, São Paulo, SP – Brazil; 36 São Paulo SP Brazil Hospital do Servidor Público Estadual de São Paulo, São Paulo, SP – Brazil; 37 Porto Alegre RS Brazil Hospital Moinhos de Vento, Porto Alegre, RS – Brazil; 38 Salvador BA Brazil Hospital Universitário Professor Edgard Santos da Faculdade de Medicina da Universidade Federal da Bahia, Salvador, BA – Brazil; 39 Brasília DF Brazil Grupo Santa Hospitais, Brasília, DF – Brazil; 40 Serra ES Brazil Vitória Apart Hospital, Serra, ES – Brazil; 41 Vitória ES Brazil Hospital Universitário Cassiano Antônio de Moraes (UFES), Vitória, ES – Brazil; 42 Campinas SP Brazil Unimed Campinas, Campinas, SP – Brazil; 43 Rio de Janeiro RJ Brazil RedeD’Or Hospitais, Rio de Janeiro RJ – Brazil; 44 Vitória ES Brazil Centrocor Vitória, Vitória, ES – Brazil; 45 Cascavel PR Brazil Centro Universitário Assis Gurgacz, Cascavel, PR – Brazil; 46 Rio de Janeiro RJ Brazil Universidade do Estado do Rio de Janeiro (UERJ), Rio de Janeiro RJ – Brazil

**Table:** 

Brazilian Atrial Fibrillation Guidelines – 2025	
The report below lists declarations of interest as reported to the SBC by the experts during the period of the development of these statement, 2023/2025.	
Expert	Type of relationship with industry
Adalberto Menezes Lorga Filho	Financial declaration A - Economically relevant payments of any kind made to (i) you, (ii) your spouse/partner or any other person living with you, (iii) any legal person in which any of these is either a direct or indirect controlling owner, business partner, shareholder or participant; any payments received for lectures, lessons, training instruction, compensation, fees paid for participation in advisory boards, investigative boards or other committees, etc. from the brazilian or international pharmaceutical, orthosis, prosthesis, equipment and implants industry: - Libbs, Daiichi Sankyo: atrial fibrillation.
Afonso Luiz Tavares de Albuquerque	Nothing to be declared
Alexsandro Fagundes	Nothing to be declared
Almir Alamino Lacalle	Nothing to be declared
Ana Luisa Calixto Rodrigues	Nothing to be declared
Andre D'Avila	Nothing to be declared
André Gustavo da Silva Rezende	Other relationships Performance, in the previous year, as a medical auditor for health insurance companies or the like: - Technical reviewer in the area of Cardiac Arrhythmias at the operators Hapvida, Notre Dame, Intermedicas.
Angelo Amato Vincenzo de Paola	Nothing to be declared
Anibal Pires Borges	Financial declaration A - Economically relevant payments of any kind made to (i) you, (ii) your spouse/partner or any other person living with you, (iii) any legal person in which any of these is either a direct or indirect controlling owner, business partner, shareholder or participant; any payments received for lectures, lessons, training instruction, compensation, fees paid for participation in advisory boards, investigative boards or other committees, etc. from the brazilian or international pharmaceutical, orthosis, prosthesis, equipment and implants industry: - Jonhson & Johnson: lectures. Other relationships Funding of continuing medical education activities, including travel, accommodation and registration in conferences and courses, from the brazilian or international pharmaceutical, orthosis, prosthesis, equipment and implants industry: - Johnson & Johnson: company-owned events.
Benhur Davi Henz	Nothing to be declared
Carlos Antônio Abunader Kalil	Financial declaration A - Economically relevant payments of any kind made to (i) you, (ii) your spouse/partner or any other person living with you, (iii) any legal person in which any of these is either a direct or indirect controlling owner, business partner, shareholder or participant; any payments received for lectures, lessons, training instruction, compensation, fees paid for participation in advisory boards, investigative boards or other committees, etc. from the brazilian or international pharmaceutical, orthosis, prosthesis, equipment and implants industry: - Biotronik, ABBOTT, Boston Sc, Johnson & Johnson: lectures. Other relationships Funding of continuing medical education activities, including travel, accommodation and registration in conferences and courses, from the brazilian or international pharmaceutical, orthosis, prosthesis, equipment and implants industry: - Biotronik, ABBOTT, Boston Sc, Johnson & Jonhson: events
Carlos Eduardo de Souza Miranda	Financial declaration A - Economically relevant payments of any kind made to (i) you, (ii) your spouse/partner or any other person living with you, (iii) any legal person in which any of these is either a direct or indirect controlling owner, business partner, shareholder or participant; any payments received for lectures, lessons, training instruction, compensation, fees paid for participation in advisory boards, investigative boards or other committees, etc. from the brazilian or international pharmaceutical, orthosis, prosthesis, equipment and implants industry: - Daiichi Sankyo: Lixiana.
Carlos Eduardo Duarte	Financial declaration A - Economically relevant payments of any kind made to (i) you, (ii) your spouse/partner or any other person living with you, (iii) any legal person in which any of these is either a direct or indirect controlling owner, business partner, shareholder or participant; any payments received for lectures, lessons, training instruction, compensation, fees paid for participation in advisory boards, investigative boards or other committees, etc. from the brazilian or international pharmaceutical, orthosis, prosthesis, equipment and implants industry: - Medtronic: Micra Artificial Heart Pacing.
Carlos Manuel de Almeida Brandão	Nothing to be declared
Cristiano Faria Pisani	Financial declaration A - Economically relevant payments of any kind made to (i) you, (ii) your spouse/partner or any other person living with you, (iii) any legal person in which any of these is either a direct or indirect controlling owner, business partner, shareholder or participant; any payments received for lectures, lessons, training instruction, compensation, fees paid for participation in advisory boards, investigative boards or other committees, etc. from the brazilian or international pharmaceutical, orthosis, prosthesis, equipment and implants industry: - Johnson & Johnson.
Dalmo Antonio R. Moreira	Nothing to be declared
Dário Celestino Sobral Filho	Nothing to be declared
Denise Tessariol Hachul	Nothing to be declared
Eduardo Benchimol Saad	Financial declaration A - Economically relevant payments of any kind made to (i) you, (ii) your spouse/partner or any other person living with you, (iii) any legal person in which any of these is either a direct or indirect controlling owner, business partner, shareholder or participant; any payments received for lectures, lessons, training instruction, compensation, fees paid for participation in advisory boards, investigative boards or other committees, etc. from the brazilian or international pharmaceutical, orthosis, prosthesis, equipment and implants industry: - Abbott; J&J.
Elerson Arfelli	Financial declaration A - Economically relevant payments of any kind made to (i) you, (ii) your spouse/partner or any other person living with you, (iii) any legal person in which any of these is either a direct or indirect controlling owner, business partner, shareholder or participant; any payments received for lectures, lessons, training instruction, compensation, fees paid for participation in advisory boards, investigative boards or other committees, etc. from the brazilian or international pharmaceutical, orthosis, prosthesis, equipment and implants industry: - Daiichi Sankyo.
Enrique Indalécio Pachón Mateo	Nothing to be declared
Érika Olivier Vilela Bragança	Financial declaration A - Economically relevant payments of any kind made to (i) you, (ii) your spouse/partner or any other person living with you, (iii) any legal person in which any of these is either a direct or indirect controlling owner, business partner, shareholder or participant; any payments received for lectures, lessons, training instruction, compensation, fees paid for participation in advisory boards, investigative boards or other committees, etc. from the brazilian or international pharmaceutical, orthosis, prosthesis, equipment and implants industry: - Biolab: Dozoito. Other relationships Funding of continuing medical education activities, including travel, accommodation and registration in conferences and courses, from the brazilian or international pharmaceutical, orthosis, prosthesis, equipment and implants industry: - Merck.
Fatima Dumas Cintra	Other relationships Funding of continuing medical education activities, including travel, accommodation and registration in conferences and courses, from the brazilian or international pharmaceutical, orthosis, prosthesis, equipment and implants industry: - Takeda. Any economically relevant equity interest in companies in the healthcare or education industry or in any companies competing with or supplying to SBC: - Health area.
Fernando Ribeiro de Moraes Neto	Financial declaration A - Economically relevant payments of any kind made to (i) you, (ii) your spouse/partner or any other person living with you, (iii) any legal person in which any of these is either a direct or indirect controlling owner, business partner, shareholder or participant; any payments received for lectures, lessons, training instruction, compensation, fees paid for participation in advisory boards, investigative boards or other committees, etc. from the brazilian or international pharmaceutical, orthosis, prosthesis, equipment and implants industry: - Edwards: Bioprosthesis, valve replacement. Other relationships Funding of continuing medical education activities, including travel, accommodation and registration in conferences and courses, from the brazilian or international pharmaceutical, orthosis, prosthesis, equipment and implants industry: - Edwards: continuing medical education.
Francisco Carlos da Costa Darrieux	Financial declaration A - Economically relevant payments of any kind made to (i) you, (ii) your spouse/partner or any other person living with you, (iii) any legal person in which any of these is either a direct or indirect controlling owner, business partner, shareholder or participant; any payments received for lectures, lessons, training instruction, compensation, fees paid for participation in advisory boards, investigative boards or other committees, etc. from the brazilian or international pharmaceutical, orthosis, prosthesis, equipment and implants industry: - Daichii Sankyo; Adium; Aché.
Guilherme Fenelon	Other relationships Funding of continuing medical education activities, including travel, accommodation and registration in conferences and courses, from the brazilian or international pharmaceutical, orthosis, prosthesis, equipment and implants industry: - Johnson & Johnson: catheter ablation.
Gustavo Glotz de Lima	Financial declaration A - Economically relevant payments of any kind made to (i) you, (ii) your spouse/partner or any other person living with you, (iii) any legal person in which any of these is either a direct or indirect controlling owner, business partner, shareholder or participant; any payments received for lectures, lessons, training instruction, compensation, fees paid for participation in advisory boards, investigative boards or other committees, etc. from the brazilian or international pharmaceutical, orthosis, prosthesis, equipment and implants industry: - Abbott: arrhythmias. Other relationships Funding of continuing medical education activities, including travel, accommodation and registration in conferences and courses, from the brazilian or international pharmaceutical, orthosis, prosthesis, equipment and implants industry: - Abbott: arrhythmias.
Jacob Atié	Financial declaration A - Economically relevant payments of any kind made to (i) you, (ii) your spouse/partner or any other person living with you, (iii) any legal person in which any of these is either a direct or indirect controlling owner, business partner, shareholder or participant; any payments received for lectures, lessons, training instruction, compensation, fees paid for participation in advisory boards, investigative boards or other committees, etc. from the brazilian or international pharmaceutical, orthosis, prosthesis, equipment and implants industry: - Boston Scientific, Johnson & Johnson: lectures on atrial fibrillation. Other relationships Funding of continuing medical education activities, including travel, accommodation and registration in conferences and courses, from the brazilian or international pharmaceutical, orthosis, prosthesis, equipment and implants industry: - Boston Scientific, Johnson & Johnson: FOR AF ablation.
João Carlos Ferreira Leal	Financial declaration A - Economically relevant payments of any kind made to (i) you, (ii) your spouse/partner or any other person living with you, (iii) any legal person in which any of these is either a direct or indirect controlling owner, business partner, shareholder or participant; any payments received for lectures, lessons, training instruction, compensation, fees paid for participation in advisory boards, investigative boards or other committees, etc. from the brazilian or international pharmaceutical, orthosis, prosthesis, equipment and implants industry: - Neomex/Artivion: prótese híbrida Evita open plus e On-X; Braile Biomédica: prótese de liberação rápida Alpha. B - Research funding under your direct/personal responsibility (directed to the department or institution) from the brazilian or international pharmaceutical, orthosis, prosthesis, equipment and implants industry: - Braile Biomédica: PI of the Vivere biological prosthesis study. C - Personal research funding paid by the brazilian or international pharmaceutical, orthosis, prosthesis, equipment and implants industry: - Braile Biomédica: PI of the Vivere biological prosthesis study. Other relationships Funding of continuing medical education activities, including travel, accommodation and registration in conferences and courses, from the brazilian or international pharmaceutical, orthosis, prosthesis, equipment and implants industry: - Neomex/Artivion; Braile Biomédica.
Jose Carlos Moura Jorge	Nothing to be declared
José Carlos Pachón Mateos	Nothing to be declared
José Marcos Moreira	Nothing to be declared
José Tarcísio Medeiros de Vasconcelos	Financial declaration A - Economically relevant payments of any kind made to (i) you, (ii) your spouse/partner or any other person living with you, (iii) any legal person in which any of these is either a direct or indirect controlling owner, business partner, shareholder or participant; any payments received for lectures, lessons, training instruction, compensation, fees paid for participation in advisory boards, investigative boards or other committees, etc. from the brazilian or international pharmaceutical, orthosis, prosthesis, equipment and implants industry: - Boston Scientific, Abbott. B - Research funding under your direct/personal responsibility (directed to the department or institution) from the brazilian or international pharmaceutical, orthosis, prosthesis, equipment and implants industry: - Daichi Sankyo. C - Financiamento de pesquisa (pessoal), cujas receitas tenham sido provenientes da indústria farmacêutica, de órteses, próteses, equipamentos e implantes, brasileiras ou estrangeiras: - Daichi Sankyo. Other relationships Funding of continuing medical education activities, including travel, accommodation and registration in conferences and courses, from the brazilian or international pharmaceutical, orthosis, prosthesis, equipment and implants industry: - Boston Scientific, Abbott.
Leandro Ioschpe Zimerman	Other relationships Participation in procurement committees for supplies or drugs in health institutions or any similar roles taken: - Head of the Cardiovascular Diagnostics and Therapy Unit at the Hospital de Clínicas de Porto Alegre; decision on the possible purchase of materials.
Luciana Sacilotto	Nothing to be declared
Luciana Vidal Armaganijan	Nothing to be declared
Luiz Pereira de Magalhães	Nothing to be declared
Luiz Roberto Leite da Silva	Other relationships Funding of continuing medical education activities, including travel, accommodation and registration in conferences and courses, from the brazilian or international pharmaceutical, orthosis, prosthesis, equipment and implants industry: - Biosense Webster: atrial fibrillation ablation.
Marcelo Garcia Leal	Nothing to be declared
Marcio Augusto Silva	Nothing to be declared
Marcio Jansen de Oliveira Figueiredo	Financial declaration A - Economically relevant payments of any kind made to (i) you, (ii) your spouse/partner or any other person living with you, (iii) any legal person in which any of these is either a direct or indirect controlling owner, business partner, shareholder or participant; any payments received for lectures, lessons, training instruction, compensation, fees paid for participation in advisory boards, investigative boards or other committees, etc. from the brazilian or international pharmaceutical, orthosis, prosthesis, equipment and implants industry: - Abbott: Ritmonorm; EMS: Xakylis.
Martha Valéria Tavares Pinheiro	Other relationships Funding of continuing medical education activities, including travel, accommodation and registration in conferences and courses, from the brazilian or international pharmaceutical, orthosis, prosthesis, equipment and implants industry: - Boston: Farapulse.
Mauricio Ibrahim Scanavacca	Financial declaration A - Economically relevant payments of any kind made to (i) you, (ii) your spouse/partner or any other person living with you, (iii) any legal person in which any of these is either a direct or indirect controlling owner, business partner, shareholder or participant; any payments received for lectures, lessons, training instruction, compensation, fees paid for participation in advisory boards, investigative boards or other committees, etc. from the brazilian or international pharmaceutical, orthosis, prosthesis, equipment and implants industry: - Daiichi Sankyo: anticoagulation. B - Research funding under your direct/personal responsibility (directed to the department or institution) from the brazilian or international pharmaceutical, orthosis, prosthesis, equipment and implants industry: - J&J: ventricular tachycardia ablation. C - Personal research funding paid by the brazilian or international pharmaceutical, orthosis, prosthesis, equipment and implants industry: - ABBOT: catheter ablation of vagal vessel cindame. Other relationships Funding of continuing medical education activities, including travel, accommodation and registration in conferences and courses, from the brazilian or international pharmaceutical, orthosis, prosthesis, equipment and implants industry: - J&J: Industry-sponsored symposium.
Maurício Pimentel	Financial declaration A - Economically relevant payments of any kind made to (i) you, (ii) your spouse/partner or any other person living with you, (iii) any legal person in which any of these is either a direct or indirect controlling owner, business partner, shareholder or participant; any payments received for lectures, lessons, training instruction, compensation, fees paid for participation in advisory boards, investigative boards or other committees, etc. from the brazilian or international pharmaceutical, orthosis, prosthesis, equipment and implants industry: - Libbs: atrial fibrillation.
Olga Ferreira de Souza	Nothing to be declared
Renato D. Lopes	Financial declaration A - Economically relevant payments of any kind made to (i) you, (ii) your spouse/partner or any other person living with you, (iii) any legal person in which any of these is either a direct or indirect controlling owner, business partner, shareholder or participant; any payments received for lectures, lessons, training instruction, compensation, fees paid for participation in advisory boards, investigative boards or other committees, etc. from the brazilian or international pharmaceutical, orthosis, prosthesis, equipment and implants industry: - Pfizer, Daiichi Sankyo, Novo Nordisk, Bayer, Boehringer Ingelheim, Bristol-Myers Squibb. B - Research funding under your direct/personal responsibility (directed to the department or institution) from the brazilian or international pharmaceutical, orthosis, prosthesis, equipment and implants industry: - Amgen, Bristol-Myers Squibb, GlaxoSmithKline, Medtronic, Pfizer, Sanofi-Aventis. Other relationships Funding of continuing medical education activities, including travel, accommodation and registration in conferences and courses, from the brazilian or international pharmaceutical, orthosis, prosthesis, equipment and implants industry: - Pfizer, Daiichi Sankyo, Novo Nordisk, Novartis.
Ricardo Alves da Costa	Nothing to be declared
Ricardo Ryoshim Kuniyoshi	Financial declaration A - Economically relevant payments of any kind made to (i) you, (ii) your spouse/partner or any other person living with you, (iii) any legal person in which any of these is either a direct or indirect controlling owner, business partner, shareholder or participant; any payments received for lectures, lessons, training instruction, compensation, fees paid for participation in advisory boards, investigative boards or other committees, etc. from the brazilian or international pharmaceutical, orthosis, prosthesis, equipment and implants industry: - Daiichi Sankyo: Lixiana; Boehringer Ingelheim: Pradaxa; Biotronik: arritmias.
Rui Manuel de Sousa Sequeira Antunes de Almeida	Financial declaration A - Economically relevant payments of any kind made to (i) you, (ii) your spouse/partner or any other person living with you, (iii) any legal person in which any of these is either a direct or indirect controlling owner, business partner, shareholder or participant; any payments received for lectures, lessons, training instruction, compensation, fees paid for participation in advisory boards, investigative boards or other committees, etc. from the brazilian or international pharmaceutical, orthosis, prosthesis, equipment and implants industry: - Meril Brasil: Dafodil. B - Research funding under your direct/personal responsibility (directed to the department or institution) from the brazilian or international pharmaceutical, orthosis, prosthesis, equipment and implants industry: - Meril Brasil: Dafodil.
Silvia Helena Cardoso Boghossian	Financial declaration A - Economically relevant payments of any kind made to (i) you, (ii) your spouse/partner or any other person living with you, (iii) any legal person in which any of these is either a direct or indirect controlling owner, business partner, shareholder or participant; any payments received for lectures, lessons, training instruction, compensation, fees paid for participation in advisory boards, investigative boards or other committees, etc. from the brazilian or international pharmaceutical, orthosis, prosthesis, equipment and implants industry: - Medtronic; Boston Scientific.
Tan Chen Wu	Nothing to be declared
Thais Aguiar do Nascimento	Nothing to be declared
Thiago da Rocha Rodrigues	Financial declaration A - Economically relevant payments of any kind made to (i) you, (ii) your spouse/partner or any other person living with you, (iii) any legal person in which any of these is either a direct or indirect controlling owner, business partner, shareholder or participant; any payments received for lectures, lessons, training instruction, compensation, fees paid for participation in advisory boards, investigative boards or other committees, etc. from the brazilian or international pharmaceutical, orthosis, prosthesis, equipment and implants industry: - Libbs.
Veridiana Silva de Andrade	Other relationships Funding of continuing medical education activities, including travel, accommodation and registration in conferences and courses, from the brazilian or international pharmaceutical, orthosis, prosthesis, equipment and implants industry: - Medtronic: Curso Micra.

## Table of Contents

**1. Introduction** 12
**2. Epidemiology** 13
**3. Definition and Classification** 14
  **3.1. Definition** 14
  **3.2. Classification** 14
**4. Pathophysiological Mechanisms** 18
  **4.1. Disease Progression** 18
  **4.2. Atrial Cardiomyopathy** 18
  **4.3. Predisposing Factors for Atrial Fibrillation** 19
    **4.3.1. Intrinsic Factors** 19
      ***4.3.1.1. Age and Sex*** 19
      ***4.3.1.2. Genetic Polymorphisms*** 19
    **4.3.2. Comorbidities** 20
      ***4.3.2.1. Arterial Hypertension*** 20
      ***4.3.2.2. Heart Failure*** 20
      ***4.3.2.3. Diabetes Mellitus*** 21
      ***4.3.2.4. Gastroesophageal Reflux*** 21
      ***4.3.2.5. Systemic Infections *** 21
      ***4.3.2.6. Other Conditions*** 21
  **4.4. Lifestyle Habits** 21
    **4.4.1. Obesity** 21
    **4.4.2. Sedentarism** 21
    **4.4.3. Poor Sleep Quality** 22
    **4.4.4. Alcohol Use** 23
    **4.4.5. Smoking** 23
    **4.4.6. Caffeine Use** 23
**5. Diagnosing Atrial Fibrillation** 23
  **5.1. Clinical Manifestations** 23
  **5.2. Impact on Quality of Life and Hospitalization** 25
  **5.3. Clinical Assessment of Patients with Atrial Fibrillation** 25
  **5.4. Screening Patients at Risk of Atrial Fibrillation** 25
  **5.5. Prolonged Electrocardiographic Monitoring** 27
**6. Atrial Fibrillation and Thromboembolic Events** 27
  **6.1. Algorithms for Assessing the Risk of Thromboembolic Events** 27
  **6.2. Algorithms for Assessing Bleeding Risk** 28
  **6.3. Preventing Thromboembolic Events** 28
    **6.3.1. Anticoagulants Available in Clinical Practice** 28
    **6.3.2. Individualized Anticoagulation Strategies** 29
  **6.4. Recommendations on Patients Treated with Anticoagulants** 30
    **6.4.1. Guidelines for Bleeding in Patients Treated with Direct-acting Oral Anticoagulants** 31
  **6.5. Anticoagulant Therapy in Special Situations** 33
    **6.5.1. Electrical Cardioversion** 33
    **6.5.2. Valvular Atrial Fibrillation** 34
      ***6.5.2.1. Oral Anticoagulation in Patients with Valvular Heart Disease in Native Valves*** 34
      ***6.5.2.2. Oral Anticoagulation in Patients with Moderate-to-Severe Mitral Stenosis*** 34
      ***6.5.2.3. Oral Anticoagulation in Patients with Valvular Prostheses*** 34
    **6.5.3. Anticoagulation in Acute Coronary Syndrome** 37
    **6.5.4. Perioperative Anticoagulation Management in Cardiac Ablation and Surgery** 38
    **6.5.5. Anticoagulation in Subclinical Atrial Fibrillation** 40
  **6.6. Left Atrial Appendage Occlusion and Anticoagulation** 41
    **6.6.1. Contraindications for Anticoagulant Therapy** 41
    **6.6.2. Percutaneous Left Atrial Appendage Occlusion** 41
    **6.6.3. Surgical Occlusion of the Left Atrial Appendage** 42
    **6.6.4. Anticoagulation after Left Atrial Appendage Occlusion** 43
      ***6.6.4.1. Anticoagulation after Percutaneous Left Atrial Appendage Occlusion*** 43
      ***6.6.4.2. Antithrombotic Therapy After Surgical Occlusion of the Left Atrial Appendage*** 45
  **6.7. Strategies to Minimize Bleeding Risk** 46
  **6.8. Choosing an Oral Anticoagulant** 47
**7. Rate or Rhythm Control in Atrial Fibrillation** 47
  **7.1. Antiarrhythmic Agents** 47
    **7.1.1. Propafenone** 47
    **7.1.2. Amiodarone** 47
    **7.1.3. Sotalol** 48
  **7.2. Rate Control Drugs** 48
    **7.2.1. Beta-blockers** 48
    **7.2.2. Calcium Blockers** 48
    **7.2.3. Digoxin** 48
  **7.3. Choosing between Rhythm and Rate Control** 49
  **7.4. Rate Control** 50
    **7.4.1. Therapeutic Goal** 50
    **7.4.2. Acute Control** 50
    **7.4.3. Long-Term Control** 50
    **7.4.4. Atrioventricular Node Ablation** 51
  **7.5. Rhythm Control – Special Situations** 52
    **7.5.1. Electrical Cardioversion** 52
    **7.5.2. Chemical Cardioversion** 52
      ***7.5.2.1. The "Pill-in-the-pocket" Approach*** 52
  **7.6. Pharmacological Sinus Rhythm Treatment** 53
**8. Atrial Fibrillation Ablation** 53
  **8.1. Mapping and Ablation Technologies** 53
    **8.1.1. Three-Dimensional Mapping Systems** 53
    **8.1.2. Intracardiac Echocardiography in Atrial Fibrillation Ablation** 53
    **8.1.3. Ablation Technologies** 55
  **8.2. Recommendations on Atrial Fibrillation Ablation** 57
    **8.2.1. Clinical Studies on Atrial Fibrillation Ablation** 57
  **8.3. Complications** 59
  **8.4. Main Complications** 59
    **8.4.1. Thermal Injury to the Esophagus** 59
    **8.4.2. Atrioesophageal or Esophago-Pericardial Fistula** 61
    **8.4.3. Gastroesophageal Motility Changes** 61
    **8.4.4. Cardiac Tamponade** 61
    **8.4.5. Systemic Thromboembolic Events** 62
    **8.4.6. Air Embolism** 62
    **8.4.7. Pulmonary Vein Stenosis** 62
    **8.4.8. Phrenic Paralysis** 62
    **8.4.9. Vascular Complications** 62
    **8.4.10. Other Complications** 63
  **8.5. Cryoablation** 63
    **8.5.1. Cryoablation Techniques for Atrial Fibrillation** 63
  **8.6. Catheter Ablation of Atrial Fibrillation in Patients with Heart Failure** 63
**9. Surgical Treatment for Atrial Fibrillation** 65
  **9.1. Recommendations on Surgical Treatment for Atrial Fibrillation** 66
    **9.1.1. Surgical Treatment for Atrial Fibrillation Concomitant with Other Types of Cardiac Surgery** 66
    **9.1.2. Isolated Atrial Fibrillation Surgery** 66
  **9.2. Surgical Techniques** 67
  **9.3. Long-Term Results and Complications** 67
  **9.4. Hybrid Procedures** 67
**10. Multidisciplinary Approaches to Atrial Fibrillation** 67
  **10.1. Implementing Lifestyle Change Programs** 67
**11. Special Situations in Atrial Fibrillation** 67
  **11.1. Intracranial Hemorrhage** 67
  **11.2. The Oldest Old Population** 68
  **11.3. Minority Groups** 68
  **11.4. Kidney Disease** 69
  **11.5. Atrial Fibrillation in Pregnancy** 70
  **11.6. Atrial Fibrillation (AF), the Parasympathetic Nervous System, and Cardioneuroablation** 70
  **11.7. Atrial Fibrillation in Athletes** 71
  **11.8. Arrhythmic Syndromes** 71
    **11.8.1. Arrhythmic Syndrome in Young People without Apparent Structural Heart Disease** 71
  **11.9. Atrial Fibrillation and Atrial Flutter in Adult Congenital Heart Disease** 72
  **11.10. Atrial Fibrillation in Women** 73
  **11.11. Atrial Fibrillation in Patients with Implantable Electronic Cardiac Devices** 74
    **11.11.1. Detection** 74
    **11.11.2. Programming Adjustments** 74
    **11.11.3. Cardioversion and Ablation** 75
  **11.12. Atrial Fibrillation After Cardiac and Non-Cardiac Surgeries** 75
    **11.12.1. Predictive Factors** 75
    **11.12.2. Comorbidities and Epidemiological Data** 75
      ***11.12.2.1. Surgical Predictors*** 76
    **11.12.3. Preoperative Prevention** 76
    **11.12.4. Postoperative Atrial Fibrillation Treatment** 76
**12. Atrial Fibrillation and Cognitive Changes** 76
**13. The Importance of Education for Patients with Atrial Fibrillation** 77
**14. Cost-benefit Analysis and Appropriate Resource Use in Atrial Fibrillation** 77

## 1. Introduction

Atrial fibrillation (AF) is the most common type of sustained arrhythmia seen in clinical practice. With the aging of the population, estimates of its prevalence and incidence are becoming increasingly alarming. It is estimated that the number of AF patients > 55 years of age will more than double by 2060.^1^ This could have a major impact on health systems in Brazil and worldwide, since stroke, the main consequence of AF, is a very debilitating condition. AF increases the chance of stroke by 400% on average and is associated with a higher risk of all-cause mortality and heart failure (HF).^2^

**Figure:** Care model and pillars of integrated atrial fibrillation treatment.

Numerous research groups have been investigating the pathophysiological mechanisms of AF and ways to improve both treatment and mapping and ablation systems. In parallel, there has been great progress in diagnostic tools for AF and long-term electrocardiographic (ECG) monitoring systems. New anticoagulants have assumed a prominent role in thromboembolic prevention, and left atrial appendage (LAA) occlusion is becoming increasingly accessible in care centers for patients with AF.

As a result, new guidelines are needed for clinical AF management, which has changed significantly in recent years. Multidisciplinarity is among the main changes. Cardiologists are accompanied by electrophysiologists, nephrologists, hematologists, nurses, physical therapists, and caregivers in clinical management. However, patients must be at the center of the decision-making process, supported by a team of specialists to individualize treatment according to their needs. Treatment must encompass a number of aspects, which can be summarized in 5 pillars of action required for optimal treatment (Central Illustration).

## 2. Epidemiology

AF, the most common type of recurrent arrhythmia seen in clinical practice, causes substantial morbidity and mortality in affected patients. It is independently associated with lower quality of life and increased risk of stroke, HF, and overall mortality.^3,4^

The incidence and prevalence of AF are increasing globally. According to the Framingham Study, the prevalence of AF has increased threefold in the last 50 years.^5^ It is estimated that the prevalence of AF in the United States will increase from 5.2 million in 2010 to 12.1 million by 2030.^6,7^ The standardized prevalence of AF is higher in high-income regions, such as North America and Australasia^6^ (Figure 1). In Europe, the prevalence of AF in adults > 55 years of age was 8.8 million in 2010 and is projected to reach 17.9 million by 2060.^8^

**Figure 1:** Global age-standardized prevalence of atrial fibrillation/atrial flutter per 100,000 in both sexes in 2020. Adapted from Tsao et al.^6^

These significant numbers indicate a major impact on population health. The total number of deaths attributed to AF and atrial flutter (AFL) in 2020 was 330,000 (130,000 men). Age-standardized mortality is highest in Western Europe and Australasia^6^ (Figure 2).

**Figure 2:** Global age-standardized mortality due to atrial fibrillation/atrial flutter in both sexes in 2020. Adapted from Tsao et al.^6^

The age-standardized prevalence of atrial AF/AFL in Brazil increased from 519 per 100,000 inhabitants in 1990 to 537 per 100,000 inhabitants in 2019 (3.5% variation).^9^ The ELSA-Brazil study investigated the frequency of AF and AFL according to electrocardiography (ECG), self-report, and drug therapy for stroke prevention in 13,260 patients. It was found that 2.5% of patients had AF/AFL and that non-adherence to anticoagulant therapy was common, especially among women.^10^ The EPISONO study assessed 767 participants (mean age, 42 years) using polysomnography and ECG monitoring during sleep, finding that the AF/AFL prevalence varied from 0.2% to 1.65% and that it was highest among patients with severe sleep apnea.^11^ However, another study found an AF/AFL prevalence of 2.9% in men and 2.0% in women among 1524 older residents of the city of São Paulo.^12^

According to robust epidemiological data, AF is now a major public health problem, especially in Brazil, which is undergoing rapid and significant population aging. This has led to increased public health care expenditures and consumption of health care resources, especially for potentially avoidable hospitalizations (Figure 3).

**Figure 3:** Hospitalizations related to clinical procedures for cardiovascular diseases in Brazil 2008-2019. Source: The Brazilian Unified Health System's Mortality Information System. Adapted from Oliveira et al.^9^

## 3. Definition and Classification

### 3.1. Definition

AF is defined as atrial arrhythmia with complete disorganization of atrial electrical activity and consequent ineffectiveness of contraction. In ECG, AF is characterized by irregular RR intervals (provided that atrioventricular conduction is preserved) and a lack of well-defined and sequential P waves, which have been replaced by fibrillatory waves in the baseline ECG, secondary to electrical disorganization of the atria (Figure 4).

**Figure 4:** Rhythm change shown in 12-lead Holter monitoring. The A and C beats are preceded by P waves, while the B beats are compatible with isolated atrial ectopia. The blue line indicates the point of rhythm change, including the lack of a P wave and RR irregularity. Figure provided by Dr. Fatima Dumas Cintra.

### 3.2. Classification

Once AF has been diagnosed, classifying it becomes extremely important, since this establishes diagnostic parameters, provides information about the arrhythmia burden, and indicates the extent of the disease. This information is also fundamental for the most appropriate therapy and provides homogeneous criteria for future clinical studies.

Initially, AF must be classified as clinical or subclinical (Table 1 and Table 2). With advancing technology and greater awareness of the importance of early diagnosis, active screening for these patients has expanded. Today, wearable or implantable devices are of great value in early detection,^13-15^ although it is extremely important to define clear criteria for correct AF diagnosis. To do this, the arrhythmia must be recorded in ECG, given that AF episodes must last ≥ 30 seconds to be considered clinical arrhythmia.^16^ Thus, regardless of the symptoms, AF is diagnosed as clinical if, through conventional 12-lead ECG monitoring or a single-lead ECG rhythm monitoring, a physician determines that it lasts ≥ 30 seconds.^17^ Recommendations on AF diagnosis are presented in Box 1.

**Table 1:** Atrial fibrillation classification parameters: based on presentation

Clinical AF	Symptomatic or asymptomatic AF recorded with 12-lead ECG or single-lead rhythm ECG lasting ≥ 30 s*.
Subclinical AF	Asymptomatic patients with accelerated atrial rhythm according to a CIED#

* ECG recordings, even those obtained by wearable devices, must be evaluated and certified by a qualified physician. #To reduce artifacts, an accelerated atrial rhythm is normally considered episodes (certified by a specialist) with a frequency ≥ 175 bpm. AF: atrial fibrillation; ECG: electrocardiogram; CIED: cardiac implantable electronic device.

**Table 2:** Atrial fibrillation classification parameters: based on duration

Paroxysmal AF	AF lasting < 7 days (regardless of the reversal method)
Persistent AF	AF lasting > 7 days but < 1 year
Long-standing persistent AF	AF lasting > 1 year
Permanent AF	AF cases in which the doctor and patient decide not to reestablish sinus rhythm regardless of onset time
Post-ablation AF	Non-recurrence of arrhythmia after catheter or surgical ablation

**Box 1:** Recommendations on diagnosing atrial fibrillation

	Class of recommendation	Level of evidence
Electrocardiographic documentation is necessary to diagnose AF	**I**	**C**
Clinical AF must be diagnosed by 12-lead ECG or single-lead monitoring, finding P wave and RR irregularity ≥ 30 seconds	**I**	**C**

However, subclinical AF occurs in asymptomatic patients. Accelerated atrial rhythm can be determined through cardiac implantable electronic device (CIED) data, or readings suggestive of AF can be provided by wearable devices.^17^ Because clinical studies vary widely regarding atrial heart rate (HR) cut-off points (between 160 and 200 bpm) and episode duration (10 seconds to 24 hours), it is difficult to define precise HR parameters and episode duration for accelerated atrial rhythm. CIEDs generally classify HR ≥ 175 bpm for ≥ 5 minutes as accelerated atrial rhythm to avoid recording artifacts.

In fact, technological advances in detecting AF and episodes of accelerated atrial rhythm, through CIEDs, implantable event monitors, or wearable devices, have substantially increased clinical and subclinical AF diagnosis. Clinical validation studies using ECG have found that wearable devices have a sensitivity of 67.7%-100% and specificity of 60.7%-100% for diagnosing AF.^18^ The fact that such devices, especially in association with smartphones, are becoming increasingly accessible represents both an opportunity and a challenge in AF monitoring. The main challenges include the need to define which groups should be monitored, the best device to use, how to access health services, and what actions should be taken based on device data.^20^ Another issue is that such patients have not been included in the large clinical studies on which current AF treatment is based. Therefore, until the best therapeutic options for subclinical AF have been discovered, these cases must be handled with caution and analyzed individually in their clinical context to determine the most appropriate therapy (see item 6.5.4).^21^ Figure 5 describes the characteristics of clinical and subclinical AF.

**Figure 5:** The overlap between clinical and subclinical atrial fibrillation for treatment decision-making. AF: atrial fibrilation.

Another important factor in AF is duration (Table 1 and Table 2) which, in association with other clinical factors, is used to determine the chance of reestablishing and/or maintaining sinus rhythm.^17,22^ Thus, AF can be classified as: paroxysmal (< 7 days, regardless the means of reversal, ie, spontaneous, chemical, or electrical); persistent (> 7 days but < 1 year, regardless of whether spontaneous, chemical, or electrical reversal occurs); long-standing persistent (> 1 year, including application of a rhythm control strategy for reversal and sinus rhythm maintenance); or permanent (ie, regardless of the duration, the doctor and patient agree not to attempt to reestablish sinus rhythm). In the latter type, if rhythm control is reconsidered at any point, the AF will be re-classified as long-standing persistent. Patients presenting both paroxysmal and persistent AF, should be classified according to the most prevalent form.

Some widely used terms to classify AF in clinical practice should be abandoned because they do not provide correct and/or relevant information for follow-up. One example is "isolated/solitary AF", since, even when no pathology is present, all AF has a cause, such as an intrinsic electrical disorder.^23^ Another outdated term is valvular and non-valvular AF.^9^ The current recommendation is to specify the patient's problem, identifying, for example, moderate-to-severe mitral stenosis or mechanical valve prostheses, and measures are to be taken in accordance with recommendations on the specific clinical condition. Finally, the term "chronic AF", which is nonspecific, adds nothing to clinical management and, hence, should not be used.

American guidelines present a classification system based on disease progression. This initiative is important because it includes patients who have undergone successful ablation and have had no clinical recurrence. This group of patients, which is becoming increasingly common in clinical practice, were not formerly represented. Although these patients no longer present AF, they are still at risk of new events and require different therapeutic measures, such as lifestyle modification, monitoring for symptomatic and asymptomatic AF episodes, and appropriate anticoagulant therapy. Figure 6 outlines the AF stages described in the American guidelines.^24^

**Figure 6:** Stages of atrial fibrillation Adapted from Joglar et al.^24^ AF: atrial fibrillation.

## 4. Pathophysiological Mechanisms

The pathophysiology of AF onset and maintenance involves a complex interrelationship between triggers, modulating factors, and electrophysiological, hemodynamic, and structural changes in the atrial myocardium (substrate) (see Figure 7

**Figure 7:** Pathophysiology of atrial fibrillation. Adapted from Cintra et al.^25^

The main hypothesis for AF onset and maintenance involves ectopic foci as an arrhythmia trigger and reentry as a factor in maintenance.^26^ Although most ectopic foci originate in the pulmonary veins, foci originating in the superior vena cava, Marshall ligament, coronary sinus, LA wall, and the crista terminalis have also been described.

The pulmonary veins play a fundamental role in AF onset and maintenance. The pulmonary veins include cardiomyocytes that extend from the LA, which have electrophysiological and anatomical properties that facilitate the occurrence of arrhythmia. The molecular basis for ectopic foci is mainly attributed to calcium flow abnormalities.^27^ The fibers in these cardiomyocytes have different orientations, with little lateral coupling, which favors areas of fibrosis and local disarray, thus increasing susceptibility to ectopic foci and reentry phenomena.^26^

The occurrence of reentrant circuits in conductive atrial substrate is the main mechanism of sustained AF. Structural changes characterized by atrial dilation, changes in the interstitial matrix, and fibrosis alter normal conduction and reduce atrial refractoriness, thus enabling the occurrence of multiple simultaneous reentry waves, facilitating sustained AF. Autonomic activity also plays an important role in the pathophysiology of AF. Combined sympathetic and vagal activation can make the atrial substrate even more prone to arrhythmia onset and maintenance^28,29^ (Figure 8).

**Figure 8:** Conceptual model of the arrhythmogenic mechanism of atrial fibrillation based on the pathophysiological pillars of atrial substrate, precipitators, and triggers.

### 4.1. Disease Progression

AF has a natural history of progression (Figure 6).^27^ Registry data show that progression from paroxysmal to persistent AF is 8.6% at 1 year and 24.7% at 5 years.^28^ Electrical remodeling and continued exposure to predisposing factors determine the progressive nature of the arrhythmogenic substrate.

### 4.2. Atrial Cardiomyopathy

Atrial cardiomyopathy has been defined as "structural, architectural, contractile, or electrophysiological changes affecting the atria with the potential to produce relevant clinical manifestations".^30^ The conceptualization of atrial cardiomyopathy is an attempt to better understand the relationship between the atrial myocardial substrate and its role in arrhythmogenesis and the occurrence of thromboembolic phenomena. Atrial cardiomyopathy is defined histopathologically, but changes indicative of this condition can be recognized through non-invasive methods, such as ECG (interatrial block, P wave prolongation, P wave terminal force in V1), echocardiogram (increased atrial volume), resonance (atrial volume, late atrial enhancement), or through invasive methods, such as low voltage areas in electroanatomical mapping.^31^

From this perspective, AF can be understood as the clinical manifestation of an altered myocardium rather than an independent arrhythmic event. Atrial cardiomyopathy is associated with an increased risk of thromboembolic events, regardless of clinical history of AF.^32,33^ Furthermore, signs of more advanced atrial cardiomyopathy are associated with a lower AF ablation success rate.^31^ Thus, recognizing atrial cardiomyopathy is becoming increasingly essential in clinical decision-making regarding AF treatment and thromboembolic event prevention.

### 4.3. Predisposing Factors for Atrial Fibrillation

In addition to intrinsic factors, such as age, sex, and genetic influence, comorbidities and lifestyle habits can contribute to AF onset. Such factors help form the atrial arrhythmogenic substrate that originates and sustains this tachyarrhythmia. The most recent evidence indicates that risk factor treatment, including lifestyle changes, is a fundamental component of AF prevention and treatment.^27,34-36^ Recommendations on AF risk factor control are shown in Box 2.

**Box 2:** Atrial fibrillation risk factor control

Recommendations	Class of recommendation	Level of evidence
Treating SAH through strict blood pressure control is recommended to reduce the risk of AF recurrence, the risk of stroke, and the progression of the disease	**I**	**A**
Every patient with AF and HF should receive optimized HF treatment	**I**	**A**
Treating diabetes and adequately controlling blood glucose can reduce the recurrence of AF and the progression of the disease	**I**	**C**
In patients with AF and gastroesophageal reflux disease, appropriate reflux treatment may reduce the risk of AF	**I**	**B**
A reduction of at least 10% in body weight is recommended for patients with obesity or those living with excess weight who have AF	**I**	**B**
When there are no contraindications, regular physical activity is recommended to prevent AF	**I**	**B**
The reduction of alcohol consumption (less than 30 grams per week) is recommended to reduce the recurrence of AF	**I**	**B**
Clinical investigation of sleep-disordered breathing is recommended in patients with AF; however, the benefits of CPAP treatment are uncertain	**IIa**	**B**
High-performance exercise should not be recommended to prevent AF	**III**	**B**

AF: Atrial fibrillation; HF: heart failure; SAH: systemic arterial hypertension; CPAP: continuous positive airway pressure.

#### 4.3.1. Intrinsic Factors

##### 4.3.1.1. Age and Sex

The incidence of AF increases with age, doubling every decade.^27,37^ However, some authors question this relationship, stating that due to increased longevity, detection is now more probable in older individuals. Other factors must also be considered, including greater demand for medical care due to health concerns and increased access to intermittent arrhythmia detection methods.^5^ Most epidemiological studies on AF are based on hospital records or the electronic databases of medical insurance services, which are subject to bias, leading to wide variation in estimates of AF incidence and prevalence. No population study with long-term follow-up has confirmed a relationship between advanced age and AF.

Epidemiological concepts aside, clinical data suggest an association between aging and AF. The influence of age as a risk determinant may be related to longer exposure to cardiovascular risk factors that somehow compromise atrial electrical activity. Men are 1.5 times more likely to experience AF, although the incidences for men and women are similar after 60 years of age.^35^

##### 4.3.1.2. Genetic Polymorphisms

Gap junctions formed by structural transmembrane proteins called connexins are the intercellular pathway responsible for rapid transmission of the action potential and electrical coupling of the heart. Abnormal electrical coupling caused by changes in the distribution of connexins has been associated with greater susceptibility to AF. Mutations or polymorphisms in 4 connexin isoforms have already been associated with AF.^38^ The fact is that more than 160 mutations have already been implicated in AF onset in recent decades, including mutations in structural proteins, as well as in ion channels and transcription factors.^39^

Lipoprotein A, a recognized risk factor for cardiovascular arteriosclerosis, independent of diet, has also recently been implicated as a mediator of AF, suggesting that this lipoprotein has far-reaching deleterious effects, thus corroborating genetic vulnerability to the development of AF.^40^

#### 4.3.2. Comorbidities

##### 4.3.2.1. Arterial Hypertension

Arterial hypertension may be the most important risk factor for AF due to its high prevalence in the general population. The relative risk (RR) of AF onset in hypertensive patients varies from 20% to 50%. One study indicated that systemic arterial hypertension (SAH) alone is responsible for 14% of all cases of AF.^41^ Differential pulse pressure and slightly increased systolic pressure values (130-139 mm Hg) are more associated with AF than systolic pressure < 120 mm Hg.^42^ The risk of arrhythmia increases by 26% for every 20 mm Hg increase in systolic pressure, which is of great clinical importance since it indicates that strict blood pressure control is essential for treatment. It should also be noted that AF recurrence increases when blood pressure rises due inadequate treatment.

Clinical studies indicate that effective blood pressure control associated with reduced left ventricular (LV) hypertrophy according to Cornell ECG criteria reduces AF incidence.^43-45^ According to the SPRINT trial, intensive SAH treatment (target SBP < 120 mm Hg) leads to a 26% greater reduction in AF risk than standard treatment (target SBP < 140 mm Hg).^46^ In Japan, lower hypertension rates have been associated with a significant reduction in AF incidence, particularly among older adults.^47^ These findings indicate that controlling hypertension and, consequently, reducing target organ damage (such as LV hypertrophy), reduces the risk of AF. Furthermore, by analyzing the Sokolow and Cornell criteria for LV hypertrophy, ECG can be a useful tool for determining clinical improvement.

##### 4.3.2.2. Heart Failure

HF increases the risk of AF by 4 to 6 times.^27,35^ Higher incidence is associated with worsening functional level and patient symptoms: it is 5%-10% in New York Heart Association functional class I, 10%-26% in classes II and III, and 40%-50% in class IV.^41,48^ These data indicate that more decompensated patients, those with greater degrees of systolic ventricular dysfunction, enlarged atria, and a greater degree of sympathetic effect are the most affected.^49^

Isolated diastolic dysfunction is also associated with an increased incidence of AF, possibly reflecting shared risk factors, including advanced age and high blood pressure.^50^ In such situations, increased end ventricular pressure is reflected in the atria, causing them to distend and dilate.^51^

AF occurs secondary to several factors in HF, including a high frequency of atrial ectopic beats, atrial dilation, hypervolemia, and increased autonomic tone (sympathetic hyperactivity). The fact that it occurs in more decompensated patients and, thus, in those with more intense adrenergic activity (substrate-stabilizing factor), supports this idea. Thus, clinical compensation can improve hemodynamic conditions and restore normal heart rhythm. Knowing that AF onset occurs with clinical worsening of HF, prevention consists of aggressively treating congestion. In such cases, primary arrhythmia prevention plays a preponderant role.

##### 4.3.2.3. Diabetes Mellitus

An American study found that diabetes is an independent risk factor for AF, with the risk being twice as high in younger patients.^52^ Diabetes doubles the risk of AF, regardless of other comorbidities.^53^ The adverse effects of diabetes on various systems may also contribute to a higher risk of AF, which occurs in up to 40% of patients with high glycemic levels. A higher risk of AF is associated with diabetes incidence and duration, evinced by high levels of glycated hemoglobin.^54^ Thus, it can be concluded that sustained high glycemic levels are the main risk factors for AF in this endocrinopathy. Diabetes is often associated with other AF risk factors, such as obesity, sleep apnea, and high blood pressure, which increase its impact.

The emergence of autonomic dysfunction in diabetes mellitus, known as cardiac autonomic neuropathy, increases the risk of atrial arrhythmias, which involve increased sympathetic activity and reduced parasympathetic activity. Morbidity in type II diabetes patients with cardiac autonomic neuropathy varies between 34% and 44%, demonstrated through RR interval variability analysis.^54^

Maintaining normal blood glucose levels, including low glycated hemoglobin levels, reduces the risk of AF in that it reduces the risk of cardiac autonomic neuropathy and the systemic inflammatory process caused by diabetes.

##### 4.3.2.4. Gastroesophageal Reflux

Gastroesophageal reflux is associated with AF by causing esophageal irritation directly over the LA.^55,56^ Vagal response appears to be another predisposing factor for AF, particularly in individuals with normal hearts and athletes (eg, marathon runners). Isolated case reports have shown that proton pump inhibitors can reduce the risk of AF in affected patients.^57^

Hiatal hernias can also trigger AF episodes due to their mechanical effects, compressing the atrial wall and causing extrasystoles. Since this condition is more common in older patients, other confounding (eg, hypertension, obesity, diabetes) may predispose patients to AF. However, some studies have shown a reduction in AF episodes after hernia repair surgery, which suggests a direct cause and effect relationship.^57^

##### 4.3.2.5. Systemic Infections

Studies have found an association between systemic infections (eg, gastrointestinal, respiratory, and urinary) with a higher risk of AF, as well as a higher risk of future complications during hospitalization and after discharge.^58,59^ Systemic infections predispose individuals to acute AF episodes, probably in those already predisposed to AF, which highlights their extraordinary clinical importance due to the patients’ severe clinical course after discharge. It should be pointed out that in infectious conditions, oxidative stress and inflammatory cytokines precipitate instability in reentry circuits in an already remodeled LA, giving rise to AF.

##### 4.3.2.6. Other Conditions

Numerous other conditions can predispose individuals to AF. Mitral valve disease, especially moderate or severe mitral stenosis, often accompanies changes in heart rhythm. Other conditions associated with pulmonary hyperflow, while interatrial septal defects are associated with an increased occurrence of AF. Even after treatment, a residual shunt is also associated with a greater likelihood of AF.^60^ Other non-cardiac conditions, such as hyperthyroidism, kidney disease, and chronic obstructive pulmonary disease also favor AF (Figure 9).

**Figure 9:** Risk factors for atrial fibrillation.

### 4.4. Lifestyle Habits

The high number of AF cases observed in clinical practice cannot be explained exclusively by population aging: other risk factors play an important role in onset. Recently, lifestyle factors have been associated with worse outcomes in AF, offering new opportunities for better clinical control through lifestyle change.

#### 4.4.1. Obesity

Obesity has increased significantly in Brazil and worldwide. Its prevalence increased from 11.8% in 2006 to 20.3% in 2019, and the estimated prevalences of overweight and obesity in Brazil for 2030 are 68.1% and 29.6%, respectively.^61^ An important meta-analysis of 626,603 individuals found that the risk of AF increased by 29% for every 5-unit increase in body mass index.^62^ A sub-analysis of RE-SPECT ESUS, a randomized, double-blind trial on secondary stroke prevention in patients with embolic stroke of undetermined origin, found that obese individuals with AF have a higher risk of stroke, thromboembolism, and death.^63^ This association can also be explained by genetics: a study of more than 50,000 individuals found that genetic variants associated with high body mass index correlate with AF incidence, suggesting a causal relationship between the conditions.^64^

Several prospective studies have been conducted to evaluate the effect of weight reduction on the occurrence of AF.^65-69^ The LEGACY study followed 355 patients for 4 years, dividing them into 3 groups according to weight loss at the end of the study. The probability of being arrhythmia-free was 6 times greater among participants who lost (and maintained) > 10% of their body weight than among those who lost < 3% or gained weight during the study period.^66^ Another prospective observational study of 281 AF ablation patients, 149 of whom had a body mass index > 27 kg/m^2^ and underwent risk factor management (individualized weight loss program), found that risk factor management led to greater drug-unassisted arrhythmia-free survival than the control group.^65^ Therefore, maintaining a healthy weight appears to help prevent AF and reduce AF recurrence. However, some studies have found that low lean mass can be associated with AF,^70^ and weight loss alone, without comprehensive risk factor control, may be ineffective in preventing recurrence.^71^

#### 4.4.2. Sedentarism

According to the CARDIO-FIT study, for each metabolic equivalent acquired during the protocol, there was a 9% reduction in arrhythmia recurrence, even after correction for weight and other risk factors.^72^ Furthermore, regular physical activity has been found to improve exercise capacity, quality of life, and LV ejection fraction (EF) in patients with AF.^73^ More recently, it was demonstrated that 1 year of aerobic training improves endothelial function and is associated with reduced thrombogenic and pro-inflammatory markers in patients with AF.^74^

However, the relationship between physical activity and AF seems to be a U- curve rather than a linear pattern, ie, extreme conditions, whether sedentarism or strenuous exercise, increase the risk of AF.^75,76^ It should be pointed out that only a very small percentage of the population exercises beyond recommended amounts, although the threshold between healthy and harmful amounts is still controversial.^77^ An ongoing clinical trial on the detraining of high-performance athletes with AF may help clarify the relationship between exercise and AF.^78^

#### 4.4.3. Poor Sleep Quality

In a national epidemiological study, the occurrence of nocturnal cardiac arrhythmia was more frequent in patients with severe obstructive sleep apnea (OSA), defined as an apnea/hypopnea index > 30 events/hour.^11^ Several studies have found a high co-occurrence of OSA and AF, ranging between 60% and 82%.^79,80^ In fact, these conditions share risk factors, although demonstrating causality has proven difficult. Nevertheless, a prospective study of patients referred for electrical cardioversion of atrial AF/AFL found recurrence rates of 82% and 42% in patients with untreated and treated OSA, respectively (p = 0.013).^81^ Furthermore, in the untreated group, recurrence was even greater among those with a greater drop in oxygen saturation during an apnea event (p = 0.034). Treating OSA reduces the risk of AF recurrence not only in patients who undergo electrical cardioversion but after catheter ablation, as well. In an observational study of 426 patients who underwent electrical pulmonary vein isolation, OSA was confirmed by polysomnography in 62: a continuous positive airway pressure group (n = 32) and a control group that received no treatment (n = 30). The continuous positive airway pressure group had greater AF-free survival than the control group (71.9% vs. 36.7%; p = 0.01). The authors concluded that continuous positive airway pressure therapy in patients with OSA who undergo percutaneous AF treatment reduces arrhythmia recurrence, while in AF cases with untreated OSA, electrical isolation has little clinical value.^82,83^ A meta-analysis on the role of OSA in patients with AF who undergo catheter ablation concluded that OSA is associated with a higher risk of AF recurrence after ablation (RR 1.25; p = 0.003).^84,85^

On the other hand, a small randomized study aimed at evaluating AF recurrence and burden after electrical isolation of the pulmonary veins did not show any benefit of using CPAP compared to the control group.^86^ This guideline recommends adequate sleep evaluation in patients with AF; however, randomized studies are needed to clarify the role of CPAP in the prevention and treatment of AF.^87^

#### 4.4.4. Alcohol Use

The effects of alcohol on atrial remodeling and the autonomic nervous system may, in part, explain higher AF recurrence among alcohol consumers. Of note, alcohol abstinence is associated with reduced arrhythmia recurrence.^88^ A meta-analysis of 9 observational studies on patients undergoing catheter ablation of AF (N = 5436) found that moderate-to-high alcohol consumers had a higher risk of recurrence.^89^ A multicenter prospective randomized trial in Australian hospitals included patients with paroxysmal or permanent AF who consumed > 10 drinks each week and were in sinus rhythm at baseline. The sample (N = 140) was divided 1:1 into abstinence and habitual use groups. AF recurrence was 53% in the abstinence group and 73% in the control group. The time to first recurrence was longer in the abstinence group, and the total number of events after 6 months of follow-up was significantly lower in the abstinence group.^90^

#### 4.4.5. Smoking

Smoking has recently been associated with AF (hazard ratio [HR], 1.51-2.05), in a dose-response relationship (highest risk in the highest tertile: > 675 pack-years).^91-93^ Smoking cessation reduces the risk of AF: a study of 15,792 patients found that the risk of AF was reduced by 36% among those who stopped smoking.^91^

The mechanisms involved in AF onset probably include the inflammatory role of nicotine, increased sympathetic activity, acute blood pressure elevation, myocardial hypoperfusion secondary to coronary artery disease, and chronic obstructive pulmonary disease. Persistent smoking is also associated with greater AF recurrence after catheter ablation of the pulmonary veins, and inflammation from nicotine may be involved in this process.^94^ Smoking cessation can reduce the harmful effects of nicotine on atrial electrical activity and improve AF outcomes.^95^

#### 4.4.6. Caffeine Use

The relationship between AF and coffee consumption is under investigation, although, to date, no studies have demonstrated an association between caffeine consumption and AF. On the contrary, an analysis of 2 Spanish cohorts found that intermediate coffee consumers (1 to 7 cups a week) had a lower risk of AF.^96^ A prospective study found similar results in a sample of 18,960 participants, ie, drinking 1 to 3 cups of coffee a day was associated with a lower risk of AF.^97^ A recent meta-analysis also failed to find that coffee consumption increases the occurrence of AF.^98^

## 5. Diagnosing Atrial Fibrillation

AF diagnosis depends on ECG recording of arrhythmia characterized by undetectable sinus P waves, irregular RR cycles (with preserved atrioventricular [AV] conduction), uncoordinated atrial electrical activity, and cycles between atrial deflections corresponding to depolarization (fibrillatory waves) usually < 200 ms.^16^ The often asymptomatic and intermittent nature of AF can make diagnosis difficult. Because arrhythmia is not always detected on successive office or emergency room ECGs, prolonged ambulatory ECG monitoring is necessary when AF is suspected. The available methods of AF screening include 24-h Holter monitoring (1 to 7 days), external loop event monitoring (1 to 4 weeks), wearable devices (smartwatches and smartphone applications), in addition to CIEDs, such as event monitors and artificial cardiac pacemakers.^20^ When a rapid atrial rhythm is documented, the arrhythmia must be recorded by ECG, which must be confirmed by a physician, understanding that an AF episode must last ≥ 30 seconds to be considered clinical AF (see Section 3.2).

Subclinical AF involves asymptomatic episodes of accelerated atrial rhythm recorded by a CIED. To reduce artifacts, accelerated atrial rhythm is normally considered ≥ 175 bpm and must be evaluated and certified by a specialist, as shown in Figure 10.

**Figure 10:** Accelerated atrial rhythm recorded by an implantable electronic cardiac device. The atrial canal (arrow) shows a very fast, irregular atrial signal compatible with AF. Figure provided by Dr. Thiago da Rocha Rodrigues.

AF burden is determined through continuous ECG monitoring as the total time AF is detected during a specific period. Most studies are based on a 24-hour monitoring period.^99^

### 5.1. Clinical Manifestations

AF can involve a wide spectrum of clinical manifestations (Table 3). Some symptoms are subtle and easily overlooked, while others can significantly affect the patient's quality of life and pose serious health risks. Patients with paroxysmal AF tend to report more symptoms (80%) than those with permanent AF (51%), with the latter group more frequently reporting dyspnea, fatigue, and exercise intolerance.^100^

**Table 3:** Summary of clinical manifestations related to AF

Palpitations
Fatigue
Dyspnea and heart failure
Chest discomfort and/or pain
Dizziness and syncope
Stroke and systemic thromboembolism
Exercise intolerance
Anxiety and depression
Cognitive decline and risk of dementia
Hospitalization and worsening quality of life
Increased mortality
Other manifestations (polyuria, nocturia, symptoms related to other comorbidities: hyperthyroidism, sleep apnea, autoimmune, systemic diseases, etc.)

Palpitations are the most commonly reported AF symptom.^101^ Patients often describe a rapid, irregular pulse in the precordial region, sometimes feeling "like something is shaking in my chest," which may be intermittent or persistent. Palpitations may be associated with anxiety, dizziness, or dyspnea.^101,102^ In patients who do not complain of palpitations, AF symptoms are generally more subtle, especially in older adults with conduction system dysfunction and AF with a slow ventricular response.^103^ Asymptomatic AF has been associated with less favorable prognosis. This apparently paradoxical finding is due to the fact that recognizing the arrhythmia allows for therapeutic measures that enable better clinical control, as well as a lower rate of complications.^104^

Fatigue and generalized weakness are frequent in patients with AF. Both irregular rhythm and rapid ventricular response can decrease cardiac output, leading to reduced tissue oxygen supply and a feeling of tiredness. AF can cause dyspnea, especially during physical exertion. The irregularity of atrial contractions compromises effective ventricular filling, leading to decreased cardiac output. Patients may experience insidious-onset dyspnea that worsens with even minimal exertion. Around 30% to 40% of patients with AF may develop HF at some point during diagnosis or treatment; likewise, around 30% to 40% of patients with HF may develop AF.^104,105^ The incidence of HF with preserved ejection fraction (HFpEF) was found to be twice as high in patients with AF than in those without AF.^106^ Elevated atrial natriuretic peptide levels, frequently found in AF, may reflect the co-occurrence of HFpEF.^107^ However, chronically elevated HR can result in tachycardiomyopathy, a condition of worsening LV systolic function. Initially, this dysfunction may be asymptomatic, but persistently elevated HR promotes progressive worsening of LVEF, leading to HF and dilated cardiomyopathy. Differential diagnosis between AF secondary to tachycardiomyopathy and AF in pre-existing HF is important. In general, tachycardiomyopathy shows normalization of LVEF after arrhythmia reversal. Additionally, elevated biomarkers such as BNP are more common in patients with primary HF.

Angina may occur in some patients with AF and, although the arrhythmia itself is not directly responsible for myocardial ischemia, the co-occurrence of coronary artery disease may contribute to chest pain.^108^ Furthermore, rapid ventricular rate associated with AF may increase myocardial oxygen demand, exacerbating angina symptoms.

Episodes of dizziness or pre-syncope are common manifestations of AF. Reduced cardiac output resulting from rapid and irregular heart rhythms can lead to cerebral hypoperfusion, causing dizziness. In severe cases, inadequate blood flow to the brain can result in syncope, especially in patients with diastolic dysfunction. Furthermore, AF associated with sinus dysfunction or an atrioventricular conduction disturbance can cause significant bradyarrhythmia that alternates with tachycardia (known as tachy-brady syndrome), or it can result in extremely low HR with advanced or total atrioventricular block, which is often associated with antiarrhythmic drug (AAD) treatment.

A concern in AF is the increased risk of stroke and peripheral systemic thromboembolism. Chaotic atrial activity promotes blood stasis in the atria, causing predisposition to thrombus formation, which can lead to embolism, especially in the brain, resulting in ischemic stroke. Around 15% to 30% of all ischemic strokes are caused by AF. Strokes in patients with AF tend to be more serious and debilitating than those of other etiologies.^109^ A meta-analysis that evaluated thromboembolic events related to AF demonstrated that the incidence of systemic thromboembolism during follow-up was lower than that of cerebral embolism (0.24 vs 1.92/100 person-years, comprising 12% of all clinical thromboembolic events, but with a comparable risk of death), as well as ischemic stroke. Around 60% of these events involve the lower extremities and 30% involve visceral/mesenteric systems, while only 11% involve the upper extremities.^110^

Patients with AF may have reduced exercise tolerance. This symptom is more frequent among athletes who develop AF,^111^ especially high-performance athletes. Irregular heart rhythm and inefficient HR response can limit the heart's ability to respond to increased metabolic demand during physical activity. Consequently, individuals with AF may quickly become fatigued or unable to exercise at levels they previously tolerated, almost always reporting symptoms such as "loss of yield". Another common occurrence is a disproportionate increase in HR during effort due to increased atrioventricular conduction, which is related to greater adrenergic stimulus.

The chronic nature of AF, including associated symptoms and complications, can have a significant impact on a patient's mental well-being.^112^ Anxiety and depression are commonly observed in individuals with AF, often due to the disruptive nature of the arrhythmia, fear of complications, and limitations in daily activities. Cognitive decline and dementia may also occur. Studies indicate that chronic cerebral hypoperfusion, microembolisms and/or microbleeds, as well as inflammation and white matter lesions, are factors in AF-related dementia. Cognitive decline can occur regardless of previous clinical stroke, although a family history of early-onset dementia is not uncommon in these patients.^113,114^

AF may occasionally be associated with polyuria and nocturia, especially after arrhythmia reversal, due to the release of atrial natriuretic peptide.

### 5.2. Impact on Quality of Life and Hospitalization

AF is associated with a significant increase in hospitalization rates worldwide,^115^ and reduced quality of life has been associated with a higher risk of hospitalization.^116^ The clinical impact of AF can range from completely asymptomatic to extremely debilitated patients, whose quality of life is similar to other heart diseases. Dorian et al. found that patients with AF had lower quality of life in all domains of the Short-Form 36 questionnaire than healthy individuals or post-angioplasty or post-myocardial infarction patients.^117^ Furthermore, psychological characteristics, coronary artery disease, and diabetes may play an important role in identifying individuals at greater risk of impaired quality of life and more severe AF-related symptoms.^118^ Psychosocial factors, such as anxiety, sleep disorders, work-related stress and noise level, should also be considered contributors to AF-related symptom severity.^119^

A quality of life questionnaire was developed and validated specifically for patients with AF in Brazil, addressing its distinctive symptoms and treatment.^120,121^ In addition to reducing morbidity and mortality, quality of life should also be considered a therapeutic objective in AF.

### 5.3. Clinical Assessment of Patients with Atrial Fibrillation

Patients with suspected or diagnosed AF must be clinically evaluated to characterize the arrhythmia, estimate time since onset, and determine the frequency and duration of episodes, HR behavior, associated symptoms, and their impact on quality of life and everyday functioning. Furthermore, the occurrence of other cardiovascular symptoms, such as dyspnea, chest pain, and pre-syncope or syncope, associated or not with arrhythmia, may reveal the clinical repercussions, as well as raise suspicion of co-occurring heart diseases. Identifying predisposing factors for AF, such as SAH, HF, diabetes mellitus, CAD, obesity, OSA, sedentarism, alcohol consumption, hyperthyroidism, etc., is fundamental for determining subsequent therapeutic strategies. Assessing the patient's cardiovascular history and identifying structural heart diseases helps define the pathophysiological substrate of the arrhythmia and determine the appropriate treatment. Research into thromboembolic history, especially ischemic stroke, risk factors, and bleeding risk, are a fundamental part of the initial assessment.

Signs of cardiovascular change should be assessed in the physical examination, such as jugular distension, peripheral edema, pulmonary congestion, irregular pulse, changes in heart sounds, murmurs, hypotension, and arterial hypertension. In all patients, 12-lead ECG is recommended to establish the diagnosis and evaluate ventricular rate, conduction disturbances, ischemia, and other signs of structural heart disease.

Newly diagnosed patients must undergo basic laboratory tests for blood glucose, renal function, lipid profile, electrolytes, thyroid hormones, and blood count with platelet count. In transthoracic ECG, the dimensions of the cardiac chambers, LV and RV function, changes in segmental contractility, the diameter and volume of the LA and right atrium, and storage and valvular diseases should be assessed. Other specific complementary examinations can be used according to the patient's clinical context. The recommended tests for initial assessment of patients with AF are shown in Box 3.

**Box 3:** Recommended tests for initial evaluation of patients with atrial fibrillation

Recommendations	Class of recommendation	Level of evidence
Initial clinical assessment must include detailed clinical history, 12-lead ECG, and laboratory tests (blood glucose, renal function, lipid profile, electrolytes, thyroid hormones, and blood and platelet count)	**I**	**A**
Transthoracic echocardiography should be performed at the first assessment	**I**	**A**

**Box 4:** Recommendations on the investigation of patients with atrial fibrillation

Recommendations	Class of recommendation	Level of evidence
Opportunistic screening with pulse palpation or 12-lead ECG is recommended for patients > 65 years of age	**I**	**B**
When assessing patients with CIEDs, search for accelerated atrial rhythm	**I**	**B**
ECG and 24- to 72-hour Holter monitoring can be used in patients with ischemic stroke or systemic embolism who have risk factors for AF	**I**	**B**
Systematic screening for AF must be individualized according to clinical suspicion and the patient's risk profile	**IIa**	**B**
In patients diagnosed with cryptogenic ischemic stroke, prolonged monitoring with an external or implantable device is required	**IIa**	**B**

AF: atrial fibrillation; ECG: electrocardiogram; CIED: cardiac implantable electronic devices.

### 5.4. Screening Patients at Risk of Atrial Fibrillation

Detecting AF depends primarily on the duration of ECG monitoring. Choosing the appropriate tool for each patient is of great importance. The high medical and hospital costs of AF justify early detection and treatment, especially in populations at higher risk.^122^ AF can be opportunistically or systematically screened according to the age groups (especially ≥ 65 years of age) and in people with the above mentioned risk factors.^123,124^

Opportunistic screening is performed by a health care professional through pulse palpation or ECG during routine care or symptom assessment. However, systematic screening programs involves specific programs that encourage the observation of heart rhythm behavior in a disciplined manner, which can be carried out by patients themselves, with periodic assessments using non-invasive detection devices or specific programs for active AF detection. A cost-effective approach was based on pulse palpation or the use of portable ECG devices. Both opportunistic and systematic screening are more cost-effective than routine clinical assessments for patients over 65 years of age. Community campaigns for pulse palpation, with guidance for patients on how to perform this simple maneuver, are an option for the general population.^125-127^

Apart from periodic pulse palpation, the non-invasive forms of diagnostic screening available in Brazil include intermittent ECG, the Holter system (24 hours to 7 days), and external event monitors (loop recorders), which continuously monitor ECG for ≥ 2 weeks depending on patient tolerance. Studies have shown that frequent 12-lead ECG recordings increased arrhythmia detection fourfold compared to recordings performed during routine annual clinical evaluations. Repeated ECG recordings over a two-week period detected asymptomatic AF in 7.4% of individuals with two or more risk factors for stroke.^125^

Non-invasive heart rate assessment devices are becoming increasingly popular. Portable or wearable devices that detect the pulse rate using plethysmography (eg, pulse oximeters, automatic blood pressure measuring devices, bracelets, and smartwatches) are a simple, accessible way to identify an irregular pulse compatible with AF.^128^ Home ECG monitoring through non-invasive devices (such as smartwatches or electrode interfaces, which record ECG through mobile apps with ≥ 1 leads) provides sufficient data for accurate diagnosis, provided the recordings are validated by a qualified physician. Previous studies with large patient samples have already shown that smartwatch ECG monitoring can accurately detect AF.^13,129^ It should be pointed out that not all of these monitoring devices have been approved by Brazilian Health Regulatory Agency, which is necessary for validation and clinical use. Table 4 summarizes the sensitivity and specificity of the main methods of AF detection. Figure 11 presents a list of the main tools used for AF screening.

**Table 4:** The sensitivity and specificity of atrial fibrillation screening methods (12-lead ECG = gold standard)

Tipo de monitorização	Specificity	Sensitivity
Type of Monitoring	87-97%	70-81%
Pulse palpation	93-100%	86-92%
Blood pressure monitors	98%	76-95%
Electrocardiography	91.5-98.5%	91.4-100%
Smartphone apps	99%	83-94%

Adapted from Hindricks et al.^17^

**Figure 11:** Tools for atrial fibrillation/atrial flutter screening.

### 5.5. Prolonged Electrocardiographic Monitoring

For patients using some type of CIED, these devices should periodically search for the accelerated atrial rhythm that characterizes subclinical AF. The ASSERT trial, which evaluated 2580 patients > 65 years of age with CIED, found a 10.1% incidence of accelerated atrial rhythm lasting > 6 minutes, as well as a higher risk of clinical AF and ischemic stroke in this population.^130^

AF is responsible for about 15% of all ischemic strokes.^130^ A frequent problem in clinical practice is investigating AF in individuals who present with cryptogenic ischemic stroke, especially non-lacunar stroke attributed to an unknown thromboembolic cause, which corresponds to approximately 20% to 40% of ischemic stroke cases.^131^ If AF is detected in these patients, it may be necessary to replace antiplatelet aggregation with oral anticoagulation (OAC). The EMBRACE study found that longer ECG monitoring periods up to 30 days after an ischemic stroke or transient ischemic attack have a higher probability of detecting AF. Additionally, using a loop recorder for 30 days (with automatic AF detection) better detected AF > 30 seconds than Holter monitoring (16.1% vs 3.2%; p < 0.001) in patients who had experienced an ischemic stroke or transient ischemic attack in the last 6 months.^131^ In the CRYSTAL AF trial, 12 months of monitoring with an implantable loop detected AF in 12.4% of patients with cryptogenic ischemic stroke vs 2.0% in those who received conventional monitoring (ECG and Holter), with a RR of 7.3 (p < 0.001).^132^

## 6. Atrial Fibrillation and Thromboembolic Events

### 6.1. Algorithms for Assessing the Risk of Thromboembolic Events

AF is the main cause of thromboembolic events of cardiac origin.^133^ The risk of stroke during AF doubles with each decade of life after 55 years of age, and its incidence is 25% higher in patients > 80 years of age. AF is responsible for almost one-third of all cerebral ischemic events, the main mechanism being cardioembolism.^134^

Local factors, such as reduced LAA flow velocity, dilation, endocardial injury, inflammation, and atrial fibrosis, participate in the pathophysiological mechanism of thrombus formation in AF^135^ and, since it was demonstrated that OAC can reduce the occurrence of thromboembolic events, risk scores have been developed, validated and widely used in decision-making about the introduction of anticoagulant therapy in patients with AF. The most commonly used score is the CHA_2_DS_2_-VA score, previously called CHA2DS2-VAsC (Table 5). The European Society of Cardiology's initiative to change the nomenclature of the score, removing female sex from the scoring system, is based on the fact that sex is a modifiable risk factor, as well as because it excluded a large group of patients with non-binary identities, transgender individuals, or those undergoing hormone therapy. This modification was endorsed for its inclusive nature, and the term CHA_2_DS_2_-VA will be used in this document.

**Table 5:** The CHA_2_DS_2_-VA score

CHA_2_DS_2_-VASc	Score
Congestive heart failure/left ventricular dysfunction	1
Hypertension	1
Age ≥ 75 yrs	2
Diabetes mellitus	1
Stroke/transient ischaemic attack/ Thrombo-embolism	2
Vascular disease (prior myocardial infarction, peripheral artery disease or aortic plaque)	1
Age 65–74 yrs	1

The CHA_2_DS_2_-VA score has high sensitivity and negative predictive value for defining, primarily, the low-risk population, but it has low specificity for detecting high-risk patients.^136-138^

OAC is recommended for patients with CHA_2_DS_2_-VA score ≥ 2. In men scoring 1 and women scoring 2, the risk is considered low (1.3% per year), and anticoagulant therapy should be individualized according to risk profile, bleeding, and patient preferences. Patients scoring 0 do not require anticoagulation. The form and burden of AF have been implicated as additional risk factors for thromboembolic events: the risk is higher in patients with persistent AF and a greater AF burden.^139^

Other scores, such as ABC-AF-stroke and ABC-AF-bleeding (age, biomarkers and clinical history)^140^ and Atria (Anticoagulation and Risk Factors in Atrial Fibrillation)^141^ include biomarkers (N-terminal pro-type B-natriuretic peptide, cardiac troponin-T and growth differentiation factor 15) and renal dysfunction, respectively. The Garfield-AF Risk Tool is based on a machine learning risk prediction model that predicts all-cause mortality, ischemic and hemorrhagic stroke, and systemic embolism in patients with AF.^142^ Although these scores can distinguish between patients with low and high risk of stroke and bleeding, practicality issues and additional cost restrict their use in clinical practice.

### 6.2. Algorithms for Assessing Bleeding Risk

Since several thromboembolic risk factors have been associated with bleeding events, correcting or adjusting modifiable factors is essential for assessing the risks and benefits of subsequent anticoagulant therapy.

A systematic review of 38 studies found that the HAS-BLED score best predicted bleeding risk (Table 6).^143^ Although scores ≥ 3 indicate high risk, they should not be considered a contraindication to anticoagulant therapy. In such cases, better hypertension control, avoiding concomitant use of anti-inflammatory drugs and alcohol, and replacing warfarin with a direct-acting OAC (DOAC) are suggested to mitigate the risk.^134,144^

**Table 6:** Escore HAS-BLED para avaliação de risco de sangramento

HAS-BLED risk	Score
Hypertension (>160 mm Hg)	1
Abnormal renal/liver function CrCl ≤ 50 mL/min or creatinine ≥ 2.26 mg/dL or hemodialysis or renal transplant; bilirubin ≥ 2x normal + GOT or GPT or AF ≥ 3x normal or liver cirrhosis	1 or 2
Stroke	1
Bleeding	1
Labile international normalized ratio	1
Age > 65 years	1
Drug use (NSAIDs, antiplatelet agents) or alcohol consumption (>20 U per week)	1 or 2

GOT: glutamic oxaloacetic transaminase; GPT: glutamic-pyruvic transaminase; NSAIDs: non-steroidal anti-inflammatory drugs. Adapted from Steffel et al.^144^

The ORBIT score, which considers factors such as advanced age, low hemoglobin values, history of bleeding, renal dysfunction, and concomitant antiplatelet drug therapy^145^ did not predict major bleeding events in anticoagulated AF patients better than the HAS-BLED score.^146^

Bleeding risk is dynamic, and periodic reassessment of risk factors is essential. In the mAFA-II trial, prospective dynamic monitoring and reassessment through the HAS-BLED score, along with app-based holistic management, was associated with fewer serious bleeding events and increased anticoagulant use compared with usual care at 12 months of follow-up.^147^

Recommendations on thromboembolic event prevention in patients with non-valvular AF are summarized in Box 5.

**Box 5:** Recommendations on preventing thromboembolic phenomena in non-valvular atrial fibrillation

Recommendations	Class of recommendation	Level of evidence
The CHA_2_DS_2_-VA score should be used in all patients	**I**	**A**
In patients with a CHA_2_DS_2_-VA score ≥ 2, antithrombotic therapy is recommended	**I**	**A**
Antithrombotic therapy is not recommended for low-risk patients (CHA_2_DS_2_-VA = 0)	**I**	**A**
Antithrombotic therapy can be considered in patients with CHA_2_DS_2_-VA = 1 based on the clinical benefits, bleeding risk, AF form, and patient preference	**IIa**	**B**
Oral anticoagulation is recommended for all patients with AF and hypertrophic cardiomyopathy, cardiac amyloidosis, and hyperthyroidism regardless of the CHA2DS2-VA score, for the prevention of embolic events	**I**	**B**
Periodic reevaluation of thromboembolic and bleeding risk is recommended for patients with AF	**I**	**B**
The HAS-BLED score can determine bleeding risk and help identify modifiable factors and high-risk patients	**IIa**	**B**
In patients eligible for antithrombotic therapy, DOACs are preferable to VKAs, except in patients with mechanical heart valves or moderate-to-severe mitral stenosis	**I**	**A**
For patients using VKAs, the INR target should be 2.0-3.0 and should remain in the therapeutic range > 70% of the time	**I**	**B**
Mono- or combined antiplatelet therapy is not recommended for stroke prevention in patients with AF	**III**	**A**
In the absence of absolute contraindications, estimated bleeding risk should not guide decision-making about antithrombotic therapy	**III**	**A**
Oral anticoagulation in patients with subclinical AF may be considered with edoxaban or apixaban, taking into account risk, benefit, and patient preference	**IIa**	**B**

AF: Atrial fibrillation; DOAC: direct-acting oral anticoagulant; INR: international normalized ratio; VKA: vitamin K antagonist.

### 6.3. Preventing Thromboembolic Events

#### 6.3.1. Anticoagulants Available in Clinical Practice

OAC, the first-line therapy for thromboembolism prevention in patients with AF, is guided by estimated stroke risk. Compared with control or placebo, vitamin K antagonist (VKA) therapy reduced stroke risk by 64% and mortality by 26%.^148^ VKA are effective in preventing thromboembolic events, but involve a series of limitations in clinical practice, such as a narrow therapeutic window, variable pharmacokinetics and pharmacodynamics, a wide variety of drug and food interactions, and the need for regular monitoring and adjustment.^134^

Several randomized clinical trials have determined that DOACs are either non-inferior or superior to warfarin for stroke and systemic embolism prevention in patients with non-valvular AF.^149-152^

A meta-analysis of large pivotal studies found that DOACs were associated with a 19% reduction in stroke/systemic embolism risk and a 51% reduction in hemorrhagic stroke risk. DOAC therapy was also associated with a significant 10% reduction in all-cause mortality, a 14% reduction in major bleeding risk, and a 52% reduction in intracranial bleeding, although its gastrointestinal bleeding rate was 25% higher than VKA.^153^

Likewise, COMBINE AF data indicated a lower risk of stroke or systemic embolism in patients treated with DOACs than in those treated with warfarin. Intracranial bleeding risk was reduced by 55% with DOAC therapy, while all-cause mortality and major bleeding rates were reduced by 8% and 14%, respectively. DOACs increased the RR of gastrointestinal bleeding by 31% compared to warfarin.^154^

Substantial data confirm that DOACs are at least as safe and effective as warfarin in clinical practice.^155^ Given that 4 DOACs are available in different strengths for different indications and with different criteria for dose reduction, a personalized approach may be the best strategy for clinical management.

#### 6.3.2. Individualized Anticoagulation Strategies

Although DOACs have greatly contributed to the success of antithrombotic therapy, several factors must be considered when choosing the best anticoagulation strategy. Warfarin remains the first line of treatment for patients with AF and moderate-to-severe mitral stenosis of rheumatic origin and patients with a mechanical cardiac prosthesis. In these patients, the target INR should be around 2 to 3. However, other forms of valvular disease, such as aortic insufficiency or stenosis, mitral insufficiency, bioprostheses, and valve repair, have been included in pivotal studies with DOACs, and a systematic review involving these patients found that these medications are safe and efficacious.^156^

Renal function is a relevant factor in deciding which anticoagulation strategy to use. Although patients with kidney disease easily meet the criteria for anticoagulation, they are also subject, paradoxically, to a high risk of bleeding. Furthermore, these patients are often excluded from large clinical studies, making it even more difficult to select an anticoagulant. The evidence for anticoagulation in stage 4 kidney disease (glomerular filtration rate 15-29 mL/min/1.73 m^2^) is largely based on observational studies. An analysis of patients with low CrCl (CrCl)(25-30 mL/min) from the ARISTOTLE study found that, compared to its safety in patients with CrCl > 30 mL/min, apixaban effectively prevented stroke and was even safer than warfarin. Thus, apixaban has a unique safety profile for patients with more advanced non-dialysis-dependent kidney disease.^157^ End-stage patients (CrCl < 15 mL/min) or those undergoing renal replacement therapy are a challenge in clinical practice. Meta-analyses of observational studies have not found that warfarin is effective for these patients and that it leads to an exponential increase in bleeding risk. Furthermore, controlling INR is a difficult task in this population. Finally, warfarin also leads to pathological calcification in patients with end-stage renal disease. Therefore, the real effectiveness of warfarin in patients with end-stage renal disease is unknown. A retrospective study in this population found no difference thromboembolic event rates between apixaban and warfarin, although patients treated with apixaban had fewer bleeding episodes.^158^ The RENAL-AF trial attempted to assess the safety of apixaban in dialysis patients, but it was interrupted early and became only exploratory, finding similar efficacy and safety to warfarin. Therefore, it is unknown whether anticoagulation benefits patients on dialysis. When these patients are treated with anticoagulation, apixaban seems a reasonable option, although more definitive data are needed. For further information, see the table on special situations in AF.

Frail older adults also have special characteristics that must be considered. A recent study evaluated the safety of switching from warfarin to a DOAC in frail older patients with AF (age > 75 years and Groningen frailty score > 3). The authors found that switching to a DOAC increased the risk of bleeding without reducing the risk of thromboembolic events.^159^ However, relevant methodological issues do not allow us to apply the results in clinical practice, and it would be prudent to wait for new studies in this population. In addition to being a small study and ending early, the outcomes were measured by the researching physician, indicating potential bias. The 69% increase in bleeding with DOACs compared to warfarin is not biologically plausible according to the current literature. Furthermore, 75% of the patients used rivaroxaban, edoxaban, or dabigatran, drugs with higher rates of gastrointestinal bleeding than warfarin, especially in older adults. In summary, stable patients on warfarin can continue using it, but DOACs may be a safe and effective alternative, particularly for frail older patients.

The type and dosage of anticoagulant must be based on the patient's general profile and then reassessed at each clinical evaluation during follow-up, adhering to dose adjustment models from pivotal studies on each medication to minimize the risk of stroke and bleeding.^143^

The pharmacokinetic and pharmacodynamic properties of DOACs and recommended doses are shown in Tables 7 and 8.

**Table 7:** Pharmacokinetic and pharmacodynamic characteristics of direct-acting oral anticoagulants

	Dabigatran	Rivaroxaban	Apixaban	Edoxaban
**Dosage**	2x/day	1x/day	2x/day	1x/day
**Metabolism by CYP450**	Null	32%	15%	< 4%
**Half-life (hours)**	12-17	5-13	12	10-14
**Hours until _Cmax_**	1-3	2-4	3-4	1-2

Cmax: maximum concentration. Adapted from Steffel et al.^144^

**Table 8:** Recommended doses for direct-acting oral anticoagulants

	Dabigatran	Rivaroxaban	Apixaban	Edoxaban
**Standard dose**	150 mg 2x/day	20 mg 1x/day	5 mg 2x/day	60 mg 1x/day
**Adjusted dose**	110 mg 2x/day*	15 mg 1x/day	2.5 mg 2x/day	30 mg 1x/day
**Criteria for dose change**	– Age ≥ 80 years – Concomitant verapamil use or – Increased risk of bleeding	CrCl between 15 and 49 ml/min	At least 2 of the 3 criteria: – Age ≥ 80 years, – Body weight ≤ 60 kg – Creatinine ≥ 1.5 mg/dL	Any of the following criteria: – GFR 15-50 mL/min – Body weight ≤ 60 kg – Concurrent use of dronedarone, cyclosporine, erythromycin, or ketoconazole

Adapted from Hindricks et al.^17^

* The 110 mg dose of dabigatran is not an adjusted dose but a reduced dose tested in the RE-LY trial without adjustment criteria. Since its bleeding rate was lower than warfarin and had similar efficacy, it is recommended for patients at increased risk of bleeding. CrCl: creatinine clearance according to the Cockcroft-Gault formula; GFR: glomerular filtration rate.

### 6.4. Recommendations on Patients Treated with Anticoagulants

Only half the patients with AF and risk factors for stroke are treated with anticoagulants.^17^ Thus, interventions are needed to increase the proportion of adequately treated patients. In the IMPACT-AF trial, a multifaceted and multilevel educational intervention for patients with AF and their care providers that included regular follow-up, the proportion of patients treated with anticoagulation increased significantly (12% in the intervention group vs 3% in the control group, p = 0.0002). Despite the increase in OAC, bleeding rates did not increase, and lower stroke rates were reported in the intervention group. (1% vs 2%, p = 0.043).^160^

Adherence to DOAC therapy is critical, considering that the anticoagulant effect decreases 12–24 hours after the last intake. Although discontinuation rates are significantly lower for DOACs than VKAs due to their better pharmacokinetic profile and favorable safety and efficacy results, discontinuation is still a relevant issue.^161^ Younger age, renal dysfunction, excessive alcohol consumption, lower CHA_2_DS_2_-VA scores and high cost are important causes of treatment discontinuation.^162^ Thus, adherence is extremely important and it must be thoroughly discussed with patients and their families at the beginning of anticoagulant therapy and at each subsequent consultation.

Although DOACs have fewer drug interactions, assessing potential interactions with drugs that use the cytochrome P450 and CYP3A4 pathways is recommended. Furthermore, careful renal function monitoring is required in patients with reduced renal function. The lower the glomerular filtration rate, the more frequent this evaluation should be.

Involving patients in decision-making and discussing anticoagulation options together is key to adequately assessing their needs. Important factors involved in monitoring patients treated with anticoagulation are shown in Table 9.

**Table 9:** Factors involved in monitoring patients treated with anticoagulants

	Frequency	Comments
**Adherence**	Every visit	Medical education. Consider preference for 1 or 2 doses a day
**Thromboembolism**	Every visit	Clinical and imaging assessment when necessary
**Bleeding**	Every visit	
**Adverse events**	Every visit	Carefully evaluate the relationship with the DOAC: change or interrupt the medication.
**Concomitant medications**	Every visit	Drug interactions. Also consider temporary treatment.
**Laboratory**	Use formula*	If CrCl < 60 mg/kg/24 h
	4 months	> 75 years or frailty
	Yearly	If none of the above conditions
**Modifiable factors for bleeding**	Every visit	Uncontrolled hypertension (> 160 mm Hg), aspirin and NSAID therapy, excessive alcohol consumption
**DOAC and patient profile**	Every visit	DOAC prescribed according to patient profile Appropriate dose for clinical condition

CrCl: creatinine clearance; DOAC: direct-acting anticoagulant; NSAID: non-steroidal anti-inflammatory drug.

* For patients with CrCl < 60 mg/kg/24 h, the test request rate must be based on the following formula: rate (in months) = CrCl/10. Adapted from Steffel et al.^144^

#### 6.4.1. Guidelines for Bleeding in Patients Treated with Direct-acting Oral Anticoagulants

Although DOACs have changed the therapeutic approach to non-valvular AF regarding thromboembolic event prevention, related bleeding complications may be a limitation. The incidence of major bleeding in patients treated with DOACs is 2%-3% per year, and the incidence of hemorrhagic stroke is 0.1%-0.5%.^163^ Given their short half-life, minor bleeding can be resolved by discontinuing the medication.

Active bleeding assessment in patients treated with DOACs involves detecting the site of the hemorrhage, determining its severity, and gathering information about the most recent dose of the drug. Factors that influence bleeding risk, such as alcohol use, concomitant aspirin therapy, antithrombotic therapy, and renal dysfunction, should be explored. More specific coagulation tests, such as anti-Xa activity, can be performed, although they are not always available and are often unnecessary to control bleeding. In most cases, appropriate clinical decision-making is based on determining the most recent dose of DOAC and evaluating renal function, hemoglobin, hematocrit, and platelets.

Pharmacokinetic characteristics, which differ among DOACs, can determine treatment changes. Dabigatran, for example, is weakly bound to plasma proteins and may be removable by hemodialysis. Rivaroxaban and apixaban, however, are not dialyzable due to their strong protein binding in plasma.^164^

Minor bleeding events should be treated with supportive measures, such as mechanical compression or minor surgery, to achieve hemostasis. DOACs have a short plasma half-life, and hemostasis can be expected in 12 to 24 h after a missed dose. However, moderate bleeding events may require blood transfusions. If the last dose was taken < 2 to 4 h before bleeding onset, activated charcoal and/or gastric lavage may be considered to reduce further exposure. Of note, activated charcoal is contraindicated for digestive bleeding. It is available in powder form and can be diluted in water or juice if the patient is awake, or administered through a nasogastric tube at a dose of 1 g per kg of body weight. Specific interventions to identify and control the cause of bleeding (eg, digestive endoscopy) should be performed immediately. Dialysis can effectively reduce dabigatran concentration and has been associated with reduced bleeding duration and/or severity.

Severe or life-threatening bleeding requires immediate reversal of the antithrombotic effect. Antidotes may be considered in such cases. Idarucizumab is a monoclonal antibody fragment that binds to dabigatran, with an affinity 350 times greater than that of thrombin, resulting in the neutralization of the anticoagulant effect. The safety of this medication has been demonstrated in several studies with older adults and kidney disease patients. In the RE-VERSE AD study, the agent used in emergency situations safely and quickly neutralized the anticoagulant effects of dabigatran in a group of 503 patients, without pro-coagulant effects. The anticoagulant effect was reversed in 100% of the patients, approximately 1.6 to 2.5 hours after administration. Thromboembolic events occurred in 6.3% to 7.4% of patients.^165^

Andexanet is an inactive recombinant protein that binds to activated factor X inhibitors (rivaroxaban, apixaban, and edoxaban), reversing their anticoagulant effect. Satisfactory reversal has been reported after intravenous administration.^166^ However, DOACs are often not restarted, which might result in high thrombotic event rates.

Finally, ciraparantag, which has not yet been officially approved, is a synthetic drug that directly inhibits direct factor Xa inhibitors and enoxaparin through non-covalent hydrogen bonds, removing these drugs from their target site and reversing their anticoagulant effects.^167^

Fresh frozen plasma can be administered, but as a means of supplementing clotting factors, since its concentrations are much lower than those in prothrombin complex concentrates. It is reasonable to recommend these concentrates in situations of severe bleeding.^168,169^ Although antidotes can be effectively administered in cases of severe bleeding or emergency surgery, their use is relatively rare in clinical practice.

The risk of death after severe DOAC-related bleeding is still significant despite the high rate of effective hemostasis with antidotes. Failure to achieve effective hemostasis strongly correlates with unfavorable outcomes and death. A meta-analysis reported that andexanet has particularly high thromboembolism rates.^170^ Therefore, new studies, especially on implementation strategies and care systematization for patients with severe bleeding, are needed to better understand the role of antidotes in different clinical scenarios.

Figure 12 summarizes bleeding management for patients treated with DOACs. Recommendations on antidote use in cases of bleeding secondary to treatment with DOACs are shown in Table 10, while the main studies on this topic are described in Table 11.

**Figure 12:** Decision-making algorithm for patients with active bleeding secondary to treatment with direct-acting oral anticoagulants.

**Table 10:** Recommendations on antidote use in patients treated with direct-acting anticoagulants

Life-threatening bleeding: central nervous system, uncontrollable bleeding
Bleeding in a critical cavity or organ: intraspinal, intraocular, pericardial, retroperitoneal, or intramuscular with compartment syndrome
Major bleeding despite local hemostasis measures or risk of recurrent bleeding
Emergency surgery or intervention in patients at high risk of bleeding: neurosurgery, lumbar puncture, cardiac, vascular, and liver surgery

**Table 11:** Antidotes for direct-acting oral anticoagulants

Antidote	Target	Description	Estudo principal	Inclusion criteria	Main results
**Idarucizumab**	Dabigatran	Monoclonal antibody fragment	RE-VERSE AD^165^	Uncontrollable bleeding (GI or CNS) Urgent surgery	Bleeding stopped in an average of 2.5 hours Complete reversal of dabigatran's action in 97.5% of patients
**Andexanet**	Direct factor Xa inhibitor	Recombinant molecule derived from human factor Xa	ANNEXA-4^171^	Major bleeding (GI or CNS) 18 h after DOAC	Effective hemostasis within 12 h in 82% of patients. Activity of apixaban and rivaroxaban reduced by 92%
**Ciraparantag** *	Direct factor Xa inhibitor	Synthetic molecule, reverses oral direct factor Xa inhibitors	Ansell et al.^167^ Investigational drug		Total reversal of anticoagulant action in ≤ 30 min

* Not approved for use in clinical practice. CNS: central nervous system; DOAC: direct-acting oral anticoagulant; GI: gastrointestinal tract.

Four-factor prothrombin complex concentrates can be considered as an alternative treatment to reverse the anticoagulant effect of rivaroxaban, apixaban, and edoxaban, despite very limited evidence. Recommendations on active bleeding management in patients treated with DOACs are shown in Box 6.

**Box 6:** Recommendations on active bleeding management in patients treated with direct-acting anticoagulants

Recommendations	Class of recommendation	Level of evidence
Interrupt OAC until bleeding is classified (mild, moderate, or severe) and treated	**I**	**C**
Perform diagnostic interventions to identify the bleeding site in patients without an identified cause	**I**	**C**
Use idarucizumab in patients with severe bleeding and risk of death secondary to dabigatran therapy	**IIa**	**B**
Use andexanet in patients with severe bleeding and risk of death secondary to factor Xa inhibitor anticoagulant therapy	**IIa**	**B**
Use activated charcoal and gastric lavage in patients with moderate or severe bleeding ≤ 4 hours after taking DOACs	**IIa**	**C**
Use prothrombin complex concentrate for moderate bleeding or in patients with severe bleeding unresponsive to the antidote	**IIa**	**C**
Use fresh frozen plasma and platelet concentrate in patients with moderate bleeding when no other therapies are available	**IIa**	**C**
Use activated charcoal for gastrointestinal bleeding	**III**	**C**

DOAC: direct-acting oral anticoagulant; OAC: oral anticoagulant.

### 6.5. Anticoagulant Therapy in Special Situations

#### 6.5.1. Electrical Cardioversion

Patients undergoing electrical cardioversion must be assessed for thromboembolic event risk. Arrhythmia duration (< 24 hours or ≥ 24 hours) has been used to determine the timing of cardioversion. When < 24 hours, it must be preceded by anticoagulation with DOACs (dabigatran, rivaroxaban, apixaban, or edoxaban), subcutaneous heparin (enoxaparin 1 mg/kg), or bolus intravenous heparin (60-70 U/kg).^17^ When onset occurred > 24 hours or unknown, transesophageal echocardiography can be performed to detect thrombus. In the absence of thrombus, cardioversion can be performed after anticoagulation with heparin (intravenous bolus or subcutaneous) or a DOAC.^17^ If transesophageal echocardiography is impossible or when there is a thrombus, a DOAC or warfarin (target INR 2.0-3.0) is needed for 3 weeks before cardioversion.^17^ Of note, the 3 weeks of warfarin should be counted from the first therapeutic level of INR. It is essential to ensure correct DOAC dosage. If treatment adherence is doubtful, transesophageal echocardiography should be considered.^172^

Support for DOAC therapy in cardioversion was initially based on subgroup analyses of large phase 3 studies (RELY, ROCKET-AF, ARISTOTLE, and ENGAGE AF-TIMI).^152,173-175^ Subsequent AF cardioversion studies found that DOACs have similar efficacy and safety to VKA. (Table 12).^176-178^ Some analyses have even found advantages to DOAC therapy.^179^ Recommendations on anticoagulant therapy in electrical cardioversion are shown in Box 7.

**Table 12:** Clinical studies on direct-acting oral anticoagulants in cardioversion of atrial fibrillation

	X-VeRT	ENSURE-AF	EMANATE
**Anticoagulant**	Rivaroxaban	Edoxaban	Apixaban
**Number of patients**	1504	2199	1500
**Study design**	randomized	randomized	randomized
**AF duration**	≥ 48 h	≥ 48 h	< 48 h and ≥ 48 h
**Comparator**	VKA	VKA/low molecular weight heparin	VKA/heparin
**Strategy**	TEE + Rivaroxaban for ≥ 4 hours before ECV	TEE + Edoxaban ≥ 2 hours before ECV	Apixaban ≥ 2 hours before ECV or after 5 doses
**Anticoagulation after ECV**	42 days	28 days	30 days
**Effectiveness outcome**	Composite of stroke, TIA, systemic embolism, heart attack or cardiovascular death	Composite of stroke, TIA, systemic embolism, heart attack or cardiovascular death	Stroke, systemic embolism, mortality
**DOAC & VKA results**	5 (0.5%) vs. 5 (1.0%)	5 (0.5%) vs. 11 (1.0%)	0 (0%) vs. 6 (0.8%)
**Safety outcome**	Major bleeding	Major or clinically relevant bleeding	Major or clinically relevant bleeding
**DOAC & VKA results**	6 (0.6%) vs. 4 (0.8%)	16 (1.5%) vs. 11 (1.0%)	14 (1.9%) vs. 19 (2.5%)

DOAC: direct-acting oral anticoagulant; ECV: electrical cardioversion; TEE: transesophageal echocardiography; TIA: transient ischemic attack; VKA: vitamin K-antagonists. Adapted from Lucà et al.^180^

**Box 7:** Recommendations on anticoagulant therapy in electrical cardioversion

Recommendations	Class of recommendation	Level of evidence
Electrical cardioversion in patients with AF lasting longer than 24 hours should be performed after a minimum of 3 weeks of anticoagulation with VKAs or DOACs and maintained for at least 4 weeks	**I**	**B**
The use of transesophageal echocardiography is recommended as an alternative to the 3 weeks of prior oral anticoagulation	**I**	**B**
The use of anticoagulation is recommended for at least 4 weeks after cardioversion in all patients, regardless of the CHA2DS2-VA score. Indefinite use should be based on the patient's risk profile	**I**	**C**
Emphasize the importance of adhering direct-acting anticoagulant therapy to patients	**I**	**C**
Antiplatelet therapy (alone or in combination) is not indicated for AF cardioversion	**III**	**B**

AF: atrial fibrillation

In addition to AF duration, embolic risk must be considered when scheduling cardioversion. Patients with AF < 12 hours and a low CHA_2_DS_2_-VA score (0 in men and 1 in women) are at low risk. Patients with AF of valvular etiology (severe mitral stenosis or mechanical valve prosthesis) or a previous embolic event are at high risk and should always be treated with the same strategy for > 24 hours.^179^ This approach also applies to patients with AF lasting 12-24 hours (CHA_2_DS_2_-VA ≥ 2 for men and ≥ 3 for women).^179^

OAC should be continued for ≥ 4 weeks after cardioversion in all patients, regardless of AF duration or risk profile.^179^ After this period, OAC maintenance depends on embolic risk: patients with CHA_2_DS_2_-VASc scores ≥ 2 must be anticoagulated indefinitely. In patients with a score of 0, anticoagulation can be discontinued. In patients with intermediate risk (CHA_2_DS_2_-VA = 1), the decision must be individualized. Aspirin is contraindicated in this context. The decision-making algorithm for patients undergoing stable AF cardioversion is illustrated in Figure 13. Recommendations on thromboembolic event prevention in AF cardioversion are shown in Box 7. AF patients presenting with hemodynamic instability should undergo electrical cardioversion and an anticoagulant should be introduced as quickly as possible.

**Figure 13:** Management of patients indicated for cardioversion of stable AF. AF: atrial fibrillation; DOAC: direct-acting oral anticoagulant.

If a thrombus is detected in transesophageal echocardiography, the patient should be anticoagulated for ≥ 3 weeks before cardioversion. Repeating ECG after 3 weeks of anticoagulation is not generally recommended; it is assumed that treatment has been effective and, hence, that new thrombi have not formed.

#### 6.5.2. Valvular Atrial Fibrillation

Valvular heart disease is an independent risk factor for of AF. It is estimated that more than one-third of patients with AF have some form of valvular disease. Patients with valvular disease and AF are at higher risk of embolic events than those without it.^181^

For patients with AF and valvular disease, the anticoagulant should be chosen according to the etiology, the affected valve, and the degree of impairment, in addition to the presence and type of valve prosthesis. Therefore, OAC selection should be individualized in patients with AF and valvular heart disease.

##### 6.5.2.1. Oral Anticoagulation in Patients with Valvular Heart Disease in Native Valves

Although large pivotal studies on anticoagulation have excluded patients with moderate-to-severe mitral stenosis and mechanical prostheses, many patients with valvular heart disease were included. Of an overall sample of 71,531 patients, 13,585 (19%) had some type of valvular heart disease. Mitral regurgitation was the most prevalent type, affecting 10,633 patients (14.9%). Aortic valve pathologies occurred in 3794 patients: aortic regurgitation in 2559 (3.6%) and aortic stenosis in 1235 (1.7%). Tricuspid regurgitation occurred in 4.6% of patients, approximately 1% had previous valve surgery/repair, and 0.5% had mild mitral stenosis.^182-185^

Patients with valvular disease were older, had more comorbidities, and higher rates of stroke, systemic embolism, and major bleeding. Nevertheless, the efficacy and safety of DOACs were comparable to warfarin, with generally no differences between the groups. The only exception was a greater bleeding risk in patients treated with rivaroxaban.^182,185-187^ Thus, DOACs are the drug of choice in patients with AF and valvular diseases, including mitral regurgitation, aortic regurgitation, and aortic stenosis (especially of degenerative etiology).

##### 6.5.2.2. Oral Anticoagulation in Patients with Moderate-to-Severe Mitral Stenosis

INVICTUS was the first randomized trial to compare the efficacy and safety of rivaroxaban vs VKA. Its sample included in 4531 patients with AF and moderate-to-severe mitral stenosis of rheumatic etiology. Primary outcomes (a composite of stroke, systemic embolism, myocardial infarction, death from vascular or unknown causes) occurred more commonly in the rivaroxaban group (8.21%/year vs 6.5%/year). When analyzed in isolation, the rivaroxaban group had higher rates of death and ischemic stroke. Regarding bleeding rates, no differences were observed between the groups.^188^ The ongoing DAVID-MS trial is investigating the effects of dabigatran vs warfarin in patients with moderate-to-severe mitral stenosis.^189^ Based on current evidence, patients with AF and moderate-to-severe mitral stenosis of rheumatic etiology who are eligible for anticoagulation should be treated with VKAs.

##### 6.5.2.3. Oral Anticoagulation in Patients with Valvular Prostheses

RIVER, a randomized Brazilian trial assessing the efficacy and safety of rivaroxaban, included 1005 patients with AF and a bioprosthetic mitral valve. Rivaroxaban was non-inferior to warfarin for the primary endpoint of death, major cardiovascular events, or major bleeding. The rivaroxaban group also had lower rates of cardiovascular death, major bleeding, and stroke. Furthermore, patients included in the rivaroxaban group in the first 3 months after valve replacement (N = 189) had a significant reduction in the trial's primary outcome (6.4% vs 18.9%).^190^ Hence, DOACs are a safe and effective alternative for anticoagulation in patients with AF who are undergoing mitral valve replacement with a bioprosthesis.

In patients undergoing transcatheter aortic valve implantation (TAVI), observational studies and initial reports disagreed regarding DOACs vs warfarin.^191,192^ Two randomized clinical trials on this topic have recently been published. The ATLANTIS trial included 1451 patients undergoing TAVI, who were divided into groups with and without AF. In the AF group, the primary outcome (a composite of death, stroke, ischemic attack, systemic embolism, and valve thrombosis) was similar between the apixaban and warfarin groups. The results were similar for bleeding rates. In the non-AF group, which compared apixaban to standard-of-care therapy (92% received antiplatelet therapy alone), apixaban increased the risk of the primary outcome, although it reduced the risk of clinical or subclinical valve thrombosis.^193^

The ENVISAGE-TAVI AF trial compared edoxaban with VKA in 1426 patients with AF undergoing TAVI. The groups had similar results for a composite outcome of death, stroke, systemic embolism, acute myocardial infarction, valve thrombosis, and major bleeding. However, higher rates of gastrointestinal bleeding occurred in patients who used edoxaban 60 mg and in those who concomitantly used antiplatelet agents.^194^

The POPular TAVI trial assessed the safety of associating anticoagulant and antiplatelet agents in patients with AF who were undergoing TAVI. This trial included 313 patients (more than 94% had AF and approximately 30% used DOACs). Anticoagulation alone resulted in fewer bleeding events, without a concomitant increase in ischemic events, suggesting that anticoagulant therapy alone may be sufficient for these patients.^195^

Based on these data, DOACs can be considered a safe and effective option for patients with AF undergoing TAVI who are eligible for anticoagulant treatment. The decision to associate a DOAC with an antiplatelet agent must be individualized and, in general, reserved for those who have recently (< 3 months) undergone percutaneous coronary intervention.

The RE-ALIGN study investigated anticoagulation with DOACs in patients with AF and mechanical valve prostheses, comparing dabigatran to warfarin. The study planned to include 405 patients with mechanical valve prostheses in the aortic or mitral positions. However, after including 252 patients, it was interrupted early due to the higher rates of stroke and major bleeding in the dabigatran group.^196^ Likewise, the PROACT-Xa study planned to include 1000 patients undergoing mechanical prosthesis implantation in the aortic position (On-X valve), comparing apixaban to warfarin regarding a primary composite outcome of valve thrombosis and thromboembolic events. After including 863 participants, the study was interrupted due to the higher occurrence of thromboembolic events in the apixaban group. There was no significant difference in bleeding rates between the groups.^197^ Therefore, patients with mechanical prostheses must be anticoagulated with warfarin. Figure 14 shows a flowchart of OAC in patients with valvular heart disease and AF.

**Figure 14:** Flowchart for oral anticoagulation in patients with valvular heart disease and AF. DOAC: Direct-acting oral anticoagulant; TAVI: Transcatheter aortic valve implantation.

Recommendations on anticoagulation in patients with valvular heart disease and AF who are eligible for anticoagulation are shown in Box 8.

**Box 8:** Recommendations on anticoagulation in patients with valvular heart disease and atrial fibrillation who are eligible for anticoagulation

Recommendations	Class of recommendation	Level of evidence
In patients with mechanical valve prostheses, warfarin is the drug of choice.	**I**	**A**
In patients with aortic stenosis, aortic regurgitation, and mitral regurgitation, DOACs are preferable to warfarin.	**I**	**A**
In patients with moderate and severe mitral stenosis, warfarin is the drug of choice.	**I**	**B**
DOACs represent a safe and effective alternative for anticoagulation in patients with AF who have undergone mitral valve replacement with a bioprosthesis.	**IIa**	**B**
DOACs represent a safe and effective alternative for anticoagulation in patients with AF who have undergone aortic bioprosthesis implantation (TAVI).	**IIa**	**B**
DOACs are contraindicated in patients with moderate-to-severe mitral stenosis.	**III**	**A**
DOACs are contraindicated in patients with mechanical valve prostheses.	**III**	**A**

DOAC: Direct-acting oral anticoagulant; TAVI: Transcatheter aortic valve implantation.

#### 6.5.3. Anticoagulation in Acute Coronary Syndrome

In patients with acute coronary syndrome (ACS) and AF who are treated with OAC, adequate consideration of bleeding risk and intervention time is required. OAC is a relative contraindication to thrombolysis, and, preferentially, these patients should be screened for primary angioplasty.^198^ Radial access should be prioritized, given the lower risk of bleeding, and more potent P2Y12 inhibitors, such as prasugrel and ticagrelor, should be avoided.^198,199^

Patients with ST-segment elevation ACS and non-ST-segment elevation ACS, as well as high-risk non-ST-segment elevation ACS, should receive an additional dose of parenteral anticoagulant (eg, unfractionated heparin 60 IU/kg or enoxaparin 0.5 mg/kg), regardless of the most recent dose of OAC.^200^ In non-ST-segment elevation ACS without criteria for emergency percutaneous coronary intervention, OAC should be interrupted until the INR is < 2 for warfarin and for 24 hours for DOACs. If the glomerular filtration rate is < 30 ml/min, the DOAC should be interrupted until the glomerular filtration rate reaches > 30 ml/min for 36 h. OAC should then be associated with parenteral anticoagulation until the time of the procedure.^201^ Recommendations on anticoagulant therapy during the acute phase of coronary syndrome are shown in Box 9.

**Box 9:** Recommendations on anticoagulant therapy during acute coronary syndrome

Recommendations	Class of recommendation	Level of evidence
Preferentially, anticoagulated patients should be referred for primary angioplasty	**IIa**	**C**
Patients with ST-segment elevation ACS and oral anticoagulation should receive the usual dose of parenteral anticoagulant	**IIa**	**C**
In patients with non-ST-segment elevation ACS and no high-risk criteria, oral anticoagulants should and transitioned to parenteral anticoagulants	**IIa**	**C**

ACS: acute coronary syndrome

Clinical studies have also investigated anticoagulant therapy after percutaneous coronary intervention, since AF combined with chronic coronary artery disease (CAD) is not an uncommon scenario. It is estimated that around 10%-15% of patients with AF undergo angioplasty at some point in their lives. Moreover, it is known that anticoagulant therapy alone is insufficient to prevent stent thrombosis, just as the use of antiplatelet agents alone does not prevent thrombotic events in AF. Therefore, combining these 2 classes of medications poses a challenge: protecting against cardiovascular events while preventing excessive increase in bleeding risk.^202^ Five randomized clinical trials (4 using DOACs and 1 using warfarin) evaluated triple therapy with aspirin, a P2Y12 inhibitor (generally clopidogrel), and an anticoagulant vs double therapy with a P2Y12 inhibitor (generally clopidogrel) and an anticoagulant in patients with AF with recent ACS or after elective angioplasty (Table 13).^203-207^

**Table 13:** Summary of the main clinical studies on antithrombotic therapy with direct-acting oral anticoagulants in patients with atrial fibrillation and coronary artery disease

Study	Year of publication	N	Treatment	Primary safety outcome	Time until de-escalation (days)	Follow-up (months)	Conclusions
**PIONEER AF-PCI**	2016	1389	Dual therapy (clopidogrel + rivaroxaban) vs 8 months triple therapy (aspirin + clopidogrel + warfarin)	Major or minor TIMI bleeding or bleeding requiring medical attention	3	12	Lower rates of clinically relevant bleeding with dual therapy
**RE-DUAL PCI**	2017	2725	Dual therapy (clopidogrel + dabigatran 110 mg or 150 mg 2x/d) vs 2.7 months triple therapy (aspirin + clopidogrel + warfarin)	ISTH major bleeding or non-major clinically relevant bleeding	5	14	Lower bleeding rates with dual therapy. Dual therapy was noninferior to triple therapy regarding thrombotic events
**AUGUSTUS**	2019	4614	Dual therapy (clopidogrel + apixaban 5 mg twice a day/warfarin) vs 6 months triple therapy (aspirin + clopidogrel + apixaban/warfarin)*	ISTH major bleeding or non-major clinically relevant bleeding	6	6	Dual therapy with clopidogrel and apixaban without aspirin resulted in less bleeding and no increase in ischemic event rate than therapy with warfarin, aspirin, or both.
**ENTRUST-AF PCI**	2019	1506	Dual therapy (clopidogrel + edoxaban 60 mg/d) vs 3 months triple therapy (aspirin + clopidogrel + warfarin)**	ISTH major bleeding or non-major clinically relevant bleeding	2	12	Dual therapy was noninferior to triple therapy with warfarin, with no significant increase in ischemic events

* Apixaban 2.5 mg twice daily if ≥ 2 of the following criteria are met: age > 80 years, weight < 60 kg, or creatinine > 1.5 mg/dl.

** Edoxaban 30 mg/d if any of the following criteria are met: CrCl 15-50 ml/min, weight < 60 kg, or concomitant P-glycoprotein inhibitor therapy. ISTH: International Society on Thrombosis and Haemostasis; TIMI: Thrombolysis in Myocardial Infarction Study Group.

Despite some degree of heterogeneity, these studies generally found lower bleeding rates in dual therapy with no increase in ischemic events. However, these studies had insufficient power to evaluate ischemic outcomes. Subsequent meta-analyses and sub-analyses found lower bleeding in dual therapy, despite a trend towards an increased risk of ischemic events, especially in the first 30 days.^208-211^

In the AUGUSTUS trial, dual therapy with DOACs (without aspirin) had a better safety profile than triple therapy (aspirin + clopidogrel + warfarin), including a reduction of up to 78% in relevant bleeding rates. For 6 months of dual strategy (apixaban/clopidogrel), the number needed to treat to prevent significant bleeding was 9. Apixaban also reduced the risk of death and hospitalization.^209^ Finally, the ENTRUST trial found that dual therapy with edoxaban (60 mg 1x/day) + a P2Y12 inhibitor was non-inferior to classic triple therapy (VKA + dual antiplatelet therapy) for a composite outcome of major or clinically relevant bleeding at 12 months, and thrombotic outcomes did not differ between groups.^207^

Triple therapy including clopidogrel as a P2Y12 inhibitor and, preferably, DOACs is recommended in the first 7 days, followed by dual antiplatelet therapy with clopidogrel for 1 year in patients with previous ACS, and for 6 months after elective angioplasty. After this period, the anticoagulant must be continued alone.

On an individual basis, apixaban/aspirin/clopidogrel triple therapy can be extended for up to 1 month in patients at high risk of stent thrombosis (eg, trunk or bifurcation lesions, stent mesh > 60 mm, stent hypoexpansion, intervention for chronic occlusion, previous stent thrombosis with adequate antiplatelet treatment, angioplasty in acute coronary syndrome, decompensated diabetes, or chronic kidney disease). Such decisions must always be accompanied by bleeding assessment.

Box 10 summarizes the recommendations on anticoagulant and antiplatelet agent therapy in patients with CAD. Figure 15 outlines antithrombotic therapy in different CAD scenarios.

**Box 10:** Recommendations on anticoagulant and antiplatelet agent therapy in patients with coronary artery disease after percutaneous coronary intervention

Recommendations	Class of recommendation	Level of evidence
In patients undergoing coronary angioplasty who require anticoagulation due to AF, use triple therapy for 1 week, followed by double therapy.	**I**	**A**
In patients hospitalized for ACS as part of a clinical treatment strategy who require anticoagulation due to AF, use triple therapy for 1 week, followed by double therapy	**I**	**A**
Dual therapy with antiplatelet agents and OAC should be maintained for 6 months in patients after elective angioplasty and 1 year after ACS	**I**	**A**
Triple therapy can be extended for up to 1 month in individual cases	**IIa**	**C**
Priority should be given to clopidogrel as an antiplatelet agent in combination with OAC	**IIa**	**A**
DOACs are superior to warfarin when associated with antiplatelet agents after coronary intervention	**I**	**A**
The management of modifiable bleeding risk factors should be performed.	**I**	**B**

ACS: acute coronary syndrome; AF: atrial fibrillation; DOAC: direct-acting oral anticoagulant; OAC: oral anticoagulation.

**Figure 15:** The duration of double and triple antithrombotic therapy for patients with coronary artery disease and atrial fibrillation in different clinical scenarios: (A) patients with acute coronary syndrome or high thrombotic risk; (B) patients undergoing elective percutaneous intervention.

Unless contraindicated, proton pump inhibitors are recommended for all patients undergoing dual antithrombotic therapy.^212,213^ Likewise, physicians must be proactive regarding modifiable bleeding risk factors, including adequate blood pressure control, reducing alcohol consumption, and optimizing renal function.^214^ Recommendations on anticoagulation in association with antiplatelet agents are shown in Box 10.

#### 6.5.4. Perioperative Anticoagulation Management in Cardiac Ablation and Surgery

Although there is some variation in anticoagulant therapy management for patients undergoing percutaneous AF ablation, the procedure is generally performed without interrupting OAC, including warfarin (provided the INR is within the therapeutic range) and DOACs. In patients who are not already on anticoagulation therapy, OAC is recommended for 3 to 4 weeks prior to the procedure.^215^ A meta-analysis of 12 studies found that uninterrupted anticoagulation with DOACs for catheter ablation of AF was associated with lower rates of stroke and transient ischemic attack than VKA (0.08% vs 0.16%, respectively) and both had similar rates of systemic embolization (8.0% vs 9.6%, respectively). Major bleeding rates were significantly lower with DOAC than VKA (0.9% vs 2.0%, respectively).^216^

In general, maintaining DOAC therapy entails a lower incidence of stroke and transient ischemic attack, as well as a significant reduction in major bleeding in patients undergoing catheter ablation of AF. In RE-CIRCUIT, the largest periprocedural randomized trial to compare a DOAC with VKA, the incidence of major bleeding in the first 8 weeks after ablation was significantly lower with dabigatran (1.6% vs 6.9%, respectively).^217^ Other randomized trials, such as VENTURE-AF (rivaroxaban),^218^ AXAFA-AFNET 5 (apixaban)^219^ and ELIMINATE-AF (edoxaban)^220^ also found similar event rates for DOACs and VKAs.

However, heparin bridging increases the incidence of bleeding and should be avoided. In clinical practice, the term uninterrupted anticoagulation usually refers to regimens in which 1 or 2 doses of DOAC may be omitted prior to ablation. However, DOAC administration is truly uninterrupted in randomized trials, and recommendations to omit doses prior to the procedure are unfounded.

After the ablation procedure, the first dose should be administered in the afternoon or the following morning, according to the previous anticoagulation regimen.^218^

For patients treated with OAC, the bleeding risk in elective cardiac surgeries is considered high. DOAC therapy must be interrupted for ≥ 48 hours before the procedure, and 72- to 96-hour interruptions can be considered in patients at risk of drug accumulation, such as older adults and patients with chronic kidney disease.^221^ VKA therapy should be interrupted approximately 5 days before the procedure, and it is considered safe to perform cardiac surgery at INR < 1.5.^222^ As in most situations, heparin bridging is not routinely recommended for anticoagulated patients undergoing elective surgery.^221^ Table 14 summarizes the recommendations on anticoagulant interruption according to renal function.

**Table 14:** Direct-acting oral anticoagulant discontinuation prior to cardiac surgery according to renal function

CrCl value	Dabigatran	Rivaroxaban Apixaban Edoxaban
CrCl > 80 mL/min	≥ 48 h	≥ 48 h
CrCl 50-79 mL/min	≥ 72 h	≥ 48 h
CrCl 30-49 mL/min	≥ 96 h	≥ 48 h
CrCl 15-29 mL/min		≥ 48 h

CrCl: creatinine clearance.

After cardiac surgery, restarting anticoagulation depends on factors such as bleeding and additional interventions. A prophylactic dose of heparin is recommended in the early postoperative period (approximately 12-24 hours after the procedure) if there is adequate hemostasis, followed by full anticoagulation after approximately 36 hours, if there is no active bleeding.^222^ Once adequate hemostasis has been achieved, the drains have been removed, and the patient is clinically stable with no further invasive interventions planned, transitioning from heparin to full OAC is recommended. Recommendations on antithrombotic therapy in ablation procedures and cardiac surgery are presented in Box 11.

**Box 11:** Recommendations on antithrombotic therapy in ablation procedures and cardiac surgery

Recommendations	Class of recommendation	Level of evidence
In patients with AF and thromboembolic risk factors who are not treated with pre-ablation OAC, anticoagulation should be initiated 3 weeks before the procedure	**I**	**C**
Alternatively, transesophageal echocardiography or cardiac computed tomography angiography can be performed to exclude intracavitary thrombus	**IIa**	**C**
For patients who will undergo AF catheter ablation who are treated with VKAs or DOACs, OAC should not be interrupted before the procedure	**I**	**A**
After catheter ablation, continuous OAC with VKAs or DOACs is recommended for ≥ 2 months and indefinitely for high-risk patients	**I**	**C**
DOAC therapy must be interrupted ≥ 48 hours before cardiac surgery	**I**	**C**
In patients at high risk of bleeding, such as older adults and patients with kidney disease, anticoagulant therapy can be interrupted 72 hours before heart surgery	**IIa**	**C**

AF: atrial fibrillation; DOAC: direct-acting oral anticoagulant; OAC: oral anticoagulation; VKA: vitamin K-antagonist.

#### 6.5.5. Anticoagulation in Subclinical Atrial Fibrillation

Subclinical AF is characterized by short asymptomatic episodes, generally lasting < 24 hours, typically detected by implantable cardiac devices.^223^ This form of AF occurs in up to one-third of patients with implantable devices, such as pacemakers and implantable cardioverter defibrillators. The risk of stroke in this population appears to be lower than in patients with clinical AF.^223-225^ Thus, the benefits of OAC in this population have not yet been fully determined. Recently, the NOAH-AFNET 6 trial randomized 2536 patients with rapid atrial rhythm episodes detected by implantable devices and an increased risk of stroke to either edoxaban or placebo (56% received aspirin or placebo).^224^ Edoxaban was not superior to placebo for the composite efficacy outcome of stroke, systemic embolism, or cardiovascular death, which was associated with an increased risk of major bleeding in the edoxaban group. Because this trial was interrupted early, it did not have adequate statistical power to detect protective effects from OAC, especially regarding stroke, the most feared outcome of AF.

The ARTESiA trial, which randomized more than 4000 patients with subclinical AF and a high stroke risk to apixaban or aspirin, found a 37% lower RR for stroke and systemic embolism in the apixaban group.^226^ Importantly, the risk of fatal or more severe strokes according to the modified Rankin scale was 49% lower in the apixaban group. Many strokes in the aspirin group (45%) were severe or fatal. As expected, major bleeding increased in the apixaban group, but most episodes (85%) were treated clinically, without requiring more aggressive control measures. Of note, there was no difference between apixaban and aspirin regarding fatal intracranial bleeding or bleeding that led to hemodynamic instability.

The results of the NOAH-AFNET6 and ARTESiA trials may seem to differ, but they do not. Although the statistical power of the NOAH-AFNET6 trial was insufficient to demonstrate that OAC benefitted subclinical AF patients, the trend was similar to the results of the ARTESiA trial, including a 32% reduction in the RR of stroke.^227^ Thus, in patients with subclinical AF, the overall data indicate that OAC with apixaban or edoxaban can be considered, although therapeutic decision-making must be shared, as well as individualized based on several factors (Figure 16). It should be noted that around a quarter of patients progress to subclinical AF > 24 hours or even to clinical AF.^130^ Thus, careful monitoring is recommended when anticoagulation has not been started.

**Figure 16:** Overlap between atrial fibrillation phenotypes. Shared decision-making must consider the duration, symptoms, thromboembolic risk, bleeding risk, arrhythmia identification methods, and evidence that anticoagulation will benefit the patient. Patient preferences must be considered for better antithrombotic therapy results. AF: atrial fibrillation; ECG: electrocardiogram; CIED: cardiac implantable electronic device.

### 6.6. Left Atrial Appendage Occlusion and Anticoagulation

#### 6.6.1. Contraindications for Anticoagulant Therapy

The few absolute contraindications to OAC include severe acute bleeding, associated comorbidities (eg, severe thrombocytopenia [< 50,000/µL] and severe anemia [under investigation]), and recent severe bleeding (eg, intracranial hemorrhage). In these situations, no anticoagulant therapy should be considered. However, as soon as these conditions are resolved, anticoagulant treatment should be considered.^228^

#### 6.6.2. Percutaneous Left Atrial Appendage Occlusion

It is estimated that more than 90% of thrombi in patients with non-valvular AF originate in the LAA.^134,229^ Percutaneous LAA occlusion is a minimally invasive procedure to close the LAA using dedicated devices. The efficacy and safety of LAA occlusion have been demonstrated in several non-randomized trials and 2 randomized clinical trials (PREVAIL and PROTECT AF).^230-232^

Another real-world study of 106 patients with AF found that percutaneous LAA occlusion is safe and effective. The rate of thromboembolic events was 3.3%/year, including fewer expected events based on CHADS_2_ (66% relative reduction) and CHA_2_DS_2_-VA (59% reduction) scores.^233^ Similar results were obtained in another non-randomized, multicenter study of 150 AF patients without valvular heart disease who had a high risk of stroke and were contraindicated for OAC therapy. In these patients, the stroke rate was 1.7%/year, which was 77% lower than the number predicted by CHADS_2_ score.^234^ In a Taiwanese retrospective study of patients undergoing implantation with a Watchman device or an Amplatzer cardiac plug/Amulet, the procedure was found to be safe and technically viable, with satisfactory long-term clinical results in a mean follow-up of 28 (SD, 14) months.^235^

In a meta-analysis of 5-year follow-up data from the PREVAIL and PROTECT AF randomized trials, in which patients underwent percutaneous LAA occlusion with the Watchman device, the rates of stroke, systemic embolism, or cardiovascular/unexplained death were similar to warfarin therapy. However, patients undergoing LAA occlusion experienced fewer bleeding events, disability, or death compared to warfarin.^236^

Systematic reviews have also investigated LAA occlusion in patients with AF. Al-Abcha et al. concluded that LAA occlusion devices (Watchman, Watchman-FLX, and Amplatzer Amulet) resulted in lower rates of stroke, systemic embolism, and cardiovascular death than drug treatment (warfarin, DOAC, or VKA). Additionally, all-cause mortality, cardiovascular mortality, hemorrhagic stroke, major bleeding, and non-procedure-related major bleeding were significantly lower with device implantation. However, the risk of any type of stroke, ischemic stroke, or systemic embolism was similar between the groups.^237^ Therefore, percutaneous LAA occlusion may prevent thromboembolism in patients with non-valvular AF, but it is contraindicated for patients treated with OAC or in whom OAC therapy failed. Randomized trials and adequately designed studies directly comparing LAA occlusion with DOAC therapy could provide relevant data for clinical practice on the role LAA occlusion devices in patients with AF.

#### 6.6.3. Surgical Occlusion of the Left Atrial Appendage

For patients with non-valvular AF and a high risk of thromboembolism in whom long-term OAC therapy is contraindicated, LAA occlusion is performed to reduce the risk of cardioembolic events and bleeding.^238-241^ In the randomized LAAOS III study, patients who underwent surgical LAA occlusion and OAC had lower rates of stroke or systemic embolism than those treated with OAC alone.^242^

Current evidence indicates that concomitant LAA occlusion should be performed in patients with a history of AF who undergo cardiac surgery for another cause.^242^ In a meta-analysis, Prasad et al. found significantly lower postoperative stroke rates in patients who underwent LAA occlusion, both in the short term (30 days after the procedure) and the long term (> 2 years).^241^ Surgery can also be considered in patients with repetitive thrombus episodes who are not indicated for percutaneous intervention.^243^

Since the first description of surgical intervention for LAA occlusion in 1949, a number of approaches have been proposed, including LAA amputation, occlusion by surgical stapler (Endo GIA II, EZ45), suture, or patch, in addition occlusion with devices such as AtriClip.^239,243,244^ The approach should be tailored to the clinical and surgical characteristics of each patient. (Table 15).

**Table 15:** Surgical approaches according to patient profile

Surgical approach	Clinical and surgical characteristics
Stapler occlusion or amputation	Cardiac surgeries not involving incision of the left atrium (coronary revascularization with CPB, aortic valve surgery)
Suture ligation or occlusion	Concomitant cardiac surgery involving incision of the left atrium (mitral valve surgery, Cox-maze) Fragile tissue in the in the left atrial appendage Reoperation High risk of bleeding Patients for whom percutaneous treatment is contraindicated or has failed.
Device occlusion	Cardiac surgeries not involving incision of the left atrium (coronary revascularization with CPB, aortic valve surgery) High bleeding risk Reoperation Patients for whom percutaneous treatment is contraindicated or has failed.

CPB: cardiopulmonary bypass.

Although studies comparing different surgical techniques are scarce,^242,245^ some authors have suggested that LAA amputation is superior to other techniques not involving devices, arguing that the sutures or staples will gradually corrode the walls of the intact LAA, causing it to reopen.^246^ Incomplete LAA occlusions increase the risk of stroke or systemic embolism by 5 times.^247^

Clipping devices, such as AtriClip, have shown promising results. A randomized trial demonstrated the safety and effectiveness of this method, with success rates > 95%.^248^ For LAA occlusion, epicardial clips can be used in procedures involving median sternotomy, in minimally invasive procedures,^249^ or in robot-assisted procedures.^250^ Recently, Franciulli et al. assessed the effects of LAA occlusion with the AtriClip via thoracoscopy in patients at high risk of bleeding, finding procedural success in 100% of cases, with no adverse cerebral events or short-term mortality (in 6 months).^251^ Similar results were obtained by Branzoli et al.^252^ Thus, intervention is an option in patients contraindicated for anticoagulant or dual antiplatelet therapy.

A new technique was proposed by Ghinescu et al. for right chamber robot-assisted cardiac surgery: the LAA is invaginated into the left atrium, excised completely at the base with scissors, and the stump is then closed from the inside with a loop suture. Although this procedure has only been performed in 20 patients, every attempt was successful, resulting in complete LAA resection without a residual stump.^253^ Despite these excellent initial results, the gold standard for LAA occlusion is still uncertain. Randomized studies comparing surgical techniques with anticoagulant therapy are needed. Recommendations on percutaneous and surgical occlusion of the LAA are shown in Box 12.

**Box 12:** Recommendations on percutaneous and surgical occlusion of the left atrial appendage in patients with atrial fibrillation

Recommendations	Class of recommendation	Level of evidence
The indication for closure or surgical exclusion of the left atrial appendage should be considered in patients with AF undergoing cardiac surgery	**I**	**B**
The indication for percutaneous closure or exclusion of the left atrial appendage should be considered in patients with AF at high thromboembolic risk who have an absolute contraindication to anticoagulant therapy or in whom anticoagulant therapy has failed	**IIa**	**B**
The discontinuation of anticoagulation in patients undergoing surgical exclusion of the left atrial appendage has uncertain benefits	**IIb**	**A**

#### 6.6.4. Anticoagulation after Left Atrial Appendage Occlusion

##### 6.6.4.1. Anticoagulation after Percutaneous Left Atrial Appendage Occlusion

Prostheses become sealed as the endothelium grows over their surface. Partial or complete endothelialization usually occurs 30 to 90 days after percutaneous occlusion of the LAA.^254^ During this period, antithrombotics are recommended to prevent device thrombosis.

A meta-analysis of more than 10,000 patients found a device thrombosis rate of 3.8%. The rate of cerebral ischemic events was significantly higher in patients with device thrombosis than those without it.^255^ Factors such as hypercoagulable state, pericardial effusion, renal dysfunction, implant depth > 10 mm, and non-paroxysmal AF were associated with a higher rate of device thrombosis.^256^

The pivotal PROTECT-AF and PREVAIL trials used protocols designed to minimize the risk of procedure-related thrombosis and thromboembolism, including imaging, clinical follow-up, and antithrombotic therapy. However, such protocols have not been evaluated in a randomized manner, and real-world studies show that only about 12% of patients receive the U.S. Food and Drug Administration-approved regimen in clinical practice. VKA or DOAC monotherapy appears to be beneficial, resulting in extremely low rates of device thrombosis and thromboembolic phenomena. Ongoing randomized trials, such as ADALA, ANDES, APPROACH, DEA-LAA and FADE-DRT, will assess the effects of DOACs after percutaneous LAA occlusion.

The use of antiplatelet agents in this scenario is also empirical, and there are high rates of discontinuation during clinical follow-up. Currently, the ASPIRIN-LAAO study is evaluating the effects of discontinuing aspirin therapy 6 months after the implant.

Peri-device leakage is common and its incidence varies. A large American study of > 50,000 patients found small leaks (1-5 mm) in about 25% of patients, while leaks > 5 mm were rare (< 1%).^257^ Leaks > 5 mm represent incomplete occlusion and require continued OAC or surgical closure.

Devices with better sealing properties, deflectable sheaths that allow coaxial alignment with the LAA, and imaging-based device selection are important factors in minimizing the risk of device-related thrombosis. Occluder devices without antithrombotic therapy may be considered in patients with a very high bleeding risk.

The ongoing OPTION, CHAMPION-AF, and CATALYST trials will investigate the effects of Watchman FLX and Amulet prostheses in patients eligible for OAC vs those treated with DOACs alone. Thus, until the results of these randomized clinical trials are available, antithrombotic therapy must remain empirical for these patients.

##### 6.6.4.2. Antithrombotic Therapy After Surgical Occlusion of the Left Atrial Appendage

The LAAOS III trial compared surgical LAA occlusion vs non-occlusion in patients with AF and a CHA_2_DS_2_-VA score ≥ 2 who were undergoing cardiac surgery. This randomized trial, which included almost 5000 patients, found a 33% reduction in stroke rates in the occlusion group and no increase in adverse events. Thus, LAA occlusion suggests additional effects of anticoagulation.^242^ Since it has not been determined whether it is safe to discontinue OAC in these cases, long-term OAC therapy should be maintained based on individual thromboembolic risk (CHA_2_DS_2_-VA score), even in cases of successful surgical occlusion.

### 6.7. Strategies to Minimize Bleeding Risk

Around 1.9 million people die worldwide each year due to hemorrhaging, and in 1.5 million of these it is due to physical trauma. Anticoagulant therapy is one of the main risk factors for bleeding, and the majority of these patients are treated in emergency services in the United States. These patients have higher morbidity and mortality than those who do not use these medications.^258^ Thus, before beginning OAC therapy, the patient's clinical condition, as well as the anticoagulant's mechanism of action and the pathophysiology of hemorrhaging, must be assessed to minimize risk and resolve any events arising from treatment.

DOACs are superior to warfarin in terms of intracranial hemorrhage risk, and their use has grown progressively each year. However, they still involve risk, and the possibility of trauma and other morbid conditions that increase the incidence of bleeding must be considered, especially in older patients and individuals with kidney disease and dehydration.^17^

Bleeding risk assessment should be followed by risk/benefit assessment. HAS-BLED is the best predictor of bleeding risk,^17^ although many parameters of CHA_2_DS_2_-VA and HAS-BLED scores overlap. High bleeding scores should not be considered a contraindication for anticoagulant therapy.^134^

When deciding to begin treatment, a history of falls, especially in older patients, is usually viewed with caution. However, it is not an independent predictor of bleeding in patients treated with OAC. Furthermore, compared to warfarin, DOACs have a protective effect against falls.^259^ Data indicate that 295 falls per year would be necessary for serious bleeding risk to outweigh the lower ischemic stroke risk in OAC therapy.

The most efficient strategy to reduce bleeding risk in patients treated with OAC is correct assessment of risk factors, which can be classified as non-modifiable, potentially modifiable, or modifiable (Table 16, Boxes A, B, and C). Although of lesser clinical applicability, some biomarkers appear to be useful in stratifying bleeding risk (Table 16, Box D).^17^

**Table 16:** Risk factors for bleeding in oral anticoagulant and antiplatelet therapy. Adapted from Hindricks et al.^17^

Non-modifiable
Age > 65 years
Previous major bleeding
Severe renal impairment (on dialysis or on renal transplant)
Severe hepatic dysfunction (cirrhosis)
Malignant disease
Genetic factors (eg, CYP2C9 polymorphisms)
Previous stroke, small-vessel disease, etc.
Diabetes mellitus
Cognitive disability/dementia
**Potentially modifiable**
Extreme frailty and/or excessive risk of falls
Anemia
Reduction platelet count or function
Renal impairment with CrCl < 60 mL/min
Vitamin K antagonist management strategy
**Modifiable**
Hypertension/elevated systolic blood pressure
Concomitant antiplatelet/non-steroidal anti-inflammatory drug therapy
Excessive alcohol intake
Non-adherence to oral anticoagulant therapy
Hazardous hobbies/occupations
Bridging therapy with heparin
International normalized ratio control (target 2.0-3.0)
Time in therapeutic range > 70%
Appropriate choice of OAC and correct dosing
**Biomarkers**
GDF-15
Cystatin C/CKD-EPI
cTnT-hs
von Willebrand factor (+ other coagulation markers)

CrCl: creatinine clearance; OAC: oral anticoagulation

Identifying modifiable and potentially modifiable risk factors for bleeding is of fundamental importance before beginning anticoagulant therapy, making it possible and/or safer. Factors such as uncontrolled high blood pressure, alcohol consumption, and non-hormonal anti-inflammatory drug or aspirin therapy, for example, can be modified and excluded as bleeding risk factors. To achieve these objectives, a good doctor-patient relationship is necessary, encouraging treatment adherence by discussing the risks and benefits of treatment.

It must also be considered that risk factors for bleeding are dynamic and may vary over time. Thus, patients who have not begun OAC therapy due to bleeding risk must be periodically reevaluated to begin it as soon as these factors are controlled.^134^

### 6.8. Choosing an Oral Anticoagulant

Because no clinical trials have directly compared DOACs, it is not possible to determine whether one DOAC is superior to or safer than another. Individual clinical profiles that are more favorable to specific DOACs are summarized in Table 17.

**Table 17:** Suggested direct-acting oral anticoagulants for different clinical situations in patients with AF

Clinical situation	Suggested oral anticoagulant
Older adult	Apixaban or edoxaban
Multiple comorbidities	Apixaban or edoxaban
High risk of stroke and low risk of bleeding	Dabigatran
High risk of bleeding	Apixaban or edoxaban
High risk of gastrointestinal bleeding	Apixaban
Renal dysfunction	Apixaban or edoxaban
Moderate/severe mitral stenosis or mechanical valve prosthesis	Warfarin
Valve bioprosthesis	Rivaroxaban
Acute coronary syndrome	Apixaban + clopidogrel (ticagrelor may be considered. Avoid prasugrel)
Chronic coronary syndrome	Any DOAC without an antiplatelet agent
Dosing convenience	Edoxaban or rivaroxaban

AF: atrial fibrillation; DOAC: direct-acting oral anticoagulant. Note: No clinical trials have directly compared different DOACs. These suggestions are based on subgroup analyses of pivotal studies and do not contraindicate other DOACs. Patients must be treated on a case-by-case basis and the risk of stroke and bleeding must be discussed with patients and their families for shared decision-making.

## 7. Rate or Rhythm Control in Atrial Fibrillation

AF treatment is based on rhythm control, ie, maintaining sinus rhythm and biventricular pacing, keeping the patient in AF but controlling the response.^17,260^ Strategy selection depends on the clinical characteristics of each patient.

### 7.1. Antiarrhythmic Drugs

The Vaughan-Williams classification system includes 4 distinct AAD classes.^261^ Class I consists of sodium channel blockers, class II consists of beta-blockers, class III consists of potassium channel blockers, and class IV consists of non-dihydropyridine calcium channel blockers. The present guidelines will focus on agents commercially available In Brazil.

#### 7.1.1. Propafenone

Propafenone is a fast sodium channel blocker (Vaughan-Williams class Ic). It is used to maintain sinus rhythm and is recommended for patients without myocardial ischemia or structural heart disease. It is contraindicated in LV systolic dysfunction, since drugs in this class, such as flecainide, are associated with increased mortality in these patients, as demonstrated in the CAST trial.^262^ Its effectiveness has also been proven for acute reversal of AF episodes, through the "pill-in-the-pocket" approach, in a single dose of 450 to 600 mg, which can be increased by additional doses of 300 mg every 8 hours.^263^

After oral absorption, propafenone has a short half-life (around 5 to 8 h), requiring continuous use (2 or 3 daily doses). The usual dose varies from 150 mg twice daily to 300 mg in 3 daily doses.

The adverse effects of propafenone occur more frequently at the beginning of treatment and with higher doses. Sinus bradyarrhythmia or atrioventricular block is associated with concomitant use of other negative chronotropic drugs or in susceptible patients. Its most concerning adverse effect, especially at high doses, (including the "pill-in-the-pocket" approach), is type Ic AFL, since, apart from often being poorly tolerated, it may be associated with 1:1 AV conduction and rapid ventricular response.^264^

Relatively common complaints include a metallic taste and neurological symptoms, such as dizziness, headache, insomnia, blurred vision, and nightmares. Patients with a history of bronchospasm may experience exacerbated attacks due to its beta-blocking effect. Other rare effects include lupus-like syndrome, agranulocytosis, and liver dysfunction.^265^

#### 7.1.2. Amiodarone

The predominant effect of amiodarone, an AAD frequently used to control rhythm in AF, is potassium channel block, which is characteristic of class III agents. However, its antiarrhythmic properties go beyond this effect, blocking sodium and calcium channels and non-competitive blocking of beta- and alpha-adrenergic receptors, ie, it has characteristics of all classes of AADs. Although it can increase the QT interval and have a pro-arrhythmic effect, the incidence of torsades de pointes is low, generally related to high doses, electrolyte disturbances (mainly hypokalemia), and the concomitant use of drugs that contribute to increased QT interval, such as other antiarrhythmic agents, phenothiazines, tricyclic antidepressants, thiazide diuretics, etc.

Amiodarone can be administered intravenously or orally. Although the former is also used to treat acute episodes of AF, the rate of reversion to sinus rhythm in this situation is relatively low. However, oral administration requires more time to reach the maximum pharmacological effect, generally a few months. Since it is a fat-soluble drug, tissue accumulation facilitates maintenance of therapeutic levels, which can be observed months after discontinuance. However, its affinity for certain tissues, such as hepatic and pulmonary, may be responsible for associated toxicity in these organs.^266^

Amiodarone, the most effective antiarrhythmic agent, is twice as effective as propafenone and sotalol in preventing recurrent episodes.^267^ Its great advantage is that it can be used in patients with structural heart disease and LV systolic dysfunction to maintain sinus rhythm and control HR, the latter in exceptional situations after treatment with beta-blockers and digitalis.

Adverse effects are the greatest limitation of long-term amiodarone therapy, leading to discontinuance in around 15% of patients. This can be minimized through doses < 200 mg/d, which maintains efficacy in a considerable number of patients. Adverse effect rates range from 6% at doses of 200 mg to 2% at lower doses.^268^ Hyperthyroidism occurs in approximately 1% of patients, a situation that is more difficult to control, often requiring discontinuance. Pulmonary toxicity, which leads to pulmonary fibrosis, occurs in approximately 3% of patients.^269^ This condition is generally related to high doses, and in some cases it may be reversible with discontinuance. Corneal impregnation is common but rarely translates into decreased visual acuity. Patients on long-term amiodarone therapy must periodically undergo renal and hepatic function assessment, thyroid hormone measurement, chest x-ray, and ophthalmological assessment, in addition to other specific tests depending on suspected involvement of other organs.^270^

#### 7.1.3. Sotalol

Sotalol molecules consist of a racemic mixture of the isomers d-sotalol and l-sotalol. The right-handed form has class III antiarrhythmic properties (potassium channel block), while the left-handed form is predominantly a beta-blocker. It is excreted through the kidneys, and lower doses must be used in patients with CrCl < 60 ml/min. It is contraindicated when CrCl is < 40 ml/min.

In patients with AF, sotalol's efficacy is similar to propafenone and inferior to amiodarone. It is particularly useful in patients with ischemic heart disease and LVEF > 40%, but it is not recommended for patients with severe systolic dysfunction due to increased mortality, as was observed in the SWORD trial. It should also be avoided in patients with significant myocardial hypertrophy.^271^ The recommended dose varies between 160 and 320 mg/d divided into 2 daily doses. Its extracardiac adverse effects are common to other beta-blockers, and it is generally well tolerated at normal doses.

### 7.2. Rate Control Drugs

#### 7.2.1. Beta-blockers

Beta-blockers, class II antiarrhythmics in the Vaughan-Williams system, are the preferred agents for HR control, both in the acute phase and in outpatient follow-up. These drugs predominantly block beta-adrenergic receptors, although alpha-adrenergic blockade is another effect, causing peripheral vasodilation, which becomes less pronounced with increasing cardioselectivity. β1 receptors, which are more numerous in the sinus and atrioventricular nodes, play an important role in modulating sinus rhythm and atrioventricular conduction. β2 adrenergic blockade promotes bronchospasm, a limiting effect in individuals with bronchial hyperreactivity, such as asthmatics and those with chronic obstructive pulmonary disease. Bisoprolol, metoprolol, and atenolol are cardioselective (in order from most to least), and have the least potential for extracardiac adverse effects. Bisoprolol, carvedilol, and metoprolol can be used in patients with LV systolic dysfunction.

In persistent/permanent AF, beta-blockers are the most widely used agents to reduce ventricular response.^272^ Despite their recognized effectiveness for controlling HR, their impact, ie, reduced mortality and hospitalizations and improved quality of life, has been the subject of much discussion in recent years. The benefits of beta-blockers are more pronounced in patients with HF and after myocardial infarction, mainly in relation to AF incidence.^273^ However, the expected mortality reduction has not been observed in patients who have already developed AF.^274^ More consistent evidence is needed to determine the superiority of a specific class of medications in HR control for patients with AF.

Depending on patient sensitivity, there can be unwanted cardiovascular effects, such as significant bradycardia and atrioventricular block, which requires dose reduction or even discontinuation. Extracardiac effects have been reported in a reasonable percentage of patients and are less common in more cardioselective agents. Beside bronchospasm, the most commonly reported adverse effects include fatigue, dizziness, insomnia, nightmares, and erectile dysfunction. In diabetic patients, signs of hypoglycemia may be masked.

#### 7.2.2. Calcium Blockers

Non-dihydropyridine calcium channel blockers, such as verapamil and diltiazem, have pharmacological effects similar to beta-blockers, decreasing sinus rhythm and atrioventricular conduction. They do this by blocking slow calcium current, acting predominantly on the slow response cells of the sinus and atrioventricular nodes. They are classified as class IV antiarrhythmics and are effective in rate control for patients with AF.^275^ They can be used as a first-line agent or as an alternative to beta-blockers, especially in patients contraindicated for beta-blockers or who have experienced adverse effects from them. Due to their negative inotropic effect, they should not be used in patients with LVEF < 40%.

#### 7.2.3. Digoxin

Digoxin therapy should be restricted to patients with inadequate rate control who are already using beta-blockers or calcium blockers or who are intolerant to them.^276^ Digoxin should be avoided in patients with advanced kidney disease. When association with amiodarone is necessary, it must be administered with caution due to the toxicity risk.

Table 18 provides useful information about AADs used to treat AF.

**Table 18:** Practical aspects of antiarrhythmic drugs used to treat atrial fibrillation

Drug	Route of Elimination	Dosage	Extracardiac adverse reactions	Main Drug Interactions
Propafenone	Hepatic/renal	150-300 mg (2-3x/day)	Bronchospasm, dizziness, visual blurring, metallic taste, headache, insomnia	Warfarin, dabigatran, edoxaban, ketoconazole, fluoxetine, paroxetine
Amiodarone	Hepatic	100-400 mg (1x/day)	Hypo- and hyperthyroidism, pulmonary fibrosis, neuropathy, photosensitivity, hepatotoxicity	Digoxin, diltiazem, verapamil, warfarin, dabigatran, edoxaban, phenytoin, cyclosporine, statins, sildenafil, tricyclic antidepressants
Beta-blockers				
Atenolol	Hepatic/renal	25-100 mg (1-2x/day)	Bronchospasm, fatigue, dizziness, insomnia, nightmares, cold extremities, nausea, constipation, erectile dysfunction, weight gain, hyperglycemia, attenuation of signs of hypoglycemia	Amiodarone, fluoxetine, paroxetine, digoxin, clonidine, phenobarbital, NSAIDs
Metoprolol	Hepatic/renal	25-200 mg (1-2x/day)		
Propranolol	Hepatic/renal	40-240 mg (2-3x/day)		
Bisoprolol	Hepatic/renal	1.25-10 mg (1x/day)		
Carvedilol	Hepatic	3.125-50 mg (2x/day)		
Sotalol	Renal	80-160 mg (2-3x/day)		
Calcium blockers				
Verapamil	Hepatic/renal	80-160 mg (2-3x/day)	Fatigue, dizziness, constipation, headache, nausea, ankle edema, constipation, gingival hyperplasia (verapamil)	Digoxin, ivabradine, colchicine carbamazepine, phenytoin, phenobarbital, cyclosporine, statins, dabigatran, edoxaban
Diltiazem	Hepatic/renal	30-120 mg (2-3x/day)		
Digoxin	Renal	0.125-0.25 mg (1x/day)	Dizziness, blurred vision, vomiting, diarrhea	Alprazolam, cyclophosphamide, doxorubicin, methotrexate, ketoconazole, beta-blockers, amiodarone, verapamil, diltiazem

NSAIDs: non-steroidal anti-inflammatory drugs.

### 7.3. Choosing between Rhythm and Rate Control

Rhythm control for AF should aim to improve symptoms and prevent cardiovascular complications. Two therapeutic strategies are possible in this context: rhythm control (maintaining sinus rhythm) and rate control (without prioritizing arrhythmia reversal). The question of which strategy is superior has been the subject of controversy for more than 20 years.^277,278^

From a physiological point of view, rhythm control has numerous advantages in AF, such as maintaining atrial contraction and normal atrial structure, atrioventricular synchronization, rate response to different demands, RR interval regularity, etc. These factors are related to lower thrombogenicity, symptom reduction, and preserved LV systolic function, thus reducing the risk of events that affect prognosis.

Rhythm control is currently considered superior to rate control in patients with AF. The EAST AFNET 4 trial found lower mortality and a lower incidence of both thromboembolic events and hospitalization in patients where the goal was rhythm control.^279^ A 2023 meta-analysis, where it was found a > 20% reduction in mortality outcomes and the incidence of ischemic stroke and hospitalization for HF.^280^

Rhythm control should be prioritized in patients with a greater chance of maintaining sinus rhythm (age < 65 years with mild-to-moderate atrial remodeling). Similarly, rhythm control is preferred in situations with a significant impact on reducing morbidity and mortality in the medium and long term, such as: symptomatic patients, those with difficult heart rate control, tachycardiomyopathy, HF, and patients at high thromboembolic risk.

Rate control may be a strategy in situations of very old or asymptomatic patients with normal ventricular function. Recommendations on rhythm or rate control for patients with AF are shown in Box 13.

**Box 13:** Recommendations on rhythm or rate control in patients with atrial fibrillation

Recommendations	Class of recommendation	Level of evidence
Rhythm control is recommended as the preferred treatment strategy.	**I**	**A**
The heart rate control strategy can be used in asymptomatic patients or those with a low likelihood of maintaining sinus rhythm.	**IIa**	**A**

### 7.4. Rate Control

Rate control during AF is important to avoid the development of tachycardiomyopathy or medium-to-long term symptoms. Some conditions, such as increased vagal tone and drug therapy that slows atrioventricular conduction, reduce ventricular response during AF. However, conditions such as hyperthyroidism, excess catecholamine, and Wolff-Parkinson-White syndrome may be responsible for higher ventricular rates.

The most common forms of pharmacological rate control are calcium channel antagonists (diltiazem, verapamil), beta-blockers, digoxin, and amiodarone. Atrioventricular junction ablation with pacemaker implantation is a non-pharmacological option for rate control.

#### 7.4.1. Therapeutic Goal

When rate control is used, the resting HR target must be observed. Holter monitoring is an important tool for quantifying the mean HR over 24 hours. Heart rate control should aim for a resting HR < 80 bpm, but more lenient HR control (resting HR < 110 bpm) in asymptomatic patients was not associated with a higher rate of cardiovascular events.^281^ Because patients with HR > 100 bpm have a higher 1-year risk of death or HF, this value is suggested as the upper limit for the therapeutic goal.^281^

#### 7.4.2. Acute Control

Although electrical cardioversion is generally indicated for acute AF with hemodynamic instability, in some situations it is impossible or has been unsuccessful. In such patients, as well as in those with rapid ventricular response but no hemodynamic instability, acute rate control is appropriate. Secondary causes of instability, such as dehydration, bleeding, anemia, or infection must always be ruled out, especially in emergency care.

The most common drugs for acute control are beta-blockers, such as metoprolol or esmolol. Since these drugs, especially intravenous forms, can lead to arterial hypotension, the most recommended beta-blocker is esmolol, due to its shorter half-life. A recent comparative meta-analysis indicated that diltiazem was more effective than metoprolol for acute rate control.^282^ Nevertheless, intravenous calcium antagonists are less available in Brazil.

When adequate control is not achieved through these drugs, the next option is to add intravenous digitalis, while the final pharmacological option is intravenous amiodarone. An association of intravenous magnesium sulfate and drugs that block the AV node can also play a role in acute control of AF response. The most likely mechanism is to block slow calcium channel entry into the AV node.^283^ The main advantage of this drug is its safety.^283-286^ A recent meta-analysis found that intravenous magnesium was superior to conventional treatment for biventricular pacing (63% vs 40%; OR, 2.49 [CI, 1.80-3.45]) and was moderately effective in sinus rhythm conversion (21% vs 14%; OR, 1.75 [CI, 1.08-2.84]).^285^ Ivabradine may help control ventricular response.^287,288^

To transition from intravenous to oral administration, the same amount must be administered orally as was administered intravenously within 24 hours in ≥ 1 dose, depending on tablet formulation. Table 19 shows the drugs used for acute rate control.

**Table 19:** Dosage of intravenous drugs used for acute rate control

Drug	Dose
**Esmolol**	500 µg/kg bolus for 1 min; maintenance 10-40 µg/kg/min (1-10 µg in cases of ventricular dysfunction)
**Metoprolol tartrate**	2.5-5 mg IV bolus; maximum 4 doses
**Amiodarone**	300 mg diluted in 5% glucose serum for 30-60 min; maintenance 900-1200 mg IV over 24 hours via the central route
**Magnesium Sulfate**	Low dose: 4.5 g diluted in 100 ml infused over 30 m High dose: 9 g diluted in 100 ml infused over 30 m
**Deslanoside**	Dosage: Rapid digitalization (24 hours) in urgent cases: 0.8–1.6 mg, administered intravenously or intramuscularly, divided into 1–4 doses. Maintenance: 0.2–0.6 mg/day, administered intramuscularly or intravenously.

#### 7.4.3. Long-term Control

Long-term rate control is generally achieved through beta-blockers or calcium channel antagonists, medications that work both at rest and during exertion. Digoxin, which has less effect during high adrenergic activity, and amiodarone are also occasionally used alternatives.^289^ In isolated or combined therapy, rate control is achieved in more than 80% of patients, but this must involve careful monitoring.

Beta-blockers can be used in patients with normal or reduced systolic function and are especially indicated in patients with ischemic heart disease and high adrenergic activity. In cases involving ventricular dysfunction, metoprolol succinate, carvedilol, and bisoprolol are the most indicated drugs.

Calcium channel antagonists reduce cardiac inotropism and should not be used in patients with systolic dysfunction. Verapamil more effectively reduces ventricular response than diltiazem, but it also has a greater negative inotropic effect. Calcium channel antagonists are generally the preferred option in patients with bronchopulmonary diseases when beta-blockers are contraindicated.

Digoxin is more frequently used in patients with systolic ventricular dysfunction, although it is actually more common as a combined therapy with beta-blockers or calcium channel antagonists. Since it effect is lost during exercise, it is rarely used as monotherapy, especially in young patients, even though recent studies have found good quality of life results compared to bisoprolol in patients with HF.^290^ Nevertheless, digoxin therapy requires caution because the therapeutic range is close to the intoxication range, and studies have reported increased mortality, especially with serum digoxin concentrations > 1.2 ng/mL.^291^

Oral amiodarone may occasionally be used for ventricular rate control, but it is quite uncommon due to its high long-term side effect profile. Table 20 describes the drugs and dosage used for long-term ventricular rate control in AF.

**Table 20:** Daily oral doses used in long-term heart rate control

Droga	Dose
**Diltiazem**	180-360 mg
**Verapamil**	80-480 mg
**Atenolol**	25-100 mg
**Bisoprolol**	1.25-20 mg
**Carvedilol**	6,25-100 mg
**Metoprolol succinato**	50-400 mg
**Metoprolol tartarato**	50-200 mg
**Nebivolol**	2.5-10 mg
**Digoxina**	0.0625-0.25 mg
**Amiodarona**	100-400 mg (usually 200 mg 5-7x/week)

#### 7.4.4. Atrioventricular Node Ablation

AV node ablation with permanent pacemaker implantation is an extremely efficient alternative when pharmaceutical ventricular response control has failed or is not tolerated. Despite its invasiveness, its main advantages, apart from its efficiency, are its simplicity and relative low risk, resulting in improved symptoms, quality of life, and exercise capacity.^17,292,293^

This strategy can be used in patients who already have an implantable cardiac device, whether due to sinus node disease, cardiac resynchronization, or cardiac defibrillation.^294^ In patients without a cardiac device, the implantation should be performed first, and a few weeks later, AV node ablation can be carried out.

When the ablation is performed, the device must be inhibited so that the His bundle electrogram can be mapped. The ablation site is generally the right side of the AV node in a more proximal location, based on electrograms of the atrium, His bundle and ventricle to obtain a more proximal block. In case of poor pacemaker capture, a junctional leak may be better tolerated.^295^ In some situations where this region cannot be blocked, a more distal block can be used in the His bundle region or, in patients with right bundle branch block, the His bundle region should be approached through the left ventricle.

It is extremely important that in the first weeks after AV node ablation, pacing must be maintained at 80-90 bpm,^17^ since cases of spontaneous polymorphic ventricular tachycardia have been reported after AV node ablation, a complication that this strategy may prevent.^296,297^

In some patients with cardiac resynchronization and AF, AV node ablation may be essential to ensure a higher pacing rate.^298,299^ The CERTIFY clinical trial^300^ found that, in patients with AF undergoing cardiac resynchronization therapy, total mortality (6.8 vs 6.1 per 100 person-years) and cardiac mortality (4.2 vs 4.0) were similar between those who received AV junction ablation and patients in sinus rhythm.

Recently, physiological pacing techniques have been developed for the His bundle and left branch.^294^ In patients with AF and implantable cardiac devices treated with His bundle pacing^301,302^ and left bundle branch pacing,^303^ AV node ablation is safe and effective.

Few studies have compared rhythm control through pulmonary vein isolation with AV node ablation and biventricular pacing. The PABA-CHF trial included patients with HF (New York Heart Association class II or III) and ventricular dysfunction (LVEF ≤ 40%), finding, in 6 months of follow-up, better EF, quality of life, and 6-minute walk test results in the group who underwent pulmonary vein isolation than in those who underwent AV node ablation and biventricular pacing.^304^ In a sample of patients from the German Ablation Registry who had either undergone pulmonary vein isolation (4444, 95%) or AV node ablation (234, 5%), the AV node ablation patients were on average 10 years older (71 [SD, 10] years vs 61 [SD, 10] years, p < 0.001), had more cardiovascular comorbidities (44% vs 7%, p < 0.001), and had a higher mortality rate in 1 year of follow-up (9.8% vs 0.5%). However, symptoms improved for the majority of patients in both groups (80%), although there were fewer hospitalizations in the AV node ablation group (31% vs 18%, p < 0.001).^305^

Box 14 presents recommendations on AV node ablation.

**Box 14:** Recommendations on Atrioventricular Node Ablation

Recommendations	Class of recommendation	Level of evidence
AV node ablation is recommended for patients with an implantable cardioverter defibrillator for whom AF therapies are inappropriate and in those with atrial flutter with rapid ventricular response who are ineligible for pulmonary vein isolation.	**I**	**B**
In patients with AF and rapid ventricular response who undergo AV ablation, initial heart rate pacing should be maintained between 80 and 90 bpm to reduce the risk of sudden death.	**I**	**C**
AV node ablation is recommended for patients on biventricular, His bundle, or left branch pacing resynchronization therapy who have AF or atrial flutter with a low pacing rate and who are ineligible for pulmonary vein isolation.	**I**	**B**
AV node ablation should be considered for rate control in patients with permanent pacemakers who have not responded to or are intolerant to rhythm or rate control strategies	**IIa**	**B**
AV node ablation should be considered for rate control in patients who have not responded to or are intolerant to rhythm or rate control strategies and are ineligible for pulmonary vein isolation, given that these patients will be pacemaker dependent	**IIb**	**B**

AF: Atrial fibrillation; AV: Atrioventricular; HR: Heart rate.

### 7.5. Rhythm Control – Special Situations

#### 7.5.1. Electrical Cardioversion

AF is asymptomatic or causes mild symptoms in most patients. However, in cases involving hemodynamic instability, synchronized electrical cardioversion should be performed immediately (200 J biphasic).^17,306^ In stable cases, electrical cardioversion restores sinus rhythm more quickly and effectively than AADs, reducing hospitalization time, although it requires adequate fasting and sedation. Through anteroposterior positioning of the pads or paddles, more energy can pass through the LA. However, in a recent meta-analysis, there was no difference in the reversal to sinus rhythm when comparing the antero-posterior and antero-lateral positions.^307^ Pretreatment with AADs facilitates cardioversion, mainly by preventing immediate recurrence. A recent randomized trial demonstrated that shock application at maximum energy is more effective than a gradual increment and could be considered an alternative in cases refractory to usual dose (200 J). Active compression during electrical cardioversion also helps achieve sinus rhythm.^308^

#### 7.5.2. Chemical Cardioversion

Due to their practicality, AADs are a common method of reverting to sinus rhythm. However, these drugs can cause serious adverse effects, such as torsades de pointes, especially in older patients with multiple comorbidities and electrolyte alterations (hypokalemia). Furthermore, the efficacy of AADs in reversing AF is relatively low compared to placebo, partly because spontaneous reversal is high, especially in paroxysmal forms of AF. In a recent randomized clinical trial, expectant management (using only rate control and cardioversion, if necessary, within 48 hours) was equivalent to immediate cardioversion in patients with paroxysmal AF.^309^ This strategy can be considered in patients with recent-onset AF, although it is difficult to apply in Brazil, since the drugs available for pharmacological cardioversion are limited to amiodarone and propafenone, and only amiodarone can be used in patients with structural heart disease. The recommended dosage and main side effects of drugs used for pharmacological cardioversion are listed in Table 21.

**Table 21:** Drugs and dosages used to reverse atrial fibrillation and maintain sinus rhythm

Drug	Reversion	Rhythm Maintenance	Adverse events
**Amiodarone**	IV 150-300 mg diluted in SG 5% (infuse in 15-30 min) followed by 900 mg in 24 h	Attack: 600 to 800 mg/day in divided doses up to a total of 10 g. Maintenance: 200-400 mg 1 x a day	Bradycardia, TdP, photosensitivity
			Hypo-/hyperthyroidism
			Pulmonary, hepatic toxicity, polyneuropathy
**Propafenone**	450-600 mg single oral dose or IV 1.5 to 2.0 mg/kg in 10 to 20 min	150-300 mg 3 x a day	Bradycardia, proarrhythmia, gastrointestinal disorders, headache
**Sotalol**	Not recommended	80-160 mg 2 x a day	Bradycardia, TdP, bronchospasm

IV: intravenous; TdP: torsades de pointes.

Elective cardioversion (electrical or chemical) should be preceded by an assessment of embolic risk, which is discussed in section 6.5.1.

#### 7.5.2.1. The "Pill-in-the-pocket" Approach

Although paroxysmal AF is self-limiting by definition, the pill-in-the-pocket approach has been used to shorten AF duration and reduce emergency room visits, especially in patients with symptomatic and infrequent episodes. Likewise, these regimens can be used in recent-onset persistent AF (< 7 days), since it is known that the sooner they are used, the better the reversion results. Reversion to sinus rhythm ranges from 56% to 94%.^263,310^ The pill-in-the-pocket approach should be used only in patients whose heart is structurally normal or has minimal alterations, and it should be avoided in patients with ventricular hypertrophy (> 13 mm), ventricular dysfunction, or ischemic cardiomyopathy.

After the approach has been defined, a dose of beta-blocker or calcium antagonist should be administered before the AAD to avoid the occurrence of 1:1 AFL, which can occur in 3% to 7% of cases.^311^ Hence, the initial application should always be performed in a hospital environment to assess the safety of this strategy. Propafenone is used in a single dose of 600 mg (2 tablets), which can be reduced to 450 mg in people weighing < 70 kg.^263^

A small case series investigated additional doses of propafenone in long-term users, aiming at a pill-in-the-pocket approach, while respecting a maximum dose of 900 mg/day. For patients who already use 600 mg/day, adding only 300 mg to the regimen resulted in a 77% success rate in reversal.^312^

### 7.6. Pharmacological Sinus Rhythm Treatment

Despite its limitations, pharmacological treatment remains a cornerstone of AF treatment. Commonly used AADs for maintaining sinus rhythm in Brazil are propafenone, sotalol and amiodarone. The choice of AAD for maintaining sinus rhythm should be based on individual patient characteristics, such as risk factors and comorbidities. Bear in mind that these drugs have potential side effects and contraindications (Table 21). Figures 17 and 18 demonstrate the rhythm control strategies in patients with symptomatic AF without structural heart disease and in patients with heart failure, respectively.

**Figure 17:** Rhythm control strategies in patients with symptomatic atrial fibrillation (AF) without structural heart disease.

**Figure 18:** Rhythm control strategies in patients with symptomatic atrial fibrillation (AF) and heart failure. AV: atrioventricular; LVEF: left ventricular ejection fraction. *Tachycardiomyopathy, young patients with few comorbidities, minimally dilated atrium, paroxysmal or recent persistent forms, early-stage heart failure, ventricle with minimal fibrosis. ** Advanced heart failure, ventricular fibrosis, advanced atrial fibrotic cardiomyopathy, recurrence after ablation, very elderly patients with multiple comorbidities, long-standing AF. # AV node ablation in cases of failure to control frequency and rhythm.

Propafenone has been used as a first-line drug in individuals with a structurally normal heart, but it is contraindicated in those with structural heart disease (CAD, HF) due to the risk of ventricular arrhythmia and increased mortality.^17,22^ Long-term rhythm control with propafenone is considered satisfactory. In a study of patients treated with sustained-release propafenone 325 mg twice daily, the 1-year recurrence rate was 41% (vs 68% for placebo).^313^

The efficacy of sotalol is equivalent to propafenone for maintaining sinus rhythm.^267^ However, its use is more restricted, which is mainly due to the risk of proarrhythmias (torsades de pointes). This drug can prolong atrial and ventricular refractoriness and, consequently, can increase the QT interval. Risk factors include bradycardia, female sex, renal impairment, and LV hypertrophy.^314^ Therefore, in patients with AF who are treated with sotalol, monitoring the QT interval, serum potassium levels, CrCl, and other risks of proarrhythmia is recommended. Sotalol is contraindicated in patients with HF with reduced EF (HFrEF).^17^

Amiodarone has been shown to be the most effective AAD for maintaining sinus rhythm,^267,315^ but it is associated with cardiac and non-cardiac complications. Although amiodarone can be used to maintain sinus rhythm in all patients with AF, due to the risk of adverse events, it is especially recommended for patients with structural heart disease (CAD, valvular disease, and HFrEF).^267,316^

## 8. Atrial Fibrillation Ablation

### 8.1. Mapping and Ablation Technologies

#### 8.1.1. Three-Dimensional Mapping Systems

After Haïssaguerre et al.^317^ discovered the role of arrhythmogenic foci in pulmonary veins during AF initiation, electrical pulmonary vein isolation has become the pillar of arrhythmia treatment.^318,319^ To correctly identify the anatomical substrate and facilitate catheter navigation in the LA, auxiliary imaging techniques such as 3D electroanatomical mapping and intracardiac echocardiography have been incorporated. Today, these technologies are considered standard, reducing procedure time and exposure to fluoroscopy.^320,321^

Mapping systems assist with direct visualization of the catheter while navigating 3-dimensional anatomy, an improvement over the limited visualization provided by radioscopy, which was traditionally used in electrophysiology laboratories. The three most commonly used systems in Brazil are CARTO (Biosense Webster), EnSite (Abbott Medical), and Rhythmia (Boston Scientific). The technology is based on the three-dimensional reconstruction of the atrial cavity and pulmonary veins through the creation of an electromagnetic field (CARTO and Rhythmia) or changes in voltage and tissue impedance (NavX), accurately defining the anatomy, functional substrate, and electrical activation, in addition to marking the radiofrequency lesions on the created map, reducing the complexity of the technique and the need for fluoroscopy.^322^

#### 8.1.2. Intracardiac Echocardiography in Atrial Fibrillation Ablation

As an imaging modality for AF ablation, intracardiac echocardiography was initially focused on echo-guided transseptal puncture,^323^ precisely identifying the oval fossa and determining the best puncture site, thus allowing safe access to the LA without iodinated contrast.^324,325^ More recently, intracardiac echocardiography has been used during all stages of the ablation procedure to reduce and even eliminate fluoroscopy, especially when associated with electroanatomical mapping systems.^326^

Intracardiac echocardiogram is extremely useful during electroanatomical mapping, providing extremely relevant real-time information, such as the exact location of the pulmonary vein ostia and antra, as well as regions that are difficult to delimit, such as the cuneiform fold (limits between the left atrial appendage and the anterior region of the left pulmonary veins) (Figure 19B), supernumerary pulmonary veins, and anomalous drainage. The esophagus can also be visualized though intracardiac echocardiography,^327^ identifying potential lesions.^328^

During pulmonary vein isolation, adequate radiofrequency (RF) application is directly dependent on the time the ablation catheter is in contact with atrial tissue. Unstable catheter positioning can result in future reconnection due to a non-transmural lesion. Intracardiac echocardiography allows real-time monitoring of catheter-tissue contact.

However, excessive tissue reactions can occur in RF ablation, which can be easily identified as hyperreflective areas in intracardiac echocardiography (Figure 19C).^329^ This phenomenon, usually a precursor to steam popping, can lead to serious complications, such as cardiac perforation. Thus, real-time monitoring of tissue lesion formation through intracardiac echocardiography can help prevent complications. In fact, a recent large study of > 100,000 patients undergoing AF ablation found that not using intracardiac echocardiography was associated with a 4.8-fold increased risk of cardiac perforation.^330^

Due to its real-time ultrasound monitoring capability, intracardiac echocardiography allows early detection of serious complications, such as pericardial effusion (Figure 19D), including diagnosis prior to the occurrence of hypotension secondary to cardiac tamponade. Visualizing the pericardial space at the beginning of ablation and periodically throughout the procedure is desirable, allowing comparison of images captured at different times and detection of early-stage pericardial effusion, prior to its hemodynamic complications. Thus, early therapeutic measures, such as anticoagulation reversal and percutaneous drainage, can be taken to prevent hemodynamic collapse.

The formation of intracardiac thrombi during catheter ablation is also associated with high embolic risk, and intracardiac echocardiography is the ideal tool for rapid detection and adequate treatment.^331-333^ Another pre-procedure measure is echocardiography in specific regions of the heart (eg, the coronary sinus and pulmonary artery), which can detect thrombi in the LAA in a similar manner to transesophageal echocardiography.^334-336^ When evaluating patients in whom an LAA thrombus was detected during transesophageal echocardiography, intracardiac echocardiography of the pulmonary artery can detect false positive cases.^337^

Together, these measures ensure the safety of ablation and have been responsible for its rapid dissemination. In a study investigating transseptal access alone, intracardiac echocardiography was an independent factor in preventing complications when accessing the LA.^338^ Complication rates and fluoroscopy requirements have been significantly reduced in AF ablation procedures involving intracardiac echocardiography.^339-341^ Although it entails additional costs,^342^ intracardiac echocardiography reduces the average length of hospital stay, which has a positive impact on hospital costs.^343^ Figure 19 reviews the importance of intracardiac echocardiography in AF ablation.

**Figure 19:** ntracardiac echocardiography during an atrial fibrillation procedure. A: Visualization of the needle in the fossa ovalis during transseptal puncture; B: With the probe positioned in the right ventricle, an image of the left atrium reveals the left atrial appendage and the ridge between the left atrial appendage and the anterior region of the left pulmonary veins; C: RF application near the left inferior pulmonary vein. Hyperreflective tissue adjacent to the catheter indicates effective application, so the catheter should be moved to another area to avoid excessive injury; D: Pericardial effusion is identified by positioning the intracardiac echocardiography probe in the right ventricle at the beginning of the procedure. The procedure was safely performed by comparing images captured at the beginning and end of the ablation. RF: radiofrequency.

#### 8.1.3. Ablation Technologies

Over the course of more than 20 years of development, there have been numerous technological advances in mapping systems, catheters, and energy sources for tissue injury. Conventional point-to-point RF ablation is the most widely used type: irrigated ablation catheters and contact force sensors are recommended to increase the effectiveness of permanent isolation and thermal lesion safety in the LA.^344-347^

Regarding the long-term effectiveness of radiofrequency ablation, greater pathophysiological understanding of lesion formation has led to improvements in catheters and other technologies, as well as the creation of lesion quality indexes that incorporate contact force, power, and application time. Understanding how these factors interact is essential for long-term lesion durability, reducing pulmonary vein reconnection and arrhythmia recurrence.^348^

Another validated approach is cryoablation, in which a balloon catheter is positioned in the antrum of the pulmonary veins and can simultaneously lesion the entire circumference in contact with the tissue, which reduces the total procedure time, but it involves greater exposure to fluoroscopy. Randomized trials involving both strategies have shown comparable results, with the energy source selected according to the available technology and the electrophysiologist's experience.^348,349^

Currently, contact force is mandatory when using point-to-point RF ablation and, if possible, it should be associated with intracardiac echocardiography for more effective ablation, better results, and fewer complications.^321,322,340-342^ Cryoablation does not require association with electroanatomical systems, although associating it with intracardiac ultrasound increases its safety and reduces X-ray exposure.

More recently, pulsed field ablation (electroporation) has been studied, in which high-energy, short-duration electrical impulses are applied to the target tissue, leading to cellular apoptosis. Since different tissue types have different thresholds for field-induced damage, this technology aims to selectively target cardiac cells, thus avoiding lesions to the esophagus or phrenic nerve.^350^ Initial clinical trials and registries show promising results for complete electrical isolation of veins, lesion durability, and clinical recurrence at 1 year of follow-up.^351-357^ The ADVENT trial, a non-inferiority study, demonstrated that pulsed-field ablation was equivalent to thermal ablation for a combined efficacy endpoint including failure to isolate pulmonary veins, atrial arrhythmia > 30 seconds, class I and III AAD therapy, and a safety endpoint.^356^ However, more robust data are still needed about the ideal protocol, number of pulses, frequency, and catheter configuration and orientation to ensure efficacy without complications, such as excessive muscle contraction, tissue heating, or bubble formation leading to embolic phenomena.

In conclusion, more and more tools are becoming available in clinical practice, and each has its pros and cons. The choice of which resource to use should be guided by cost, availability, and experience of the center and medical team.

### 8.2. Recommendations on Atrial Fibrillation Ablation

Clinical experience and technological advances have provided greater effectiveness and safety in AF treatment. AF ablation has a higher success rate than AADs,^358,359^ although there is still no clear evidence that it leads to reduced mortality.^360^ A meta-analysis of 5 clinical trials (994 patients) found lower recurrence of AFL and symptomatic AF, in addition to lower hospital admission rates, in patients treated with catheter ablation than in those treated with AADs.^361^ Decision-making about ablation should consider recurrence and complication rates. Several predictors of AF recurrence after catheter ablation have been identified. A study of 702 patients undergoing their first RF or cryoablation procedure identified that metabolic syndrome and loss of renal function are independent predictors of lower ablation efficacy.^362^ This analysis gave rise to the ALARMEc score (AF type, LA size, kidney disease, metabolic syndrome, and cardiomyopathy), which was subsequently tested as a recurrence predictor in 213 patients. However, in isolated analysis, only AF type (non-paroxysmal) and enlarged LA were found to be independent predictors.^363^ A Brazilian study of 95 patients found that larger anteroposterior LA diameter was a recurrence predictor (HR 2.58 for each adjusted mm, 95% CI 1.36-4.89).^364^ In the study that led to the easy-to-apply CAAP-AF score, 6 recurrence predictors were identified: coronary artery disease, increased LA diameter, advanced age, persistent or long-standing persistent AF, therapeutic failure of AADs, and female sex. With scores ranging from 0 to 13 points, the 2-year AF recurrence-free survival was 100% for scores of 0 and 29.1% for scores > 10 points in the development cohort. The results were replicated in the validation cohort, in which 2-year AF recurrence-free survival was 100% for scores of 0 and 51.3% for scores > 10 points.^365^ A score based on a European AF ablation registry was recently published, using artificial intelligence to identify post-procedure recurrence predictors. The 3128 patients were divided into derivation (80% of the sample) and validation (20% of the sample) cohorts. This score showed good accuracy for predicting 1-year recurrence (area under the curve, 0.72), with clinical variables such as age, sex, comorbidities, and AF type (paroxysmal or persistent), echocardiographic data of LV diastolic volume, LVEF, LA anteroposterior diameter, and CHA_2_DS_2_-VA score included in the final model.^366^

Since there is still no ideal score for predicting recurrence, when ablation is recommended a more intensive assessment of risk factors should is needed, with the factors adjusted to the patient's situation. What has been observed so far is that persistent AF, large LA diameter or volume, and comorbidities are related to higher recurrence rates.

Recommendations on AF ablation are shown in Box 15 and Figures 17 and 18.

**Box 15:** Recommendations on atrial fibrillation ablation

Recommendations	Class of recommendation	Level of evidence
General considerations		
Before considering catheter ablation of AF, the patient should be informed of its risks and outcomes.	**I**	**C**
Repeating ablation should be considered in patients with arrhythmia recurrence, initial symptom improvement after ablation, and previous procedural data suggesting a chance of maintaining sinus rhythm.	**IIa**	**B**
Catheter Ablation after Drug Therapy Failure		
Catheter ablation for AF is recommended in patients with symptomatic paroxysmal or persistent AF who are refractory or intolerant to at least one class I or III antiarrhythmic drug.	**I**	**A**
Catheter ablation for AF is recommended in patients with symptomatic paroxysmal or persistent AF who are refractory or intolerant to beta-blockers.	**IIa**	**B**
Catheter ablation for AF should be considered in patients with paroxysmal AF with prolonged pauses at the end of AF episodes to avoid the implantation of a permanent pacemaker.	**IIa**	**C**
Catheter Ablation as First-Line Treatment		
Catheter ablation for AF should be considered as a first-line therapy in patients with symptomatic paroxysmal AF if chosen by the patient.	**I**	**B**
Catheter ablation for AF may be considered as a first-line therapy in patients with symptomatic persistent AF if chosen by the patient.	**IIa**	**B**
Catheter ablation for AF is recommended as a first-line therapy in patients with AF suspected of inducing tachycardiomyopathy (with HFrEF), regardless of symptoms.	**I**	**B**
Catheter ablation for AF should be considered as a first-line therapy in patients with HFrEF to improve mortality and reduce heart failure hospitalizations.	**IIa**	**A**

AF: atrial fibrillation; HFrEF: heart failure with reduced ejection fraction.

### 8.2.1. Clinical Studies on Atrial Fibrillation Ablation

Numerous randomized clinical studies have been conducted in different populations of patients with AF, with outcomes ranging from quality of life to AF recurrence to reduced mortality, especially in patients with HF. Some have tested different technologies.

In patients with paroxysmal AF, the recurrence-free rate of antral pulmonary vein isolation ranges from 60% to 79%,^349,367,368^ reaching 77% in long-term follow-up.^369^ However, ablation of persistent AF has had less favorable results. Although additional modifications of the arrhythmogenic substrate and pulmonary vein isolation have been recommended, they have not had a major impact on the clinical benefits of this therapy.^370,371^ In fact, the long-term results in ablation of persistent AF have been inferior to those of paroxysmal AF, and further studies are needed to clarify the involved arrhythmogenic mechanisms. It should also be pointed out that many patients require additional ablation procedures, and late recurrence is not uncommon, particularly in persistent AF.^372,373^

Earlier clinical approaches have been investigated to prevent the progression of atrial cardiomyopathy, resulting in better sinus rhythm maintenance. In fact, the results of the EARLY-AF^374^ and EAST-AFNET 4^279^ clinical trials indicate that earlier intervention may lead to more favorable clinical outcomes. This may be due to containment and deceleration of pathological and deleterious LA remodeling. In a mean follow-up of 3 years, the ATTEST trial found that RF ablation was superior to AADs in delaying paroxysmal AF progression to its persistent form.^375^ Similarly, the EARLY-AF trial found the same advantage for cryoablation as an initial strategy in comparison to pharmacological therapy in patients with paroxysmal AF, and its results were reconfirmed in a recent publication with a mean clinical follow-up of 3 years.^375^ Furthermore, a systematic review and meta-analysis of observational studies found that a shorter period between AF diagnosis and ablation increases the chance of success.^376^ Finally, a meta-analysis by Han et al.^280^ found that an early rhythm control strategy may be more beneficial than rate control in patients with AF. Its 7 studies included 2 randomized clinical trials, 1 retrospective analysis of a randomized clinical trial, and 4 observational studies. The mean follow-up time ranged from 1 to 5 years. According to the pooled analysis, early rhythm control had lower all-cause mortality rate (RR 0.76; 95% CI 0.69–0.83; p < 0.00001), lower cardiovascular mortality (RR, 0.68; 95% CI 0.63–0.74; p < 0.00001), and lower risk of ischemic stroke (RR, 0.77; 95% CI 0.67–0.87; p < 0.0010; I^2^ = 64%).^280^ Therefore, current evidence strongly demonstrates that early rhythm control is superior to rate control. Hence, in patients with AF, even those whose clinical presentation is asymptomatic, the initial approach should aim to maintain sinus rhythm rather than rate alone.

Catheter ablation has also been widely studied as a first-line therapy for paroxysmal AF. A recent meta-analysis of 994 patients from 5 clinical trials comparing RF ablation (3 studies) or cryoablation (2 studies) with AADs confirmed its favorable results. Over a mean follow-up of 5 years, catheter ablation performed better than drug therapy for recurrent symptomatic AF (OR 0.32; 95% CI 0.18-0.57; p < 0.001) AFL (OR 0.36; 95% CI 0.25–0.52; p < 0.001), and hospitalization (OR 0.25; 95% CI 0.15-0.42; p < 0.001).^361^ Similar results were obtained in another meta-analysis of 6 clinical trials comparing RF ablation or cryoablation to AAD (1212 patients: 609 in the ablation arm, 603 in the AAD arm) with 1-2 years of clinical follow-up. According to the pooled analysis, catheter ablation (RF or cryoenergy) resulted in lower recurrence of atrial tachyarrhythmia (RR 0.63; 95% CI 0.55–0.73; p < 0.00001) and symptomatic atrial tachyarrhythmia (RR 0.53; 95% CI 0.32–0.87; p = 0.01), and no significant differences were found regarding adverse events (RR 0.93; 95% CI 0.68–1.27; p = 0.64) or adverse cardiovascular events (RR 0.90; 95% CI 0.56–1.44; p = 0.65).^377^

It has been shown that ablation leads to improved quality of life in both paroxysmal and persistent AF.^378^ The CABANA trial (2204 patients) attempted to demonstrate that ablation is superior to AADs regarding hard outcomes but, despite improved symptoms in the ablation group, the approaches did not differ significantly regarding a composite outcome of death, disabling stroke, severe bleeding, and cardiorespiratory arrest (HR 0.86; 95% CI 0.65-1.15). Nevertheless, this trial was hampered by a high crossover rate: 27.5% of the AAD group ended up undergoing ablation.^360,379^ Because there has been no robust evidence that ablation results in lower mortality or stroke rates in the general population since this trial, it is recommended only for symptom management.

The CASTLE-AF trial (363 patients with HFrEF [LVEF < 35%]) found that ablation was beneficial regarding a primary composite outcome of all-cause mortality or hospitalization due to worsening HF (HR 0.62; 95% CI 0.43-0.87) and a secondary outcome of only all-cause mortality (HR 0.53; 95% CI 0.32-0.86).^380^ CASTLE-AF was an important turning point for ablation as a first option in patients with HFrEF, especially when tachycardiomyopathy is suspected. Once AF is controlled, ventricular function is restored.

AF ablation may also benefit patients with paroxysmal AF related to sick sinus syndrome. In a retrospective analysis of patients recommended for pacemaker implantation due to prolonged pauses on AF termination, 43 who underwent ablation were compared to 57 who underwent permanent pacemaker implantation and AAD therapy. After a mean follow-up of 20 months, 95% of the ablation group no longer required pacemaker implantation.^381^ Thus, in addition to symptom control, these patients can benefit by avoiding pacemaker implantation.

Thus, ablation recommendations can be separated into paroxysmal AF and persistent AF (including persistent and long-standing persistent), since no data are available on periods > 12 months. In patients with paroxysmal and persistent AF, several studies have demonstrated that point-to-point RF ablation and cryoablation techniques have similar efficacy in maintaining sinus rhythm.^349,382,383^ In patients with AFL and atypical AFL associated with AF, point-to-point RF ablation is mandatory.

There is solid evidence of different success and complication rates among low-volume (< 50), medium-volume (50-100), and high-volume (> 100 procedures per year) AF ablation centers. High-volume centers have lower complication rates and higher success rates for maintained sinus rhythm, as well as lower mortality rates.^384^

### 8.3. Complications

Percutaneous ablation procedures for AF have now become routine in most electrophysiology laboratories, and mastery of these techniques is a requirement for new specialists. The rates of clinically relevant complications for this type of ablation are now very low. In a recent publication using data from a large multicenter U.S. registry involving approximately 76,000 patients treated with ablation between 2016 and 2020, the prevalence of any type of complication was 2.5%. Major complication and in-hospital mortality rates were 0.9% and 0.05%, respectively.^385^ Between 2000 and 2010, when AF ablation procedures gained credibility in various centers, the global prevalence of complications was > 5%, with in-hospital mortality of around 0.5%.^386-388^ Data from 37,000 AF ablations performed in Australia and New Zealand between 2008 and 2017 indicated a fivefold increase in the number of procedures, including treatment of increasingly complex cases and a 30% reduction in complication rates.^389^

The center's experience is an essential factor in AF ablation complications. A European registry of 3368 patients who underwent ablation at a total of 91 centers, classified the centers as high-volume (> 180 procedures per year), medium-volume (74-179 procedures) or low-volume (< 73 procedures). The rate of cardiovascular complications and stroke was higher in low-volume centers than high-volume (RR: 1.6; p = 0.039) and medium-volume centers (RR: 1.2; p = 0.008), and the success rate was lower in low-volume centers (p < 0.001).^390^ A meta-analysis of 14 studies (315,120 patients) found that two-thirds of the procedures were performed in low-volume centers. Centers with volumes > 50 and > 100 procedures per year had lower complication rates (OR: 0.58; p < 0.001 and OR: 0.62; p < 0.001, respectively) than low-volume centers (< 50 procedures per year). Both the mortality (OR: 0.33; p < 0.001) and complication (OR: 0.25; p < 0.001) rates were lower in centers that performed > 50 procedures per year.^391^

Attempts to make ablation more effective are also more aggressive, with new techniques increasing its extent in the left and right atria. This has changed the prevalence and type of complications. For example, LAA isolation, which some groups have proposed as an adjunctive technique in pulmonary vein isolation, adds an important mechanical variable to LAA contraction. This increases the risk of postoperative cardioembolic events and makes aggressive antithrombotic protection measures necessary, which range from systematic/permanent anticoagulant therapy to LAA closure through an occlusion device.^384^ Directing lesions to the posterior wall of the left atrium in a box lesion set increases the risk of thermal injury to the esophagus, requiring intraoperative and postoperative measures for prevention, early diagnosis, and treatment.^392^

Balloon catheter cryoablation of pulmonary vein antra was introduced in 2007,^393,394^ which simplified interventions by providing venous isolation in a single application. Many electrophysiology laboratories currently consider it the first therapeutic option, especially for paroxysmal AF. With effectiveness similar to RF ablation, it is a faster procedure, although it does not reduce complications. In a large meta-analysis of 15 controlled trials (approximately 2700 patients), no significant differences were found between RF ablation and balloon catheter cryoablation regarding complications.^395^ The main complications observed in percutaneous AF ablation procedures, diagnostic criteria, and preventive and therapeutic measures are summarized in Table 22.

**Table 22:** Percutaneous ablation procedures for atrial fibrillation: main complications and available techniques

Complication	Incidence	Diagnosis	Treatment	Prevention	Prognosis
**Thermal injury to the esophagus**	Up to 60%	Fortuitous (subclinical)	Soft/liquid diet	Reduce posterior wall power	Good
		Clinical manifestations	Proton pump inhibitor	Limit contact force on posterior wall	
		Upper digestive endoscopy	Sucralfate	Esophageal bypass	
				Ablation line modification	
**Atrioesophageal fistula**	0.01-0.03%	Clinical manifestations	Esophageal stent	Same as thermal injury	Poor
		CT	Surgery		Mortality > 50%
		MRI			
**Changes in gastroesophageal motility**	Up to 74%	Clinical manifestations	Low-volume soft/liquid diet	Same as thermal injury	Good
		Upper digestive endoscopy	Metoclopramide		
		CT	Nutritional support in severe forms		
			Surgery		
**Cardiac tamponade**	< 1%	Clinical-hemodynamic manifestations	Subxiphoid puncture drainage	Intracardiac echocardiography support	Good
		Fluoroscopy	Surgery		
		Intracardiac ECG			
		Transthoracic ECG			
**Ischemic stroke**	< 1% (clinical form)	Clinical manifestations	Thrombolytic therapy	Search for thrombi by imaging	Dependent on extent
		CT	Angioplasty	Uninterrupted anticoagulation	
		MRI		Early operative anticoagulation with heparin	
		Cerebral angiography		Careful aspiration and washing of sheaths	
**Air embolism**	< 1%	Suspected by intraoperative imaging	Respiratory and hemodynamic support measures	Careful, well-lit handling of sheaths and catheters	Dependent on extent
		Clinical manifestations	Head-down tilt position		
		Cerebral CT	Hyperbaric oxygenation		
		Cerebral MRI			
**Pulmonary vein stenosis**	< 1% (clinical form)	Clinical manifestations	Angioplasty	Avoid injuries inside the pulmonary veins	Good
		CT	Stent		
		MRI			
**Permanent phrenic paralysis**	< 1%	Radiological observation	Supportive measures	Phrenic nerve stimulation	Dependent on the magnitude of ventilatory involvement
		Clinical manifestations		Monitor electrical activity of the phrenic nerve	
Vascular complication	0.2-1.5%	Clinical manifestations	Conservative	Adequate vascular access technique	Good
		Vascular ultrasound	Embolization	Ultrasound-guided puncture	
			Surgery		

CT: Computed tomography; ECG: echocardiography; MRI: Magnetic resonance imaging.

### 8.4. Main Complications

#### 8.4.1. Thermal Injury to the Esophagus

Thermal injury to the esophagus is a frequent complication of percutaneous RF ablation or cryoablation procedures, observed in up to 60% of patients. Almost all of these injuries are transient, completely healing in the first few days after the procedure. In most cases, they are limited to erythema and superficial ulcers, but may also include deeper ulcers that heal more slowly.^396^ Ulcers appear to be less common in cryoablation than RF^397^ but, when they do occur, the major concern is that they are precursors to atrioesophageal fistula.^398^ These ulcers are usually asymptomatic and are discovered during postoperative digestive endoscopy, which is performed by some centers as a routine safety measure. There is no consensus on the effectiveness of preventive measures,^399^ such as reducing power and contact force in the posterior wall, monitoring esophageal temperature with a pre-molded multipolar thermometer (interrupting ablation in the event of elevated temperature), mechanical bypass using a transesophageal echocardiography probe or other device,^400-404^ modifying the dynamics of lesion production to avoid additional heating,^405^ line modification based on preoperative imaging-defined esophageal position.^406^ Some centers routinely perform upper gastrointestinal endoscopy in the first 24 to 48 hours after ablation.^407^ In these cases, the goal is early diagnosis, monitoring, and treatment to prevent progression to atrioesophageal fistula. Since early spontaneous resolution occurs in almost all cases, no consensus about this practice has been reached. Proton pump inhibitor therapy is routine in the postoperative period to reduce gastric hydrochloride secretion and, thus, avoid damage from gastroesophageal reflux.^319^ There is strong evidence that thermal injuries to the esophagus damage the anterior vagal plexus, leading to new reflux or exacerbating pre-existing cases. However, there is no consistent evidence that routine proton pump inhibitor therapy reduces the chance of atrioesophageal fistula. Treatment is intuitive for thermal injuries to the esophagus.^408^ A soft/liquid diet is usually prescribed to avoid additional further mechanical or thermal damage and, as mentioned above, proton pump inhibitor therapy is routine. When an ulcer is diagnosed, many centers use sucralfate (vials or chewable tablets) to cover the erosion with a protective film, which is a complex consisting of the drug and lesion exudate.

#### 8.4.2. Atrioesophageal or Esophago-Pericardial Fistula

Fistulas are the most feared complication in thermal lesion-based ablation procedures.^409^ However, they are rare, occurring in 1 of every 1000-4000 patients.^410^ This dramatic complication frequently leads to air embolism, mediastinitis, pericarditis, and septicemia. Mortality is high, with rates > 50% in patients treated surgically or endoscopically and around 90% in untreated patients.^411^ Manifestation is usually late, generally 2 to 3 weeks after ablation, and the most common symptoms are fever, general decline, chest pain, and systemic embolic events, particularly stroke due to septic embolism. Computed tomography or magnetic resonance imaging is required for diagnosis; upper digestive endoscopy should be avoided upon clinical suspicion due to the possibility of precipitating a gas embolism. Treatment should be immediate, since evidence indicates that conservative management is associated with worse outcomes. Although there is no consensus on whether surgery is superior to esophageal stenting, surgical treatment appears to have better results.^412^ A multidisciplinary team should be involved in decision-making. Surgical treatment should be shared by thoracic and cardiac surgery teams. Some endoscopic techniques for fistula closure have been used recently, especially when the fistula is not yet established.^413^

#### 8.4.3. Gastroesophageal Motility Changes

As previously mentioned, thermal ablation in the posterior wall of the LA can injure the anterior vagal plexus in the esophagus, resulting in changes to esophageal and gastric motility, including gastroesophageal reflux and gastroparesis. Such changes, which are relatively common and usually transient, often go unnoticed.^414,415^ Guided postoperative anamnesis often identifies symptoms, such as gastric fullness, nausea, and belching, which can be confused with common recovery symptoms. Severe gastroparesis is uncommon, but it can be limiting and difficult to treat.^416^ In milder symptomatic forms, an easily digestible liquid diet (administered in small volumes and at short intervals) is sufficient when associated with metoclopramide and bromopride. In severe forms, a multidisciplinary team and more aggressive treatment are necessary. Until normal peristalsis is reestablished, alternative nutritional support, a gastric pacemaker, or surgery may be required.

#### 8.4.4. Cardiac Tamponade

Myocardial perforation, with consequent hemopericardium and cardiac tamponade, remains one of the main complications of AF ablation. However, with increasing technical mastery of the transseptal puncture technique and LA instrumentation, in addition to the use of intracardiac echocardiography and catheters with contact force monitoring, the prevalence of this complication has declined substantially, currently to around 0.4%.^385^ It is diagnosed through reduced movement of the cardiac silhouette in fluoroscopy, direct demonstration of pericardial effusion by intracardiac echocardiography, or hemodynamic instability. If the electrophysiology laboratory does not have access to intracardiac echocardiography, transthoracic echocardiography is required. Treatment consists of percutaneous drainage by subxiphoid puncture and aspiration until negative pressure is reached in the pericardial sac, which almost always controls the situation.^417^ Heparin reversal with protamine sulfate should be performed after the pericardial effusion has been drained. The effects of DOACs may be inactivated with idarucizumab in patients treated with dabigatran or with andexanet-alfa in patients treated with factor Xa inhibitors.^418,419^ On rare occasions, the perforation must be repaired surgically due to persistent bleeding. Late cases of tamponade, which are also rare, can occur after AF ablation, and patients may present with nonspecific symptoms, including dyspnea and right HF.^420^

#### 8.4.5. Systemic Thromboembolic Events

The prevalence of systemic embolic events (strokes or systemic embolisms) is low: < 0.2% according to recent data.^385^ However, so-called asymptomatic cerebral embolisms are frequent and have been demonstrated in up to 87% of patients who undergo AF ablation.^421-423^ These microinfarcts generally regress a few weeks after detection and have not been associated with neurocognitive changes. Embolic events may be related to thrombus formation on transseptal puncture guidewires, inside or outside transseptal sheaths, or on catheter surfaces. They may also be related to crust formation on the tip of the ablation catheter during RF application or to fragmentation of a pre-existing thrombus in the LAA. Further possibilities include mechanical atrial stunning following electrical cardioversion during the procedure, thrombophilia, or a combination of factors. Because interrupting anticoagulant therapy with a low-molecular-weight heparin bridge appears related to a higher risk of embolic events, uninterrupted DOAC therapy is currently recommended, which does not increase the prevalence of hemorrhagic complications.^424^ Imaging of the LA or LAA through transesophageal echocardiography or cardiac computed tomography^425^ is suggested immediately before the procedure in patients who have been using anticoagulants for ≥ 3 weeks, especially in patients with CHA_2_DS_2_-VA scores ≥ 2. In those who are not using anticoagulants or who have been using them < 3 weeks, imaging is recommended within 48 hours of the procedure.^319^ For patients in sinus rhythm and/or with 3 weeks of effective anticoagulation, intracardiac echocardiography can be used to exclude thrombi at the time of the procedure.^334^

Systemic anticoagulation with heparin should begin immediately before or after transseptal puncture and must continue uninterrupted during the procedure for activated clotting time values of 300-350 seconds. Transseptal sheaths should be washed with heparinized saline solution after introduction into the LA. Although many centers use continuous irrigation with heparinized solution, there is no consensus recommendation. Catheters with open irrigation are always recommended in RF ablation to minimize crust formation at the tip. Treating thromboembolic events should involve interventional radiology teams, including members from neurology. Therefore, such procedures must be performed at centers with adequate multidisciplinary support. Clinically significant strokes should be promptly treated through neurovascular intervention, including thrombolytic therapy, aspiration, and angioplasty. Asymptomatic cerebral embolisms are diagnosed incidentally and are usually not treated.

#### 8.4.6. Air Embolism

Air embolisms occur during AF ablation procedures when air from the external environment enters the LA, especially through the transseptal sheath. Their clinical importance is based on the amount of air introduced into the system. A common sign of air embolism is ischemia and injury to the inferior wall of the left ventricle. The superior position of the right coronary ostium makes it a key entry point for bubbles. These changes are usually transient and may progress to total AV block, requiring temporary artificial cardiac pacing. Massive embolisms can lead to hemodynamic collapse, hypoxemia, and irreversible brain damage.^426^ Treatment involves maximizing oxygenation, hydration, treating shock and, when possible, aspirating the infused air.^427^ Downward head tilt is a beneficial maneuver, and hyperbaric oxygenation may reverse the condition. To prevent complications, bubbles must be carefully removed from sheaths, tubing, and catheters using light. Greater attention is required when using multiple irrigation routes and during balloon cryoablation.

#### 8.4.7. Pulmonary Vein Stenosis

Pulmonary vein stenosis is a complication of RF ablation^428^ and cryoablation.^429,430^ Severe, clinically relevant stenosis (diameter reduction > 70%) is now rare in pulmonary vein isolation procedures, with an incidence < 1%.^431^ In early RF ablations, when deeper isolation of the pulmonary vein ostia was performed, this complication was frequent. However, with more antral strategies, its occurrence has been drastically reduced.^318,431,432^ Severe stenosis may present with dyspnea, hemoptysis, cough, pneumonia, chest pain, and delayed onset, generally weeks after the procedure. Such symptoms should always arouse clinical suspicion, and they should be investigated with computed tomography angiography or magnetic resonance imaging.^433^ Treatment depends on the clinical significance and magnitude: angioplasty is the treatment of choice for severe forms, and stent implantation should always be considered, due to the lower chance of restenosis.^434^

#### 8.4.8. Phrenic Paralysis

Permanent phrenic paralysis now occurs almost exclusively in cryoablation procedures.^435,436^ It occurs due to the anatomical proximity of the right phrenic nerve to the right pulmonary vein antrum. The permanent form is rare, with an incidence of around 0.3%.^394,436^ This complication is normally avoided through phrenic stimulation during the procedure via a catheter positioned in the superior vena cava. Local stimulation > 10 mA is used, and its effectiveness is observed visually or by palpation of diaphragm movement with each pacemaker pulse. There must be no effects from neuromuscular blockers during cryoablation in the right veins. Movement loss or reduction indicates that the phrenic nerve is cooling and that the procedure must be immediately interrupted. There are techniques for directly monitoring the electrical activity of the phrenic nerve, but they are not commonly used due to their technical difficulty and the lack of significant advantages over indirect methods. Phrenic paralysis is usually a transient complication, completely resolving in the first 3 months after ablation. There is no specific treatment for permanent phrenic paralysis.

#### 8.4.9. Vascular Complications

Vascular complications are inherent to venous access punctures, and most electrophysiology laboratories exclusively use femoral venous access for these procedures. The main complications are inguinal hematoma, arteriovenous fistula, and femoral artery pseudoaneurysm.^318,387,437^ These complications are usually the result of inadvertent arterial punctures and inadequate hemostasis at the end of the procedure. Clinical manifestations include tumor formation in the puncture area and varying degrees of local pain. There should always be clinical suspicion of a major complication when there is significant postoperative pain, and vascular ultrasound should be performed when a vascular complication is suspected. Retroperitoneal hematoma is a more serious situation, resulting from higher puncture sites and pseudoaneurysms associated with accidental arterial punctures. Therapy requires interventional radiology and ranges from conservative management to embolization or surgical treatment. Ultrasound-guided puncture should be encouraged because it can reduce the incidence of this type of complication.^438^

#### 8.4.10. Other Complications

Some rarer postoperative complications of AF ablation have been described. Non-compliant LA syndrome, which was originally observed after mitral valve surgery, has been observed after RF ablation of AF.^438,440^ It is clinically characterized by HF due to increased LA filling pressures, accompanied by dyspnea and pulmonary arterial hypertension in the absence of LV dysfunction. It responds well to diuretic therapy and anti-inflammatory drugs. Cases of mild dyspnea and cough after ablation are not rare, and many centers routinely use diuretics in the first few days after ablation. Rare cases of Takotsubo syndrome have also been reported in patients undergoing percutaneous AF ablation: the estimated incidence is 0.04%.^441^

### 8.5. Cryoablation

Several randomized trials and observational studies have reported similar efficacy and safety for RF and cryoenergy ablations.^349,436,442-448^ Some studies have found lower hospitalization and complication rates with cryoablation.^449^ The main mechanism of cryoablation is cell lysis caused by ice formation in the intra- and extracellular environment, leading to necrosis, apoptosis, and a lower inflammatory response. This results in lower edema, a factor implicated in vein reconnection. Cryotherapy does not promote protein denaturation, preserving the collagen and elastin in connective tissue and, consequently, preserving the extracellular matrix, minimizing the risk of thrombus formation, vein stenosis, and esophageal injury.^450^

Important parameters of cryotherapy-related tissue damage include the cooling rate and duration, the rewarming time, and repetition of the cooling/heating/cooling cycle.^451^ Some parameters measured during the procedure, such as adequate contact of the balloon in the pulmonary vein antrum prior to cooling, recording the pulmonary vein potentials to determine the time from the start of cooling until the vein is electrically isolated (time-to-isolation [TTI]) and the tissue rewarming time, are important indicators of effective transmural lesion formation.^394,452^

#### 8.5.1. Cryoablation Techniques for Atrial Fibrillation

Contact between the cryoballoon and cardiac tissue is an important criterion for lesion formation and pulmonary vein isolation. Pulmonary vein occlusion is essential for successful cryoballoon usage. Adequate occlusion can be confirmed by fluoroscopy using contrast delivered through the balloon tip into the pulmonary vein or by observing leakage in intracardiac or transesophageal echocardiography.^453,454^

Pulmonary vein isolation can be monitored in real time through the cryoablation catheter. Several current protocols include TTI assessment during cryoablation.^455-457^ Studies have identified a greater tendency to recurrence in longer TTI.^35-39^ Isolation, when achieved within the first 60 seconds of cryotherapy, is associated with reduced pulmonary vein reconnection and with long-term maintenance,^451,458,459^ including a trend against reconnection when the TTI is < 40 seconds. Although TTI and dosage are closely associated with cryoablation efficacy, bonus freezing is appropriate when the TTI cannot be visualized or is ≥ 60 seconds. Isolation should be confirmed by testing whether the vein's inflow and outflow are obstructed.

Balloon temperature is an important parameter that must be monitored during the procedure. Attention should be paid to temperatures below −55 ºC for prolonged periods, and temperatures below −60 ºC should be avoided.^451^ The time it takes for the balloon to reach 0 °C after the end of cryotherapy should be measured to determine lesion quality and, consequently, the durability of vein isolation. In initial cryoablation studies, the applications lasted 240 to 300 seconds, followed by a bonus application.^460^ The application time has now been reduced to 180 seconds, and the bonus application has been eliminated, which has not affected the procedure's long-term effectiveness.^461,462^

### 8.6. Catheter Ablation of Atrial Fibrillation in Patients with Heart Failure

AF and HF frequently coexist: at least 50% of patients with HF have AF, and 30% of patients with AF develop HF. In patients with AF, elevated HR, loss of atrial systole, and temporal irregularity of the ventricular systole are pathophysiological changes that can induce ventricular dysfunction or aggravate pre-existing HFrEF. In parallel, the hemodynamic and hormonal changes involved in HFrEF create favorable conditions for sustained AF.^106,463^

Until recently, there was no clinical evidence that restoring and maintaining sinus rhythm was beneficial for this type of patient.^277,463^ However, between 2008 and 2014, 4 randomized trials (224 patients) found that ablation may have beneficial hemodynamic and clinical effects.^304,465-467^ In 3 of these trials, the patients had persistent AF (functional class II-III HF, mean EF 26%). The patients were mostly men (89%), aged 57-63 years, and ischemic heart disease was the predominant etiology of HFrEF. AF ablation significantly improved quality of life, oxygen consumption, and 6-minute walk test results compared to control treatment. In the persistent AF subgroup, LVEF increased significantly in patients who underwent AF ablation, and adverse effects did not significantly differ between groups.^465^

CAMERA-MRI was a multicenter randomized clinical trial involving patients with persistent AF and idiopathic cardiomyopathy (LVEF ≤ 45%). All patients underwent HR control and cardiac magnetic resonance imaging to assess LVEF and late gadolinium enhancement to determine the extent of fibrosis before randomization to catheter ablation or HR control therapy. Between 2013 and 2016, 66 patients were randomized, 33 to each arm. Absolute LVEF improved by 18 (SD, 13)% in the ablation group, compared to 4.4 (SD, 13)% in the HR control group (p < 0.0001). Normalized EF (≥ 50%) was 58% and 9%, respectively (p = 0.0002). In the catheter ablation group, the absence of late gadolinium enhancement predicted greater improvement in absolute LVEF and normalization at 6 months (73% vs. 29%; p = 0.0093).^468^

AATAC was the first multicenter randomized trial to indicate that ablation may reduce mortality. A total of 203 patients with persistent AF and HFrEF (NYHA II to III, LVEF < 40%) with dual-chamber implantable cardioverter defibrillators or cardiac resynchronization therapy defibrillators, were included, being randomized to catheter ablation (n = 102) or amiodarone (n = 101). During a minimum follow-up of 24 months, 70% of the ablation group was recurrence free, compared to 34% in the amiodarone group (p < 0.001). The hospitalization rate was 31% in the ablation group and 57% in the amiodarone group (p < 0.001). Importantly, mortality (the trial's secondary endpoint) was significantly lower in the ablation group (8% vs. 18%, p = 0.03).^469^

The CASTLE-AF trial^380^ confirmed the findings of the AATAC trial, demonstrating that, compared to rate control, catheter ablation of AF significantly reduces mortality in patients with HFrEF. In this trial, 263 patients with symptomatic paroxysmal or persistent AF were randomized, 179 patients to AF catheter ablation and 183 to clinical treatment, with rate or rhythm control. All patients had an implanted defibrillator and were NYHA class II, III, or IV, with LVEF ≤ 35%. After a mean follow-up of 37.8 months, the primary outcome, a composite of all-cause mortality and hospitalization for worsening HF, favored catheter ablation over clinical treatment. In the ablation group, 63% of patients were in sinus rhythm at 60 months vs 22% in the clinical treatment group. The primary composite outcome occurred in 51 (28.5%) patients in the ablation group and in 82 (44.6%) patients in the clinical treatment group (HR = 0.62; p = 0.007). There were significant reductions in all-cause mortality (13.4% vs. 25.0%; HR = 0.53, p = 0.01), and cardiovascular mortality (11.2% vs. 22.3%; HR = 0.49, p = 0.009) in the ablation group. Furthermore, the catheter ablation group had a lower hospitalization rate for worsening HF (20.7%) than the clinical treatment group (35.9%; HR = 0.56, p = 0.004). Catheter ablation also reduced AF burden, improving 6-minute walk test results and LVEF (8%).^380^ The main limitation of this study was its high selectivity, representing only 10% of the evaluated patients.

An attempt to generalize the results of CASTLE-AF to the real world found that < 10% of the general population had characteristics similar to those of the trial and that the risk reduction for mortality and hospital admission in ablation was lower than previously observed (RR 18% vs 40% in CASTLE-AF).^470^

The CASTLE-HTx trial assigned patients with terminal HF (mean EF 29 [SD, 6]%) to ablation (mean EF 25 [SD, 6]%) or drug therapy. In median follow-up of 18 months, occurrence of the primary composite endpoint (all-cause mortality, implantation of a left ventricular assist device, or urgent heart transplant) was lower in the ablation group (8% vs 30% in the drug therapy group). EF increased 7.8 (SD, 7.6)% in the ablation group, and the AF burden was reduced by 31.4 (SD, 33.3)%.^471^

The AMICA trial included 140 patients with persistent/long-standing AF and LVEF ≤ 35% (65 [SD, 8] years, 90% men) who were randomly allocated to catheter ablation or best medical therapy. Although the ablation group remained in sinus rhythm longer than the medical therapy group, there was no significant difference in the primary outcome, absolute increase in LVEF at 1 year (8.8% vs 7.3% in the ablation and medical therapy groups, respectively). There were also no significant differences in secondary outcomes, which included 6-minute walk test results, quality of life, and N-terminal pro-type B-natriuretic peptide levels.^472^ Of note, AMICA's sample apparently had more advanced HF than CASTLE-AF, since their mean ventricular function was lower (27% vs. 32%), more patients were NYHA class III-IV (100% vs. 70%), and more had persistent or long-standing persistent AF (60% vs. 31%). Nevertheless, LA size did not significantly differ between the populations. These data could suggest that the hemodynamic effects of restored sinus rhythm may be less evident in patients with more advanced HFrEF. However, at 1 year of follow-up, LVEF increase did not differ between the AMICA (8.8%), CASTLE-AF (7.0%), and AATAC (9.6%) trials. The lack of difference between ablation and medical therapy in AMICA was actually due to better EF recovery in the clinical group (7.3%) than in the CASTLE-AF or AATAC trials (2.0% and 4.2%, respectively), which might be explained by the greater number of patients with resynchronization devices in the AMICA study. Interestingly, primary outcome subgroup analyses in CASTLE-AF suggested that ablation benefitted patients in NYHA II with EF > 25%, but not in those in NYHA III with EF < 25%. The most important factor in sinus rhythm restoration and maintenance in patients with HFrEF, whether achieved through medical therapy or ablation, may be the degree of AF-induced tachycardiomyopathy.

There is a greater potential for complications during and after ablation in patients with HF, and they should be treated in hospitals with a medium or high volume of ablation procedures. A heart team, including a physician specialized in HF, is recommended. Recommendations on AF ablation in patients with HF are presented in Box 16.

**Box 16:** Recommendations on atrial fibrillation ablation in patients with heart failure

Recommendations	Class of recommendation	Level of evidence
Patients with HF being evaluated for AF ablation should be assigned a heart team, including a physician experienced in HF.	**I**	**B**
AF ablation is recommended for patients with HFrEF and a high probability of AF-induced tachycardiomyopathy.	**I**	**B**
In patients with HFrEF and AF, AF ablation is recommended due to shorter hospital stay and lower mortality.	**IIa**	**B**
AF ablation is recommended for patients with AF, controlled HR (resting HR < 80 bpm), and NYHA class IV or EF < 20%.	**IIb**	**B**

AF: atrial fibrillation; HF: Heart failure; HFrEF: heart failure with reduced ejection fraction; NYHA: New York Heart Association.

## 9. Surgical Treatment for Atrial Fibrillation

The Cox-maze procedure, first described in 1987, was the only form of invasive AF treatment for a number of years.^473^ To interrupt the atrial macro-reentry circuits, a cut-suture technique was used to create transmural scars and form conduction barriers in the atria, thus interrupting AF. Associating LAA resection further reduced thromboembolic events.^473-475^

The third version (Cox-maze III) became the standard surgical treatment for AF, maintaining long-term sinus rhythm in 73% to 97% of patients.^476^ However, due to its complexity, including the need for thoracotomy, and its considerable rate of complications and related morbidity, it has been limited to patients whose AF is refractory to clinical treatment and who were indicated for thoracotomy for other cardiac surgeries (eg, for mitral valve disease).^477^

Due to new invasive tools using energy sources such as cryothermy and RF, the traditional cut-suture technique has been replaced with catheter ablation, which, along with bipolar RF ablation forceps, has greatly advanced AF treatment.^478^ Access is now less invasive, which dispenses with extracorporeal circulation, and linear transmural lesions through the epicardium have made catheter ablation the gold standard surgical treatment for AF.^479^

In 2002, Gaynor et al. significantly expanded surgical treatment for AF worldwide with the Cox-maze IV procedure, which was a combination of bipolar RF and cryoenergy that replicated Cox-maze III incisions.^480^ This minimally invasive technique, which uses bipolar RF clamps, has made the procedure, which was formerly restricted to patients who required additional cardiac surgery, feasible for patients with AF alone. The records of the Society of Thoracic Surgeons clearly demonstrate this technique's expansion, increasing from 3987 procedures in 2004 to 12,737 in 2004.^481^ The advancements in surgical techniques are presented in Table 23.

**Table 23:** Modifications of the Cox-Maze Technique

Procedure	Modification from the Previous Version	Procedure Limitations
**Cox-Maze I**	NA	Inability to produce appropriate sinus tachycardia
		Postoperative left atrial dysfunction
**Cox-Maze II**	Left Atrium: Transverse atriotomy across the dome of the left atrium moved posteriorly	Prolonged intra-atrial conduction
		Complete transection of the SVC required for left atrial exposure
	Right Atrium: Elimination of the SVC for right atrial lesion	
**Cox-Maze III**	Right Atrium: Placement of the septal incision posterior to the SVC orifice	Prolonged ECC time and technical difficulty
**Cox-Maze IV**	Combination of bipolar RF ablation and cryoablation	Need for ECC
	Left Atrium: Box lesion around the posterior left atrium	

VECC: extracorporeal circulation; RF: radiofrequency; SVC: superior vena cava.

However, technical limitations have persisted in critical regions, making hybrid ablation necessary in certain situations. This technique combines minimally invasive surgery and catheter ablation, either simultaneously or sequentially, which allows surgically ablated areas to be tested for isolation, in addition to ablation of regions unreachable through surgery.^482^

The Cox-Maze (CM) IV is currently the gold standard surgical treatment for AF,^483-487^ with a success rate of 93%. It also has the advantage of requiring a shorter aortic cross-clamp time—47±26 minutes for CM IV compared to 93±34 minutes for CM III. A recent meta-analysis demonstrated that isolated CM IV performed via minithoracotomy is as effective as the sternotomy approach, with shorter hospital stays. The authors emphasize that a minimally invasive approach should be the procedure of choice whenever feasible.^488^ The CM IV procedure, when combined with other concomitant procedures, has also shown excellent outcomes, including improved quality of life, increased long-term survival, and reduced long-term risk of stroke.^486,489^

Randomized trials and meta-analyses have reported that hybrid procedures are superior to catheter ablation alone for maintaining sinus rhythm in patients with persistent and long-standing persistent AF, despite their higher complication rate.^476-478^ Resection or isolation (suturing or clipping) of the LAA to prevent thromboembolic phenomena is often associated with these techniques but is sometimes performed in isolation. The CM IV procedure, when combined with other concomitant procedures, has also demonstrated excellent results, such as improved quality of life, increased long-term survival, and a reduced long-term risk of stroke.^486,489^

### 9.1. Recommendations on Surgical Treatment for Atrial Fibrillation

#### 9.1.1. Surgical Treatment for Atrial Fibrillation Concomitant with Other Cardiac Surgeries

The majority of published results are for combined surgeries due to mitral valve disease (replacement or repair), although myocardial revascularization is also possible.^492,493^ A 2022 meta-analysis of 3 randomized trials with concomitant mitral valve surgery found that the Cox-maze procedure (III or IV) resulted in greater AF-free survival than pulmonary vein isolation alone.^494^ The benefits of combining the Cox-maze technique with mitral valve treatment are evident in improved mortality rates and quality of life.

However, safety issues (morbidity and mortality) still require further refinement and remain major limitations. According to recent systematic reviews and meta-analyses, surgical ablation associated with mitral valve treatment resulted in a lower 1-year AF recurrence rate without increasing 30-day mortality or major cardiovascular events.^495,496^

#### 9.1.2. Isolated Atrial Fibrillation Surgery

Isolated surgical treatment of AF is intended for patients with arrhythmia but not structural heart disease. Secondary objectives may include LAA occlusion and RF ablation using thoracoscopy for access. A meta-analysis of 3 randomized controlled trials and 2 retrospective cohort studies (587 patients) found that thoracoscopic ablation was superior to catheter ablation. In the randomized controlled trials, the proportion of patients without atrial arrhythmia or AAD therapy in 1 year of follow-up was significantly higher in the thoracoscopic ablation group.^497^

The FAST trial randomized patients with a higher chance catheter ablation failure to either epicardial thoracoscopic AF ablation or endocardial catheter ablation, finding lower recurrence after thoracoscopic ablation than catheter ablation (56% vs. 87%, respectively) in long-term follow-up (mean 7 years). However, the median length of hospital stay was longer and complication rates were higher for thoracoscopy than catheter ablation.^498^

Thoracoscopic ablation is more effective for rhythm control than catheter ablation, but it is a more invasive procedure and involves higher complication rates and longer hospital stays, making it more suitable for patients with prior failed catheter ablation or a high risk of failure. Recommendations on surgical ablation are shown in Box 17.

**Box 17:** Recommendations on surgical and hybrid ablation

Recommendations	Class of recommendation	Level of evidence
Patients with symptomatic, recurrent AF refractory to at least one antiarrhythmic drug undergoing mitral valve surgery: Concomitant surgical ablation of AF should be considered. The experience of the surgical team and the benefits of maintaining sinus rhythm should be weighed against the related surgical risks.	I	B
Patients with symptomatic, recurrent AF refractory to at least one antiarrhythmic drug undergoing surgery not involving the mitral valve: Concomitant surgical ablation of AF may be considered. The surgical team's experience and the benefits of maintaining sinus rhythm should be weighed against the related surgical risks.	IIa	B
Hybrid ablation should be considered for patients with symptomatic, paroxysmal, or persistent AF refractory to antiarrhythmic drugs and isolated endocardial ablation, with joint decision-making between the electrophysiologist and cardiac surgeon.	IIb	B

### 9.2. Long-Term Results and Complications

For decades, surgical treatment of AF has been an effective option for long-term sinus rhythm maintenance. The Cox-maze procedure has a remarkable AF-free success rate and low prevalence of late stroke.^473,474,499^ Patients undergoing the Cox-maze procedure have better survival than those with untreated AF.^500^ Cox-maze IV is superior to catheter ablation or surgical ablation, particularly in patients with persistent or long-standing persistent AF.^486^ A systematic review compared medium-term clinical results between patients who underwent the Cox-maze procedure and those who underwent pulmonary vein isolation concomitantly with mitral valve replacement, finding lower 1-year recurrence of AF in the Cox-maze group.^494^

The long-term results of the Cox-maze procedure are also excellent. In 10 years of follow-up, Weimar et al. reported that 85% of patients were without symptomatic AF.^501^ Only 1 late stroke was reported, and 80% of the patients did not require anticoagulant therapy during follow-up.^501^ Similar results were reported by Khiabani et al., with AF-free rates at 1, 5, and 10 years of 92%, 84%, and 77%, respectively.^483^

The Cox-maze IV procedure alone also has good results in patients with persistent AF: 7 years after the procedure, the morbidity rate was low and 88% did not have symptomatic AF.^485^

The Cox-maze procedure is equally effective when performed through a right minithoracotomy or a sternotomy.^502,503^ This approach is associated with fewer complications, lower mortality rates, and shorter intensive care unit and hospital stays.^502^ The main complications associated with Cox-maze IV are higher rates of pacemaker implantation and acute renal injury. The rate of pacemaker implantation ranges from 21.5% to 6.3%.^484,492^ The highest incidence of renal injury occurs in patients undergoing concomitant Cox-maze IV (32% vs 16%), due to the longer extracorporeal circulation time.^504^

To date, no robust study has verified specific adverse events associated with the Cox-maze procedure. Factors such as technical variability, association or not with another surgical procedure, advanced patient age, and comorbidities make it difficult to analyze complications directly related to the procedure.

### 9.3. Hybrid Procedures

Hybrid procedures for AF ablation, which were introduced in 2009,^505^ consist of an initial epicardial surgical approach (uni-/bilateral thoracoscopy or laparoscopy through the subxiphoid region) that can encompass the LA posterior wall, autonomic plexuses, Marshall's ligament or LAA occlusion. Transmurality can be confirmed and complemented with electroanatomical mapping and endocardial ablation, performed concomitantly or a few months after the initial procedure.

CONVERGE was the first randomized trial to include a large number of patients with long-standing persistent AF (mean duration = 4.4 months). The 153 patients were randomized in a 2:1 ratio (102 to hybrid ablation, 51 to endocardial catheter ablation), and hybrid ablation was more effective (67% vs 50% p = 0.03) in 1 year of follow-up.^490^ A meta-analysis of 16 studies (only 1 randomized; 1242 patients) found no AF recurrence in 78%, 75%, and 73% of the patients after 1, 2, and 3 years of outpatient follow-up, respectively.^506^

The decision to perform these procedures should be made by a multidisciplinary heart team led by a cardiac surgeon and an electrophysiologist, who care for patients undergoing this type of intervention.^507^ Further technological development and clinical trials are needed to verify the cost-effectiveness, AF burden, quality of life, and ventricular function improvement of patients undergoing hybrid procedures, since these parameters are frequently compromised in patients with persistent AF. Technical variations must be studied, such as the concomitant use of epicardial and endocardial approaches, LAA occlusion, and endocardial strategies that can significantly influence the procedure's effectiveness and safety.

## 10. Multidisciplinary Approaches to Atrial Fibrillation

### 10.1. Implementing Lifestyle Change Programs

Hendriks et al. found that patients treated in multidisciplinary clinics dedicated to AF treatment (led by nurses and supervised by cardiologists) had a 35% lower combined outcome of hospital admission and cardiovascular mortality than patients who underwent conventional treatment with a cardiologist.^508^ Integrated treatment involving health care professionals, patients, and family members can improve clinical outcomes and treatment adherence.^509,510^

Younis et al. investigated the role of specialized rehabilitation programs in 292 patients with AF between 2009 and 2015 for a primary composite outcome of cardiovascular hospitalization and overall mortality, finding that patients with better cardiopulmonary performance had significantly better outcomes (RR 0.40; p = 0.001). There was a significant reduction in events among patients with improved cardiopulmonary performance (RR 0.83; p = 0.04).^511^

Since AF substrate involves multiple comorbidities and different clinical scenarios, a multidisciplinary approach involving general practitioners, cardiologists, arrhythmologists, endocrinologists, sleep clinics, cardiac rehabilitation clinics, nurses, physical educators, and physical therapists is recommended to improve clinical outcomes, reduce AF recurrence, and prevent progression to persistent forms of arrhythmia.

## 11. Special Situations in Atrial Fibrillation

### 11.1. Intracranial Hemorrhage

Intracranial hemorrhage is one of the most delicate situations in AF treatment. Due to its potential lethality in patients treated with OAC or antiplatelet agents, restarting anticoagulation is avoided in patients with intracranial hemorrhage, although some are at high risk for ischemic stroke.^512^ The decision to restart anticoagulation in these patients should be made by a multidisciplinary team that includes a cardiologist, a neurologist, a neurosurgeon, and a hematologist and should also involve the patient, family members, and/or guardians. After an intracranial hemorrhage occurs, the decision to initiate anticoagulation is not simple, since these patients have been excluded from all stroke prevention studies in patients with AF. Careful analysis of the case, including risk and benefit assessment based on brain imaging results, will help the team reach a decision.^221^

Risk factors for recurrent intracranial hemorrhage should be evaluated and divided into 2 groups: modifiable and non-modifiable (Table 24). When most risk factors are modifiable and no other factors contraindicate anticoagulation, and the risk/benefit ratio is considered favorable, anticoagulation can be restarted (Figure 19). The ideal time to restart anticoagulant therapy is not clear, but it should occur after the acute phase, generally a minimum of 4 weeks. Although no studies have compared DOACs and warfarin in patients with intracranial hemorrhage, DOACs are preferred because randomized clinical trials have found that they reduce intracranial hemorrhage by approximately 50%.^153^

**Table 24:** Risk factors for intracranial hemorrhage recurrence

Modifiable Factors	Non-Modifiable Factors
Uncontrolled hypertension	Advanced Age
Low LDL and triglyceride levels	Male Sex
Excessive alcohol consumption	Asian Ethnicity
Oral anticoagulant therapy	Chronic Renal Failure
Sympathomimetic drug therapy*	Cerebral Amyloid Angiopathy
	Small vessel disease of the brain

* Cocaine, Heroin, Amphetamine, Ephedrine. LDL: low-density lipoprotein.

In patients with non-modifiable risk factors or risk factors that contraindicate anticoagulation due to a high risk of recurrence, LAA occlusion is an option,^512^ although no specific studies have been conducted on this topic. Figure 20 shows the factors to be considered when restarting anticoagulation after intracranial hemorrhage.

**Figure 20:** Restarting anticoagulation after intracranial hemorrhage. Figure adapted from the 2021 European Heart Rhythm Association Practical Guide on the use of DOACs(2). DOAC: Direct-acting oral anticoagulant; LAA: left atrial appendage. a: No evidence from randomized clinical trials; b: Imaging study is mandatory before restarting DOAC.

### 11.2. The Oldest Old Population

The diagnosis of atrial fibrillation (AF) in elderly patients can be challenging. In this population, AF may be asymptomatic or present with atypical symptoms, potentially delaying diagnosis. Moreover, the coexistence of multiple comorbidities and their respective pharmacological treatments can mask AF symptoms or complicate the diagnostic process. The difficulty in distinguishing AF from other cardiac arrhythmias, such as atrial tachycardia (AT), also poses a challenge in caring for the geriatric population.

Changes that occur with age in atrial structure, contractility, and electrophysiology may predispose individuals to AF. HFpEF, which is common in this age group, may also provoke rhythm abnormality. In older adults, whether symptomatic or oligosymptomatic, frequent atrial extrasystoles and AFL are indicative of greater electrical instability and are a marker of atrial cardiomyopathy, which suggests a greater risk of AF. The Copenhagen Holter study, which used 48-hour Holter monitoring to assess 678 individuals without known cardiovascular disease or previous AF, found a strong association between ischemic stroke and atrial extrasystoles ≥ 30 premature atrial contractions per hour and/or any runs of ≥ 20 premature atrial contractions.^513^

The association between excessive supraventricular ectopic activity and subclinical cerebrovascular disease was also investigated in a cross-sectional study of 462 men (mean age, 68.1 years) with neither apparent stroke nor AF who underwent 24-hour Holter electrocardiography and brain magnetic resonance imaging. Excessive supraventricular ectopic activity was independently associated with white matter hyperintensity and intracranial atherosclerotic stenosis, which are both strong predictors of ischemic stroke.^514^

### 11.3. Minority Groups

Although the literature recognizes the existence of racial differences in cardiovascular disease and stroke, studies on the clinical epidemiology of AF in non-White populations is scarce. Sarraju et al. reviewed the studies included in the American Heart Association's AF Guidelines, finding that less than half included any racial data.^515^ In a review of 134 randomized clinical trials, Khan et al. found that only 12.7% included any information on race.^516^

The prevalence of AF in Asia is around 1%, which is lower than in the West. However, due to the size of this population, it is estimated that AF will affect around 60-70 million Asians by 2050. The true prevalence of AF in Africa is unknown, although small studies have found prevalences from 0.7% to 5.5%, and some believe that by 2050 Africa will have the highest AF prevalence in the world.^517-519^

Epidemiological studies have shown that AF is more prevalent among Whites than other races. In a recent study of the National Inpatient Sample database (330,000,000 hospital discharges between 2003 and 2013), the prevalence of AF was significantly higher among White than Black people (11.3% vs 4.6%, p < 0.001). This difference was even more significant in patients > 75 years of age (31.4% vs 18.2%, respectively, p < 0.001).^520^ The fact that the prevalence of AF is lower among Blacks than Whites, despite a higher prevalence of traditional risk factors, is called the "AF paradox".^35,521-523^ This paradox is also relevant among South Asians, who have higher age-adjusted rates of diabetes, hypertension, and coronary heart disease.^524^

Studies conducted over the last 15 years have shown that people of European descent have 9 genetic loci associated with AF risk.^525^ Black and Hispanic patients with early-onset AF have a higher chance of a first-degree relative with AF than White patients, suggesting a genetic predisposition, which should be further explored.^526^

AF symptoms also differ according to race. According to the European Arrhythmia Association, Black patients report more severe and debilitating symptoms (20%) than Whites (16.4%) or Hispanics (8.5%). Furthermore, quality of life among Black patients is worse after 2 years of follow-up.^527^

Furthermore, the clinical course of AF differs according to race. A study of more than 500,000 U.S. Medicare patients with AF aged > 65 years found that Blacks and Hispanics had a higher risk of mortality (46% and 11%, respectively) and stroke (66% and 21%) than Whites.^528^ A subanalysis of the ROCKET AF trial, which tested rivaroxaban, found that the incidence of ischemic and hemorrhagic stroke was higher among non-Hispanic Black and Asian patients.^529^ The multinational ARISTOTLE trial, which used apixaban as an anticoagulant, found that some Asian patients were at higher risk of both ischemic and hemorrhagic stroke.^517^

Therefore, racial differences affect treatment results, even after adjusting for socioeconomic factors. The challenge is to tailor new health coverage plans and clinical workflows to increase access and quality of care for racial minorities.^530^

### 11.4. Kidney Disease

Chronic kidney disease, which occurs in > 40% of patients with AF, increases mortality.^531,532^ However, AF may accelerate its progression.^532^ CrCl is the best independent predictor of complications in patients with kidney disease and AF, whether due to embolic complications or to hemorrhage.^532^

Because kidney disease is a prothrombotic and prohemorrhagic condition, the main randomized trials on cerebral embolism prevention through DOACs corrected the dosage in patients with CrCl < 50%.^149-152^ Studies found that DOACs have similar efficacy and safety in patients with and without mild-to-moderate renal impairment.^533-536^ Thus, in patients with renal dysfunction, DOAC therapy should take into account the risk factors described in Table 24. For patients with CrCl < 15 ml/min or who are on dialysis, observational studies question the benefit of OAC.^17^ Although DOACs have been found to result in lower bleeding than warfarin,^537^ a meta-analysis found that OAC does not reduce ischemic stroke or systemic embolism in these patients.^538^

RENAL-AF, a multicenter randomized trial of apixaban and warfarin in 154 chronic kidney disease patients on dialysis, was interrupted prematurely due to difficulty enrolling patients. Thus, it did not have sufficient statistical power to conclude that the drugs differed regarding bleeding in this population.^539^ However, there were 10 times more relevant bleeding events than ischemic events, whether due to ischemic stroke or systemic embolism. The AXADIA-AFNET 8 trial randomized 97 patients with AF, chronic kidney disease, and long-term hemodialysis to demonstrate the non-inferiority of apixaban in relation to phenprocoumon, a VKA, for efficacy and safety. No significant differences were found between the drugs, although, patients remained at high risk of cardiovascular events, including high mortality and bleeding.^540^ Therefore, it remains unclear whether OAC should be used in patients with AF and chronic kidney disease who are undergoing renal replacement therapy.

### 11.5. Atrial Fibrillation in Pregnancy

Cardiovascular diseases are the main cause of maternal mortality, with arrhythmias being the main cardiac complication observed during pregnancy.^541^ In pregnant women, the incidence of AF is relatively low and is related to pre-existing heart conditions, such as congenital heart disease, cardiomyopathies, and mitral valve disease, which is highly prevalent in Brazil, especially of rheumatic etiology. In pregnant women without structural heart disease, obesity and advanced age are the main risk factors for arrhythmia. The incidence of AF is approximately 5 times higher in women who become pregnant after 35 years of age than in those who do so before 25 years of age.^542^

The clinical impact of AF in pregnant women is influenced by physiological cardiovascular changes, including a significant increase in plasma volume and cardiac output beginning in the second trimester, as well as in HR, especially in the third trimester, an effect closely related to increased adrenergic tone. Increased blood volume, associated with a hyperadrenergic state, stresses the atrial wall, causing increased automatic activity and triggering activity in atrial myocytes. Due to the high hemodynamic demand, pregnant women are less tolerant of AF, and it may be a factor in decompensation of pre-existing heart disease. Through mechanisms that are not clearly understood, AF is related to a higher risk of obstetric complications, such as pre-eclampsia, eclampsia, and premature delivery.^543^

In highly symptomatic pregnant women, the clinical response may be better to rhythm control. Electrical cardioversion can be performed safely in pregnant women, with minimal repercussions on the fetal heart, which should be appropriately monitored. Pharmacological antiarrhythmic treatment in asymptomatic and clinically stable patients should be avoided in the first trimester of pregnancy, especially between the fifth and tenth weeks, during organogenesis. Propafenone and sotalol have an acceptable safety profile for maintaining sinus rhythm beginning in the second trimester (U.S. Food and Drug Administration class C: adverse fetal effects in animal studies, no human studies, but the potential benefits of treatment justify the risk). Amiodarone should not be used due to the greater risk of significant adverse effects in the fetus.^544^ Catheter ablation can be performed in more serious situations, either avoiding or minimizing fetal radiological exposure or using non-fluoroscopic techniques.^545^

If rhythm control is not feasible, rate control with cardioselective beta-blockers (bisoprolol or metoprolol) or non-dihydropyridine calcium channel blockers (verapamil or diltiazem) should be used. Digoxin is considered a second-line drug in this context and can be used in cases of unsatisfactory symptom control, either alone or with the above-described agents, provided due caution is taken regarding drug interactions. Although adverse effects, such as reduced intrauterine growth and fetal hypoglycemia, are characteristic of beta-blockers, atenolol is associated with a higher risk of adverse reactions and is not recommended during pregnancy.^546,547^ Recommendations on rate and rhythm control in pregnant patients are shown in Box 18.

**Box 18:** Recommendations on rhythm control in pregnant women

Recommendations	Class of recommendation	Level of evidence
Anticoagulation with low molecular weight heparin (LMWH) or vitamin K antagonists (VKAs) (except during the first trimester and after the 36th week of gestation) is recommended for pregnant women at high thromboembolic risk.	**I**	**C**
Beta-blockers (except atenolol) are recommended for heart rate control during pregnancy to reduce symptoms and improve maternal and fetal outcomes.	**I**	**C**
Electrical cardioversion to restore sinus rhythm is recommended in the presence of hemodynamic instability or limiting symptoms, with mandatory fetal monitoring.	**I**	**C**
Digoxin is recommended for heart rate control during pregnancy to reduce symptoms and improve maternal and fetal outcomes in cases of intolerance or lack of response to beta-blockers.	**IIa**	**C**
Propafenone or sotalol may be used for maintaining sinus rhythm from the second trimester of pregnancy in cases where heart rate control has been unsuccessful.	**IIb**	**C**
Catheter ablation may be used in selected cases of refractory treatment and limiting symptoms, ensuring minimal or no fetal fluoroscopic exposure.	**IIb**	**C**
Direct oral anticoagulants (DOACs) are not recommended for anticoagulation in pregnant women.	**III**	**B**
Oral amiodarone is not recommended for rhythm control or heart rate control at any stage of pregnancy.	**III**	**B**

Although pregnancy causes a state of hypercoagulability, the role of anticoagulation in thromboembolic event prevention is not clear. Although the CHA_2_DS_2_-VA score has not been validated in pregnant women, it is accepted as a means of identifying patients at higher risk. In significant mitral valve disease, especially of rheumatic etiology, anticoagulation should be assessed in the first trimester, with low molecular weight heparin being the safest choice during this period. Warfarin can be administered with relative safety in the second and third trimesters, and should be replaced by low molecular weight heparin in the peripartum period. There is no consistent information about the effects of DOACs during pregnancy, and this class of drugs may even be associated with spontaneous abortion and birth defects.^548^

### 11.6. Atrial Fibrillation (AF), the Parasympathetic Nervous System, and Cardioneuroablation

The vagal innervation contributes to the induction and maintenance of atrial fibrillation (AF) by influencing the electrical activity of the atria. It reduces the refractory period of the atrial walls, increases the dispersion of refractoriness, and prolongs the calcium transient, thereby sustaining AF. Furthermore, it can influence the firing of pulmonary veins, potentially triggering AF.^549^ Vagal activity may also affect atrial remodeling, resulting in structural and electrical changes that contribute to AF.^550^ These mechanisms demonstrate the vagal influence on AF, which has been observed even in young individuals without significant heart disease. However, it is important to note that AF is a complex condition with multiple contributing factors, and vagal innervation is only one of several mechanisms involved.

Sharifov et al.^551^ reported that directly infusing acetylcholine into the sinus node artery induces AF in 100% of dogs. Acetylcholine-mediated AF is facilitated by isoproterenol, which decreases the threshold concentration of acetylcholine required to trigger AF and prolonged AF episodes. Isolated infusion of isoproterenol into the sinus node artery also causes AF. However, if the vagal effect has been previously blocked with atropine, isoproterenol no longer triggers AF, which shows that catecholamines alone, without the vagal effect, are poor inducers of AF.

In the 1990s, using spectral analysis, it was found that highly innervated areas of the atrial myocardium present ECG changes that can be detected by spectral analysis using the fast Fourier transform.^552,553^ At these sites, the insertion of neural fibers reduces the lateral electrical connection of myocardial cells, allowing micro-reentry and AF maintenance. Part of the vagal innervation enters the atria through the pulmonary veins, in whose antra a large amount of parasympathetic innervation can be found. Ablation of these sites in the left and right atrial walls causes intense vagal denervation and electrically stabilizes the atrial walls, making the atria more resistant to progressive pacing and, thus, to AF reinduction.

Cardioneuroablation is based on the mapping and ablation of these ganglionated plexuses. It is a technique that allows for vagal denervation through endocardial RF ablation on the atrial walls. In this way, cardioneuroablation can be combined with atrial fibrillation (AF) ablation, specifically pulmonary vein isolation, and may have applications in this population.

A meta-analysis of AF ablation combined with empirical anatomical cardioneuroablation showed better results in the paroxysmal group with structurally normal hearts treated with concomitant parasympathetic denervation along with pulmonary vein isolation. However, no benefit was found in the persistent AF group.^554^ Recently, some ablation techniques preserve vagal innervation, such as pulsed field ablation (PFA), meaning that part of the beneficial effect of this technique might be lost.^555^ Osório et al. demonstrated that cryoablation reduces but still preserves a significant vagal effect.^556^

### 11.7. Atrial Fibrillation in Athletes

Physical activity reduces the risk of cardiovascular disease, promotes healthy aging, and contributes to physical and emotional well-being. It also reduces the occurrence of AF and should be recommended for patients with AF.^72^ However, it has been shown male high-performance athletes have a higher risk of AF,^557^ suggesting that the AF and sports have a U-curve relationship.^558^ Running and cycling are associated with the highest risk.^75^

The mechanisms involved in the increased AF risk among professional athletes are complex and include: atrial dilation, adrenergic stimulation, changes in vagal tone, inflammation, fibrosis, and changes in electrophysiological properties.^559,560^ Several other mechanisms are still under investigation.

The most likely patients are taller middle-aged men who have been involved in the sport for a long time. Although no randomized trials directly support this recommendation, patients eligible for anticoagulation should avoid contact sports. Due to the frequent sinus bradycardia among athletes, drug treatment is difficult and catheter ablation is usually preferable.

### 11.8. Arrhythmic Syndromes

#### 11.8.1. Arrhythmic Syndrome in Young People without Apparent Structural Heart Disease

AF in young people without apparent structural heart disease is rare (prevalence < 1%), a condition previously called "isolated AF".^561^ This term is now obsolete due to evolving understanding of the disease's complexity and its underlying molecular mechanisms.

Currently, 2 family patterns have been observed for AF as a monogenic disease with Mendelian inheritance: (1) AF without structural heart disease, which is related to atrial channelopathies; (2) familial AF in the context of another hereditary cardiomyopathy, with incipient signs of ventricular heart disease.^562^ To date, more than 30 genes have been associated with AF, most of which encode ion channels (proteins involved in calcium metabolism), as well as genes associated with fibrosis, conduction disease, and inflammation, now better known as atrial cardiomyopathy. Despite increasing awareness of the genetic aspects of AF, genetic testing is rarely recommended and few definitively associated genes have been found so far.^563^

Patients with channelopathies, such as Brugada syndrome, have a higher prevalence of AF, and in some cohorts the presence of AF has been considered an additional risk factor for sudden death. In short QT syndrome, AF is a diagnostic criterion and is present in half the patients.^564^ Although rarer, AF can also be observed in patients with long QT syndrome and catecholaminergic polymorphic ventricular tachycardia.^565^

Recently, AF has been observed as an early manifestation of some inherited cardiomyopathies, even before hemodynamic overload occurs. This suggests that AF may be caused by a specific cardiomyopathy that primarily affects the atrium and that it may have a progressive character.^566^

In clinical practice, one important factor in recognizing genetically-based AF is antiarrhythmic therapy. In Brugada syndrome, class I drugs are contraindicated (www.brugadadrugs.org), which prohibits acute treatment (pill-in-the-pocket approach) with propafenone. In long QT syndrome, many drugs are contraindicated due to the increased risk of QT interval prolongation and torsades de pointes (http://www.crediblemeds.org/). Furthermore, ablation in young individuals with channelopathy or atrial cardiomyopathy may not be as effective as expected.

Thus, AF in young people, previously considered an easily controlled arrhythmia with few relevant prognostic implications, has undergone a paradigm shift due to the increased availability of genetic testing and the recognition of family patterns. Genetic diagnoses have impacted several aspects of clinical management, including family screening and counseling, exercise prescription, pharmacological therapy, and recommendations on cardioverter-defibrillators. Thus, it is reasonable to use genetic evaluation of atrial fibrillation (AF) in clinical practice in selected cases, especially in younger patients (<60 years), where there is suspicion of familial AF based on the patient's clinical history, family history, and ECG characteristics. The analyses should include the SCN5A, LMNA, KCNQ1, MYL4, and TTN genes.

### 11.9. Atrial Arrhythmias in Adult Patients with Congenital Heart Disease

Intra-atrial reentrant tachycardia, including typical and atypical AFL and AF, is the most common type of sustained tachyarrhythmia in adults with congenital heart disease. In a retrospective cohort of 482 patients with congenital heart disease, atrial tachycardias were found in 61.6%, and 28.8% had AF. AF prevalence increased with age, being more common in participants > 50 years of age (51.2% vs. 44.2%).^567^

The arrhythmogenic substrate of AFL involves atrial scarring and prosthetic material, and its risk factors include the complexity of congenital heart disease, atrial incision, and the number of previous cardiac surgeries. The prevalence of AFL also increases after Fontan surgery^568^ and, in patients with corrected tetralogy of Fallot, with severe tricuspid insufficiency.^569^ However, the arrhythmogenic substrate of AF also includes electrical and structural remodeling mechanisms, in addition to risk factors, such as age and arterial hypertension, which are associated with the occurrence of AF in adults with congenital heart disease.^567,570^ Uncorrected or late-corrected atrial septal defects have been associated with a higher incidence of AF,^571^ and surgical or percutaneous closure is related to a lower prevalence of AF.^572^

Despite the increased survival of adults with congenital heart disease, the available evidence for atrial arrhythmia treatment in this population is predominantly derived from observational studies or extrapolated from large clinical trials.

Anticoagulation should be indicated according to the complexity of the congenital heart disease. Anticoagulation is recommended for all patients with AFL or AF and congenital heart disease of moderate-to-severe complexity, including patients who have undergone intracardiac surgical repair, those with cyanotic heart disease, and those with systemic right ventricle or post-Fontan surgery. In patients with AFL or AF and low-complexity congenital heart disease, anticoagulation is indicated if there is ≥ 1 thromboembolic risk factor (CHA_2_DS_2_-VA score ≥ 1).^17,570,573^ However, because the CHA_2_DS_2_-VA and HAS-BLED scores have not been validated in this population, the application and interpretation of these scores are limited. DOACs appear to be safe and effective in adults with congenital heart disease without mechanical valve prosthesis or moderate-to-severe mitral stenosis.^574-576^

It is desirable to maintain sinus rhythm in patients with congenital heart disease. Thus, rhythm control is generally considered the initial therapeutic approach for AFL or AF in patients with moderate-to-severe congenital heart disease.^574,577^ Rate control remains potentially useful in patients with simple congenital heart disease or in more complex cases when rhythm control strategies fail.^577^ In patients with AF and an atrial septal defect, AF surgery, such as the Cox-maze procedure, or catheter ablation should be considered during the procedure to close the defect.

It should be pointed out that patients with congenital heart disease and AFL or AF frequently present sinus node dysfunction, increasing the risk of sinus arrest or sinus bradycardia after acute reversal of the arrhythmia. Thus, a temporary pacemaker must be available during attempted cardioversion.^570^

Class IC AADs, such as propafenone, should be used with caution due to the risk of slowing AFL, resulting in 1:1 atrioventricular conduction.^578^ Of note, myocardial fibrosis, ventricular dysfunction, or hypertrophy is common in adults with congenital heart disease. Hence, IC class antiarrhythmics should be avoided in patients with systemic ventricular dysfunction or hypertrophy.

Amiodarone may be considered for AFL or AF recurrence prevention in patients with congenital heart disease who present with systemic ventricular dysfunction or hypertrophy, especially when catheter ablation has not been effective.^579,580^ Due to amiodarone's side effect profile, caution should be exercised when using it in patients with cyanotic congenital heart disease, low birth weight, liver, lung, or thyroid disease, or a prolonged QT interval. These conditions may increase the risk of medication-related complications.^581,582^

In the long term, catheter ablation is generally considered the first option in patients with AFL and congenital heart disease, especially when it is technically feasible and the arrhythmogenic substrate is well defined. However, although acute success is high, recurrence rates, especially in atypical AFL cases, are also high in this population, reaching up to 50%.^583-585^ When scheduling ablation in patients with complex congenital heart disease, especially those with Fontan or complex anomalies, imaging examinations of the anatomy must be performed, such as computed tomography and electroanatomical mapping systems.^586-588^ Furthermore, in some cases it is necessary to puncture the tube to access the venous atrium, and the electrophysiologist must be familiar with this method of access.^589,590^ For patients undergoing different surgeries, knowledge of the involved technique is also essential when concomitant ablation is scheduled (Box 19).

**Box 19:** Recommendations on treating intra-atrial reentrant tachycardia and atrial fibrillation in adults with congenital heart disease

Recommendations	Class of recommendation	Level of evidence
In patients with symptomatic and recurrent intra-atrial reentrant tachycardia and low-complexity congenital heart disease catheter ablation should be performed; antiarrhythmic drugs are preferable for long-term rhythm control.	**I**	**C**
In patients with symptomatic and recurrent intra-atrial reentrant tachycardia and moderate to high-complexity congenital heart disease, catheter ablation may be considered, provided that it is performed in specialized centers.	**IIa**	**C**
Catheter ablation or AF surgery (Cox-maze procedure) should be considered in patients with interatrial septal disease who are undergoing percutaneous or surgical correction, respectively.	**IIa**	**C**
Oral anticoagulation should be considered for all adults with AF or AFL and congenital heart disease who have had previous intracardiac corrective surgery, cyanosis, and previous Fontan or systemic right ventricle operations, regardless of CHA_2_DS_2_-VA score. In adults with AF or AFL and other congenital heart disease, oral anticoagulation should be considered for CHA_2_DS_2_-VA scores ≥ 2.	**I**	**C**

AF: atrial fibrillation; AFL: atrial flutter.

### 11.10. Atrial fibrillation in Women

It is estimated that 29.4 million women worldwide have AF. Although the incidence is higher among men, a greater number of older women have AF due to their longer life expectancy.^17,22^ Compared to men with AF, women are older, have a higher prevalence of hypertension, valvular disease, and HFpEF, and a low prevalence of ischemic heart disease.^591^ Women have distinct risk predictors for AF. A study of 34,221 women (1.8% AF) with 12.4 mean years of follow-up found that hypertension, particularly systolic hypertension, was a predictor of AF risk among initially healthy middle-aged women.^592,593^ The LEGACY study demonstrated that weight loss and maintenance are associated with sinus rhythm maintenance and a significant reduction in AF burden in both sexes.^66^ Body mass index was found to be linearly associated with incident risk of AF in a large group of women, which demonstrates that obesity is another important risk marker among women; dynamic weight change are also deleterious.^594^ Women who engage in vigorous exercise have a 28% lower AF incidence, although few such women were found in a large cohort. Prospective data suggest that moderate regular exercise is associated with lower AF risk in initially healthy middle-aged women, particularly when adjusted for body mass index.^595^ An increased incidence of AF has been found among healthy middle-aged women who consumed ≥ 2 drinks per day, with heavy alcohol consumption being a predictor of AF among women. In a prospective study of 30,034 women, menopause was not significantly related to AF incidence, while estrogen monotherapy was associated with higher risk, which suggests a pathophysiological link between estrogen exposure and arrhythmia in women. Compared to nulliparous women, a greater number of pregnancies was associated with a linear increase in AF risk in a large cohort of initially healthy women. Repetitive exposure to hormonal, metabolic, and physiological changes during pregnancy may predispose women to AF in later life.^596^ A study with a mean follow-up of 20.6 years found that women with AF who developed HF had higher all-cause mortality, cardiovascular mortality, and myocardial infarction. In a multivariate analysis of this population, control of systolic hypertension (systolic blood pressure < 120 mm Hg), obesity (body mass index < 30 kg/m^2^), smoking, and diabetes mellitus were associated with a progressive reduction in HF risk.^597^

Histological studies show that women with long-standing persistent AF have more fibrosis than women without AF, which is not always the case among men. This sex difference is due to different expression of genes and proteins that cause fibrotic remodeling.^598^ Magnetic resonance imaging and fibrosis quantification through delayed enhancement has confirmed that older women have more fibrosis than men in the same age group. These sex differences are even more pronounced regarding stroke, which suggests that sex plays a more pronounced role in atrial fibrotic remodeling and subsequent stroke among women.^599^

Several studies have examined the association between sex and stroke risk in AF, finding that women have a 20% to 30% higher risk than men, even after adjusting for stroke risk factors, anticoagulation, and age. However, it was recently proposed by the European Society to remove female sex from the CHADS-VA score, as the anticoagulation recommendation criteria were not different. This modification was also implemented in this guideline.

DOAC studies have generally included 35% to 40% women. A meta-analysis on sex differences in the residual risk of stroke and major bleeding in patients with non-valvular AF treated with OAC found that, compared to men, women have a higher risk of stroke and systemic embolism when treated with warfarin and a lower risk of major bleeding with DOACs. No significant sex differences were found regarding stroke risk or systemic embolism risk in DOAC therapy or in major bleeding risk in warfarin therapy. These results suggest that DOACs offer greater clinical benefit than warfarin for women with AF.^600^ For example, all-cause mortality was lower and intracranial hemorrhage risk was significantly lower among women treated with DOACs than those treated with warfarin.^601^

Women with AF have more symptoms and lower quality of life than men.^120^ According to the ORBIT trial's quality of life subanalysis (4293 patients; 42% women), women with AF were older, had a higher median CHA_2_DS_2_-VA score, and only 32.1% were asymptomatic (vs 42.5% of the men). They had lower quality of life scores, including greater limitations in daily activities and greater concern about treatment, although most were in sinus rhythm at the time of assessment.^602^

Despite being more symptomatic, women receive less rhythm control than men. Women undergo ablation and electrical cardioversion less frequently and atrioventricular junction ablation and pacemaker implantation more frequently than men.^603^ Additionally, women receive less treatment with class III AADs, and, due to electrophysiological characteristics specific to their sex, ie a longer baseline QTc interval, women are less tolerant of drugs that prolong it.^604^

Catheter ablation of AF improves symptoms, reduces mortality from HF, reduces hospitalization and stroke, and improves cognitive function in patients with AF. In general, women are less likely to undergo AF ablation and, when they do, it is later.^605^ They present more complex pre-ablation clinical findings and have a higher recurrence rate,^606^ a higher prevalence of extra-pulmonary venous foci,^607^ and higher rates of procedure-related complications.^608,609^ Regarding mortality, the Framingham study found no mortality differences between men and women with AF.^610^

Therefore, this guideline recommends that, regardless of sex, treatment should be directed towards reducing the risk of thromboembolic events, controlling rhythm, and improving quality of life. Studies that better assess sex differences in AF presentation and treatment are needed.

### 11.11. Atrial Fibrillation in Patients with Implantable Electronic Cardiac Devices

#### 11.11.1. Detection

CIED, which store electrode-recorded electrogram data, are programmed with algorithms that automatically interpret it, allowing tachyarrhythmia diagnosis. AF detection through CIED recordings may or may not be related to symptoms, 12-lead ECG results, or previous embolic events. However, the recordings require careful evaluation by a specialized physician to discriminate false events due to oversensing in the atrial channel. However, once the recorded episodes have been confirmed, the AF burden and the duration of true episodes can be quantified, which are related to the patient's overall risk.^130^

Studies that evaluated the relationship between the density of atrial arrhythmias detected on ECG and thromboembolic events point to a higher risk in patients who experience events lasting longer than 5 minutes. The total burden recorded continuously is proportionally related to the risk of embolic events: the longer the duration of AF episodes recorded, the higher the relative risk.^611^

#### 11.11.2. Programming Adjustments

Artificial cardiac pacing may be necessary in several AF scenarios. Patients with bradyarrhythmia who require pacemaker placement may develop AF over the course of follow-up, while patients with AF may also have bradyarrhythmias due to sinus node dysfunction, and atrioventricular node dysfunction (slow ventricular response), or to negative chronotropic effects from AAD therapy. However, atrial pacing appears to neither increase^612^ nor decrease the incidence of AF.^613^

In patients with dual-chamber pacemakers, the DDD or VDD modes are inadequate during AF episodes because they allow high rates for the ventricles. In patients who require rate support due to atrioventricular block, support must be provided in the inhibitory mode during sustained AF episodes, ie, the minimum pacing rate without monitoring high atrial rate (VVI, DDI). Automatic mode switching provides optimized support in patients with paroxysmal episodes because it switches to DDIR mode as soon as the algorithm detects a persistently high atrial rate. The algorithm allows a return to DDD mode after arrhythmia reversal is detected through atrial rate monitoring.^294^

Inadequate AF detection may be caused by sensitivity problems, retrograde ventriculoatrial conduction, or adjustment of the atrial refractory period after QRS detection. Likewise, inadequate pacing during the atrial refractory period may trigger atrial arrhythmia.^614^

Attention should be given to patients with interatrial conduction disorders, especially those with HF and resynchronization therapy. The suggested AV interval for these patients is generally short since it promotes biventricular pacing without fusion. Therefore, in patients with conduction delay due to Bachmann bundle block, activation of the LA (and thus left AV dyssynchrony) occurs, which leads to atrial pressure overload and a greater chance of AF. In patients with interatrial conduction disorders who are undergoing cardiac resynchronization therapy, it may be necessary to optimize AV interval programming by echocardiography.

#### 11.11.3. Cardioversion and Ablation

Patients with CIEDs may be candidates for electrical cardioversion, although the procedure's safety and the need for adjustment and evaluation after cardioversion are important issues. Biphasic defibrillators affect the efficacy of low-energy defibrillation and may provide greater safety for patients with CIEDs. Several case series have shown the safety of defibrillation in CIED patients. Concerns include battery damage, inappropriate defibrillator shock induction, abnormal inhibition, and electrode damage or displacement.^615^ In a retrospective analysis based on an extensive Danish national database, Elgaard et al. evaluated 2582 CIED patients who underwent electrical cardioversion and compared them with a matched sample of more than 12,000 patients. After 1 year, more interventions for generator replacement (HR 1.73) and electrode revision (HR 2.85) had occurred in the electrical cardioversion group. Although it was a retrospective analysis and the event latency after cardioversion was long, the authors concluded that greater attention is required for pacemaker or defibrillator patients who undergo electrical cardioversion.^616^

In patients with an CIED who undergo catheter ablation, electronic assessments should be performed before and after the procedure. In the initial telemetry, the recordings can be turned off so as not to unnecessarily record noise arising from the procedure. The detection and capture thresholds should be recorded so that the procedure cannot be considered the cause of any threshold increases. When assessing the intrinsic escape rhythm and the risk of asystole in patients dependent on cardiac pacing, asynchronous pacing modes allow RF to be safely applied, avoiding unnecessary inhibitions during the procedure. However, atrial asynchronous pacing should be avoided since it may impede activation map construction and atrial extrasystoles. For patients with implantable cardioverter defibrillators, ventricular arrhythmia therapies must be disabled, since the energy source may lead to improper detection and inappropriate shock. At the end of the procedure, the new telemetry will reveal any structural modifications and the new sensitivity and capture thresholds of the analyzed chambers, allowing electrograms recorded since the beginning of the procedure to be erased. HR monitoring, an essential part of the post-procedure results, can be performed remotely in most devices.

### 11.12. Atrial Fibrillation after Cardiac and Non-Cardiac Surgeries

AF can occur postoperatively in patients previously undiagnosed with arrhythmia.^617-619^ Although the mechanisms of postoperative AF are have not been completely defined, it is believed that triggers (eg, in the pulmonary veins) and anatomical and/or electrophysiological substrates are responsible for AF onset and maintenance. The postoperative period is a favorable environment for arrhythmia onset due to increased sympathetic tone, inflammatory factors, oxidative stress, and fluid and electrolyte disorders, especially hypomagnesemia.^620^

Despite current technological advances, the incidence of postoperative AF remains stable and affects prognosis and hospital length of stay, generating additional costs. Patients are at higher risk of stroke, myocardial infarction, hospitalization for congestive HF, and mortality, both during the immediate postoperative period and in the first 12 months after surgery.^621-623^ Approximately 90% of cases occur within 6 days of surgery. Postoperative AF occurs in 5-10% of non-cardiac surgeries and up to 55% in cardiac surgeries, depending on the surgery type and definition of AF, since some authors include any occurrence > 30 seconds, while others include only symptomatic episodes > 10 minutes (Box 20).

**Box 20:** Recommendations on patients with implantable electronic cardiac devices

Recommendations	Class	Level of evidence
Programming in AAI(R) is recommended for patients with paroxysmal AF and sinus node disease with preserved AV conduction who have an adequate ventricular response during episodes of paroxysmal AF.	**I**	**C**
Automatic Mode Switching programming is recommended for patients with paroxysmal AF when programmed in DDD/VDD mode.	**I**	**C**
The use of algorithms that promote intrinsic AV conduction search is recommended for patients with paroxysmal AF with DDD mode pacing and preserved AV conduction.	**IIa**	**B**
Programming in VVI(R) mode should be avoided in patients with sinus bradycardia or paroxysmal AF with an adequate ventricular response.	**III**	**C**

AF: atrial fibrillation; AV: atrioventricular; SSS: sick sinus syndrome; CIED: cardiac implantable electronic device.

#### 11.12.1. Predictive Factors

Predictors of postoperative AF can be assessed using clinical (cardiac or non-cardiac) and surgical (procedure type) data.

#### 11.12.2. Comorbidities and Epidemiological Data

Previous clinical history is essential in determining postoperative AF risk, with HF being one of the most important predictors. Yamashita et al. reported that HF can increase the occurrence of AF after cardiac surgery by up to 56%.^624^ Other important factors that increase AF risk include chronic obstructive pulmonary disease (36%), high blood pressure (29%), history of myocardial infarction (18%), and diabetes (6%). Advanced age, male sex, obesity, and LA enlargement are also correlated with postoperative AF.

##### 11.12.2.1. Surgical Predictors

The following surgical factors for postoperative AF stand out: surgery type (cardiac, non-cardiac thoracic, and non-cardiac non-thoracic), surgery time, the need for volume replacement, and surgical aggressiveness.

The incidence of AF after cardiac surgery also depends on surgery type. It can reach 20% after revascularization without extracorporeal circulation or > 50% after valve replacement.^625,626^ Cardiological catheter procedures, such as TAVI and percutaneous treatment of mitral insufficiency (such as MitraClip implantation), which have become more routine in clinical practice, are also associated with postoperative AF. Its occurrence is greater after mitral and tricuspid valve procedures than aortic valve procedures.

Several scores have been tested (alone or in combination) to identify patients at increased risk of AF after cardiac surgery.^627-629^ The risk of AF is lower in non-cardiac surgeries; its occurrence ranges from 0.5% in simpler surgeries, such as cesarean section, to 30% in more complex and invasive surgeries, such as colorectal surgery.^630^ The risk of AF after thoracic surgeries is 10-20%, falling between non-cardiac and cardiac surgeries, although it may be higher in more extensive surgeries, such as pneumonectomy, lung transplantation, and esophagectomy.

#### 11.12.3. Preoperative Prevention

The prevention of AF in patients who will undergo surgery is a key part of the treatment. Several drugs, both cardiological and non-cardiological, have been tested. Beta-blockers are recommended to reduce the incidence of AF, although current data indicate that they are discontinued in up to 25% of patients, mainly due to side effects, notably bradycardia and hypotension, and should be used with caution in heart surgery patients.^631^ Of note, a meta-analysis of 33 randomized trials found that the lower incidence of postoperative AF due to prophylactic beta-blocker therapy did not translate into a reduced stroke, mortality, or length of hospital stay.^632^ According to this meta-analysis, the best beta-blocker was carvedilol.

However, in non-cardiac surgeries, beta-blockers cause more risks than benefits and should not be used prophylactically. In a multicenter study (> 8000 patients), those randomized to metoprolol had higher mortality (33%) and twice as many strokes as the placebo group.^633^ In a meta-analysis of randomized trials (14,000 patients), beta-blockers reduced the incidence of postoperative heart attack and AF, but not mortality or stroke.^634^ Thus, they are not recommended as prophylactic treatment for non-cardiac surgeries (Box 21).

**Box 21:** Prevention and treatment of atrial fibrillation in the perioperative period of cardiac and non-cardiac surgery

Recommendations	Class of recommendation	Level of evidence
The use of amiodarone in the perioperative period is recommended when prevention of postoperative AF is needed in patients undergoing cardiac surgery.	**I**	**A**
Beta-blockers should not be used routinely for AF prevention in patients undergoing non-cardiac surgery.	**III**	**C**

There is evidence that prophylactic amiodarone reduces postoperative AF and hospital length of stay in cardiac surgery; however, these benefits do not translate into reduced mortality or stroke.^635-637^ They are recommended for cardiac surgeries and appear to provide greater benefits in doses > 3000 mg.^635^

Randomized trial results for colchicine have been controversial. The END-AF trial, which was prematurely terminated (resulting in low detection power), tested low doses of colchicine, finding that it does not reduce the incidence of AF after cardiac surgery.^638^ However, in the COPPS trial, 1 month of colchicine reduced AF incidence (12% vs 22%, p = 0.021) and hospital length of stay after cardiac surgery.^639^ Currently, colchicine is being investigated in the multicenter COP-AF trial, which is randomizing patients undergoing non-cardiac thoracic surgery. Its results may provide more accurate information about the drug's benefits.^640^

#### 11.12.4. Postoperative Atrial Fibrillation Treatment

As in non-postoperative AF, rate control and anticoagulation can be used alone or with an electrical or chemical cardioversion strategy. In cases of hemodynamic instability, immediate electrical cardioversion is recommended.^640^

Although there is evidence that short- and long-term prognosis is worse in patients with postoperative AF, studies have shown similar results for rhythm and rate control. However, more than 95% of these patients are in sinus rhythm when discharged from the hospital, which shows the transient nature of postoperative AF. The options for rate control or AF reversal in the postoperative period follow the same recommendations as non-postoperative AF, which have been discussed in a previous section.

## 12. Atrial Fibrillation and Cognitive Changes

Several observational studies have indicated that AF is an isolated risk factor for cognitive decline and dementia.^641-645^ Neurocognitive impairment can occur even if clinical strokes or silent cerebral infarcts cannot be defected in neuroimaging.^646^ Other known mechanisms include microinfarcts (caused by microembolisms) and microbleeds, which cannot be visualized by conventional imaging techniques. Furthermore, cerebral hypoperfusion due to atrial contraction loss and ventricular contraction variability are related to dementia onset, as is the pro-inflammatory state of AF patients treated with beta-blockers.^646^

In addition to vascular dementia, AF is associated with other types of dementia, such as Alzheimer's dementia^647^ and senile dementia.^644,647^ A meta-analysis found that AF is also associated with early-onset dementia (before 70 years of age),^647^ and that earlier AF onset was associated with an increased risk of dementia, Alzheimer's dementia, and vascular dementia.^648^

Studies have suggested that anticoagulation can reduce dementia incidence in patients with AF.^649^ DOACs appear to prevent cognitive impairment better than warfarin,^113,650^ which might be explained by its dosage and safety. The protective effect of warfarin is closely related to time in therapeutic range: values < 75% or supratherapeutic INR values are associated with a higher risk of dementia.^651^

The data on rhythm control strategies are conflicting. Catheter ablation appears to significantly reduce cognitive decline and dementia onset.^652-654^ Electrical cardioversion has been shown to promote improved cerebral flow,^655^ but further randomized trials are needed to understand the actual role of rhythm control in preventing dementia and cognitive decline. Figure 21 shows the association between AF and cognitive changes.

**Figure 21:** Association between atrial fibrillation and cognitive changes. Adapted from Diener et al.^646^ BMI: body mass index; BNP: β-natriuretic peptide; PAOD: peripheral arterial occlusive disease; CD: cognitive decline.

## 13. The Importance of Education for Patients with Atrial Fibrillation

A holistic and multidisciplinary view on the treatment of patients with AF is essential for better clinical outcomes. Educating patients, their families, physicians, and other health professionals is decisive for successful treatment. IMPACT-AF, a randomized international trial^160^ (2281 patients) found that multifaceted and multilevel educational intervention led to a > 300% increase in adequate OAC usage in patients with AF who were at increased risk of stroke and systemic embolism. The intervention was associated with a 52% reduction in thromboembolic phenomena, regardless of the anticoagulant. In addition to fewer ischemic events, bleeding rates did not increase, despite a significant increase in the proportion of anticoagulated patients. Thus, it is clear that passive and active education interventions for everyone involved are needed to overcome barriers to anticoagulation and therapeutic inertia, culminating in excellent treatment for these patients.

## 14. Cost-benefit Analysis and Appropriate Resource Use in Atrial Fibrillation

Health care financing is a challenging topic in all areas of medicine. In AF, a common cardiac arrhythmia with devastating effects on millions of people worldwide, careful cost-benefit analysis and efficient use of resources are needed. The analyses must cover the various stages of AF management: 1) screening and accurate diagnosis in a broad, continuously developing field (eg, implantable/wearable diagnostic devices, etc.); 2) prevention of thromboembolic events, including discussion of DOAC therapy and LAA occlusion; 3) the range of therapeutic possibilities for rhythm or rate control (AADs, various forms of ablation, and CIEDs); 4) risk factor control strategies, including health education initiatives. Unfortunately, neither global nor national cost-effectiveness studies are available for several of these steps.

Regarding the screening of AF, the STROKESTOP study randomized 27,975 patients with an average follow-up of 6.9 years. The study aimed to estimate the cost and effectiveness of population-based AF screening using clinical outcomes. The study demonstrated high effectiveness and cost savings with a 92.7% probability, indicating that a broad population-based strategy for screening AF in the elderly is cost-effective, and efforts should be made to expand diagnostic coverage.^656^ It is noteworthy that this study used a device with two daily ECG recordings for 14 days. Today, there are several wearable or implantable technologies that require specific cost evaluations in terms of both the benefits of screening and their effectiveness.^13,20^

From a Canadian health care system perspective, the costs and quality-adjusted life-years resulting from risk stratification were tested in a hypothetical cohort of AF patients at risk of stroke over 20 years. A precision medicine approach, ie, a combination of clinical variables (age, previous stroke, and bleeding) and biomarkers (ultrasensitive troponin, N-terminal pro-type B-natriuretic peptide, DGF-15, and hemoglobin) was compared to standard care. The precision medicine approach led to a 7% decrease in overall costs of and a 12% increase in quality-adjusted life-years. Due to the negative incremental cost-effectiveness ratio, the authors concluded that precision medicine is economically dominant and can generate cost savings.^657^

Anticoagulation based on the CHA_2_DS_2_-VASc risk score is an effective approach for preventing thromboembolic events in AF. Several studies in different countries have compared vitamin K antagonists with DOACs, finding that the vast majority of DOACs are cost effective. However, no such analysis has been performed in Brazil and, with the current licensing of patents, medication costs have reduced, making them more accessible for patients. Cost-effectiveness studies are important to guarantee continued universal access through the Brazilian Unified Health System.

Due to the variety of tools in the rhythm and rate control arsenal, it is difficult to perform specific cost-effectiveness studies. Ablation is more effective at controlling rhythm than antiarrhythmic medications, making it a first-line strategy. Based on a real-world economic assessment, a Korean study concluded that rhythm control with ablation for AF patients < 75 years of age was cost-effective, with a cost-effectiveness increase of USD 7,913 per quality-adjusted life-year.^658^ The CABANA trial found that AF ablation improves symptoms more effectively than AADs. The trial's cost-effectiveness analysis found an increase of approximately USD 58,000 per quality-adjusted life-year in the ablation group compared to the AAD group, which would be economically attractive in the USA.^659^ According to a cost-effectiveness sub-analysis of the German EAST-AFNET 4 trial, early rhythm control provides health benefits at a reasonable additional cost.^660^ However, only 25% of the sample underwent ablation, and the authors pointed out that further studies with a broad cost perspective are needed to assess different clinical subgroups, treatment types, and regions to clarify the issue. Therefore, in Brazil, where AF ablation is only performed as a supplementary procedure, cost-effectiveness analyses are important and should be encouraged.

A Brazilian cost-effectiveness study of AF ablation in the private health system (83 patients) found that in 10.7 (SD, 5.4) months of follow-up, the recurrence-free rate was 83.6%, reflecting a median monthly cost reduction of 63.5% (p < 0.001), ie, from BRL 286.00 to BRL 104.00 (Q1: BRL 57.00 Q3: BRL 232.00). This reduction encompassed both emergency and outpatient care.^661^

## Abbreviations

–AAD: Antiarrhythmic drug
–ACS: Acute coronary syndrome
–AF: Atrial fibrillation
–AFL: Atrial flutter
–AV: Atrioventricular
–CAD: Coronary artery disease
–CIED: Cardiac implantable electronic device
–CrCl: Creatinine clearance
–DOAC: Direct-acting oral anticoagulant
–ECG: Electrocardiography/electrocardiogram
–HF: Heart failure
–HFrEF: Heart failure with reduced ejection fraction
–HFpEF: Heart failure with preserved ejection fraction
–HR: Hazard ratio
–INR: International normalized ratio
–LA: Left atrium
–LAA: Left atrial appendage
–LVEF: Left ventricular ejection fraction
–OAC: Oral anticoagulation/anticoagulants
–OSA: Obstructive sleep apnea
–RF: Radiofrequency
–RR: Relative risk
–SAH: Systemic arterial hypertension
–TAVI: Transcatheter aortic valve implantation
–TTI: Time-to-isolation
–VKA: Vitamin K antagonists
